# Rediscovering Sodium Ionophores as Selective Agents for Lithium Recognition and Extraction

**DOI:** 10.1002/anie.3249075

**Published:** 2026-05-23

**Authors:** Jakub Narodowiec, Aleksandra Kazimierczak, Małgorzata Grela, Magdalena Ceborska, Magdalena Matczuk, Maja Morawiak, Kajetan Dąbrowa

**Affiliations:** ^1^ Institute of Organic Chemistry, Polish Academy of Sciences Warsaw Poland; ^2^ Faculty of Mathematics and Natural Sciences Cardinal Stefan Wyszynski University in Warsaw Warsaw Poland; ^3^ Chair of Analytical Chemistry Faculty of Chemistry Warsaw University of Technology Warsaw Poland

**Keywords:** coordination modes, host‐guest systems, ionophores, lithium, molecular recognition

## Abstract

Conventional brine evaporation leads to substantial lithium losses through the formation of lithium‐containing solid precipitates that accumulate as salt stockpiles. Increasing global demand for lithium calls for selective and scalable extraction strategies for such solid materials. Solid–liquid extraction (SLE) enables direct processing of lithium‐bearing solids without prior dissolution of the inorganic matrix; however, suitable molecular extractants are scarce and often synthetically complex. Here, we show that long‐known sodium‐selective ionophores display a clear preference for lithium in solution and function as lithium‐selective extractants under SLE conditions. Hosts **1a–h** are based on a modular scaffold accessible from catechol and N,N‐disubstituted haloacetamides and are readily prepared on a multigram scale (up to 58 g per batch). Lithium recognition proceeds through a hydration‐assisted binding mode, in which Li^+^ is coordinated by water, ether, and carbonyl oxygen donors. This enables effective transfer of hydrated lithium salts, as supported by ^1^H and ^7^Li NMR spectroscopy, ICP‐MS, colorimetric analysis, DFT calculations, and x‐ray crystallography. Under semi‐preparative SLE conditions, nearly all available LiCl is recovered from a ∼100 g multicomponent salt mixture, affording >95% LiCl purity with Li/Na, Li/K, and Li/Mg selectivities above 3000.

## Graphical abstract

Long‐classified sodium ionophores unexpectedly prefer lithium and act as efficient extractants in solid–liquid extraction (SLE). Readily prepared from catechol and haloacetamides, these hosts recover hydrated LiCl from multicomponent salt mixtures via a hydration‐assisted O···Li^+^ binding mode, enabling scalable lithium separation with Li/Na, Li/K, and Li/Mg selectivities >3000.

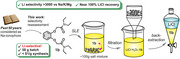

## 1 Introduction

**FIGURE 1:**
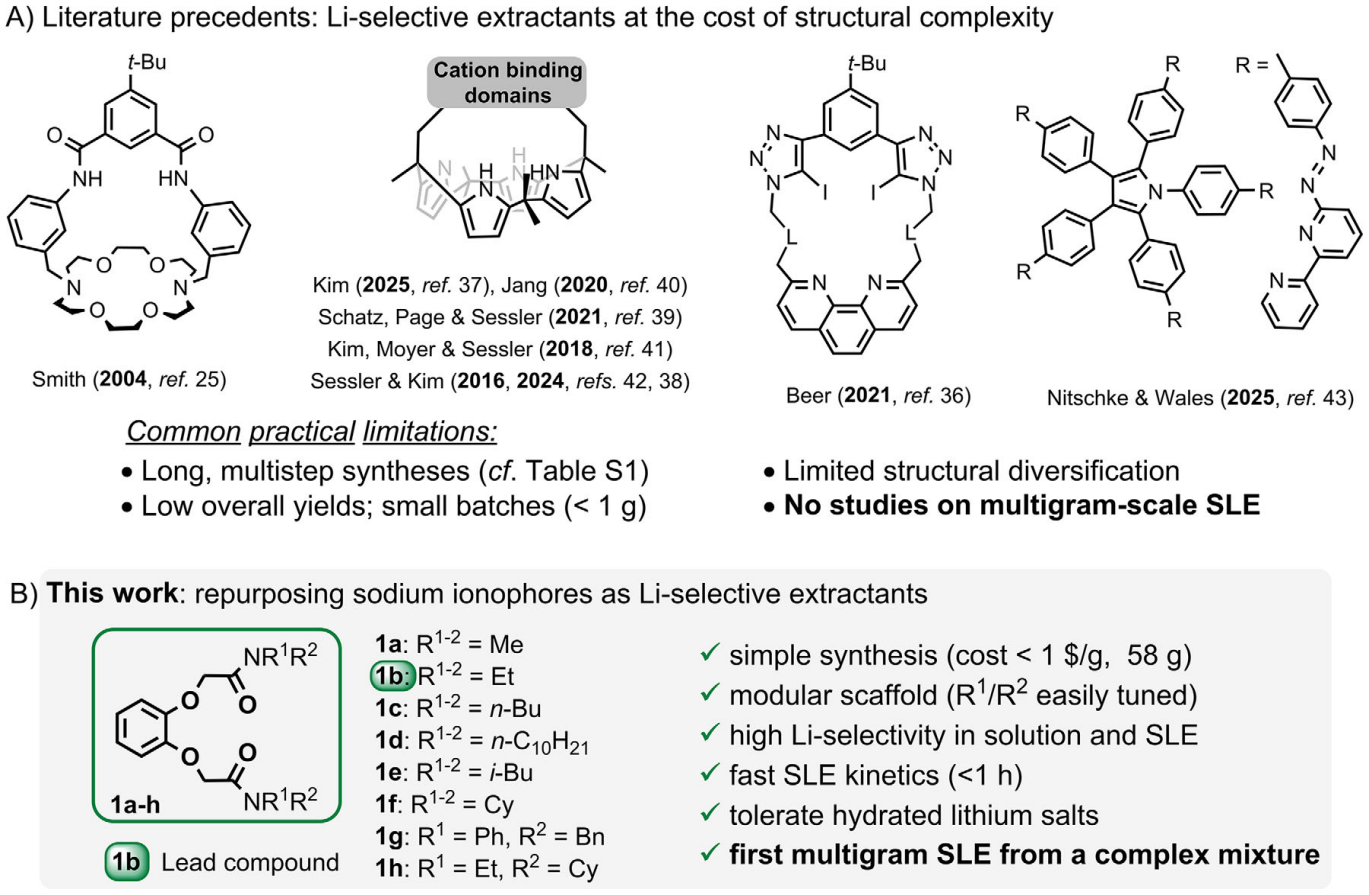
Supramolecular approaches to lithium SLE. (A) Representative receptors based on large, synthetically demanding architectures, generally limited to small‐scale studies. (B) This work: simple extractants **1a–h** derived from sodium ionophores, obtained by a short and scalable synthesis, and applied to lithium‐selective SLE from complex solid salt mixtures on a multigram scale.

The successful commercialization of lithium‐ion batteries (LIBs) in the early 90s has driven global demand for lithium, especially for use in electric vehicles and portable electronics. Conventional extraction methods, such as hard rock mining and solar evaporation from lithium‐rich brines, face several known limitations [1]. Hard‐rock extraction from spodumene ores, mostly mined in Australia and processed in China [2, 3], involves energy‐intensive thermal treatment and the use of aggressive chemicals [4, 5], while brine evaporation, used extensively in the Lithium Triangle in South America [6], often requires several months to years and typically recovers only about half of the lithium [7, 8, 9]. The remaining fraction is largely lost through co‐precipitation with Na, K, and Mg salts that are discarded and stored as salt stockpiles [10, 11, 12]. In parallel, direct lithium extraction (DLE) from aqueous phase [13, 14, 15, 16, 17, 18, 19], including sorption‐based [14, 15, 16, 17, 20], membrane‐based [16, 17, 18], and solvent extraction [14, 17, 19] approaches, has emerged as an important alternative to evaporative processing. However, solid lithium‐bearing materials remain underexplored feedstocks, and emerging recovery strategies for such materials include bioleaching [21] and solid–liquid extraction (SLE). In the latter approach, molecular extractants selectively transfer lithium salts from the solid phase into organic solvents, providing lithium in a form suitable for downstream processing and making this method particularly relevant to complex, lithium‐poor salt mixtures, including those generated on a large scale in potash and salt mining operations, which are typically not processed for lithium recovery [22, 23, 24]. A pioneering study on lithium‐selective SLE was reported by Mahoney and coworkers [25], who used a macrobicyclic receptor in CDCl_3_ to solubilize LiCl or LiBr from equimolar alkali metal halide mixtures, achieving limited Li/Na and Li/K selectivities. Building on this concept, several elegant lithium‐selective hosts have been developed in recent years [26, 27, 28, 29], including, among others [30, 31, 32, 33, 34, 35, 36], heteroditopic cryptand‐like calix[4]pyrroles [37, 38, 39, 40, 41, 42], and acyclic [43, 44, 45] and macrocyclic [46] cationophores. Despite their promising performance, they have remained confined to small‐scale studies, as they are typically accessible only in small batches from multistep syntheses (Figure 1A and Section S1.4). Moreover, most prior SLE studies relied on lithium‐to‐host ratios far above stoichiometric, which precluded exploration of efficient recovery of all or most lithium from solid mixtures. Substantial co‐solubilization of sodium, magnesium, or calcium salts has also been commonly observed.

## 2 Results and Discussion

To overcome the synthetic complexity and scalability limitations of previously reported lithium‐selective receptors, we sought simple, non‐macrocyclic extractants that could be prepared on a multigram, production‐relevant scale, making them suitable for practical large‐scale applications. Motivated by this goal, we computationally screened a series of de novo designed, oxygen‐rich binding motifs with low molecular weight and modular architecture. Surprisingly, the 1,2‐phenylenedioxydiacetamide cleft emerged among the top‐scoring candidates [47]. This motif is shared by commercial sodium ionophores II (ETH 157, **1g**) and III (ETH 2120, **1f**), which have been used in ion‐selective electrodes (ISEs) since the 1970s [48, 49, 50]. However, their designation as “*sodium‐selective*” is based entirely on ISE response [51]. To the best of our knowledge, their solution‐phase coordination chemistry has not yet been explored. The mismatch between ISE response and solution‐phase binding affinity has been previously observed [52]. It can be attributed to the specific environment in ISE membranes and to the complexity of ion‐binding and electrode‐response models. Therefore, here, we revisit the long‐known 1,2‐phenylenedioxydiacetamide backbone and demonstrate that it functions as a lithium‐selective extractant (**1a–h**), combining preparative‐scale accessibility with high selectivity and efficient extraction from lithium‐containing solid feedstocks (Figure 1B).

Compounds **1a–h** were obtained by O‐alkylation of catechol with halogenoacetamide derivatives in the presence of K_2_CO_3_ in 2‐MeTHF or acetone at room temperature, giving 65%–97% isolated yields (see Supporting Information). The resulting hosts displayed excellent solubility in chlorinated hydrocarbons; notably, **1d** and **1e** were also soluble in *n*‐heptane. We therefore first carried out binding studies in lipophilic CD_2_Cl_2_, but titration of **1b** with Li[B(C_6_F_5_)_4_] gave an almost stepwise isotherm, indicating very strong binding (*K* > 10^5^, Figures S6–S9). Therefore, to thoughtfully assess ion‐binding affinities and complexation stoichiometry, we performed ^1^H and ^7^Li NMR titrations in more competitive CD_3_CN with 0.5% H_2_O using triflate salts of Li^+^, Na^+^, and K^+^. Because all hosts displayed broadly similar titration profiles (Figures S10–S73), we selected **1b**, the simplest member of the series that exhibits strong binding, as a representative example for detailed discussion of the titration data (Figure 2).

**FIGURE 2:**
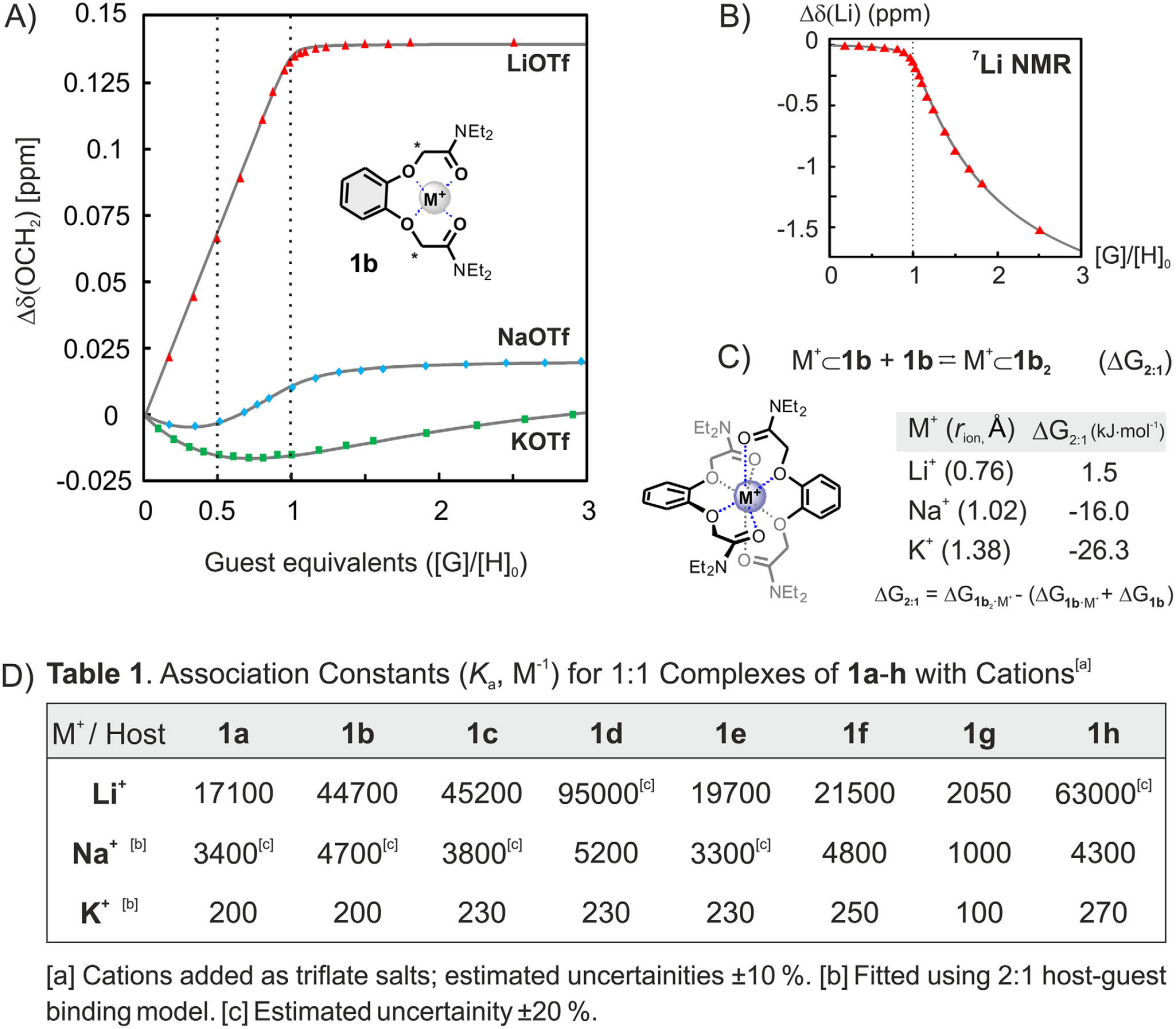
Solution binding properties of extractant **1b** and selected analogs in CD_3_CN + 0.5% H_2_O at 298 K. (A) Chemical shift changes (Δ*δ*(OCH_2_)) plotted against equivalents of metal triflates (MOTf): LiOTf (red), NaOTf (blue), and KOTf (green). (B) Corresponding ^7^Li NMR titration. (C) Proposed binding mode for Na^+^ and K^+^ involving formation of H_2_G complex; calculated Δ*G*_2:1_ and ionic radii (*r*_ion_) shown for comparison. (D) Association constants (*K*_a_) for 1:1 complexes of **1a–h** with alkali metal cations, determined by ^1^H and ^7^Li NMR titrations.

**FIGURE 3:**
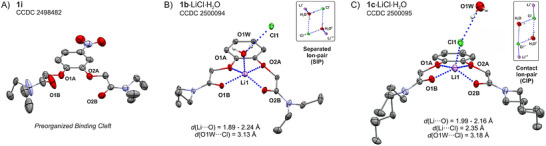
X‐ray crystal structures of **1i** (A), **1b**·LiCl·H_2_O (B), and **1c**·LiCl·H_2_O (C). **1i**, a nitro analog of **1b**, exemplifies a preorganized binding cleft, while the LiCl·H_2_O complexes illustrate a convergent O···Li^+^ coordination motif involving ether and carbonyl oxygen donors. Insets highlight the conserved tetrameric supramolecular anion–water clusters (Cl‐···H_2_O)_2_ that mediate ion pairing. Non‐acidic hydrogen atoms and disorder were omitted for clarity. Symmetry‐equivalent atoms are denoted by the # symbol. ORTEP representations shown at 50% probability level. Additional crystal structures (free **1f**, **1f**·LiOTf, **1i**·LiCl·H_2_O, and **1b**·LiBr·H_2_O) are provided in the Supporting Information.

Upon gradual addition of LiOTf, **1b** exhibited systematic downfield shifts across most resonances, with pronounced changes up to 1 equiv of guest, followed by a plateau, consistent with formation of a stable 1:1 complex. In contrast, titrations with NaOTf and KOTf exhibited non‐monotonic chemical shift changes, with an initial upfield shift of the same signals observed up to ∼0.5 equiv of guest, followed by a downfield shift, consistent with stepwise formation of 2:1 and 1:1 complexes and indicative of weaker, less specific interactions. The reduced affinity likely arises from the larger ionic radii of Na^+^ (1.02 Å) and K^+^ (1.38 Å) relative to Li^+^ (0.76 Å) and, therefore, their lower charge density. DFT calculations further indicate that 2:1 complexes are strongly favored for K^+^ and Na^+^ but not for Li^+^ (Figure S150), in line with the experimental binding isotherms. With quantitative information on solution‐phase ions binding in hand, we are able to shed new light on the performance of ISE membranes containing sodium ionophores II and III. The formation of complexes with multiple stoichiometries was shown to substantially alter selectivity and sensor behavior, offering an explanation for the observed differences between binding in solution and electrode performance [53, 54]. Consistent with this, all hosts in our series preferentially bound lithium over sodium and potassium in solution, with Li^+^/Na^+^ and Li^+^/K^+^ selectivity ratios ranging from 2.0 to 18 and 21 to 410, respectively. The strongest lithium binding was observed for host **1d** (*K*_a_ = 95000 M^−1^, R = *n*‐C_10_H_21_), representing a 5.5‐fold enhancement over **1a** (R = Me) and a two‐fold increase relative to **1b** (R = Et) and **1c** (R = *n*‐Bu). A clear trend emerged across **1a–d**, wherein elongation of linear alkyl chains led to improved lithium affinity, with strong correlation between *K*_a_ and experimentally determined log *P* (*r* ^2^ = 0.84) [55], likely due to enhanced hydrophobic interactions and improved preorganization of the binding cavity. In contrast, inclusion of all hosts eliminated this correlation (*r* ^2^ = 0.13), indicating that branched or cyclic substituents modulate lithium binding beyond lipophilicity alone, as exemplified by the reduced lithium affinity observed for **1e**, **1f**, and **1g**. Interestingly, the asymmetric host **1h** (*R* ^1^ = Et, *R* ^2^ = Cy) outperformed both **1e** and **1f** and slightly exceeded the performance of the smaller linear derivatives **1b** and **1c**, exhibiting the highest selectivity ratios in the series (Li^+^/Na^+^ = 18; Li^+^/K^+^ = 410), and suggesting that asymmetry can mitigate steric penalties. However, the precision of *K*_a_ and, thus, selectivity determination for **1h** was somewhat limited due to conformer interconversion. The weakest lithium binding and lowest selectivity were observed for **1g**, bearing phenyl and benzyl substituents (*K*_a_ = 2050 M^−1^; Li^+^/Na^+^ = 2.0; Li^+^/K^+^ = 21), which is consistent with steric congestion from the bulky aromatic groups and reduced carbonyl basicity. UV–vis titrations of **1a–h** with LiOTf and NaOTf in CH_3_CN + 0.5% H_2_O at 0.2 mM host concentration generally gave higher binding constants than NMR but confirmed Li/Na selectivities (Table S5). Together, these results show that subtle changes in the geometry, electronic properties, and steric bulk of N‐substituents can have a major impact on lithium recognition. To gain structural insight into these trends, we examined x‐ray crystal structures of selected hosts and their lithium salt complexes [56] (Figures 3 and S98–S104).

**FIGURE 4:**
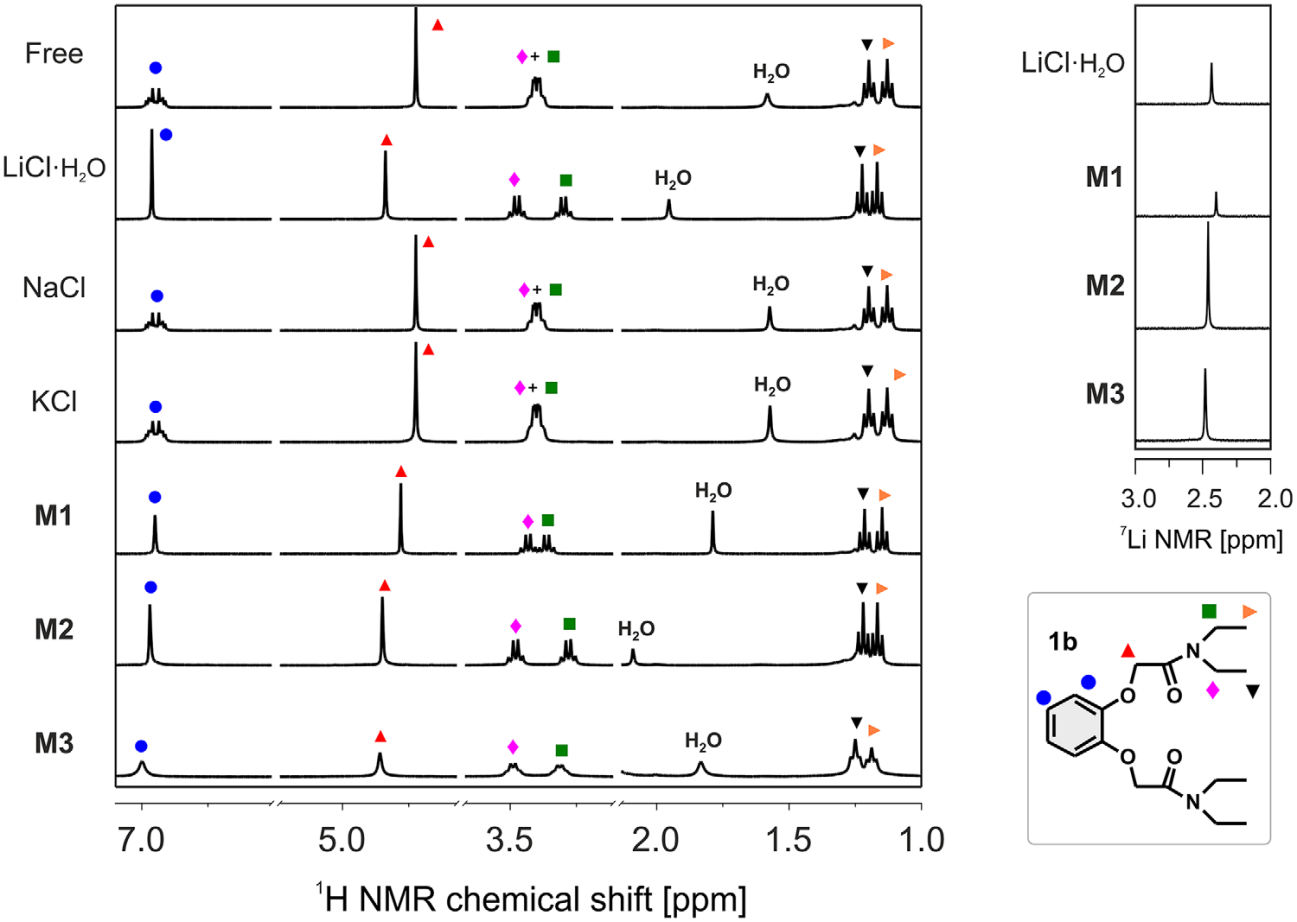
Stacked ^1^H and ^7^Li NMR spectra after SLE of alkali metal chlorides and mixtures **M1–M3** into a CDCl_3_ solution of **1b**.

Among the hosts, the structure of **1f** rationalizes its weaker lithium affinity in solution compared with **1b–1d** bearing linear alkyl chains: in its crystal structure, the binding cleft is blocked by bulky cyclohexyl substituents, both carbonyl arms point outward, and the donor atoms are misaligned, indicating a lack of preorganization (Figure S98). This collapsed conformation, likely stabilized by solvophobic effects in polar solvents [57] such as CH_3_CN/H_2_O, is consistent with the diminished binding observed for **1f**. In sharp contrast, in the structure of free **1i** (Figure 3A), a nitro analogue of host **1b**, the binding cleft is open, and all ether and carbonyl oxygen atoms are oriented inward, confirming host preorganization and a convergent array of coordination donors. Host **1i** was designed as a crystallizable analogue of **1b** by exploiting the crystallization‐promoting effect of nitro groups [58]. To further probe how structural differences influence metal binding, we analyzed x‐ray crystal structures of LiCl and LiBr complexes of **1i**, **1b**, and **1c** obtained by SLE of the corresponding salts into dichloromethane or 1,2‐dichloroethane (Figures 3B,C and S100–S103). Since LiCl·H_2_O and LiBr are essentially insoluble in these chlorinated organic solvents under SLE conditions in the absence of a host (Figure S107), the formation of these complexes provides direct evidence for lithium halide transfer into the organic phase. Notably, the lithium halide complexes **1b**·LiCl·H_2_O (Figure 3B), **1b**·LiBr·H_2_O (Figure S102), and **1i**·LiCl·H_2_O (Figure S100) share nearly identical five‐coordinate, approximately square‐pyramidal geometries, where Li^+^ is coordinated by four oxygen donor atoms and one water molecule (*d*(Li···O) = 1.94–2.24 Å). In these structures, the Cl^−^ and Br^−^ anions are not directly coordinated to lithium but instead form a hydrogen bond with the bridging water molecule (*d*(X^−^···H‐OH) = 3.21–3.38 Å), resulting in separated ion pairs in which Li^+^ defines the coordination environment and the halide adapts. A similar water‐mediated separation of lithium and halide ions has been observed in cryptand‐based systems [25, 40, 42, 59]. In contrast, in **1c**·LiCl·H_2_O, a direct Li···Cl contact (*d*(Li–Cl) = 2.35 Å) replaces the water‐bridged ion pairing, representing a rare example of a LiCl contact ion‐pair without any accompanying Li···OH_2_ interaction (Figure 3C). Notably, all **1b**, **1i**, and **1c** complexes share a conserved rhomboid tetrameric supramolecular motif involving two halide anions and two water molecules, [(X^−^···H_2_O)_2_ (X = Cl^−^, Br^−^)], as highlighted in Figure 3 insets and Figure S105, with the only difference being whether lithium coordinates to water (**1b** and **1i**) or directly to the halide (**1c**). The shift in anion positioning in the complex of the more lipophilic **1c** may help explain the positive correlation between lithium affinity and lipophilicity observed across the **1a–d** series. DOSY ^1^H NMR spectra of the **1b–d** LiCl complexes in CDCl_3_ solution (see Section S4.3), together with DFT calculations (Figure S151), indicate that water remains an integral, yet dynamically exchanging, component of the supramolecular assembly in the solution. A survey of the Cambridge Structural Database reveals that discrete chloride/water ternary clusters are known but remain rarely reported for lithium complexes [60, 61]. This observation suggests a design strategy for lithium receptors that accommodates hydrated LiX species, which lowers the enthalpic cost of lithium transfer by ∼75 kJ·mol^−1^ relative to anhydrous LiCl (see Supporting Information). In a related complex, **1f**·LiOTf, lithium maintains a similar five‐coordinate geometry, with the triflate anion bound directly to Li^+^ and no water present (Figure S104), giving a contact ion pair analogous to that in **1c**·LiCl·H_2_O. Across all lithium complexes, Li^+^ consistently adopts a five‐coordinate environment typical for neutral oxygen‐donor ligands [62]. Despite variations in anion and aromatic ring substitution, the coordination geometries and host conformations remain essentially conserved, underscoring the dominant role of lithium coordination in defining the host–guest structure. Taken together, the crystallographic and NMR data show that relatively subtle changes in host geometry and steric profile can markedly modulate lithium binding within an otherwise conserved coordination framework. Encouraged by the robustness of this platform, we next evaluated the applicability of hosts **1a–h** in lithium salt extraction. We focused on SLE from LiCl·H_2_O, NaCl, KCl, and from solid mixtures **M1–M3** into CDCl_3_. This solvent was chosen as a benchmark solvent to maintain continuity with prior SLE studies (Table S1) and to enable direct ^1^H and ^7^Li NMR characterization of the extracted species under identical conditions. **M1** and **M2** were designed to mimic primary lithium sources (salt deposits), while **M3** represented a secondary artificial resource, black mass derived from spent lithium iron phosphate (LFP) batteries. **M1** (LiCl/NaCl/KCl, 1425 ppm Li) simulates lithium‐poor sources such as Zabuye Lake in China [63], whereas **M2** (LiCl/NaCl/KCl/MgCl_2_, 6950 ppm Li) mimics typical brines from the Lithium Triangle region, which collectively supply about 50% of global lithium production [6]. ^1^H and ^7^Li NMR spectra (Figures 4 and S108–S125) recorded after SLE under reproducible conditions (vertical carousel mixing, 40 rpm, 25°C, 2 h) showed that solutions of **1a–h** in CDCl_3_ selectively transfer lithium salts from LiCl·H_2_O and from mixtures **M1–M3**.

**FIGURE 5:**
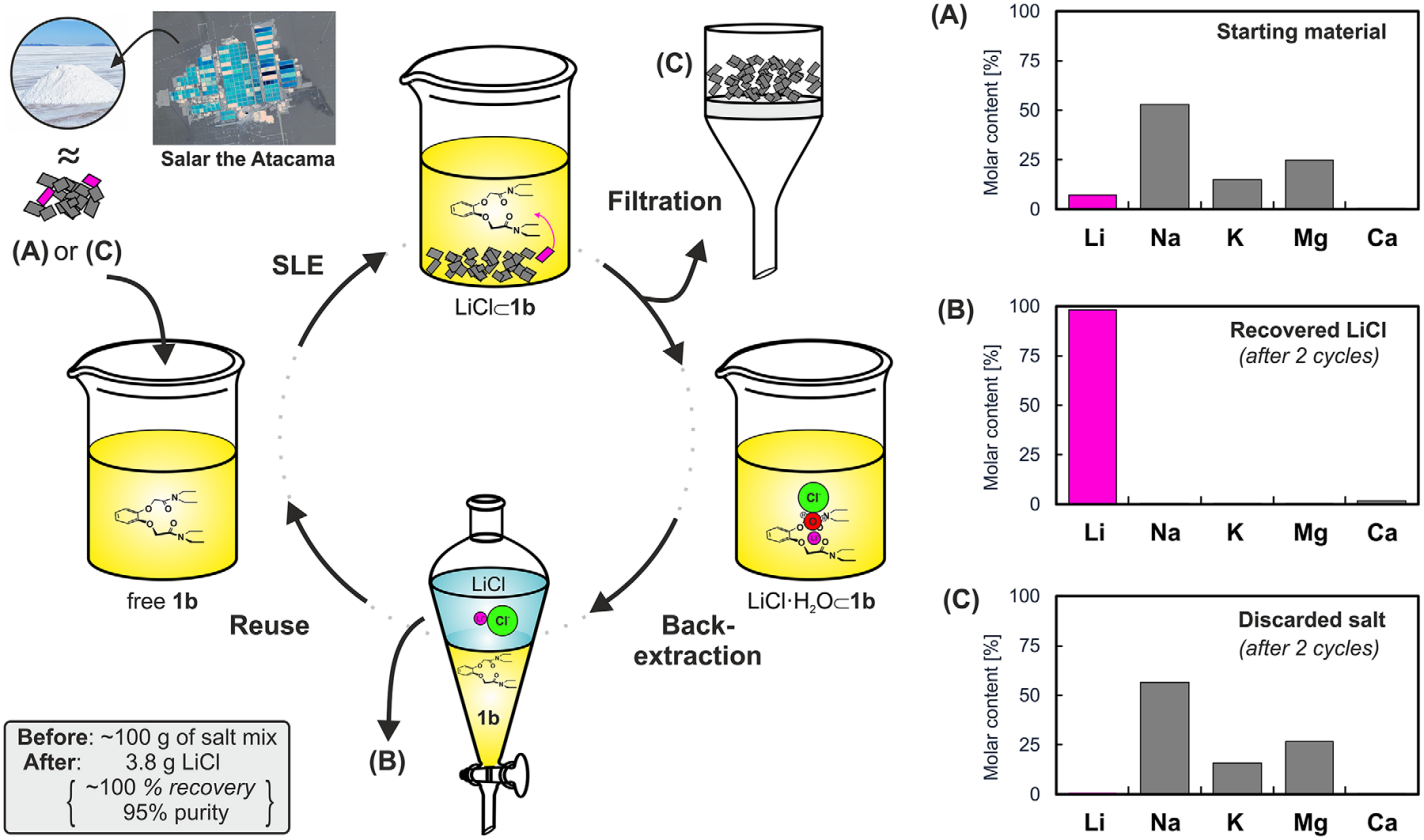
Preparative‐scale SLE using a multicomponent salt mixture (∼100 g) using **1b** in CH_2_Cl_2_ (33.6 g in 1 L, 0.10 M; 1.0 equiv of **1b** relative to LiCl), with near‐quantitative lithium recovery.

This was evidenced by substantial changes in ^1^H chemical shifts relative to free **1a–h** and the appearance of a sharp ^7^Li signal for all lithium‐containing samples, while spectra remained unchanged for NaCl and KCl alone; notably, SLE from LiCl·H_2_O in the absence of **1** did not lead to any detectable ^7^Li signal (Figure S107). For **M3**, ^19^F and ^31^P NMR spectra (Figures S112–S113) confirmed the presence of PF_6_^−^ as the counter anion, originating from the battery electrolyte and driving the extraction due to this anion's lipophilicity. As an initial qualitative measure of extraction performance, the chemical shift change of the methylene signal common to all hosts was compared for SLE of pure LiCl·H_2_O (Figure S126). The responses varied markedly, with the largest perturbations for **1b** and **1f**, suggesting that these compounds are the most efficient extractants under these conditions. The lithium binding strengths of **1a–h** determined in titration experiments did not correlate directly with their SLE performance, likely because these methods probe fundamentally different processes. For **M2**, **1f** showed minor additional resonances indicative of magnesium co‐extraction, whereas no such features were observed for **1b**, which was therefore selected for quantitative evaluation by ICP‐MS. Organic phases obtained after SLE with **1b** in CDCl_3_ were back‐extracted with distilled water, and the resulting aqueous samples were analyzed by ICP‐MS. In all cases, Na and K concentrations were below the detection limit, and for **M2,** less than 1 mol% of Mg relative to Li was detected, corresponding to Li/M selectivities > 10^5^ for Na and K and approximately 400 for Mg. For **M3**, besides lithium, pronounced extraction was observed only for copper (∼0.5 equiv relative to lithium), most probably originating from oxidized current collectors or cathode casing. Kinetic ^1^H NMR experiments showed that LiCl·H_2_O SLE with **1b** is fast, reaching equilibrium within 1 h at 25°C (Figure S139). Lithium uptake after 2 h of SLE under analogous conditions revealed essentially full saturation of **1b** with LiCl, with loadings of 93 ± 4% in CHCl_3_ and 103 ± 4% in CH_2_Cl_2_, as determined independently by spectrophotometric analyses using thorine [64]. Taken together, considering lithium binding strength, selectivity, SLE performance, and synthetic accessibility, host **1b** was selected for subsequent large‐scale extraction studies (Figures 5 and S144).

Its reusability was evaluated over three consecutive SLE cycles under two scenarios. In the first, the same portion of modified **M2** was used in all cycles, probing the effect of lithium depletion. In the second, fresh solid feed was used in each cycle, thus maintaining a constant lithium content. In both cases, **1b** losses were marginal (≤0.7% over three SLE cycles), with no detectable loss of activity, consistent with its high partition coefficient, *P*(CH_2_Cl_2_/H_2_O) = 910 ± 90 (see Supporting Information). Preparative‐scale SLE was then performed from ∼100 g of a modified **M2** mixture containing 1000 ppm CaCl_2_ and 1 L of 0.10 M **1b** in CH_2_Cl_2_ at 25°C for a total of 48 h (two 24 h cycles). The modified **M2** reproduces a metal‐ion profile characteristic of Salar de Atacama brine, the largest lithium‐producing salt flat in the Lithium Triangle [65, 66]. The organic phase obtained after SLE was back‐extracted with distilled water to strip the salt from **1b** and regenerate the extractant for the next cycle. The resulting aqueous phase was analyzed for metal content by ICP‐MS, and lithium was additionally quantified spectrophotometrically. In the first extraction, spectrophotometric analysis revealed an extraction yield of 64 ± 4% (ICP‐MS: 60 ± 6%). Recovered **1b** was then reused in a second extraction under identical conditions, giving a lithium loading of 26 ± 4% (ICP‐MS: 26 ± 3%). The combined lithium recovery yield from modified **M2** reached 95 ± 5% (ICP‐MS: 86 ± 9%). Li/M selectivities calculated from ICP‐MS analysis results were 4700, 5600, 3900, and 1.5 for M = Na, K, Mg, and Ca, respectively. LiCl content in dry mass was calculated as 98% in the fraction from the first extraction and 95% in the combined fractions from both extractions. The only significant impurity was CaCl_2_, consistent with a recent SLE study in which Ca^2+^ is also co‐extracted with Li^+^ [38]. Together, these results demonstrate that **1b** efficiently and selectively recovers LiCl, while NaCl, KCl, and MgCl_2_, present in large excess remain largely unextracted. This behavior underscores the potential of host **1b** for lithium recovery from complex salt stockpiles and related solid lithium‐containing residues. To probe the behavior of **1b** under biphasic conditions, liquid–liquid extraction (LLE) experiments were performed using 1.0 M aqueous solutions of LiCl, LiBr, LiOTf, LiNTf_2_, and an equimolar mixture of metal chlorides (M = Li, Na, K, Mg; 1.0 M each) (Figures S127–S129). ICP‐MS analysis showed only minimal lithium transfer into the organic phase for LiCl, LiBr, LiOTf, and the chloride salt mixture, whereas LiNTf_2_ was extracted efficiently, reaching near‐quantitative host loading according to ^1^H/^7^Li NMR and ICP‐MS. Thus, under aqueous biphasic conditions, lithium transfer is strongly counteranion‐dependent and becomes favorable only for sufficiently lipophilic anions. This is consistent with the absence of a dedicated anion‐binding motif in **1b**, which makes transfer of strongly hydrated anions such as chloride (Δ*H*_hyd_ = −361 kJ·mol^−1^) unfavorable, even though lithium extraction must also overcome even larger hydration enthalpy of Li^+^ (Δ*H*_hyd_ = −519 kJ·mol^−1^) [67]. Accordingly, the present system is suited to SLE rather than direct extraction from aqueous chloride brines. To further expand the functional scope of this scaffold, we synthesized, in three steps, compound **1j**, enabling colorimetric quantitation of Li^+^ in solution in the presence of an equimolar amount of Na or K with a detection limit of 0.5 µM and good accuracy (Figures S146–S147).

## 3 Conclusion

In conclusion, 1,2‐phenylenedioxydiacetamides, historically classified as sodium‐selective ionophores, constitute a readily accessible and modular platform for the selective extraction of lithium salts from solid lithium‐containing materials. The extractant series **1a–h** operates through a convergent O···Li^+^ coordination motif with hydration‐assisted binding, enabling transfer of hydrated lithium salts from multicomponent solid mixtures. Lithium recognition and selectivity were established by NMR titrations, ICP‐MS analysis, x‐ray crystallography, and DFT calculations. Under semi‐preparative SLE conditions, host **1b** enabled near‐quantitative recovery of LiCl from a multicomponent salt mixture. Further development toward aqueous biphasic extraction will require receptor designs that address anion‐transfer limitations. Overall, these results show that synthetically simple small‐molecule extractants can provide scalable alternatives to structurally complex macrocyclic‐ and cryptand‐like systems, while the modular scaffold offers a clear path for optimization toward other lithium salts and increasingly complex multi‐metal feed streams.

## Author Contributions

**Jakub Narodowiec**: investigation, writing – original draft, methodology, validation, visualization, writing – review and editing, formal analysis, data curation, conceptualization, funding acquisition. **Aleksandra Kazimierczak**: investigation. **Małgorzata Grela**: investigation. **Magdalena Ceborska**: investigation, formal analysis, data curation. **Magdalena Matczuk**: investigation, formal analysis, data curation. **Maja Morawiak**: investigation, data curation. **Kajetan Dąbrowa**: resources, supervision, conceptualization, investigation, funding acquisition, writing – original draft, visualization, validation, formal analysis, data curation, project administration, methodology, writing – review and editing.

## Notes

A patent application (EP25159602) covering part of the reported results has been submitted to the European Patent Office.

## Conflicts of Interest

The authors declare no conflicts of interest.

## Supporting information




**Supporing File 1**: anie72828‐sup‐0001‐SuppMat.pdf.

**Supporing File 2**: anie72828‐sup‐0002‐DataFile.xlsx.

**Supporing File 3**: anie72828‐sup‐0003‐DataSet.zip.

## Data Availability

The data that supports the findings of this study are available in the Supporting Information of this article.
