# Supplementary material for: Rediscovering Sodium Ionophores as Selective Agents for Lithium Recognition and Extraction
Source: Angew Chem Int Ed Engl. 2026 May 23;65(32):e3249075. doi: 10.1002/anie.3249075 (PMC13427242; doi:10.1002/anie.3249075)
Supplement: Supplementary file 1 — Supporing File 1: anie72828‐sup‐0001‐SuppMat.pdf. [file ANIE-65-e3249075-s002.pdf]

Supporting Information

Rediscovering Sodium Ionophores as Selective Agents for Lithium Recognition and Extraction

Jakub Narodowiec, Aleksandra Kazimierczak, Małgorzata Grela, Magdalena Ceborska, Magdalena Matczuk, Maja Morawiak, and Kajetan Dąbrowa *

Table of contents

1. Synthesis and characterization 2
1.1 General Remarks 2
1.2. Synthetic procedures 2
1.3. Estimation of the synthesis cost of **1b** 9
1.4 Synthetic accessibility and performance of known lithium-selective extractants 10
1.5 Determination of log*P* of **1a–h** 13
1.6 Determination of the partition coefficient of **1b** between CH_2Cl_2 and water 19
2. Binding studies 19
2.1 General remarks 19
2.2 Titrations Procedures 20
2.3 Data analysis and results 20
2.4 Titration data for **1a–h** and **B12C4** 23
3. Crystallographic data 66
4. Extraction Experiments 76
4.1 Preparation of solid mixtures **M1-M3** 76
4.2 Analytical scale SLE and LLE experiments 78
4.3 DOSY ^{1}H NMR measurements 90
4.4 Kinetics of $LiCl{\cdot}H_2O$ solubilization by **1b** 96
4.5 ICP-MS/MS analysis of samples after analytical scale SLE with **1b** 98
4.6 Large-scale experiments 99
4.7 Thermodynamic analysis of $LiCl{\cdot}H_2O$ vs LiCl solubilization 106
5. Characterization of spectrophotometric sensor **1j** 107
6. DFT calculations 109
7. NMR Spectra 114
8. References 130

1. Synthesis and characterization

1.1 General Remarks

Commercially available reagents were purchased from Sigma-Aldrich, Alfa Aesar, TCI, Ambeed, or Angene and used without purification as received unless otherwise stated. CH_2Cl_2 was purified by distillation, and HPLC-grade acetone was used. K_2CO_3 was ground in an agate mortar and dried at 300°C under vacuum. CH_2Cl_2 and chloroform used for SLE experiments were dried and deacidified by shaking with K_2CO_3, followed by filtration. Thin-layer chromatography was performed on silica gel 60 F254 plates (Merck). Compounds were purified using flash chromatography on silica gel 60 (230-400 mesh, Merck). The NMR spectra were recorded on Bruker Avance II 400 MHz (at 400 MHz, 100 MHz, 155.5 MHz for ^{1}H, ^{13}C NMR, and ^{7}Li spectra, respectively) or Varian VNMRS 600 MHz (at 600 MHz and 151 MHz for ^{1}H and ^{13}C NMR spectra, respectively) at 298 K with residual solvent peak as internal reference (δ = 7.26 ppm for ^{1}H NMR and δ = 77.16 ppm for ^{13}C NMR). For ^{7}Li NMR titration experiments, a custom-made lithium shift external reference (δ = 0.00 ppm) prepared by sealing the Teflon tube filled under vacuum with LiCl solution in D_2O was used. The splitting pattern of multiplets is described by abbreviations (s – singlet, d – doublet, t – triplet, q – quartet, dd – doublet of doublets, m – multiplet, c – covered signal, b – broad peak). *J* coupling constants values are reported in Hz. Mass spectra were measured on a Synapt G2-S HDMS (Waters) mass spectrometer equipped with an electrospray ion source and q-TOF type mass analyzer.

1.2. Synthetic procedures

All N,N-disubstituted 2-bromoacetamides **S1a**–**h**, used as precursors for hosts **1a**–**h**, were obtained on a gram scale from the appropriate secondary amine and bromoacetyl bromide according to General Procedure I (Scheme S1). Molecular extractants **1a**–**h** were obtained by double O-alkylation of catechol with the corresponding 2-bromo-N,N-acetamides **S1a**–**h** according to General Procedure II (Scheme S2). Reactions were carried out in 2-methyltetrahydrofuran (2-MeTHF) or acetone, as indicated, to give **1a**–**h** in good to excellent isolated yields. All products were characterized by ^{1}H and ^{13}C NMR spectroscopy and HRMS, as described below.

General Procedure I

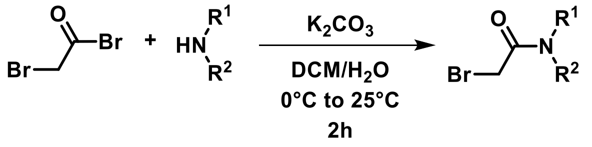

S1a: $R^{1,2}$ = Me **82%**
S1b: $R^{1,2}$ = Et **90%**
S1c: $R^{1,2}$ = *n*-Bu **94%**
S1d: $R^{1,2}$ = *n*-$C_{10}H_{21}$ **99%**
S1e: $R^{1,2}$ = *i*-Bu **98%**
S1f: $R^{1,2}$ = Cy **81%**
S1g: R^{1} = Ph, R^{2} = Bn **100%**
S1h: R^{1} = Et, R^{2} = Cy **90%**

Scheme S1. General synthetic route to N,N-disubstituted 2-bromoacetamides **S1a**–**h**.

A secondary amine (30.0 mmol, 1.00 equiv) was placed in a round-bottom flask equipped with a magnetic stir bar, and CH_2Cl_2 (30 mL) was added. The mixture was stirred until the amine dissolved completely. A solution of K_2CO_3 (6.22 g, 45.0 mmol, 1.50 equiv) in distilled water (30 mL) was then added. The reaction mixture was cooled to 0 °C in an ice bath, and bromoacetyl bromide (3.91 mL, 9.08 g, 45.0 mmol, 1.50 equiv) was added dropwise under vigorous stirring. The ice bath was removed, and the mixture was stirred for 2 h, allowing it to warm gradually to room temperature. The reaction mixture was then transferred to a separatory funnel, and the organic layer was separated. The aqueous phase was extracted with CH_2Cl_2 (2 × 20 mL). The combined organic extracts were dried over anhydrous Na_2SO_4, filtered, concentrated under reduced pressure, and dried under vacuum.

General Procedure II

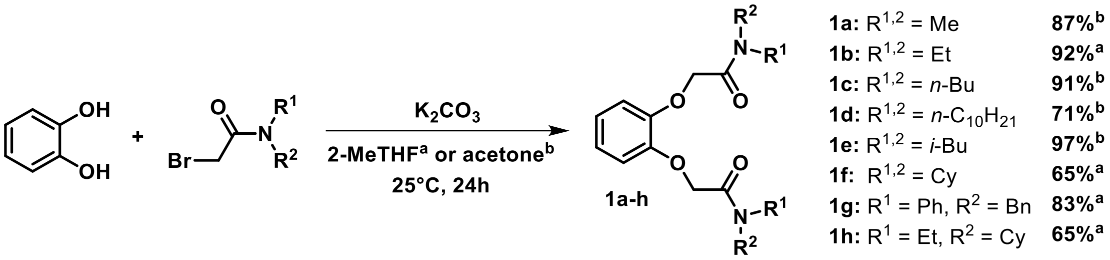

Scheme S2. General synthetic route to molecular extractants **1a**–**h**.

Catechol (1.10 g, 10.0 mmol, 1.00 equiv) and pulverized anhydrous K_2CO_3 (4.15 g, 30.0 mmol, 3.00 equiv) were placed under an argon atmosphere in a round-bottom flask equipped with a magnetic stir bar, and 2-MeTHF or acetone (20 mL) was added. The mixture was stirred for 5 min, then the respective 2-bromo-N,N-acetamide **S1a**–**h** (21.0 mmol, 2.10 equiv) was added in one portion, and the reaction was stirred overnight. The resulting suspension was filtered, and the remaining solid was washed with an additional portion of solvent. The combined filtrates were concentrated under reduced pressure, and the crude product was purified as described for each compound.

Compound S1a

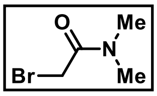

Compound **S1a** was synthesized according to a modified General Procedure I. Dimethylamine hydrochloride (2.45 g, 30.0 mmol, 1.00 equiv) was placed in a round-bottom flask equipped with a magnetic stir bar, and CH_2Cl_2 (30 mL) was added. A solution of K_2CO_3 (10.37 g, 75.0 mmol, 2.50 equiv) in distilled water (30 mL) was then added. The reaction mixture was cooled to 0 °C in an ice bath, and bromoacetyl bromide (3.91 mL, 9.08 g, 45.0 mmol, 1.50 equiv) was added dropwise under vigorous stirring. The ice bath was removed, and the mixture was stirred for 2 h, allowing it to warm gradually to room temperature. The reaction mixture was transferred to a separatory funnel, and the organic layer was separated. The aqueous phase was extracted with CH_2Cl_2 (2 × 20 mL). The combined organic extracts were dried over anhydrous Na_2SO_4, filtered, concentrated under reduced pressure, and dried under vacuum to afford the product as a yellowish oil (4.10 g, 82%). ^{1}H NMR data were in agreement with the literature values.[1]

Compound S1b

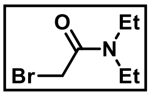

Compound **S1b** was synthesized according to General Procedure I on a 300 mmol scale. The product was obtained as a yellowish oil (52.6 g, 90%) without further purification. ^{1}H NMR data were in agreement with the literature values.[2]

Compound S1c

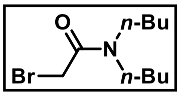

Compound **S1c** was synthesized according to General Procedure I on a 50 mmol scale. The product was obtained as a yellowish oil (11.8 g, 94%) without further purification. ^{1}H NMR data were in agreement with the literature values.[3]

Compound S1d

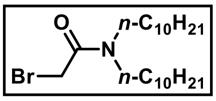

Compound **S1d** was synthesized according to General Procedure I on a 30 mmol scale. The product was obtained as a yellowish oil (12.5 g, 99%) without further purification. **^{1}H NMR** (400 MHz, $CDCl_3$) δ 3.81 (s, 2H), 3.33 – 3.21 (m, 4H), 1.64 – 1.45 (m, 4H), 1.35 – 1.16 (m, 28H), 0.86 (t, J = 6.8, 3H), 0.85 (t, J = 6.8, 3H). **^{13}C NMR** (100 MHz, $CDCl_3$) δ 166.30, 29.63, 29.62, 29.59, 29.57, 29.47, 29.38, 29.35, 29.25, 27.32, 26.94, 26.62, 22.76, 22.74, 14.18. **HRMS (ESI)** calculated for $C_{22}H_{44}NONaBr$ $[M+Na]^+$: 440.2504, found: 440.2507.

Compound S1e

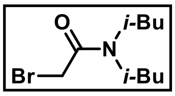

Compound **S1e** was synthesized according to General Procedure I on a 40 mmol scale. The product was obtained as a white oil (9.88 g, 99%) without further purification. ^{1}H NMR data were in agreement with the literature values.[4]

Compound S1f

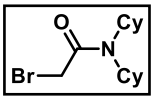

Compound **S1f** was synthesized according to General Procedure I on a 119 mmol scale. The product was obtained as a white powder (29.2 g, 81%) after crystallization from hot heptane. ^{1}H NMR data were in agreement with the literature values.[5]

Compound S1g

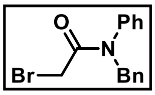

Compound **S1g** was synthesized according to General Procedure I on a 30 mmol scale. The product was obtained as a white oil (9.13 g, 100%) without further purification. ^{1}H NMR data were in agreement with the literature values.[6]

Compound S1h

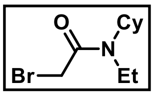

Compound **S1h** was synthesized according to General Procedure I on a 30 mmol scale. The product was obtained as a yellow oil (7.05 g, 95%) without further purification. **^{1}H NMR** (400 MHz, $CDCl_3$) δ 4.26 - 4.14 (m, 0.4H), 3.86 - 3.79 (m, 2H), 3.57 – 3.45 (m, 0.6H), 3.35 - 3.21 (m, 2H), 1.89 – 1.18 (m, 10H), 1.17 – 1.01 (m, 3H). **^{13}C NMR** (100 MHz, $CDCl_3$) δ 166.44, 165.82, 58.80, 54.71, 38.90, 37.13, 31.70, 30.58, 27.24, 27.16, 25.90, 25.86, 25.60, 25.29, 17.13, 14.37. (Note: two conformers stable on the NMR timescale). **HRMS (ESI)** calculated for $C_{10}H_{18}NONaBr$ $[M+Na]^+$: 270.0469, found: 270.0473.

Compound 1a

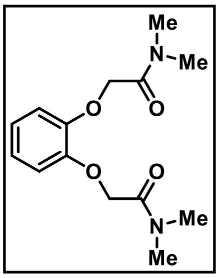

Compound **1a** was synthesized from **S1a** according to General Procedure II, using acetone as solvent. The crude product was purified by flash chromatography on silica gel (CH_2Cl_2/MeOH) to afford **1a** as a white solid (2.43 g, 87%). **^{1}H NMR** (400 MHz, $CDCl_3$): δ (ppm) 6.99–6.90 (m, 4H), 4.75 (s, 4H), 3.10 (s, 6H), 2.97 (s, 6H). **^{13}C NMR** (100 MHz, $CDCl_3$): δ (ppm) 168.27, 148.54, 122.82, 115.67, 69.02, 36.92, 36.07. **HRMS (ESI)** calculated for $C_{14}H_{20}N_2O_4Na$ $[M+Na]^+$: 303.1321, found: 303.1324.

Compound 1b

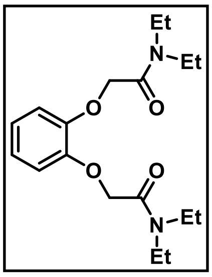

Compound **1b** was synthesized from **S1b** according to General Procedure II using 2-MeTHF as solvent. The crude product was purified by flash chromatography on silica gel (*n*-heptane/EtOAc) to afford **1b** as a pale yellow oil (3.09 g, 92%). **^{1}H NMR** (400 MHz, $CDCl_3$): 6.86–6.96 (m, 4H), 4.70 (s, 4H), 3.32–3.41 (m, 8H), 1.17 (t, J = 7.1 Hz, 6H), 1.10 (t, J = 7.1 Hz, 6H). **^{13}C NMR** (100 MHz, $CDCl_3$): 167.04, 148.32, 122.29, 115.19, 68.78, 41.50, 40.23, 14.30, 12.81. **HRMS (ESI)** calculated for $C_{18}H_{28}N_2O_4Na$ $[M+Na]^+$: 359.1951, found: 359.1947.

Large scale:

Catechol (27.5 g, 250.0 mmol, 1.00 equiv) and pulverized anhydrous K_2CO_3 (86.4 g, 625 mmol, 2.50 equiv) were placed under an argon atmosphere in a round-bottom flask equipped with a magnetic stir bar, followed by the addition of 2-MeTHF (500 mL). The mixture was stirred for 5 min, then compound **S1b** (99.5 g, 513 mmol, 2.05 equiv) was added in one portion, and the reaction was stirred overnight. The resulting suspension was filtered, and the solid was washed with an additional portion of 2-MeTHF. The combined filtrates were concentrated under reduced pressure and purified by short-pad chromatography on silica gel (*n*-heptane/EtOAc) to afford **1b** as a pale yellow oil (58.0 g, 69%).

Alternative procedure from 2-chloro-N,N-diethylacetamide:

Catechol (1.575 g, 14.3 mmol, 1.00 equiv), NaI (214 mg, 1.43 mmol, 0.10 equiv), and pulverized anhydrous K_2CO_3 (5.93 g, 42.9 mmol, 3.00 equiv) were placed under an argon atmosphere in a round-bottom flask equipped with a magnetic stir bar, followed by addition of 2-MeTHF (30 mL) and 2-chloro-N,N-diethylacetamide (4.49 g, 30.0 mmol, 2.10 equiv). The reaction mixture was stirred at 30 °C for 48 h. The resulting suspension was filtered, and the solid was washed with an additional portion of 2-MeTHF. The combined filtrates were concentrated under reduced pressure, and the residue was purified by flash chromatography on silica gel (*n*-heptane/EtOAc) to afford **1b** as a pale yellow oil (3.97 g, 83%).

Compound 1c

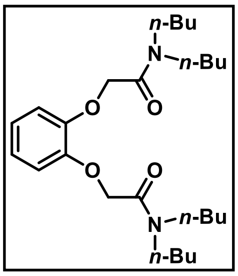

Compound **1c** was synthesized from **S1c** according to General Procedure II using acetone as solvent. The crude product was purified by flash chromatography on silica gel (*n*-heptane/EtOAc) to afford **1c** as a pale yellow oil (3.98 g, 89%). **^{1}H NMR** (400 MHz, $CDCl_3$): 6.97–6.86 (m, 4H), 4.73 (s, 4H), 3.30 (q, *J* = 7.0 Hz, 8H), 1.60 – 1.44 (m, 8H), 1.36–1.21 (m, 8H), 0.91 (t, *J* = 7.3 Hz, 6H), 0.89 (t, *J* = 7.3 Hz, 6H). **^{13}C NMR** (100 MHz, $CDCl_3$): 167.46, 148.45, 122.32, 115.39, 68.61, 47.07, 45.73, 31.15, 29.66, 20.27, 20.17, 13.94, 13.91. **HRMS (ESI)** calculated for $C_{26}H_{44}N_2O_4Na$ $[M+Na]^+$: 471.3199, found: 471.3202.

Compound 1d

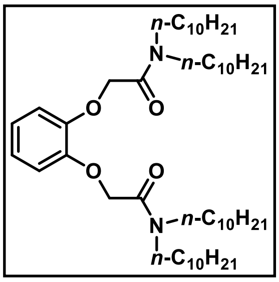

Compound **1d** was synthesized from **S1d** according to General Procedure II on a 7.58 mmol scale using acetone as solvent. The crude product was purified by flash chromatography on silica gel (*n*-heptane/EtOAc) to afford **1d** as a pale yellow oil (4.23 g, 71%). **^{1}H NMR** (600 MHz, $CDCl_3$): 7.01–6.88 (m, 4H), 4.77–4.70 (m, 4H), 4.24–4.15 (m, 1H), 3.72–3.63 (m, 1H), 3.36–3.26 (m, 4H), 1.84–1.02 (m, 26H). **^{13}C NMR** (151 MHz, $CDCl_3$): 167.52, 167.36, 167.25, 167.06, 148.71, 148.61, 148.42, 148.18, 122.42, 122.30, 122.27, 122.09, 115.81, 115.37, 115.26, 114.48, 69.36, 69.10, 68.94, 68.72, 57.16, 56.99, 54.84, 54.76, 38.02, 37.98, 36.93, 32.05, 32.02, 30.81, 26.05, 25.93, 25.71, 25.43, 25.41, 16.87, 14.83, 14.78. **HRMS (ESI)** calculated for $C_{50}H_{92}N_2O_4Na$ $[M+Na]^+$: 807.6955, found: 807.6948.

Compound 1e

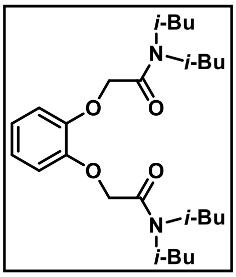

Compound **1e** was synthesized from **S1e** according to General Procedure II. The crude product was purified by flash chromatography on silica gel (*n*-heptane/EtOAc) to afford **1e** as a pale yellow oil (4.33 g, 97%). **^{1}H NMR** (400 MHz, $CDCl_3$): 6.98 – 6.85 (m, 4H), 4.77 (s, 4H), 3.19 (d, J = 7.7 Hz, 4H), 3.17 (d, J =7.8 Hz, 5H), 2.04–1.86 (m, 4H), 0.90 (d, J = 6.7 Hz, 12H), 0.80 (d, J = 6.7 Hz, 12H). **^{13}C NMR** (100 MHz, $CDCl_3$): 168.37, 148.46, 122.33, 115.62, 68.69, 54.37, 52.66, 27.43, 26.23, 20.16, 20.09. **HRMS (ESI)** calculated for $C_{26}H_{44}N_2O_4Na$ $[M+Na]^+$: 471.3199, found: 471.3203.

Compound 1f

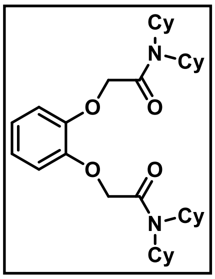

Compound **1f** was synthesized from **S1f** according to General Procedure II using 2-MeTHF as solvent. The crude product was purified by crystallization from hot EtOAc to afford **1f** as a white powder (3.59 g, 65%). **^{1}H NMR** (600 MHz, $CDCl_3$): 7.42 (dd, J = 7.6, 1.8 Hz, 1H), 7.22 (td, *J* = 7.8, 1.8 Hz, 1H), 6.96 (t, *J* = 7.4 Hz, 1H), 6.90 (d, *J* = 8.2 Hz, 1H), 4.68 (s, 2H), 4.64 (s, 2H), 4.15 (s, 2H), 3.57 (t, *J* = 11.2 Hz, 1H), 3.50 (t, *J* = 11.5 Hz, 1H), 2.93 (bs, 2H), 2.60–2.37 (m, 4H), 1.86–1.02 (m, 40H). **^{13}C NMR** (151 MHz, $CDCl_3$): 168.35, 167.15, 155.79, 128.98, 128.78, 126.45, 121.24, 111.56, 71.62, 69.58, 67.73, 57.81, 57.30, 56.47, 56.19, 34.25, 32.01, 31.50, 31.44, 30.02, 29.86, 29.14, 26.76, 26.68, 26.02, 25.97, 25.48, 25.41, 25.33, 22.82, 22.46, 14.24, 14.18. **HRMS (ESI)** calculated for $C_{34}H_{52}N_2O_4Na$ $[M+Na]^+$: 575.3825, found: 575.3830.

Compound 1g

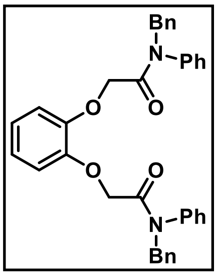

Compound **1g** was synthesized from **S1g** according to General Procedure II using 2-MeTHF as solvent. The crude product was purified by flash chromatography on silica gel (*n*-heptane/EtOAc) to afford **1g** as a white solid (4.60 g, 83%). **^{1}H NMR** (400 MHz, $CDCl_3$): 7.41 – 7.13 (m, 16H), 7.11 – 6.95 (m, 4H), 6.93 – 6.70 (m, 4H), 4.92 (s, 4H), 4.45 (s, 4H). **^{13}C NMR** (100 MHz, $CDCl_3$): 167.90, 148.48, 140.36, 137.06, 129.91, 129.11, 128.70, 128.51, 128.38, 127.64, 122.37, 115.91, 67.82, 53.34. **HRMS (ESI)** calculated for $C_{36}H_{32}N_2O_4Na$ $[M+Na]^+$: 579.2260, found: 579.2255.

Compound 1h

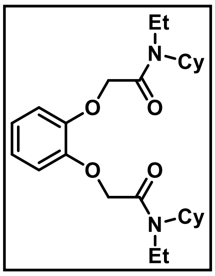

Compound **1h** was synthesized from **S1h** according to General Procedure II using 2-MeTHF as solvent. The crude product was purified by flash chromatography on silica gel (*n*-heptane/EtOAc) to afford **1h** as a yellowish oil (2.87 g, 65%). **^{1}H NMR** (600 MHz, $CDCl_3$): 7.01–6.88 (m, 4H), 4.77–4.70 (m, 4H), 4.24–4.15 (m, 1H), 3.72–3.63 (m, 1H), 3.36–3.26 (m, 4H), 1.84–1.02 (m, 26H). **^{13}C NMR** (151 MHz, $CDCl_3$): 167.52, 167.36, 167.25, 167.06, 148.71, 148.61, 148.42, 148.18, 122.42, 122.30, 122.27, 122.09, 115.81, 115.37, 115.26, 114.48, 69.36, 69.10, 68.94, 68.72, 57.16, 56.99, 54.84, 54.76, 38.02, 37.98, 36.93, 32.05, 32.02, 30.81, 26.05, 25.93, 25.71, 25.43, 25.41, 16.87, 14.83, 14.78 (Note: three conformers stable on the NMR timescale). **HRMS (ESI)** calculated for $C_{26}H_{40}N_2O_4Na$ $[M+Na]^+$: 467.2886, found: 467.2873.

Compound 1i

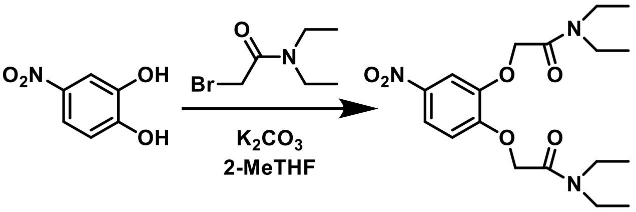

Scheme S3. Synthetic route to nitro analog **1i**.

4-Nitrocatechol (4.68 g, 30.2 mmol, 1.00 equiv) and pulverized anhydrous K_2CO_3 (12.5 g, 90.6 mmol, 3.00 equiv) were placed under an argon atmosphere in a round-bottom flask equipped with a magnetic stir bar, and 2-MeTHF (60 mL) was added. The mixture was stirred for 5 min, then **S1b** (12.8 g, 66.0 mmol, 2.19 equiv) was added in one portion, and the reaction was stirred overnight. The resulting suspension was filtered, and the solid was washed with an additional portion of 2-MeTHF. The combined filtrates were concentrated under reduced pressure, and the residue was purified by flash chromatography on silica gel (*n*-heptane/EtOAc) to afford the product as a yellowish solid (8.81 g, 77%). **^{1}H NMR** (400 MHz, $CDCl_3$): 7.86 (dd, J = 9.0, 2.6 Hz, 1H), 7.72 (d, J = 2.6 Hz, 1H), 6.97 (d, J = 9.0 Hz, 1H), 4.87 (s, 2H), 4.82 (s, 2H), 3.35 – 3.29 (m, 8H), 1.29 – 1.07 (m, 12H). **^{13}C NMR** (100 MHz, $CDCl_3$): 165.82, 165.71, 153.68, 147.88, 141.94, 118.41, 113.20, 109.61, 68.11, 67.63, 41.51, 41.43, 40.50, 40.45, 14.39, 14.34, 12.92, 12.86. **HRMS (ESI)** calculated for $C_{18}H_{27}N_3O_6Na$ $[M+Na]^+$: 404.1798, found: 404.1801.

Compound S2

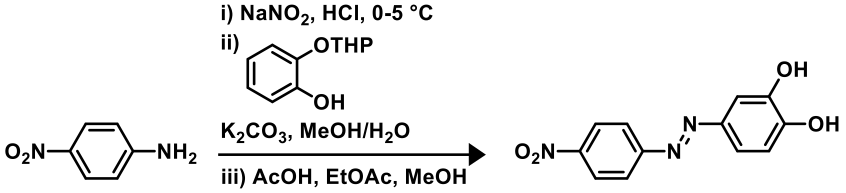

Scheme S4. Synthetic route to azobenzene precursor **S2**.

4-Nitroaniline (1.38 g, 10.0 mmol, 1.00 equiv) was placed in an Erlenmeyer flask, suspended in water (2 mL), cooled to 0 °C, and concentrated HCl (6 mL, 35–38%) was added dropwise. The resulting suspension was stirred for 5 min, then a solution of $NaNO_2$ (690 mg, 10.0 mmol, 1.00 equiv) in water (2 mL) was added dropwise. After completion of the addition, the mixture was stirred for an additional 15 min at 0 °C.

In parallel, 2-(2'-tetrahydropyranoxy)phenol (2.28 g, 10.0 mmol, ~85% purity, prepared according to the literature[7] was placed in a round-bottom flask, dissolved in MeOH (20 mL), cooled to 0 °C, and a solution of K_2CO_3 (2.76 g, 20.0 mmol, 2.00 equiv) was added under vigorous stirring. After 5 min, the cold diazonium salt solution was transferred dropwise under an argon stream to the flask containing 2-(2'-tetrahydropyranoxy)phenol, maintaining the temperature at 0 °C. Additional portions of solid K_2CO_3 (2.76 g, 20.0 mmol, 2.00 equiv each) were added after approximately ¼ and ½ of the diazonium solution had been transferred. After complete addition, the reaction mixture was stirred for a further 15 min at 0 °C, then concentrated HCl (6 mL) was added dropwise under an argon stream.

The resulting mixture was extracted with EtOAc (100 mL). The organic layer was collected, dried over anhydrous Na_2SO_4, filtered, and MeOH (10 mL) together with AcOH (2 mL) were added. The solution was stirred at room temperature overnight, concentrated in the presence of silica gel, and purified by flash column chromatography (*n*-heptane/EtOAc). The product was further purified by trituration with a 4:1 (v/v) MeOH/water mixture to afford a deep-red solid (1.14 g, 44%). ^1H NMR data were in agreement with the literature.[8]

Compound 1j

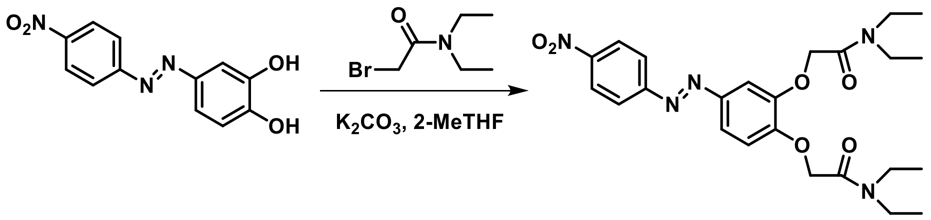

Scheme S5. Synthetic route to colorimetric lithium-selective sensor **1j**.

Compound **S2** (518 mg, 2.00 mmol, 1.00 equiv) and pulverized anhydrous K_2CO_3 (829 mg, 6.00 mmol, 3.00 equiv) were placed under an argon atmosphere in a round-bottom flask equipped with a magnetic stir bar, and 2-MeTHF (10 mL) was added. The mixture was stirred for 5 min, then 2-bromo-N,N-diethylacetamide **S1b** (970 mg, 5.00 mmol, 2.50 equiv) was added in one portion, and the reaction was stirred overnight. The reaction mixture was transferred to a separatory funnel with water and EtOAc. The organic layer was separated, and

the aqueous phase was extracted with EtOAc. The combined organic extracts were dried over anhydrous Na_2SO_4, filtered, and concentrated under reduced pressure. The crude product was purified by flash chromatography on silica gel (*n*-heptane/EtOAc) to afford the product as a red solid (700 mg, 72%). **^{1}H NMR** (400 MHz, $CDCl_3$): 8.35 (d, *J* = 9.0 Hz, 2H), 7.96 (d, *J* = 9.0 Hz, 2H), 7.69 (dd, *J* = 8.6, 2.2 Hz, 1H), 7.54 (d, *J* = 2.2 Hz, 1H), 7.10 (d, *J* = 8.6 Hz, 1H), 4.87 (s, 2H), 4.84 (s, 2H), 3.47 – 3.38 (m, 8H), 1.30 – 1.10 (m, 12H). **^{13}C NMR** (100 MHz, $CDCl_3$): 166.40, 166.38, 155.94, 152.20, 148.78, 148.43, 147.45, 124.81, 123.32, 122.80, 113.66, 105.34, 68.41, 68.05, 41.69, 41.67, 40.52, 40.51, 14.50, 14.45, 13.00, 12.95. **HRMS (ESI)** calculated for $C_{24}H_{31}N_5O_6Na$ $[M+Na]^+$: 508.2172, found: 508.2176.

1.3. Estimation of the synthesis cost of 1b

All prices are based on local vendor quotations as of the end of November 2025. The costs of labware, energy, and labour were not included, as they are strongly region-dependent and must be evaluated individually for each facility.

Substrates used for the synthesis of 52.6 g of **S1b**, with their cost:

1. Diethylamine (31.0 mL) – 0.76 USD
2. Bromoacetyl bromide (39.2 mL) – 24.5 USD
3. K_2CO_3 (62.6 g) – 0.61 USD

Total cost: 25.9 USD, 0.49 USD per 1 gram of **S1b.**

Substrates used for the synthesis of 58.0 g of **1b**, with their cost:

1. Catechol (27.5 g) – 2.08 USD
2. K_2CO_3 (86.4 g) – 0.86 USD
3. **S1b** (99.5 g) – 48.8 USD

2-MeTHF was fully regenerated by simple distillation of the filtered reaction mixture.
Total cost: 51.7 USD corresponding to 0.89 USD per gram of **1b**.

Substrates used for the synthesis of 3.97 g of **1b** with 2-chloro-N,N-diethylacetamide, with their cost:

4. Catechol (1.575 g) – 0.12 USD
5. K_2CO_3 (5.93 g) – 0.06 USD
6. Sodium iodide (0.214 g) – 0.04 USD
7. 2-chloro-N,N-diethylacetamide (4.49 g) – 0.52 USD

Total cost: 0.74 USD, corresponding to 0.19 USD per gram of **1b**.

1.4 Synthetic accessibility and performance of known lithium-selective extractants

As a benchmark for the system described herein, we surveyed the literature for lithium-selective receptors reported to enable SLE of lithium salts in organic media. Table S1 summarizes their synthetic accessibility (molar mass, number of synthetic steps, reported preparative scale, and overall yield) and outlines the type and scope of selectivity studies performed.

Overall, most reported receptors are synthetically demanding, requiring multiple steps and affording low overall yields, with even the highest reported preparative amounts typically corresponding to only a few millimoles of material due to high molar masses (*e.g.*, Table S1, entry 7). At the same time, SLE selectivity claims are frequently based on qualitative ^{1}H NMR analysis and simple equimolar mixtures of alkali metal chlorides. Quantitative datasets (ICP-MS, ICP-OES, ion chromatography) are comparatively scarce, commonly limited in scope, and often lack competition from divalent cations such as Mg^{2+} and Ca^{2+}. Where quantitative data are available, lithium selectivities are typically moderate, and extraction yields are low to moderate, despite the structural complexity of the receptors employed.

In contrast, this work introduces a set of lithium extractants that are easily accessible on a large scale and demonstrates ICP-MS-controlled, large-scale solid–liquid extraction (SLE) under competitive conditions, including substantial excesses of NaCl, KCl, and $MgCl_2$. Notably, this study provides a rare structure–activity relationship based on eight analogues **1a-h** with systematically varied substituents.

Table S1. Selected lithium extractants reported to enable SLE of lithium salts, together with their molar mass, reported preparative scale, overall yield, number of synthetic steps, and a brief summary of the selectivity study type (qualitative versus quantitative, solvent) and representative selectivity metrics (where available).

No	Molecular structure	MW [g·mol^{-1}]	Scale (total yield, number of steps)	Year	Ref.
1	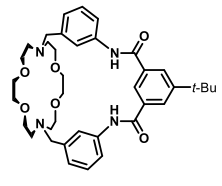	658.8	0.19 g (47%, 3 steps)	2004	(9)
		^{1}H NMR SLE into $CDCl_3$ solution from excess of LiCl/NaCl/KCl (1:1:1); moderate Li selectivity (Li/Na = 23.5, Li/K = 47).			
2	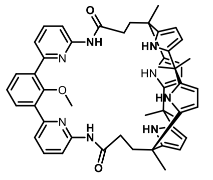	801.0	0.55 g (3.8%, 6 steps)	2016	(10)
		Qualitative ^{1}H NMR $LiNO_2$ SLE into $CDCl_3$ only; no quantitative selectivity data.			
3	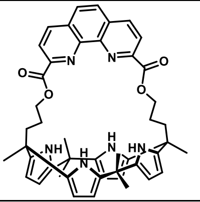 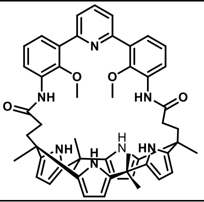	763.0<br>830.1	0.39 g (2.5%, 5 steps)<br>0.9 g (6.6%, 5 steps)	2018	(11)
		^{1}H NMR plus ICP-MS SLE from LiCl/NaCl/KCl mixtures into nitrobenzene-d_5 and $CDCl_3$; limited Li/Na selectivity under SLE conditions (Na/Li ~3 from equimolar mixture).[b]			
4	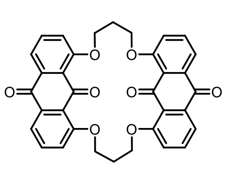	564.6	0.56 g (12%, 1 step) or<br>1.95 g (25%, 2 steps)	2019	(12)
		ICP-OES SLE from $LiCl/NaCl/KCl/MgCl_2/CaCl_2$ mixture into nitrobenzene-d_5; low Li/Na selectivity (~3) with significant K^+ and Ca^{2+} co-extraction, Li host loading ~4% when 5 equivalents of LiCl were used.			
5	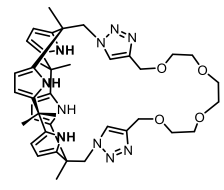	751.0	0.091 g (2.4%, 5 steps)	2020	(13)
		Qualitative ^{1}H NMR SLE from LiCl, NaCl, KCl, and their mixture into CD_2Cl_2; no quantitative analysis.			
6	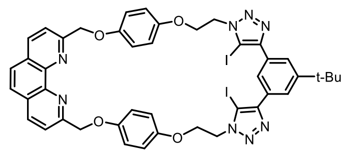	996.7	0.27 g (25%, 5 steps)	2021	(14)
		Selectivity evaluated only among LiX (X = Cl, Br, I) in $CDCl_3/CD_3CN$ mixture (3:1 v/v); no competition studies with other cations.			
7	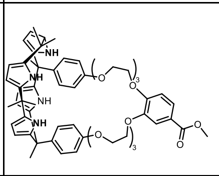 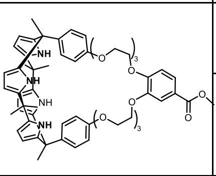	981.2<br>981.2	2.94 g (0.8%, 4 steps)<br>3.92 g (1.1%, 4 steps)	2021	(15)
		ICP-MS SLE from excess $LiCl/NaCl/KCl/MgCl_2/CaCl_2$; Li accounted for 17.5 wt% (nitrobenzene) or 30.5 wt% (MeCN) of extracted cations, with substantial co-extraction of other ions.			
8	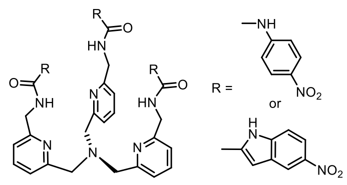	869.9<br>941.9	0.37 g (19%, 4 steps)<br>(19%, 4 steps)	2022	(16)
		^{1}H NMR study on the interactions in the solution; no extraction data.			
9	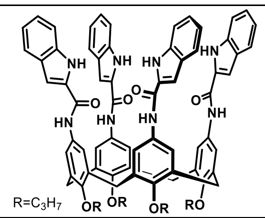	1225.5	0.5 g (20%, 4 steps)	2022	(17)
		Li selectivity inferred from ^{1}H NMR and ESI-MS after SLE from mixed MCl (M = Li, Na, K, Rb, Cs) into $CDCl_3$; no quantitative analysis.			

Table S1. (continued)

10	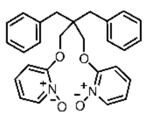	442.5	0.19 g (51%, 2 steps)	2023	(18)
		Qualitative ^{1}H NMR SLE from LiCl/NaCl/KCl and their mixture into $CDCl_3$; no quantitative selectivity data.			
11	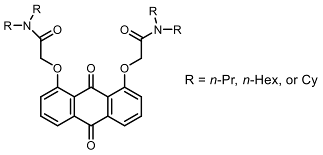	522.6<br>691.0<br>682.99	n/a [a]	2023	(19)
		^{1}H NMR and ICP-MS SLE from LiCl/NaCl/KCl mixtures into $CDCl_3$; good Li selectivity reported, Mg^{2+} and Ca^{2+} not studied.			
12	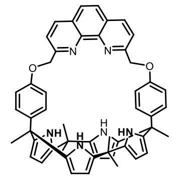	789.0	0.22 g (0.9%, 4 steps)	2024	(20)
		Qualitative ^{1}H NMR SLE from LiCl/NaCl/KCl/$MgCl_2$/$CaCl_2$ mixtures into CD_2Cl_2; no quantitative analysis.			
13	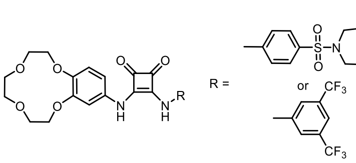	601.7<br>546.42	0.87 g (35%, 4 steps)<br>0.68 g (81%, 3 steps)	2024	(21)
		^{1}H NMR and ion chromatography SLE into CD_3CN and $CHCl_3$; moderate Li selectivity (Li/Na = 23, Li/K = 34) in $CDCl_3$, Mg^{2+} and Ca^{2+} not studied.			
14	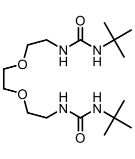	346.5	0.80 g (69%, 1 step)	2025	(22-24)
		^{1}H NMR SLE into $CDCl_3$ and CD_3CN, ICP-MS SLE into CH_3CN at 90 °C; strong competition from Mg^{2+} and limited reusability observed.			
15	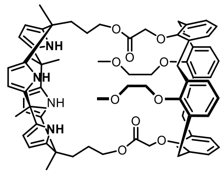	1151.5	0.14 g (1.9%, 6 steps)	2025	(25)
		Qualitative ^{1}H NMR SLE from MCl (M = Li, Na, K, Rb, Cs) and their mixtures; no quantitative analysis into $CDCl_3$ and CD_2Cl_2.			
16	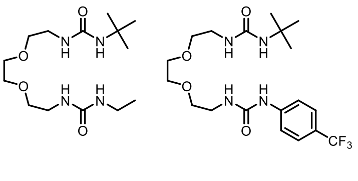	318.42<br>434.46	66% (2 steps)<br>65% (2 steps)	2025	(26)
		Qualitative ^{1}H NMR SLE into $CDCl_3$.			
17	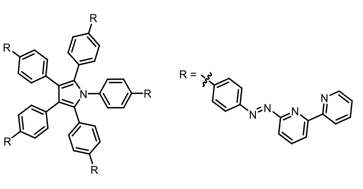	1739.0	0.09 g (47%, 4 steps)	2025	(27)
		Photoassisted $LiNTf_2$ separation from equimolar $MNTf_2$ (M = Li, Na, K) mixture; Li/Na = 12.5 and Li/K = 250.[c]			
18	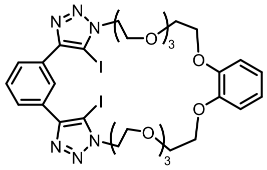	890.5	0.23 g (6.8%, 4 steps)	2026	(28)
		Low Li extraction yield (~3%) into $CHCl_3$; selectivity evidenced qualitatively by ^{1}H NMR, no quantitative data.			
19	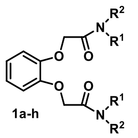 1a-h	280.3-785.3	2.43 g - 58 g (65 - 97%, 1-2 steps)	**This work**	
		Qualitative ^{1}H NMR and quantitative ICP-MS SLE at analytical (~0.1 g) and preparative (100 g) scale from LiCl/NaCl/KCl/$MgCl_2$/$CaCl_2$ mixtures with substantial excesses of Na^+ and Mg^{2+}; high Li purity in the extract was consistently observed, despite weaker Li/Ca selectivity.			

[a] Reported masses of substrates are inconsistent with the stated number of millimoles. Yield calculations based on product mass and either the number of millimoles or the substrate masses give values that differ substantially from those reported by the authors, so reliable yields cannot be assigned. [b] Analysis of the concentrations of the extracted metals, given in the Supporting Information of the original paper (Table S5), yields Na/Li mass ratios ranging from 2.8 to 4.9. [c] Although this host does not operate via SLE but rather through selective precipitation of the lithium complex, we decided to include it as a recent example of a supramolecular approach to lithium separation.

1.5 Determination of log*P* of 1a–h

The established RP-TLC strategy[29] was used to determine the lipophilicity parameter log*P* of compounds **1a**–**1h**. Silica gel 60 RP-18 TLC plates (Merck) were developed with dioxane/water mixtures as eluents, since dioxane, together with methanol, has previously been shown to be a superior modifier for this type of analysis.[30] Approximately 10 μL of acetonitrile solutions (10 mg/mL) of both reference compounds and **1a**–**1h** were applied to the TLC plates and developed with the selected solvent systems. The measured R_f values were converted to R_M according to the equation defined by Bate-Smith and Westall[31] (1):

$$R_M = log\left(\frac{1-R_f}{R_f}\right) (1)$$

The correlation between R_M and the percentage of dioxane in the mobile phase, c, was then obtained by linear regression according to the Soczewiński–Wachtmeister equation[32] (2):

$$R_M = R_M^0 + mc \ (2)$$

With the R_M^0 and m values determined for the reference set (Table S2, Figs. S1-S2), we evaluated linear correlations between these parameters and the literature log*P* values. A better correlation was obtained for R_M^0, described by $\log P = aR_M^0 + b$, with $r^2 = 0.943$, $a = 1.26$, and $b = 0.16$ (Figure S3). The precision of log*P* values calculated from this equation was estimated as ±0.6 (dashed lines), corresponding to 1.5 times the standard deviation. In the same manner, R_M^0 were then found for **1a-h**, and their log*P* were calculated (Table S3 and Figure S4).

Table S2. Fitted chromatographic parameters *m* and R_M^0 with r^2 or the set of reference compounds and their literature log*P* values ($\log P_{\mathrm{lit.}}$)

Compound	m	R_M^0	r^2	$\log P_{\mathrm{lit.}}$
Paracetamol	−1.5	0.2	0.976	1.1
Acetanilide	−2.2	1.0	0.992	1.2
Phenol	−2.3	1.2	0.996	1.5
Dimethyl Phthalate	−2.9	1.7	0.995	1.6
Indole	−3.4	2.1	0.997	2.1
Anthraquinone	−3.3	2.4	0.979	3.3
Naphthalene	−3.6	2.7	0.994	3.3
Diphenylamine	−4.1	2.9	0.985	3.5
Carbazole	−3.7	2.5	0.990	3.5
Di-*n*-butyl phthalate	−4.6	3.6	0.999	4.5
Pyrene	−4.1	3.2	0.998	5.1
Triphenylamine	−5.0	4.0	0.997	5.7
Di-*n*-octyl Phthalate	−7.4	6.4	0.987	8.1

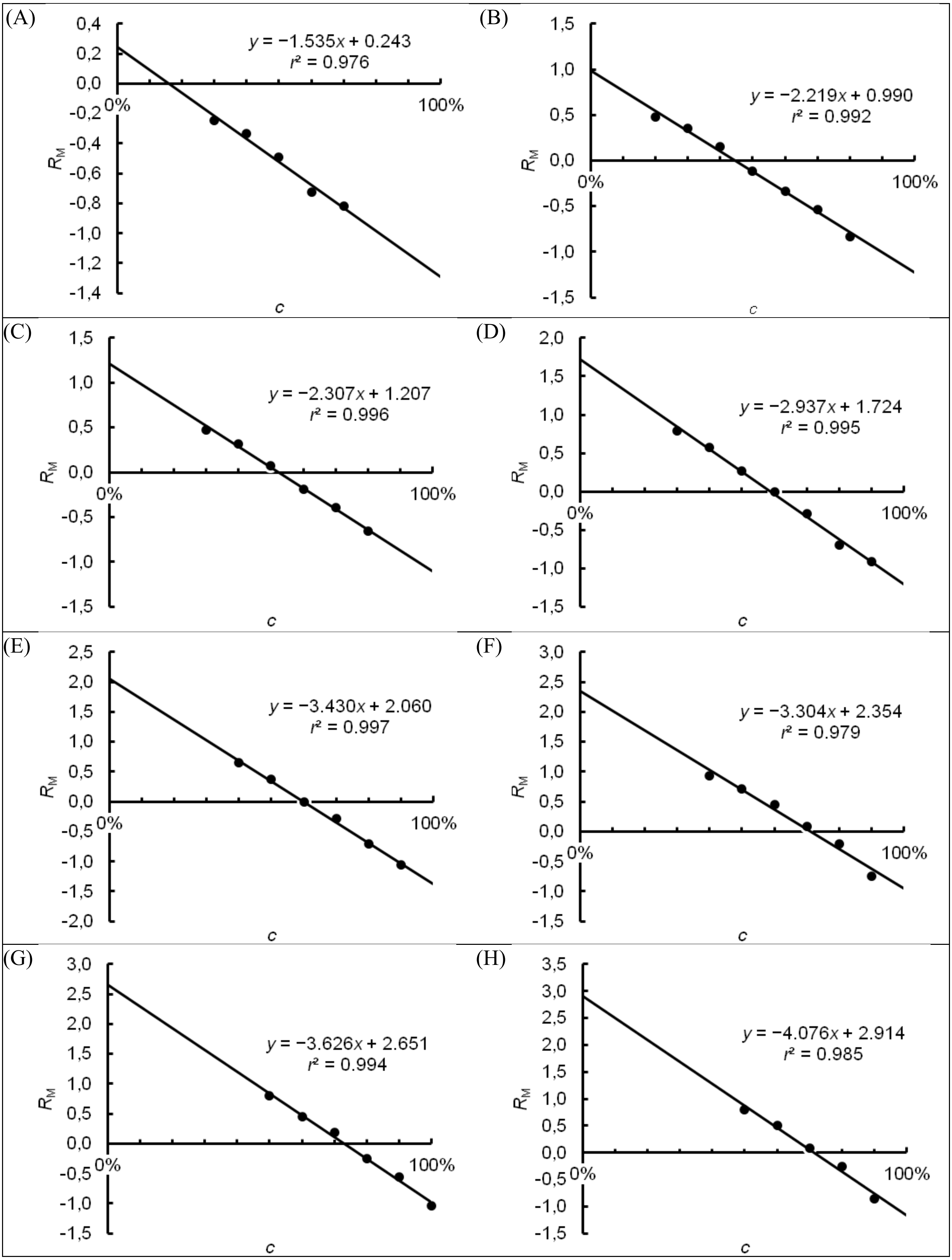

Figure S1. Linear dependence of R_M on the percentage of dioxane in the mobile phase (*c*) for the reference compounds used for log*P* calibration: (A) paracetamol, (B) acetanilide, (C) phenol, (D) dimethyl phthalate, (E) indole, (F) anthraquinone, (G) naphthalene, and (H) diphenylamine.

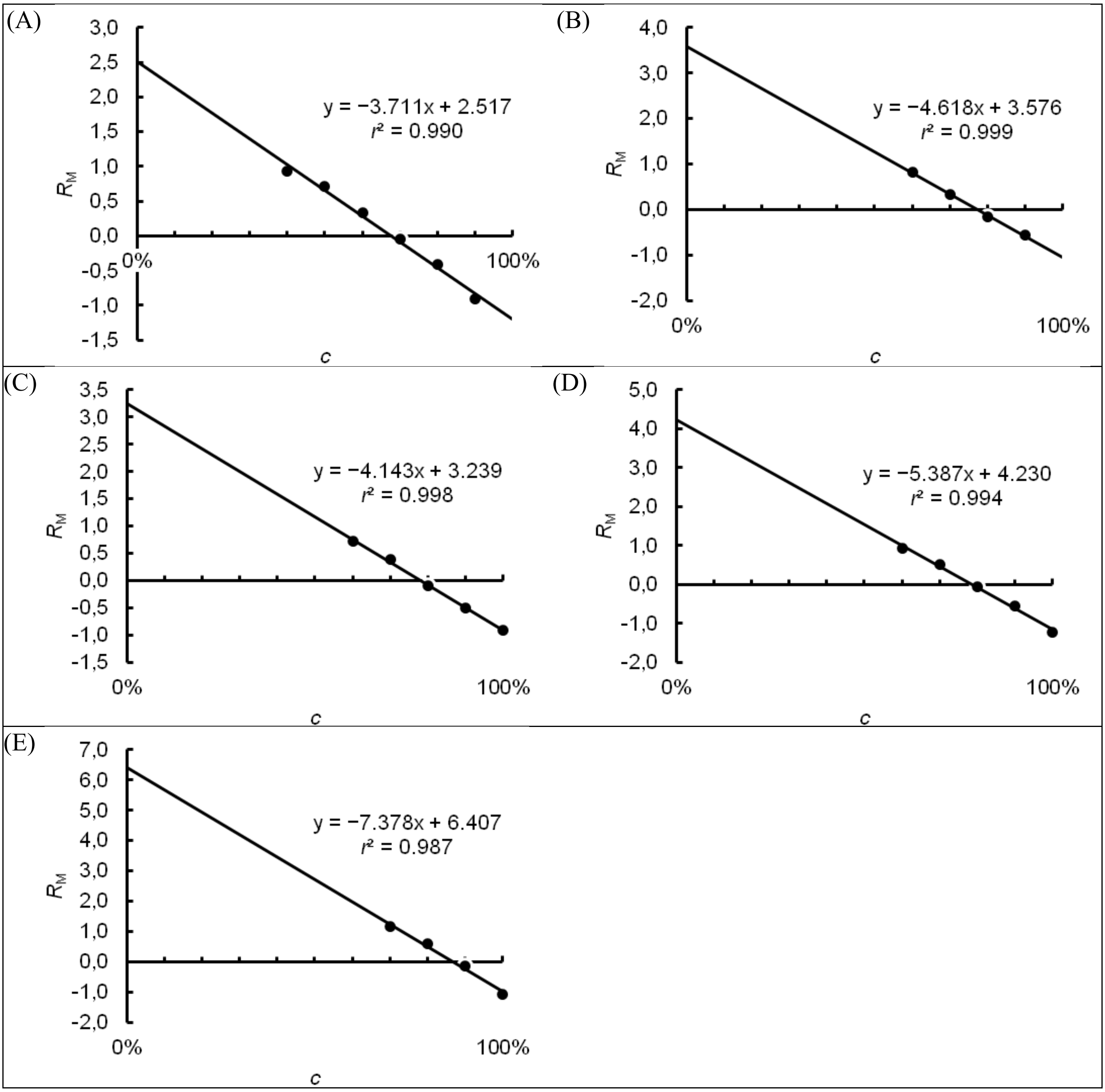

Figure S2. Linear dependence of R_M on the percentage of dioxane in the mobile phase (*c*) for the reference compounds used for log*P* calibration: (A) carbazole, (B) di-*n*-butyl phthalate, (C) pyrene, (D) triphenylamine, and (E) di-*n*-octyl phthalate.

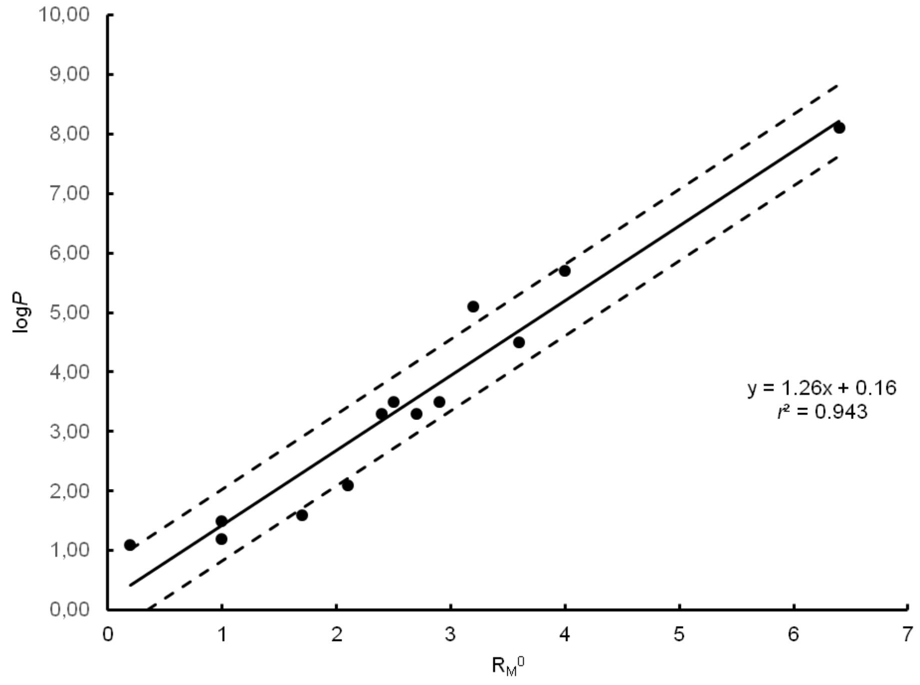

Figure S3. Calibration curve for determination of log*P* from RP-TLC data, showing the linear correlation between R_M^0 and literature log*P* values for the reference compounds.

Table S3. Parameters fitted for **1a**–**h** and their determined log*P* values.

Compound	*m*	R_M^0	r^2	log$P_{determined}$
**1a**	−2.1	0.7	0.993	**0.9 ± 0.6**
**1b**	−3.2	1.6	0.996	**2.0 ± 0.6**
**1c**	−5.4	4.0	0.995	**5.0 ± 0.6**
**1d**	−6.3	6.0	0.964	**7.6 ± 0.6**
**1e**	−5.1	3.7	0.997	**4.7 ± 0.6**
**1f**	−6.5	5.3	0.998	**6.7 ± 0.6**
**1g**	−5.5	3.9	0.986	**4.9 ± 0.6**
**1h**	−4.3	3.0	0.991	**3.8 ± 0.6**

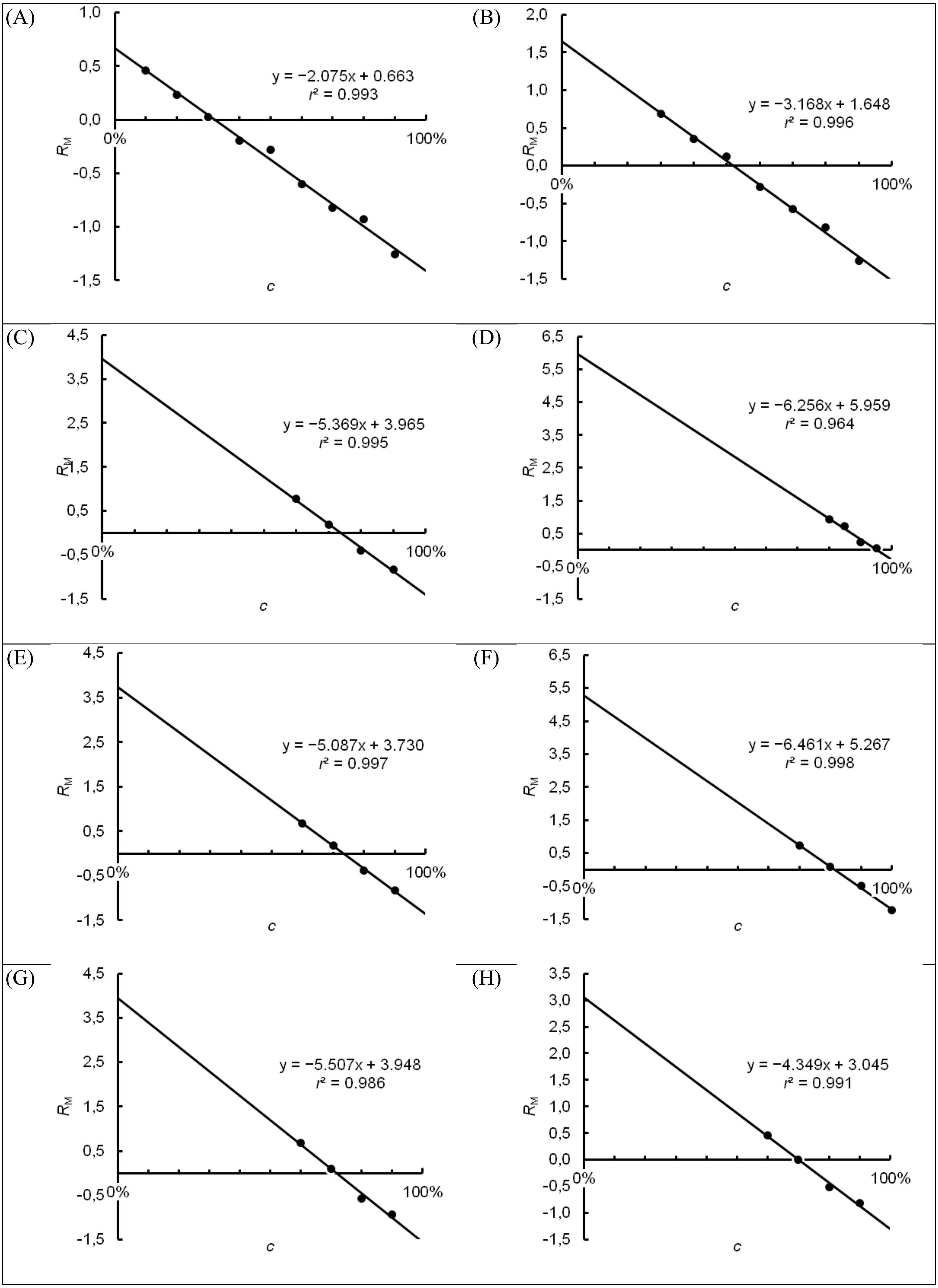

Figure S4. Linear functions fitted to R_M measured at different c for compounds **1a-h**: (A) **1a**, (B) **1b**, (C) **1c**, (D) **1d**, (E) **1e**, (F) **1f**, (G) **1g**, (H) **1h**.

1.6 Determination of the partition coefficient of 1b between CH_2Cl_2 and water

1.5 mL of 0.100 M solution of **1b** in CH_2Cl_2 was pre-washed with 1.5 mL of D_2O by stirring at 1700 rpm overnight. Then, phases were left to separate, and 1.00 mL of **1b** solution in CH_2Cl_2 was transferred to a fresh vial, and 1 mL of fresh D_2O was added. The mixture was stirred at 1700 rpm overnight and then left to separate, after which 500 µL of D_2O phase was transferred to an NMR tube and 5 µL of 12.0 mM solution of 3-(trimethylsilyl)propionic-2,2,3,3-d_4 acid sodium salt in D_2O was added as an internal reference. ^{1}H NMR spectrum (160 scans) was recorded (Figure S5). Integration (due to low signal-to-noise ratio caused by low concentration of **1b**, line fitting was used instead of classical integration algorithm) of signals of **1b** and reference gave a concentration of **1b** of 0.11 ± 0.1 mM, corresponding to $P_{CH2Cl2/water}$ = 910 ± 90.

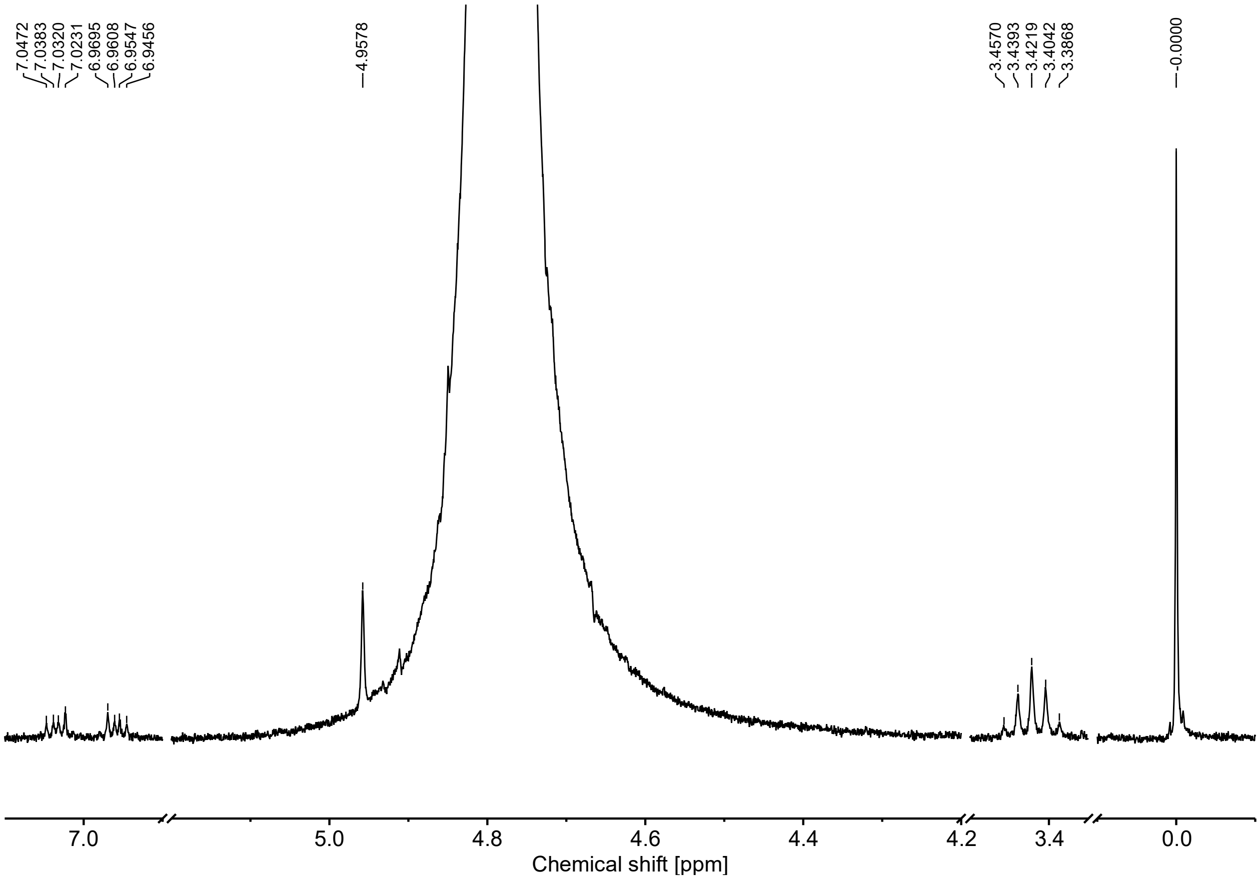

Figure S5. Partial ^{1}H NMR spectra of D_2O solution obtained after equilibration with 0.1 M solution of **1b** in CH_2Cl_2 and addition of a reference. Peaks of **1b** and peak of the reference compound are visible.

2. Binding studies

2.1 General remarks

The binding properties of **1a**–**h** were investigated by ^{1}H and, where applicable, ^{7}Li NMR, and UV-Vis titrations. Lithium, sodium, and potassium triflates (trifluoromethanesulfonates, TfO^-) were used as sources of alkali metal cations, due to their good solubility in organic solvents and the non-basic nature of the triflate anion. LiOTf and NaOTf were used as received. In the case of commercial KOTf, some insoluble impurities were observed, so KOTf was dissolved in hot anhydrous CH_3CN to give a white suspension, which was

filtered through a 0.45 μm PTFE syringe filter. The clear, colourless filtrate was evaporated on a rotary evaporator and dried under vacuum to afford purified KOTf, which was used directly for titrations. The external ^{7}Li reference was prepared by loading a PTFE tube (0.7 mm i.d., 1.1 mm o.d.), sealed at one end, with a LiCl solution in D_2O (~60 mM) under vacuum, then sealing the other end. First, exploratory ^{1}H and ^{7}Li NMR titrations of **1b** with $Li[B(C_6F_5)_4]·4Et_2O$ in CD_2Cl_2 were performed (Figs. S6–S9). These experiments revealed very strong binding and non-monotonic chemical-shift changes consistent with a predominant 2:1 (host:guest) binding mode in CH_2Cl_2. Because of the extremely high affinity, reliable values of $K_{1:1}$ and $K_{2:1}$ could not be obtained, so these data are discussed only qualitatively.

2.2 Titrations Procedures

For NMR titrations, stock solutions were prepared by dissolving an accurately weighed sample of a given receptor (±0.02 mg) in a precisely measured volume (±0.002 mL) of CD_3CN + 0.5% H_2O solvent mixture, giving ~10 mM solutions. These solutions were used directly for titrations with NaOTf and KOTf. For titrations with LiOTf, the receptor stock solution was diluted tenfold with the same CD_3CN/H_2O solvent mixture to obtain a final receptor concentration of ~1 mM (with the exception of the benzo-12-crown titration, which was performed at ~10 mM). An aliquot of the corresponding metal triflate was dissolved in the receptor solution, and 0.50 mL of this receptor solution was transferred to a screw-cap NMR tube (with the external ^{7}Li standard inserted in the case of LiOTf titrations). The metal triflate solution was then added stepwise using a microliter syringe, and ^{1}H NMR spectra (followed by ^{7}Li NMR spectra, where applicable) were recorded after each addition at 400 MHz for ^{1}H and 155.5 MHz for ^{7}Li.

For UV-Vis titrations, stock solutions were prepared by dissolving an accurately weighed sample of a given receptor (±0.02 mg) in a precisely measured volume (±0.002 mL) of CH_3CN + 0.5% H_2O solvent mixture, and then diluted with the same CH_3CN/H_2O solvent mixture to ~0.2 mM. An aliquot of the corresponding metal triflate was dissolved in the receptor solution, and 2.2 mL of this receptor solution was transferred to a screw-cap UV-Vis quartz cuvette (1 cm pathlength). The metal triflate solution was then added stepwise using a microliter syringe, and UV-Vis spectra (250 – 400 nm range) were recorded after each addition.

2.3 Data analysis and results

For ^{1}H NMR, the residual solvent signal was used as an internal reference (δ = 1.9400 ppm for CD_3CN, δ = 5.3200 ppm for CD_2Cl_2). For ^{7}Li NMR, the external $LiCl/D_2O$ capillary described above was used as the reference (δ = 0.0000 ppm). Selected chemical shifts were assigned to four decimal places, and simultaneous nonlinear curve fitting of all selected protons was performed with HypNMR2008 (v. 4.0.68).[33,34] For titrations with LiOTf, a 1:1 binding model provided good fits and was used throughout. Introducing a 2:1 (host:guest) model did not result in any significant improvement in fit quality or residuals distribution, so the formation of 2:1 complexes with LiOTf under the titration conditions was considered unlikely. ^{7}Li titrations, except for **1b** (which was fitted directly together with ^{1}H data), were treated as control experiments. ^{7}Li chemical shifts of both free Li^+ and Li^+⊂**1** were obtained by linear regression, giving binding isotherms that generally

corroborated those derived from ^{1}H NMR. The poorer quality of ^{7}Li fits compared to ^{1}H fits is attributed to the lower spectral resolution of ^{7}Li NMR and, more importantly, to the concentration dependence of ^{7}Li shifts arising from strong solvation of Li^{+}. For titrations with NaOTf and KOTf, clear non-monotonic behaviour of some signals was observed, so a 2:1 (host:guest) binding model was applied. For titrations of **1a**–**1c**, **1e**, and **1h** with NaOTf, fitting with both $K_{1:1}$ and $K_{2:1}$ fully relaxed did not converge. In these cases, $K_{1:1}$ was kept as a free parameter, whereas $K_{2:1}$ was varied manually to identify the $K_{2:1}$ range that minimized the standard deviation, providing an estimate of $K_{2:1}$. Within this range, $K_{1:1}$ showed only a weak dependence on $K_{2:1}$, which allowed reasonably reliable determination of $K_{1:1}$ even when $K_{2:1}$ remained uncertain. Additional ^{1}H NMR titrations with NaOTf were carried out at ~1 mM host concentration to test the robustness of the fitting at a lower concentration. Although some datasets readily converged, confirming values obtained for higher concentration, obtaining a fully relaxed fit for all titrations was still impossible. Therefore, the low-concentration regime was instead further evaluated by UV–Vis titrations. The NMR-derived stability constants K and selectivity factors for complexes of **1a**–**h** and benzo-12-crown-4 (**B12C4**) with Li^{+}, Na^{+}, and K^{+} are summarized in Table S4. **B12C4** is included for comparison as a classical macrocyclic host, which is generally considered to be well matched to the lithium cation. However, in the studied solvent system, this host proved to be rather sodium-selective, with $K_{1:1}(Li)/K_{1:1}(Na) < 1$. A similar behaviour was recently reported by Liu et al.,[18] who found $K_{1:1}(Li)/ K_{1:1}(Na) = 0.5$ for 12-crown-4, which is closely related to **B12C4**. A complete set of stacked ^{1}H and ^{7}Li NMR spectra, chemical-shift changes, and fitted binding isotherms is shown in Figures S6–S81 (Section 2.4).

UV-Vis titrations data were fitted in the Musketeer software tool[35] in the 295-250 nm range using a 1:1 binding model, providing very good fits. Introducing a 2:1 (host:guest) model in all cases did not result in any significant improvement in fit quality, and, consistently with this, fitted spectra of H_2G species were random and noisy, suggesting effective shutdown of 2:1 binding mode under 0.2 mM host concentration. A complete set of stacked UV-Vis spectra, absorbance changes, and fitted binding isotherms is shown in Figures S82–S97 (Section 2.4), and obtained binding constants K are summarised in Table S5.

Table S4. Stability constants K [M^{-1}] derived from NMR titrations for complexes of **1a**–**h** and benzo-12-crown-4 (**B12C4**) with Li^+, Na^+, and K^+ (as triflate salts) in CD_3CN + 0.5% H_2O, and the corresponding selectivity factors.[a]

Host	LiOTf [b]	NaOTf [c], [d]	KOTf [c]	$K_{1:1}$(Li)/$K_{1:1}$(Na)	$K_{1:1}$(Li)/$K_{1:1}$(K)
**1a**	17100	3400<br>(<50)	200<br>(20)	5	86
**1b**	44700	4700<br>(<60)	200<br>(<5)	9.5	220
**1c**	45200	3800<br>(<60)	230[d]<br>(24)[d]	12	200
**1d**	95000[d]	5200<br>(290)	230[d]<br>(130)[d]	18	410
**1e**	19700	3300<br>(<30)	230[d]<br>(9)[d]	6	90
**1f**	21500	4800<br>(110)	250[d]<br>(50)[d]	4.5	86
**1g**	2050	1000<br>(170)	100[d]<br>(7)[d]	2	21
**1h**	63000[d]	4300<br>(<100)	270[d]<br>(27)[d]	15	230
**B12C4**	28[d]	94<br>(165)	2[d]<br>(33)[d]	0.3	1.2

[a] Estimated uncertainties are ±10% unless stated. [b] Data fitted with a 1:1 model. [c] Data fitted with a 2:1 (host:guest) model; $K_{2:1}$ is given in parentheses below $K_{1:1}$. [d] Estimated uncertainty ±20%.

Table S5. Stability constants K [M^{-1}] UV-Vis titrations for complexes of **1a**–**h** with Li^+ and Na^+ (as triflate salts) in CH_3CN + 0.5% H_2O, and the corresponding selectivity factors[a]

Host	LiOTf [b]	NaOTf [c]	$K_{1:1}$(Li)/$K_{1:1}$(Na)
**1a**	65000	5800	11
**1b**	110000	6400	17
**1c**	61000	5500	11
**1d**	59000	3900	15
**1e**	58000	4700	12
**1f**	78000	9600	8
**1g**	2600	860	3
**1h**	67000	5800	12

[a] Estimated uncertainties are ±30%.

2.4 Titration data for 1a–h and B12C4

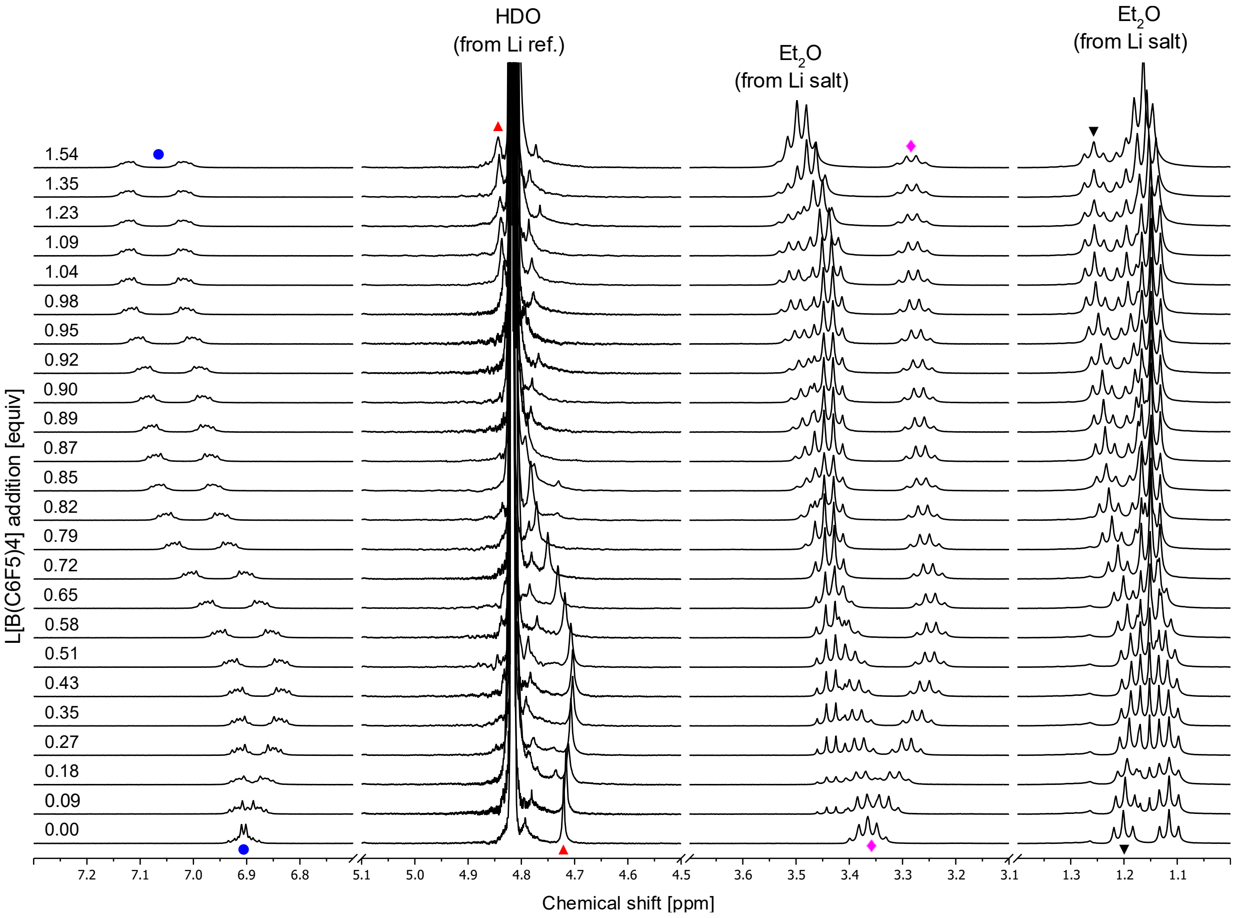

Figure S6. Stacked ^{1}H NMR spectra for titration of **1b** with $Li[B(C_6F_5)_4]\cdot 4Et_2O$ in CD_2Cl_2 at 298 K.

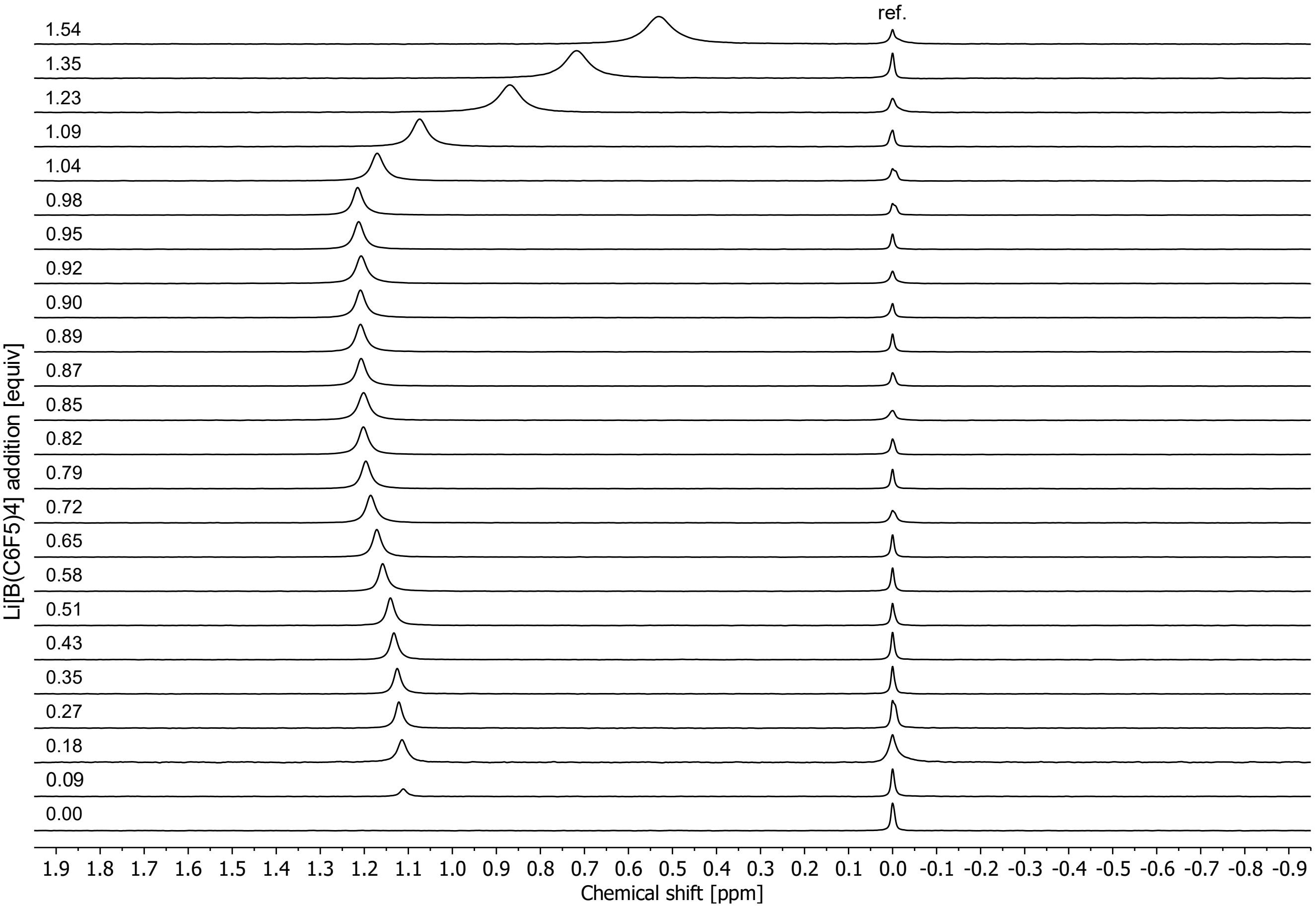

Figure S7. Stacked ^{7}Li NMR spectra for titration of **1b** with $Li[B(C_6F_5)_4]\cdot 4Et_2O$ in CD_2Cl_2 at 298 K.

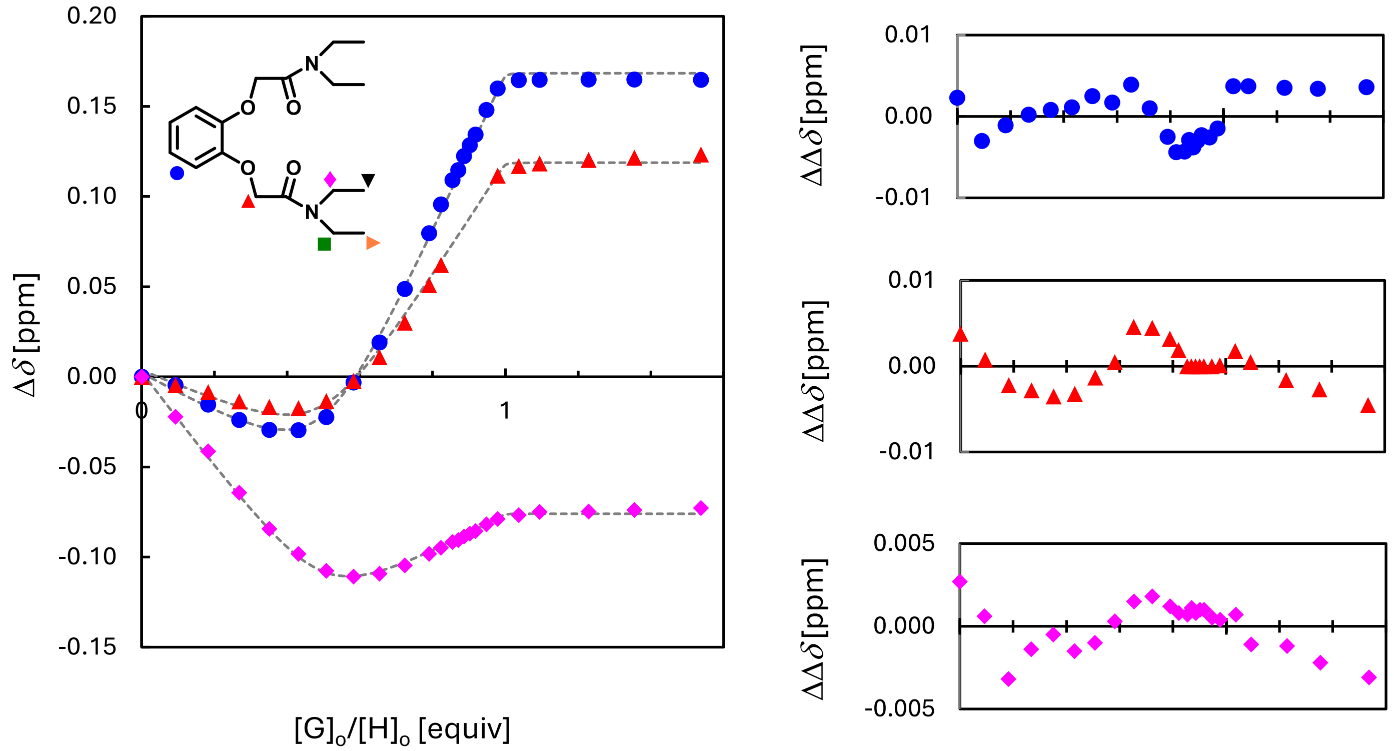

Figure S8. Experimental ^{1}H NMR chemical shift changes (symbols) and fitted binding isotherms (gray dashed lines) for titration of **1b** with $\mathrm{Li[B(C_6F_5)_4]\cdot 4Et_2O}$ in CD_2Cl_2 at 298K (left), assuming 2:1 binding model; residual distribution for the corresponding shift (right). Due to very high binding constants, it was impossible to reliably determine $K_{1:1}$ or $K_{2:1}$. Good fit between 2:1 model and experimental data is proof of a 2:1 binding mode in CD_2Cl_2, in contrast to CD_3CN/H_2O.

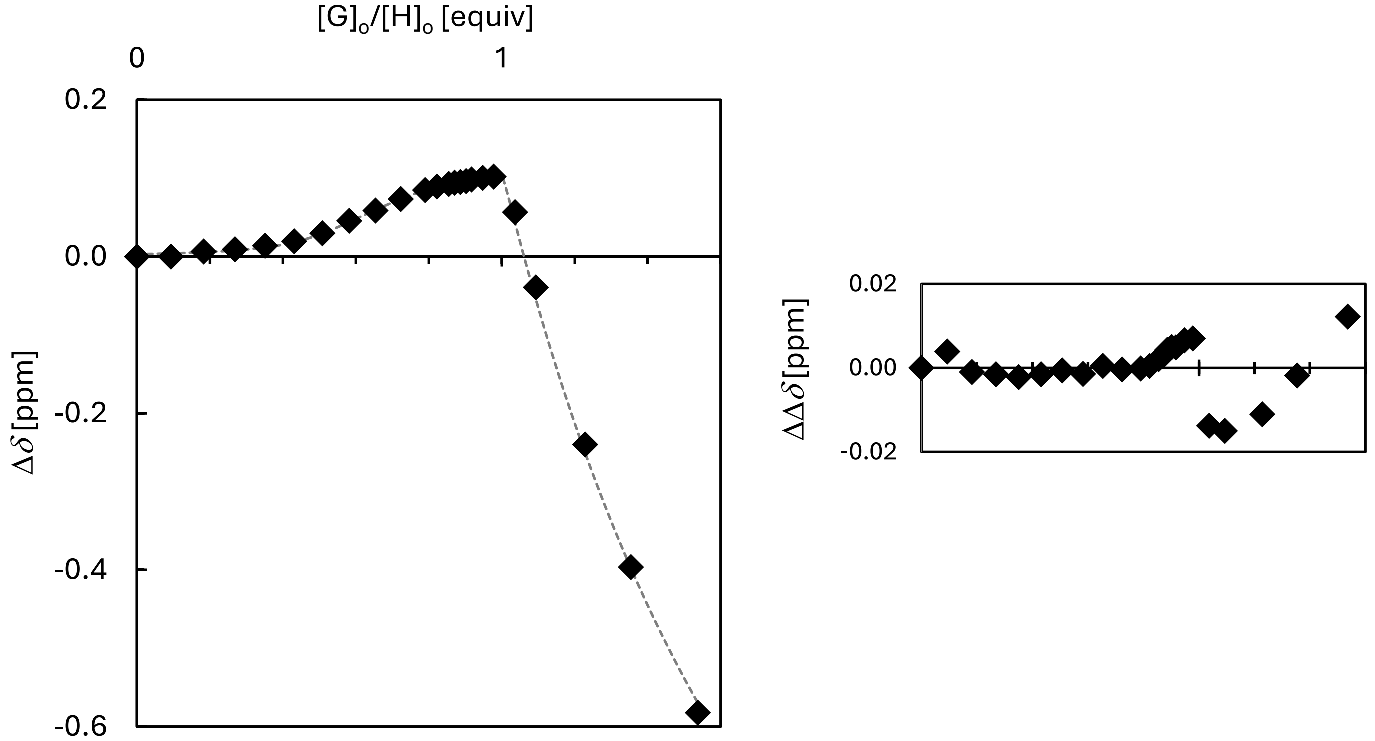

Figure S9. ^{7}Li NMR chemical-shift changes (symbols) and 2:1 binding-model fits (lines) for titration of **1b** with $\mathrm{Li[B(C_6F_5)_4]\cdot 4Et_2O}$ in CD_2Cl_2 at 298 K. Very strong binding prevented reliable determination of $K_{2:1}$ and $K_{1:1}$, but the good fit supports a 2:1 binding mode in CD_2Cl_2 vs 1:1 in CD_3CN/H_2O.

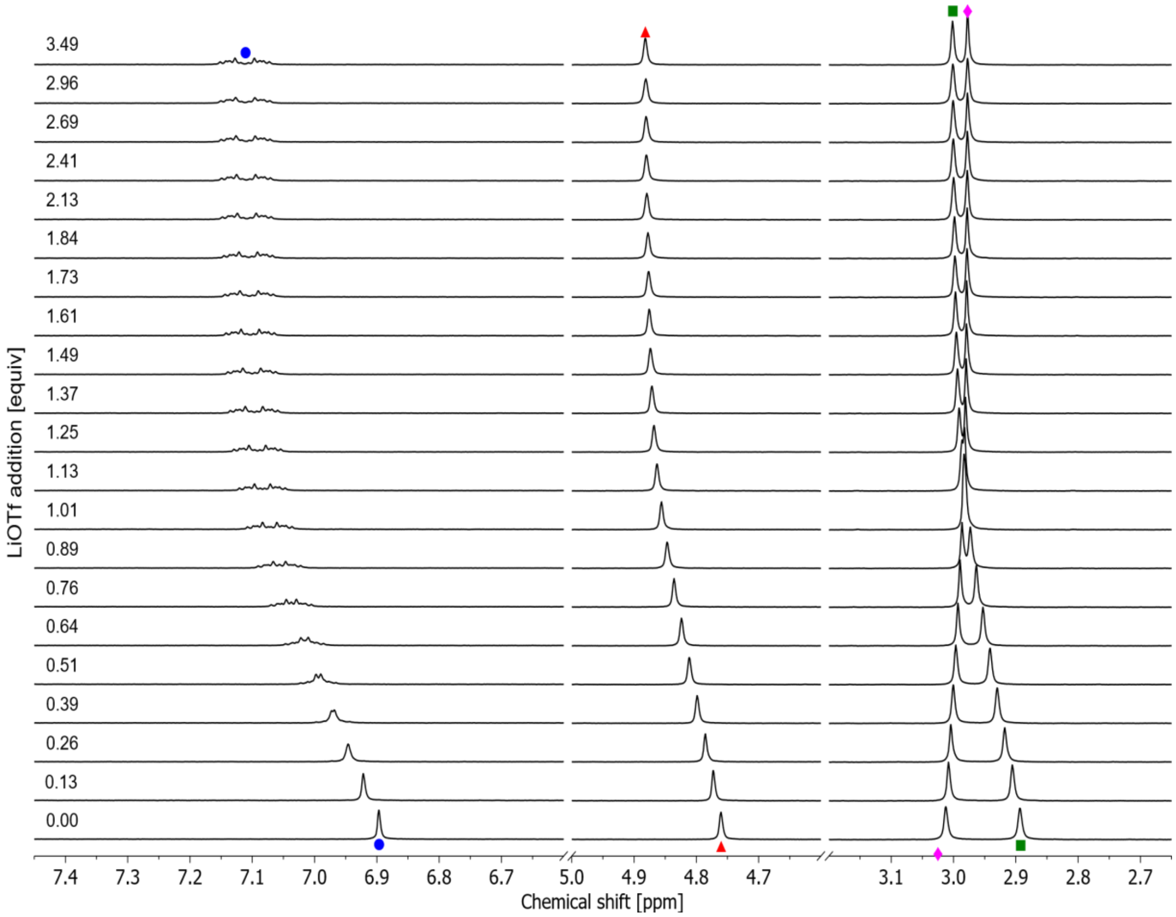

Figure S10. Stacked spectra from the ^{1}H NMR titration of **1a** with increasing amount of LiOTf at 298 K.

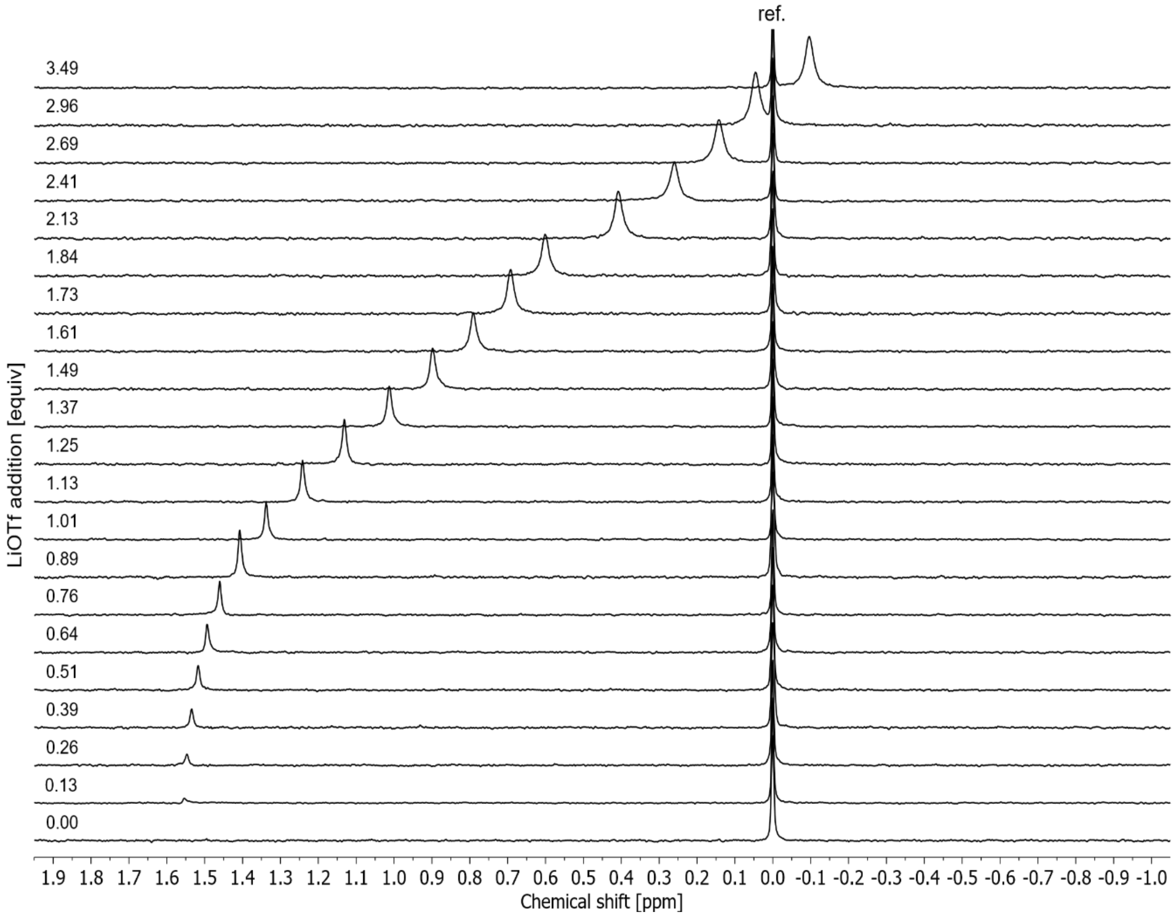

Figure S11. Stacked spectra from the ^{7}Li NMR titration of **1a** with increasing amount of LiOTf at 298 K.

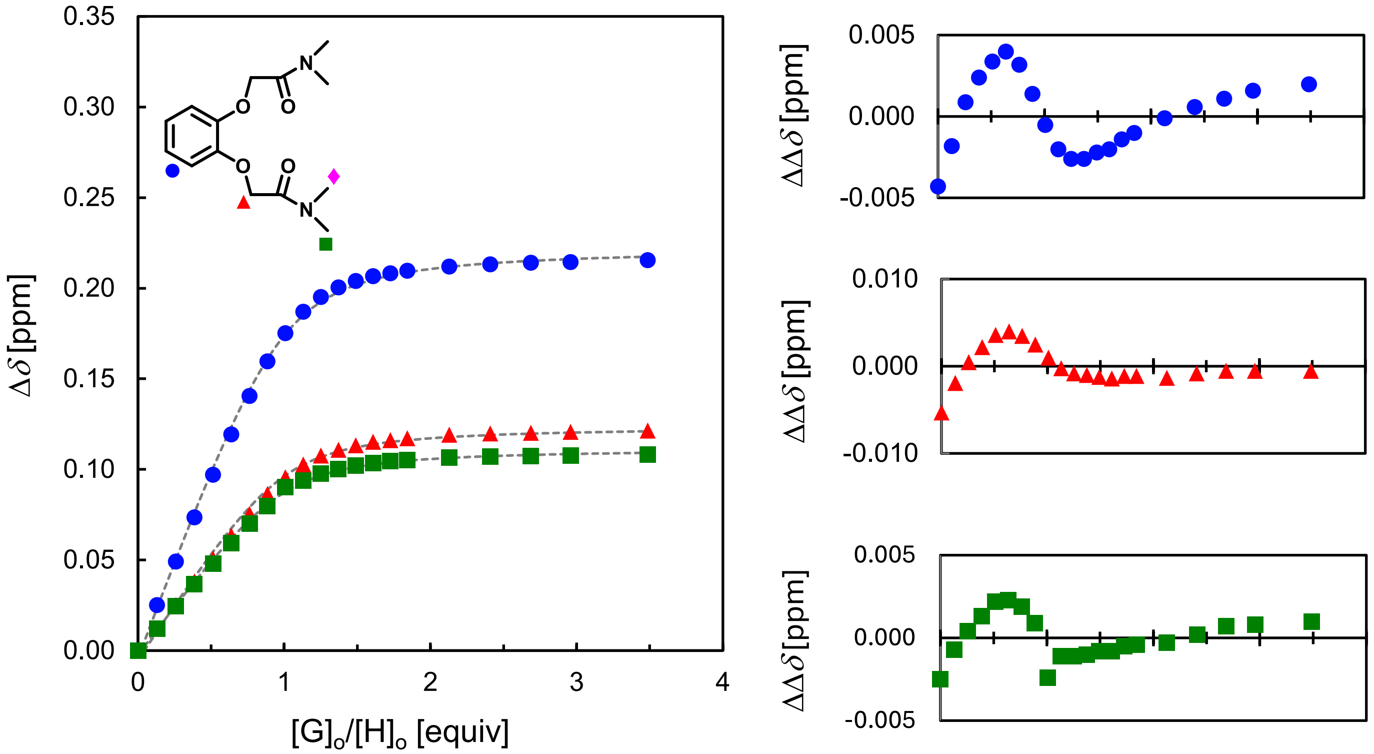

Figure S12. Selected experimental ^{1}H NMR chemical-shift changes (symbols) and fitted binding isotherms (gray dashed lines) for titration of **1a** with LiOTf in 99.5% CD_3CN + 0.5% H_2O at 298 K (left), assuming 1:1 binding model; residual distribution for the corresponding shift (right).

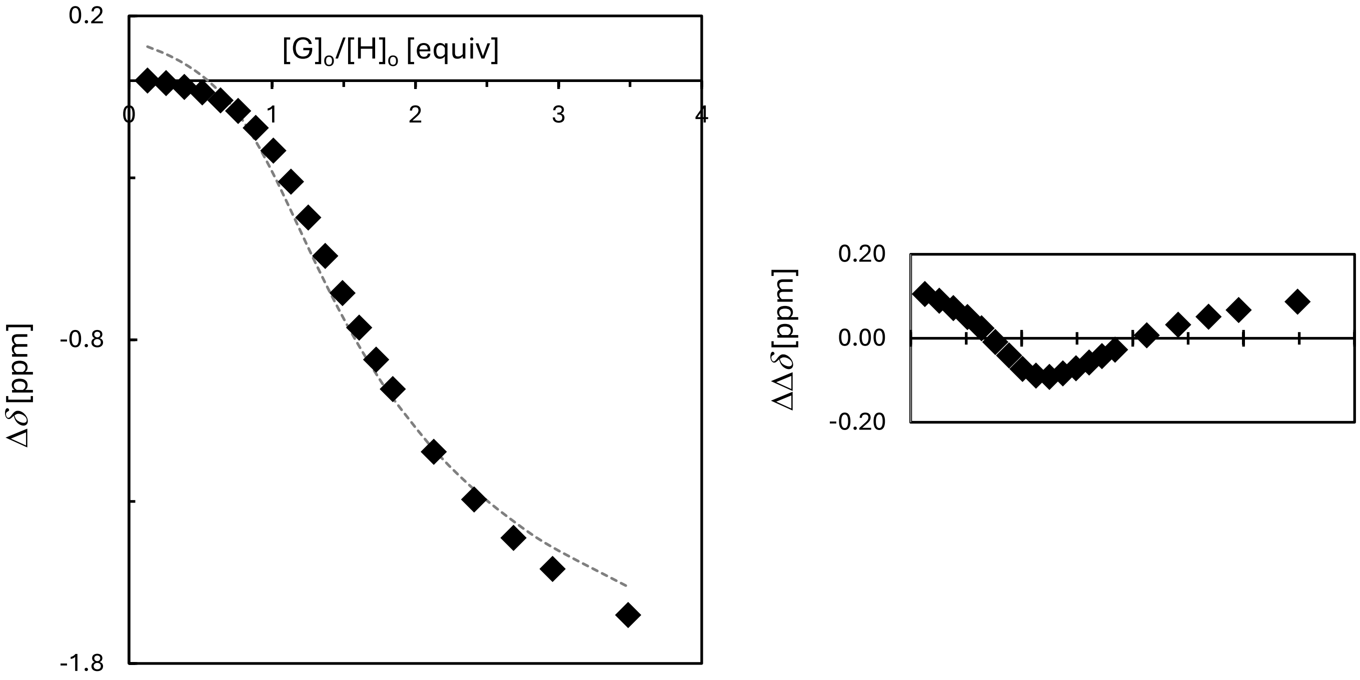

Figure S13. Experimental ^{7}Li NMR chemical-shift changes (symbols) and calculated binding isotherms (gray dashed lines) for titration of **1a** with LiOTf in 99.5% CD_3CN + 0.5% H_2O at 298 K (left), assuming 1:1 binding model; residual distribution for the corresponding shift (right).

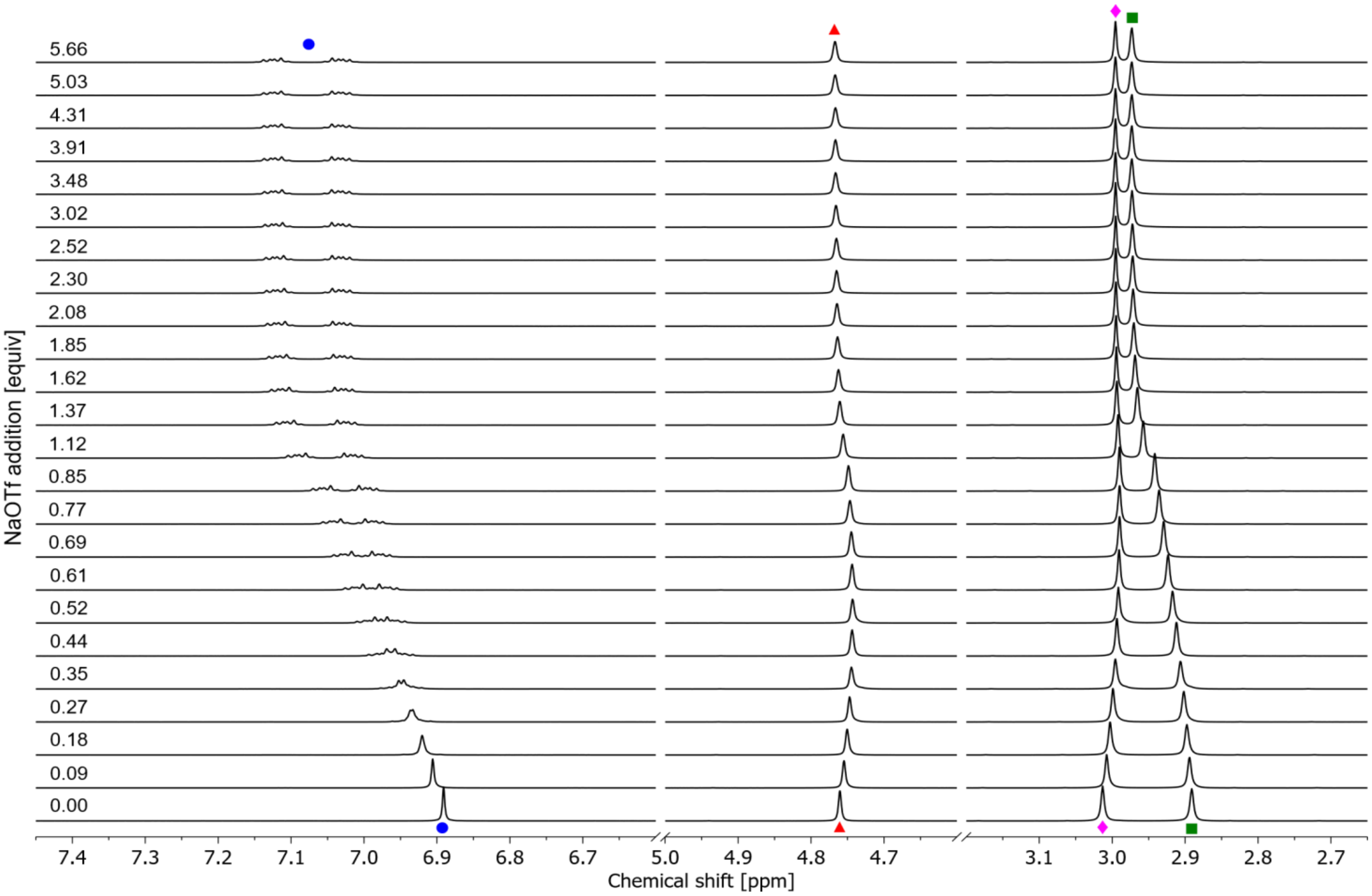

Figure S14. Stacked spectra from the ^{1}H NMR titration of **1a** with increasing amount of NaOTf at 298 K.

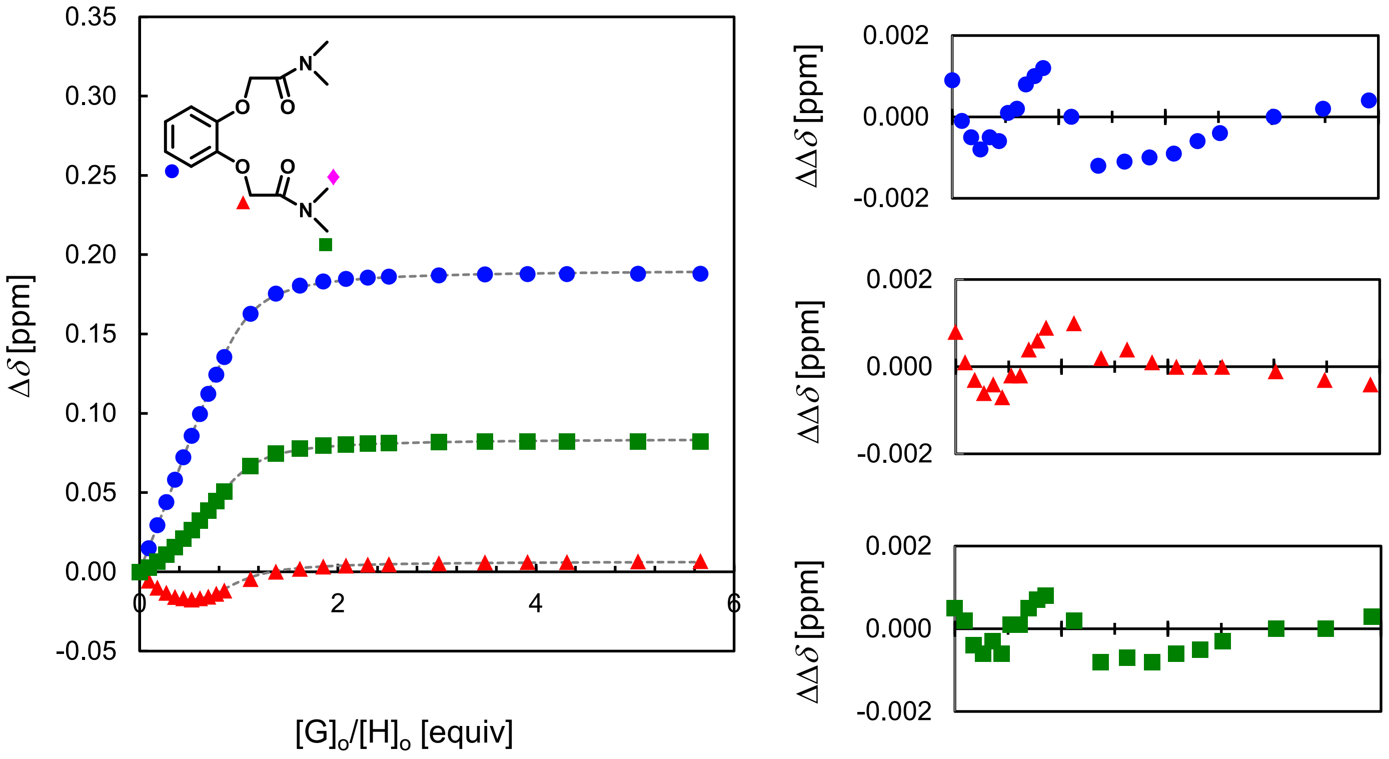

Figure S15. Experimental ^{1}H NMR chemical shift changes (symbols) and fitted binding isotherms (gray dashed lines) for titration of **1a** with NaOTf in 99.5% CD_3CN + 0.5% H_2O at 298 K (left), assuming 2:1 (host:guest) binding model; residual distribution for the corresponding shift (right).

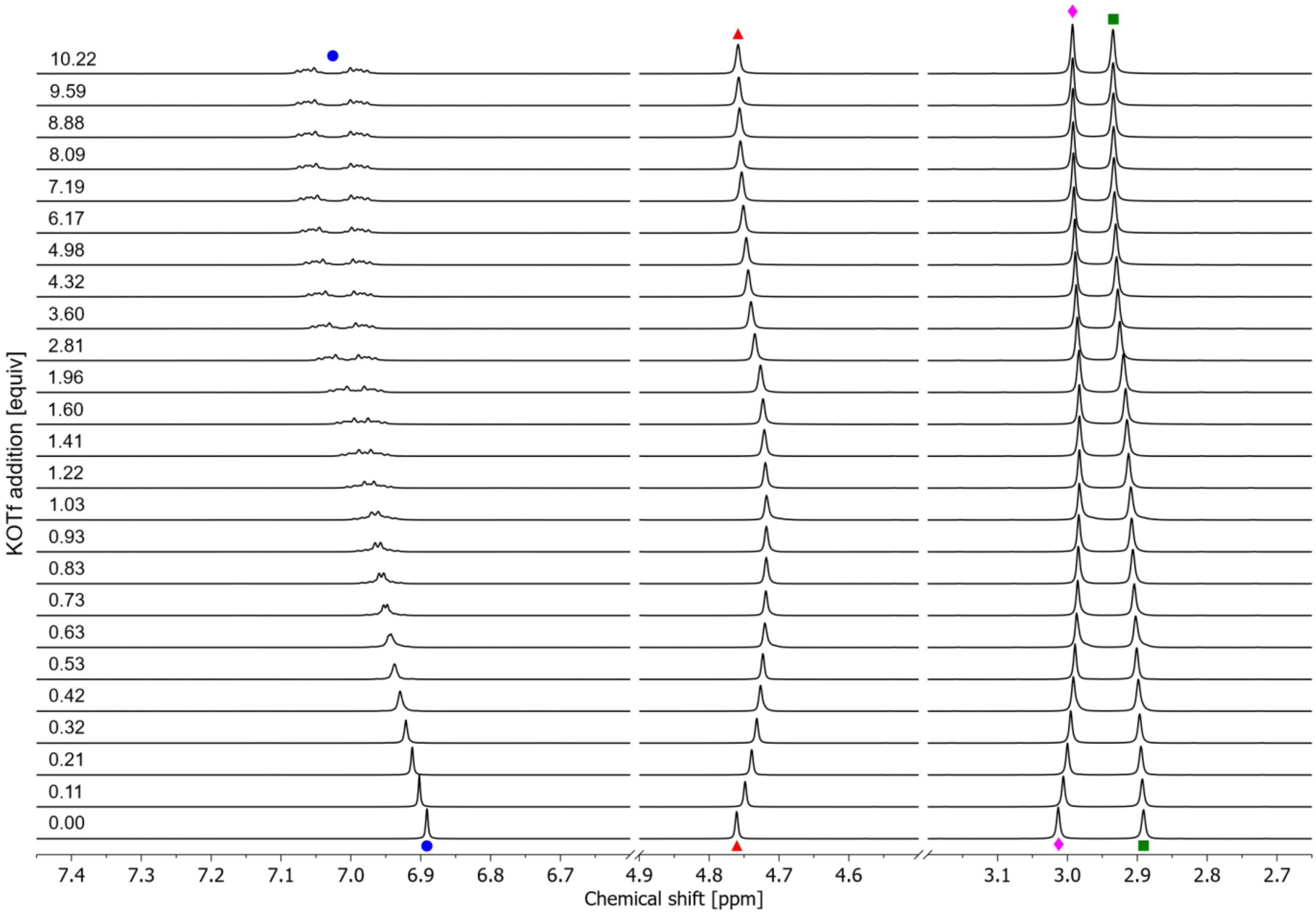

Figure S16. Stacked spectra from the ^{1}H NMR titration of **1a** with increasing amount of KOTf at 298 K.

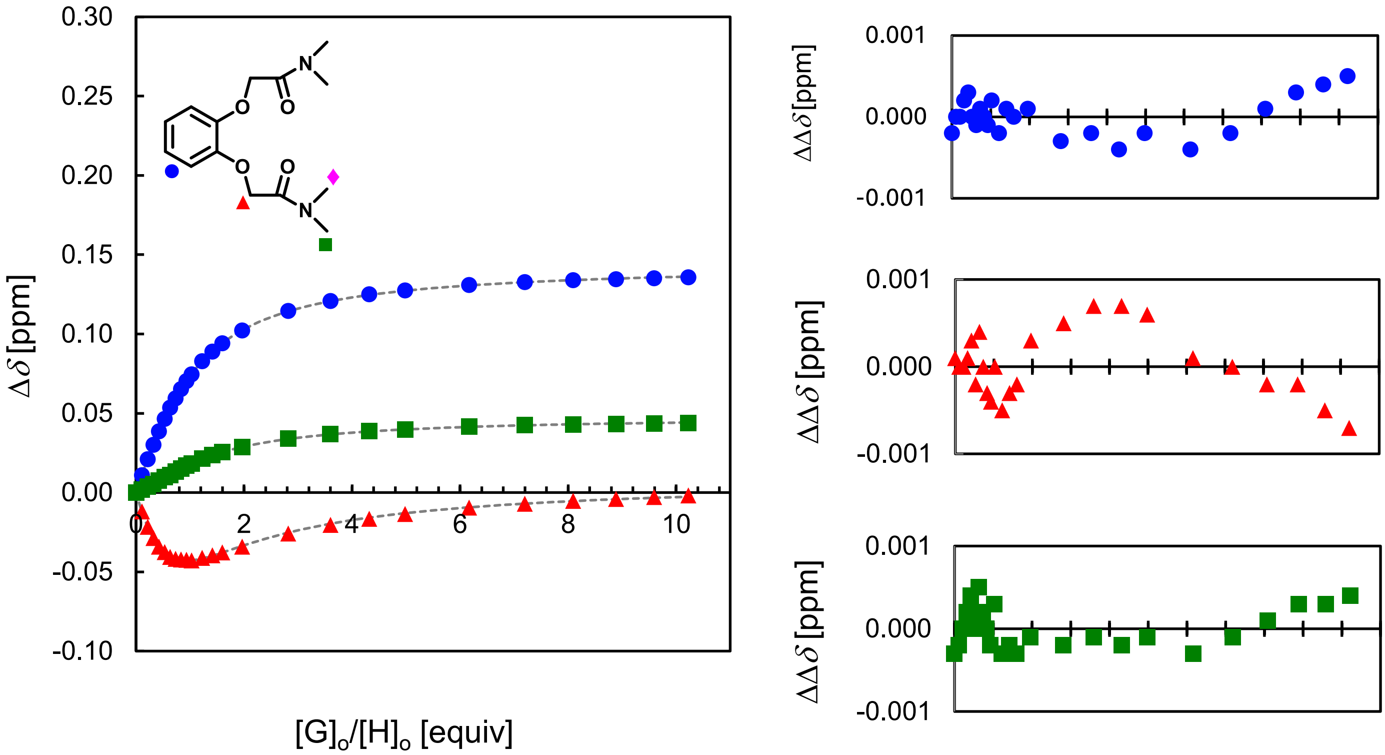

Figure S17. Experimental ^{1}H NMR chemical shift changes (symbols) and fitted binding isotherms (gray dashed lines) for titration of **1a** with KOTf in 99.5% CD_3CN + 0.5% H_2O at 298 K (left), assuming 2:1 (host:guest) binding model; residual distribution for the corresponding shift (right).

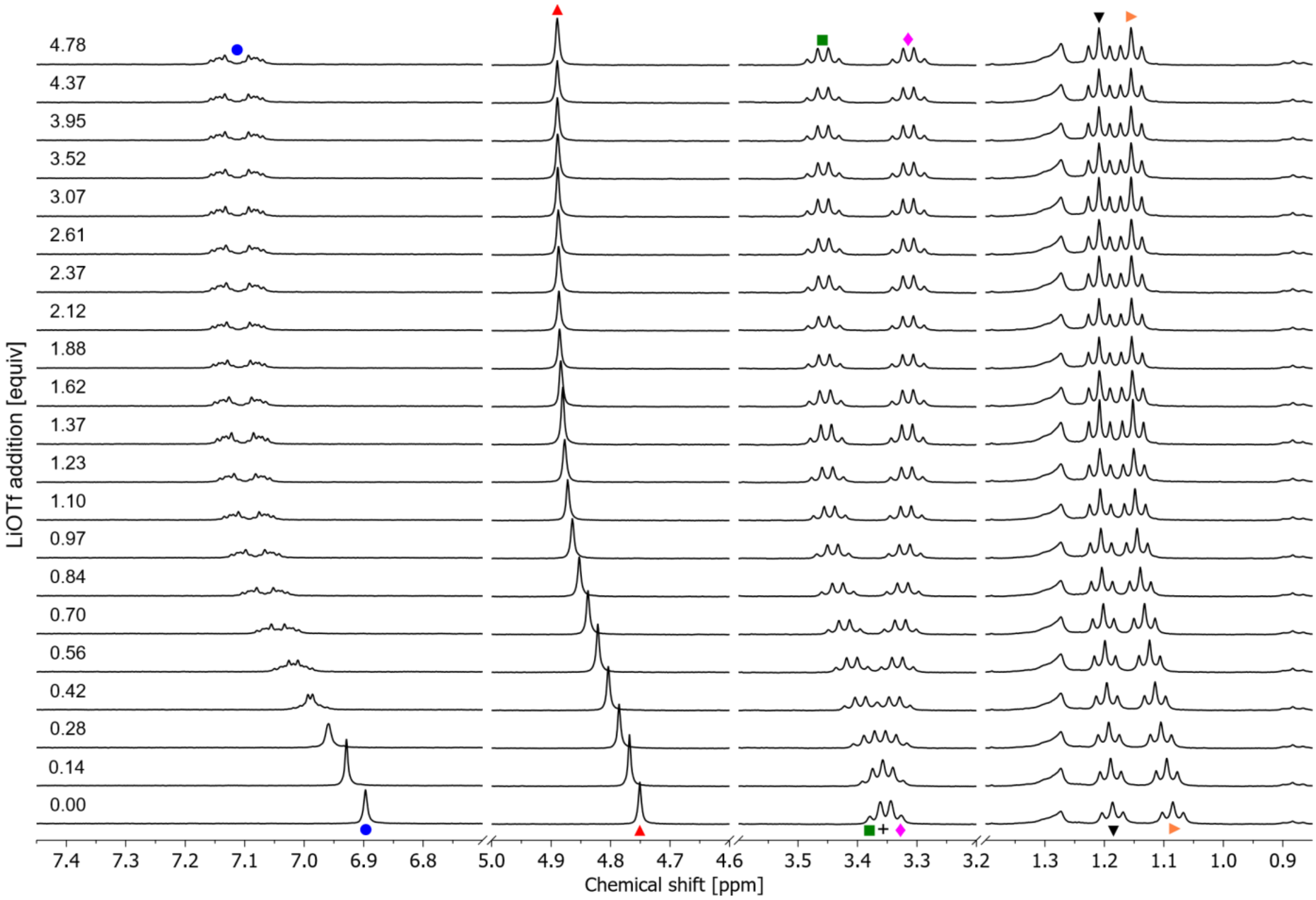

Figure S18. Stacked spectra from the ^{1}H NMR titration of **1b** with increasing amount of LiOTf at 298 K.

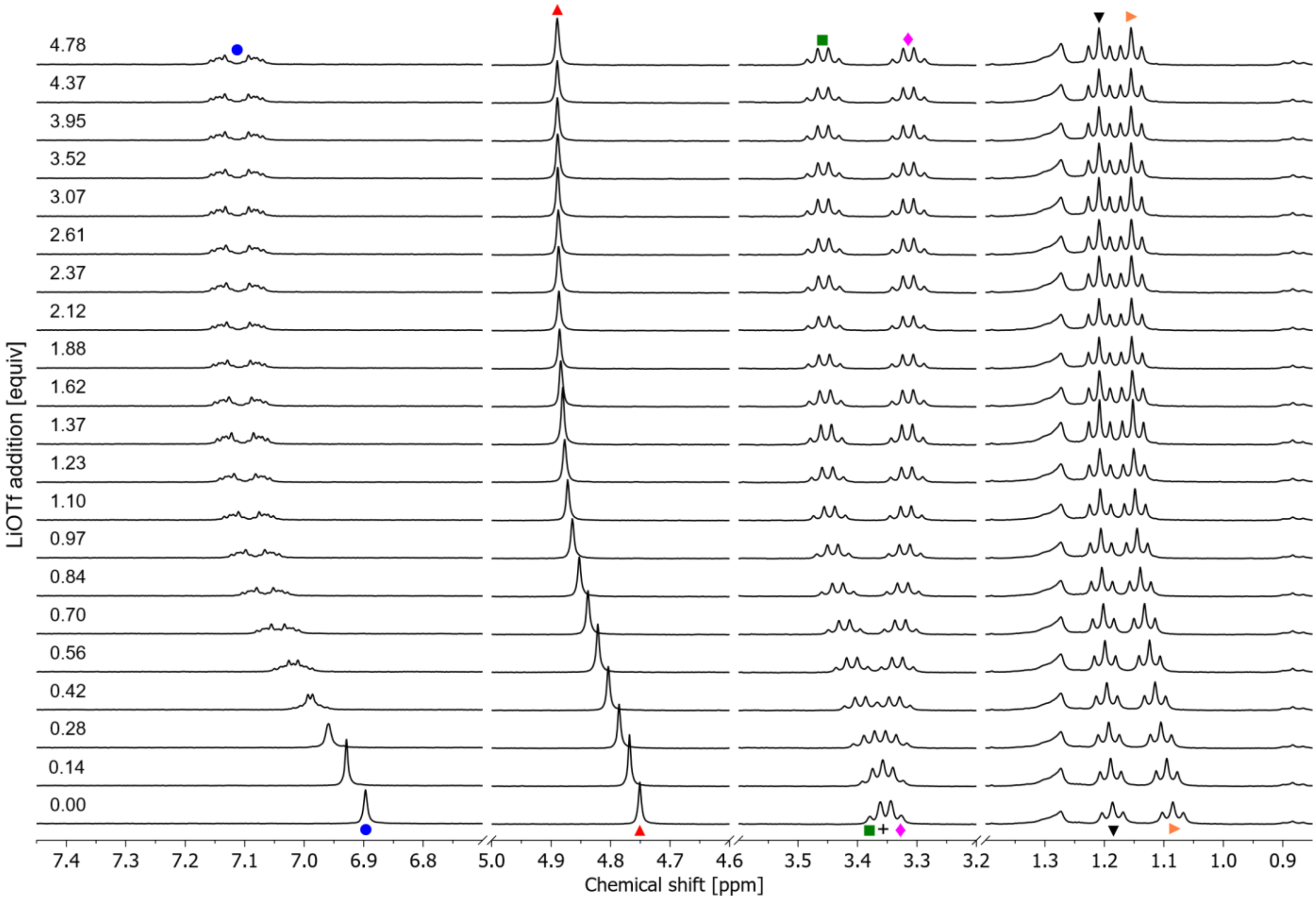

Figure S19. Stacked spectra from the ^{7}Li NMR titration of **1b** with increasing amount of LiOTf at 298 K.

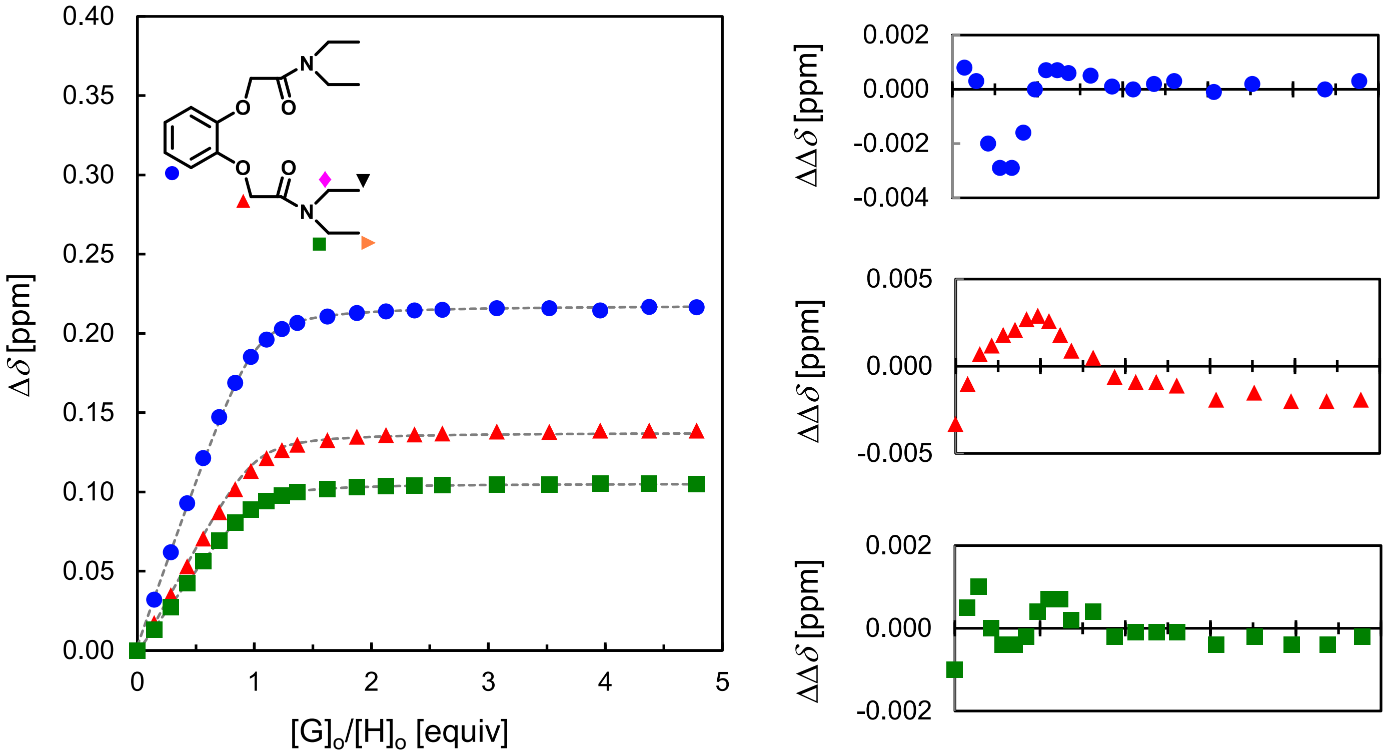

Figure S20. Experimental ^{1}H NMR chemical shift changes (symbols) and fitted binding isotherms (gray dashed lines) for titration of **1b** with LiOTf in 99.5% CD_3CN + 0.5% H_2O at 298 K (left), assuming 1:1 binding model; residual distribution for the corresponding shift (right).

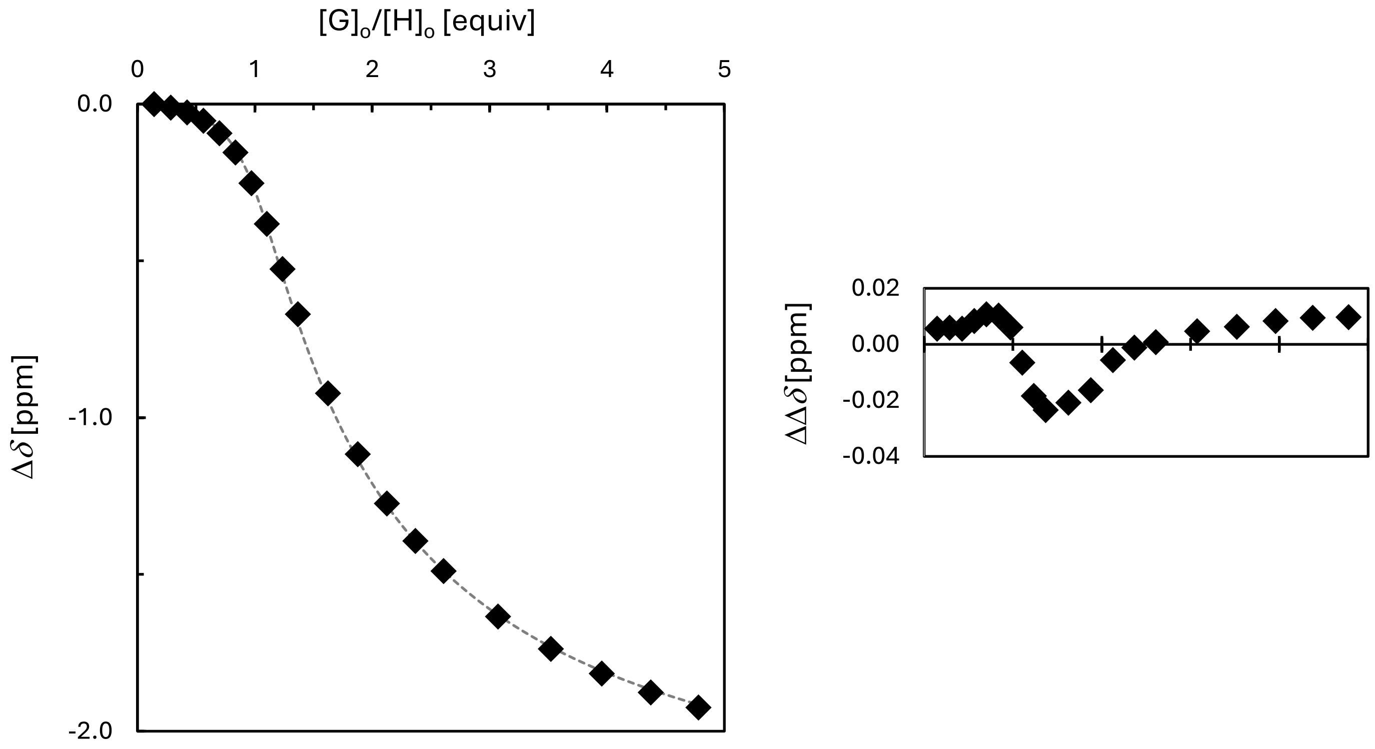

Figure S21. Experimental ^{7}Li NMR chemical shift changes (symbols) and fitted binding isotherms (gray dashed lines) for titration of **1b** with LiOTf in 99.5% CD_3CN + 0.5% H_2O at 298 K (left), assuming 1:1 binding model; residual distribution for the corresponding shift (right).

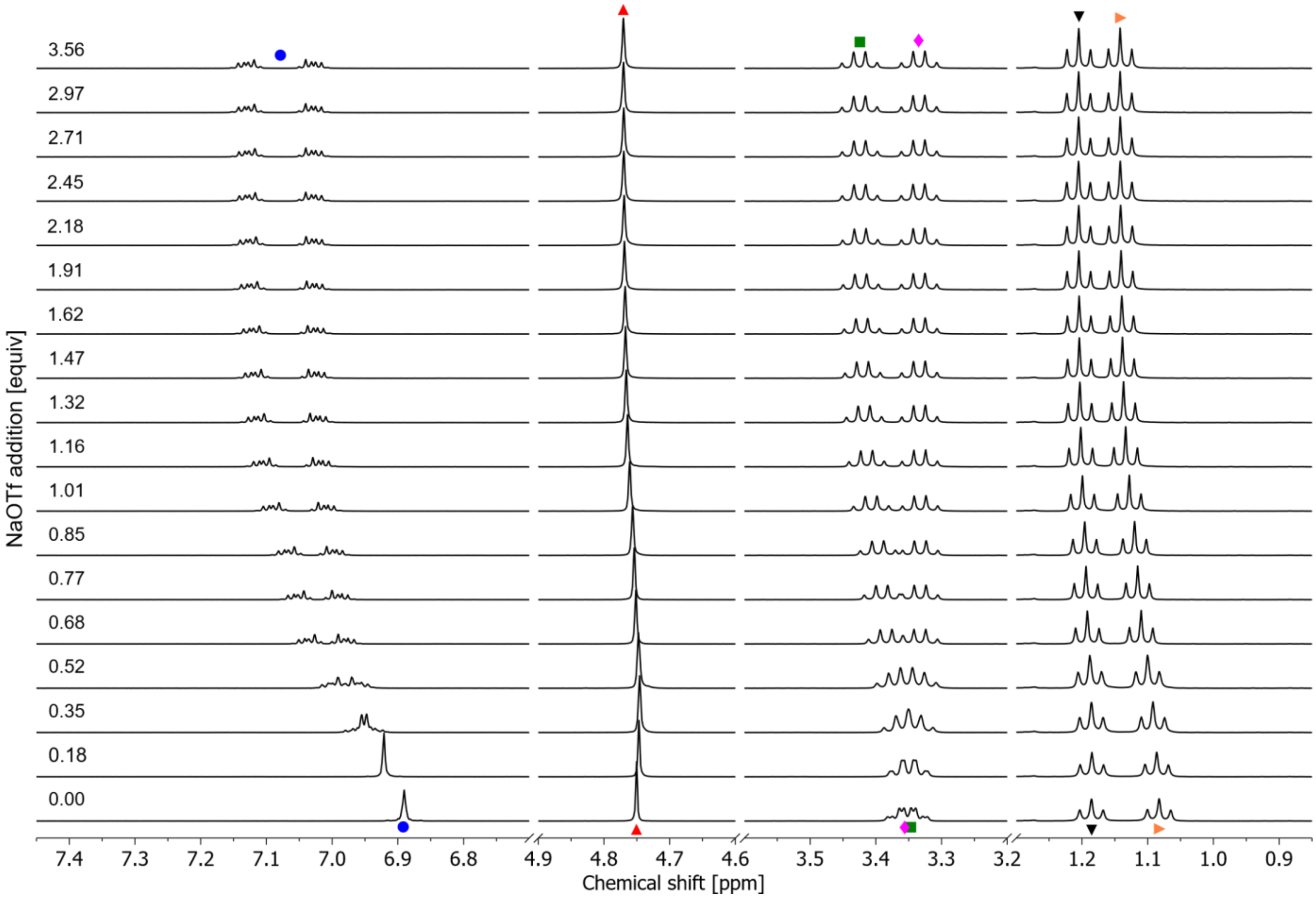

Figure S22. Stacked spectra from the ^{1}H NMR titration of **1b** with increasing amount of NaOTf.

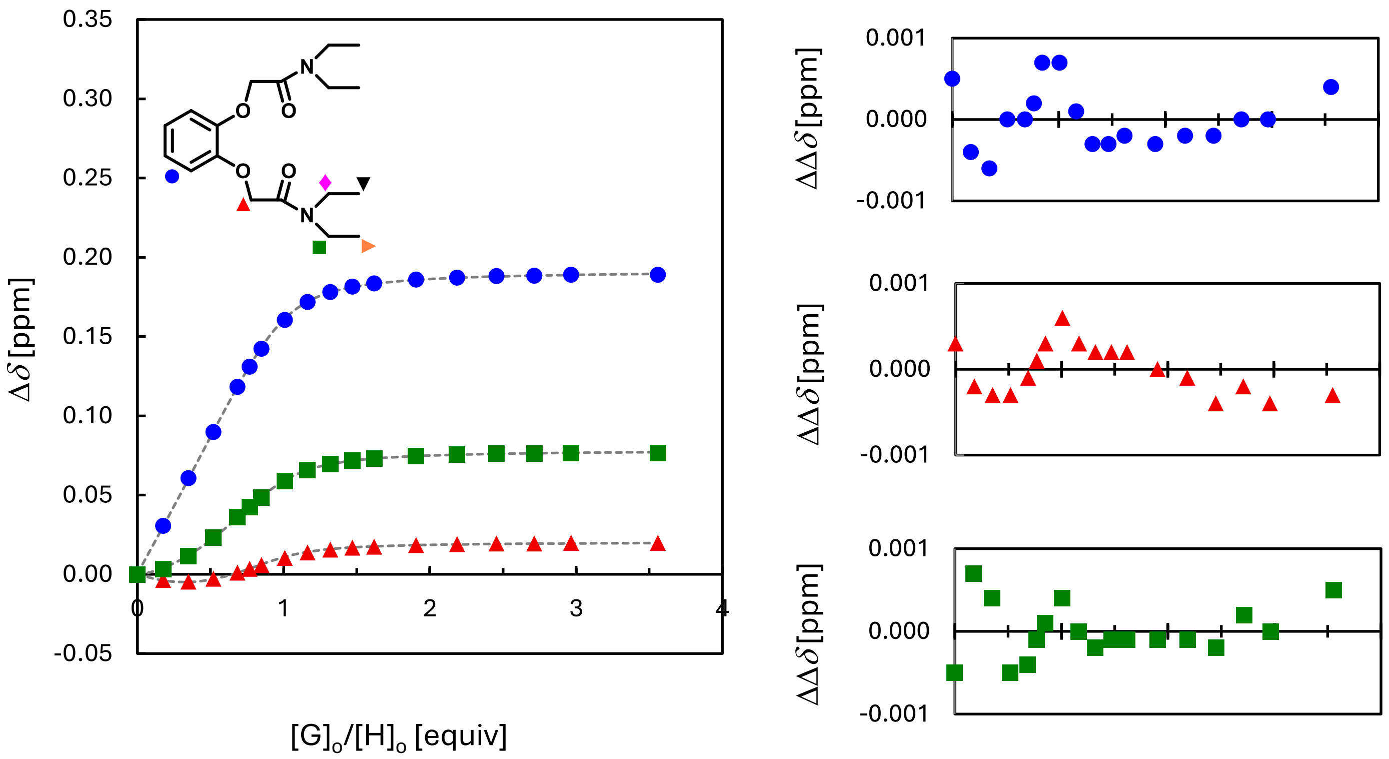

Figure S23. Experimental ^{1}H NMR chemical shift changes (symbols) and fitted binding isotherms (gray dashed lines) for titration of **1b** with NaOTf in 99.5% CD_3CN + 0.5% H_2O at 298 K (left), assuming 2:1 (host:guest) binding model; residual distribution for the corresponding shift (right).

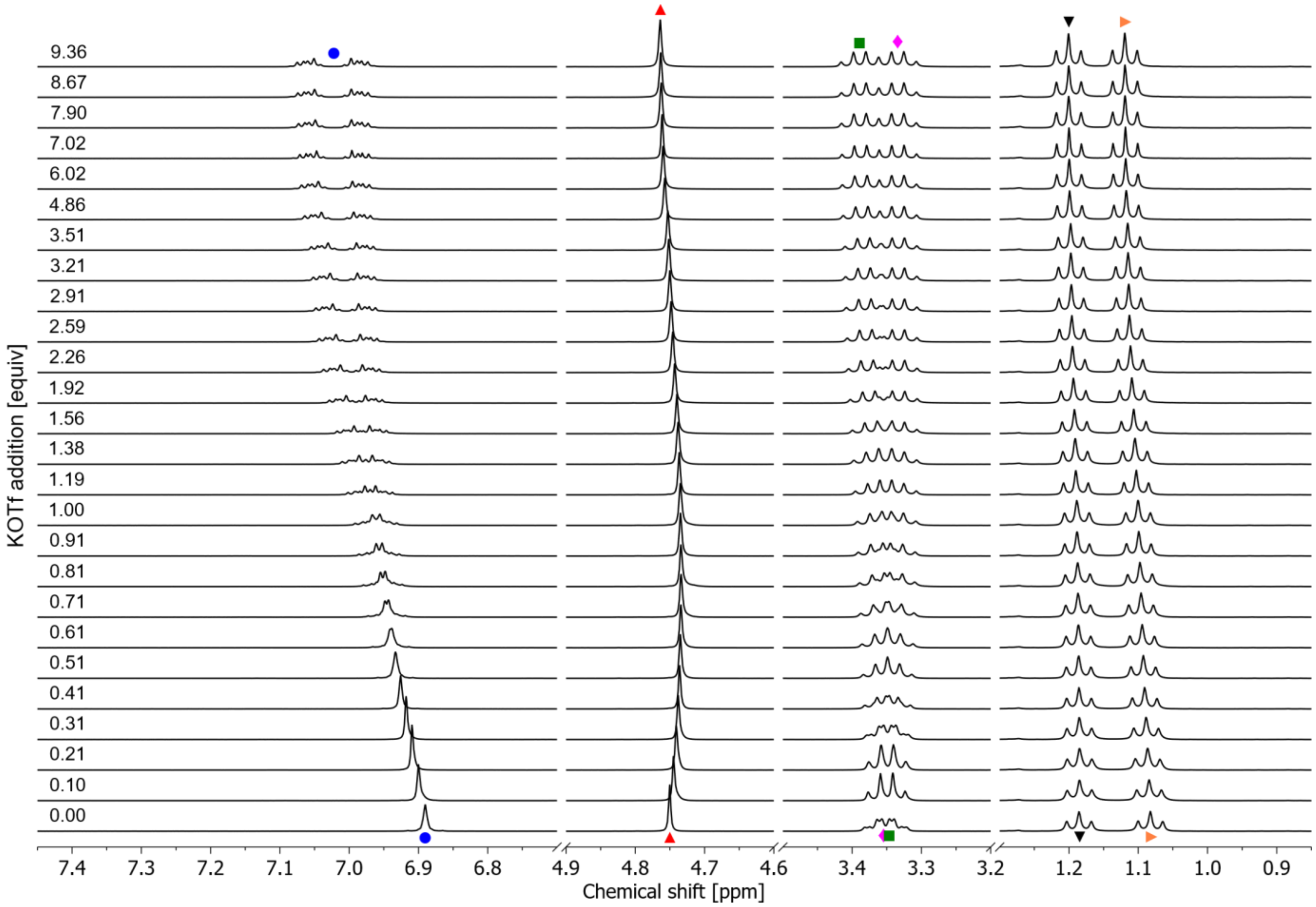

Figure S24. Stacked spectra from the ^{1}H NMR titration of **1b** with increasing amount of KOTf.

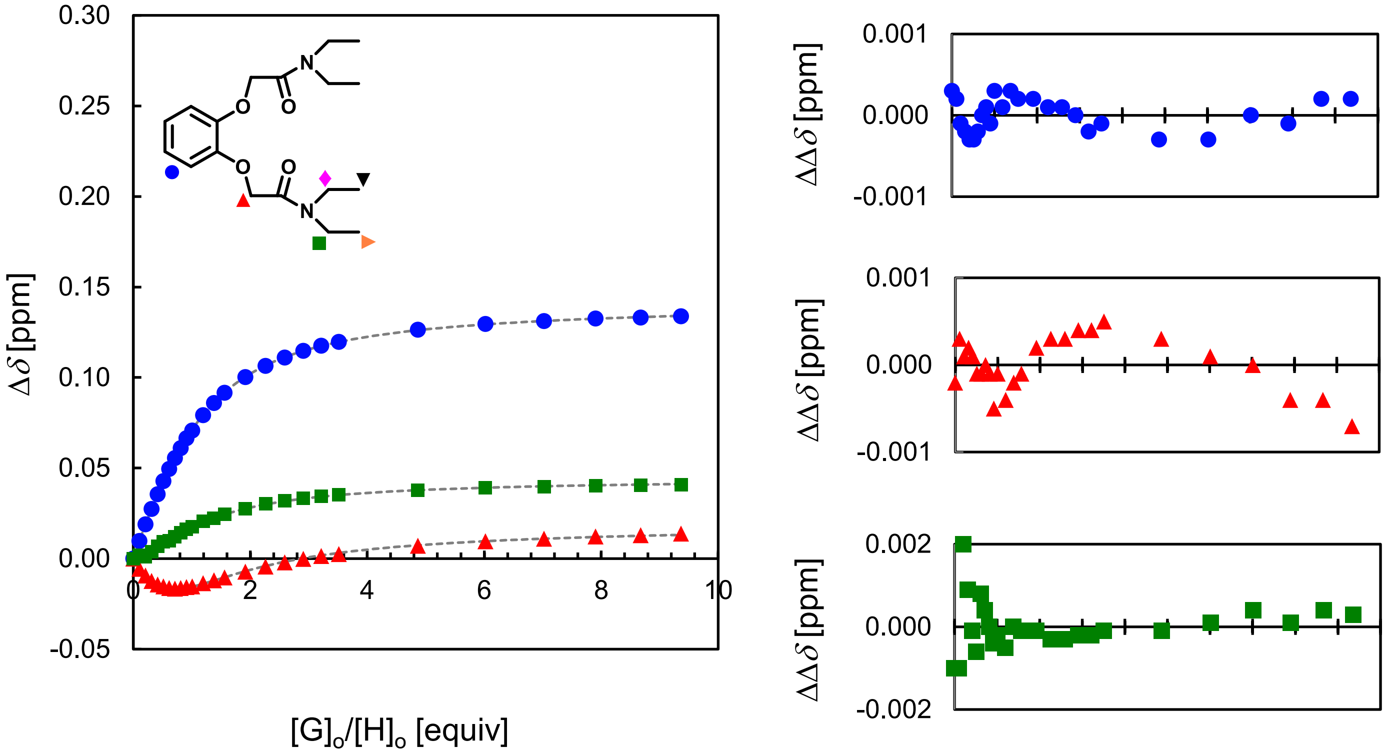

Figure S25. Experimental ^{1}H NMR chemical shift changes (symbols) and fitted binding isotherms (gray dashed lines) for titration of **1b** with KOTf in 99.5% CD_3CN + 0.5% H_2O at 298 K (left), assuming 2:1 (host:guest) binding model; residual distribution for the corresponding shift (right).

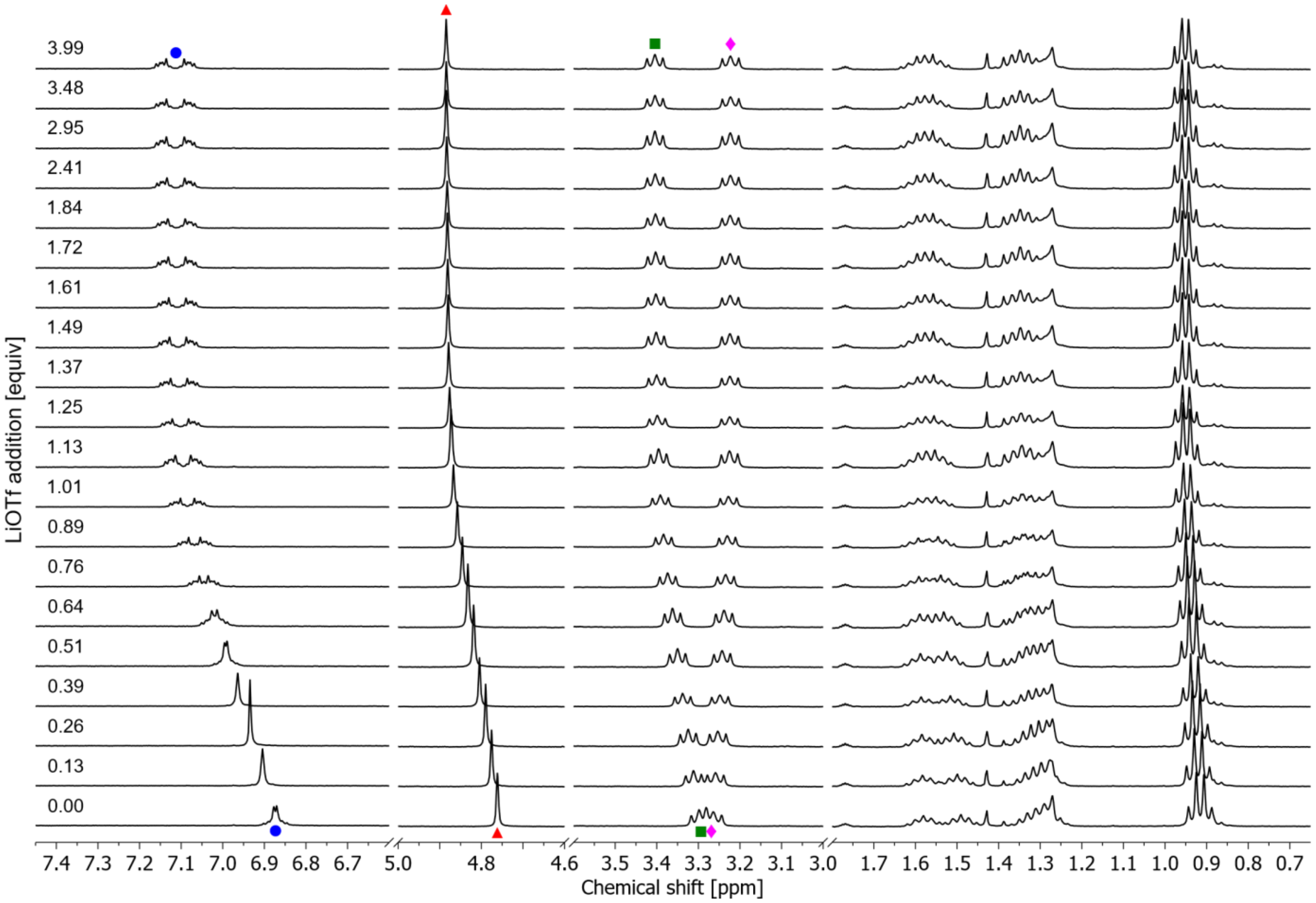

Figure S26. Stacked spectra from the ^{1}H NMR titration of **1c** with increasing amount of LiOTf.

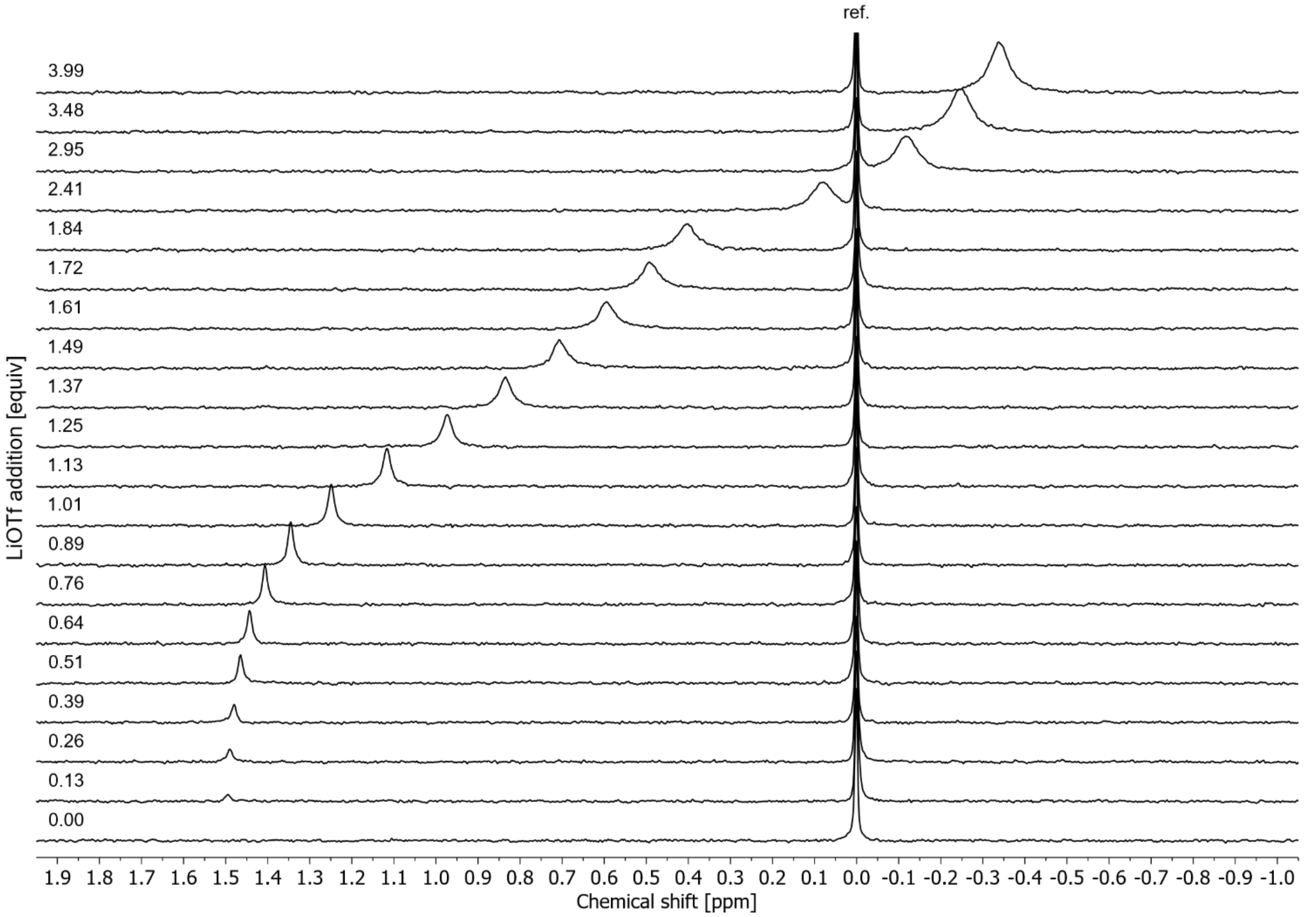

Figure S27. Stacked spectra from the ^{7}Li NMR titration of **1c** with increasing amount of LiOTf.

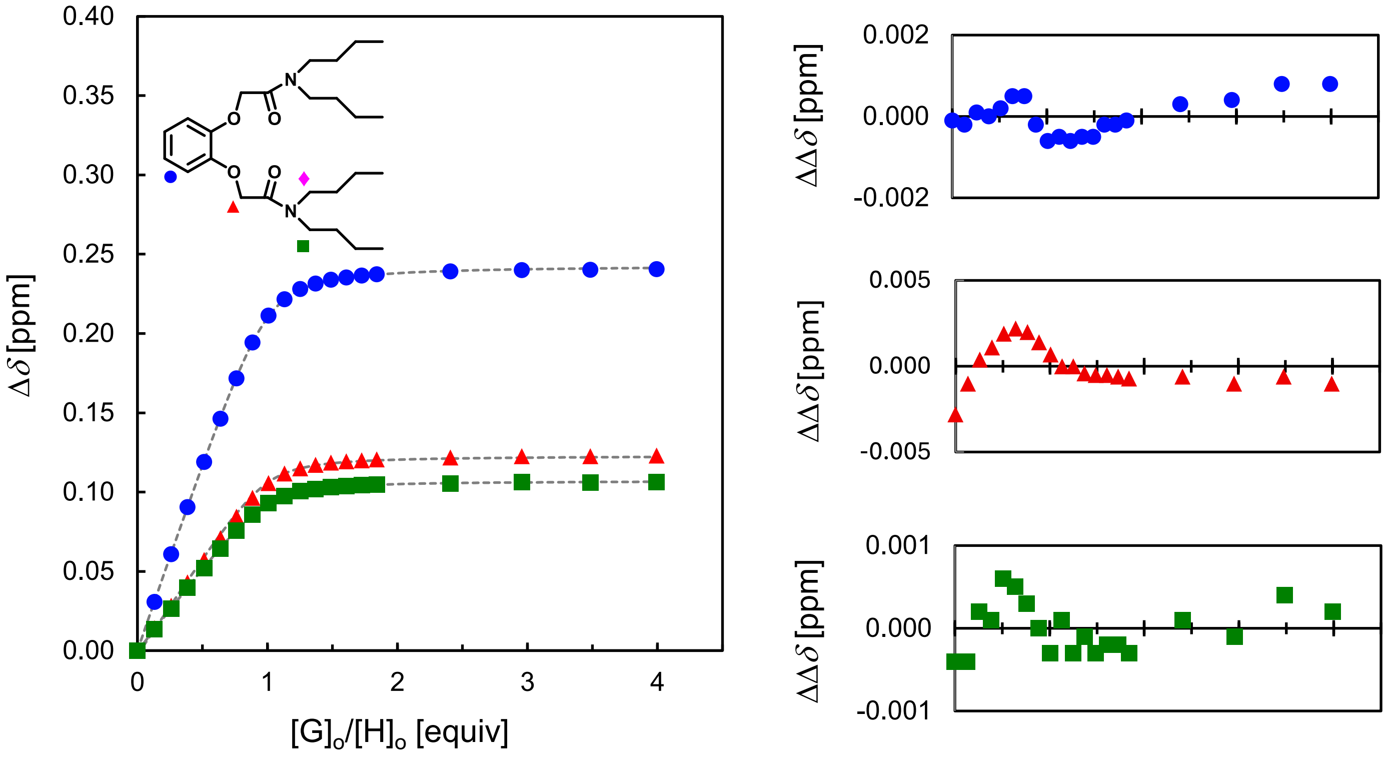

Figure S28. Experimental ^{1}H NMR chemical shift changes (symbols) and fitted binding isotherms (gray dashed lines) for titration of **1c** with LiOTf in 99.5% CD_3CN + 0.5% H_2O at 298 K (left), assuming 1:1 binding model; residual distribution for the corresponding shift (right).

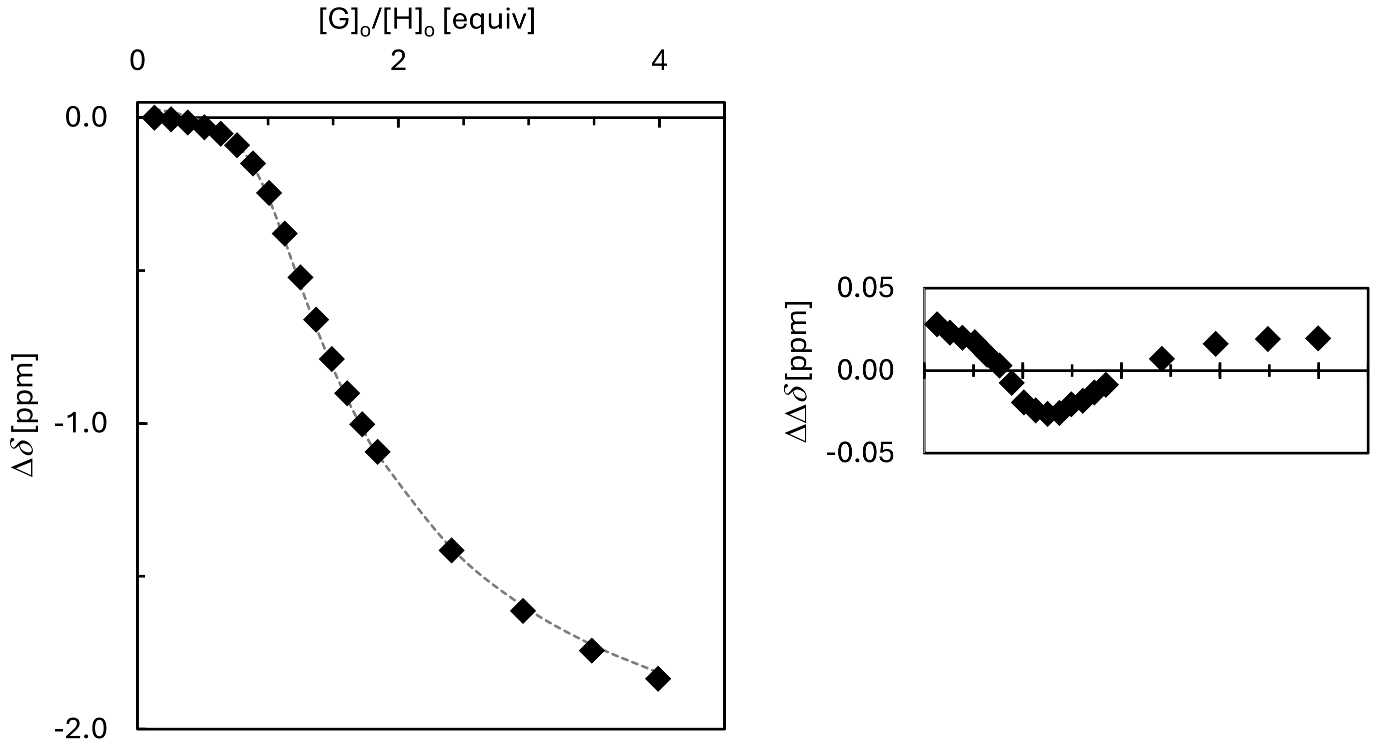

Figure S29. Experimental ^{7}Li NMR chemical shift changes (symbols) and calculated binding isotherms (gray dashed lines) for titration of **1c** with LiOTf in 99.5% CD_3CN + 0.5% H_2O at 298 K (left), assuming 1:1 binding model; residual distribution for the corresponding shift (right).

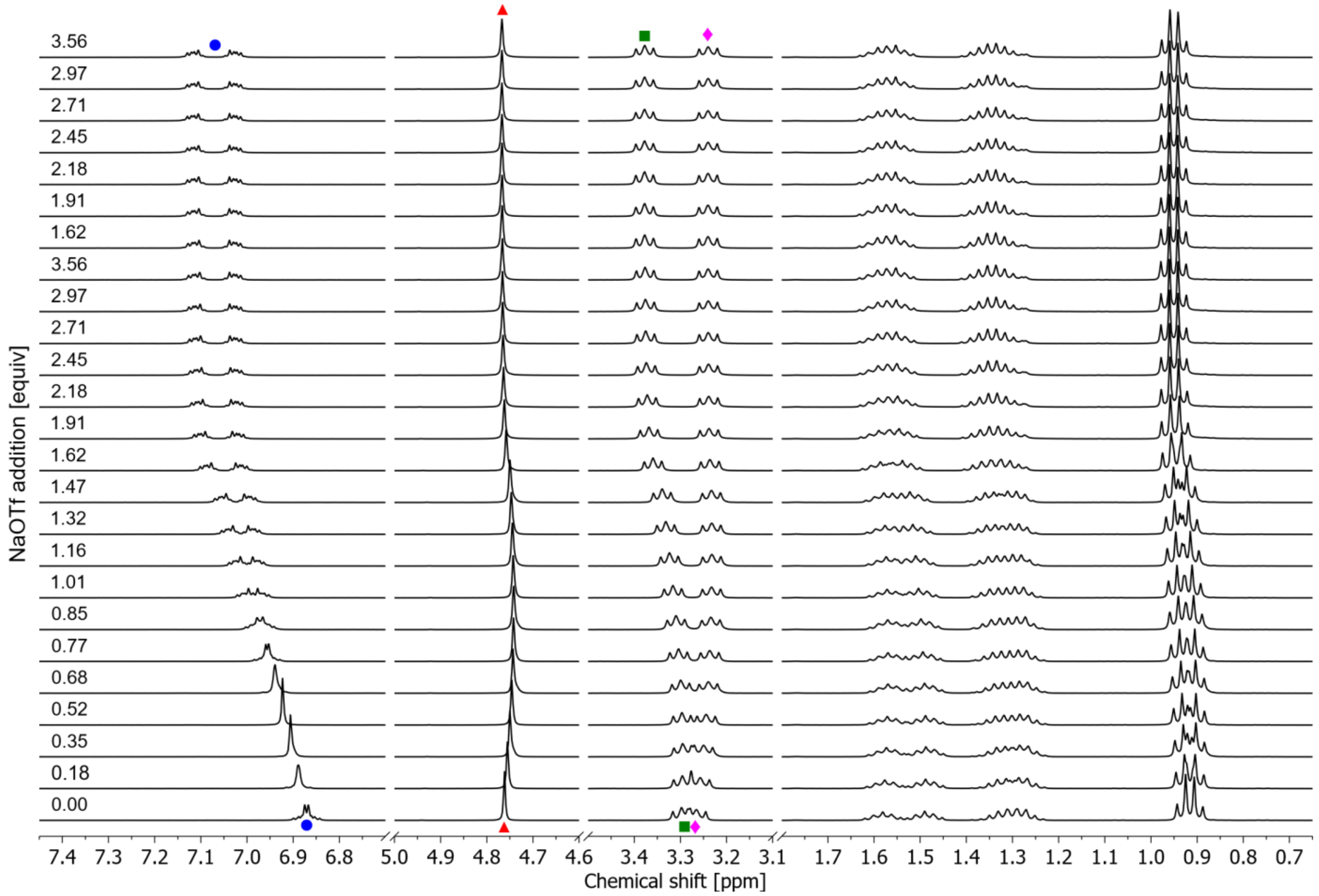

Figure S30. Stacked spectra from the ^{1}H NMR titration of **1c** with increasing amount of NaOTf.

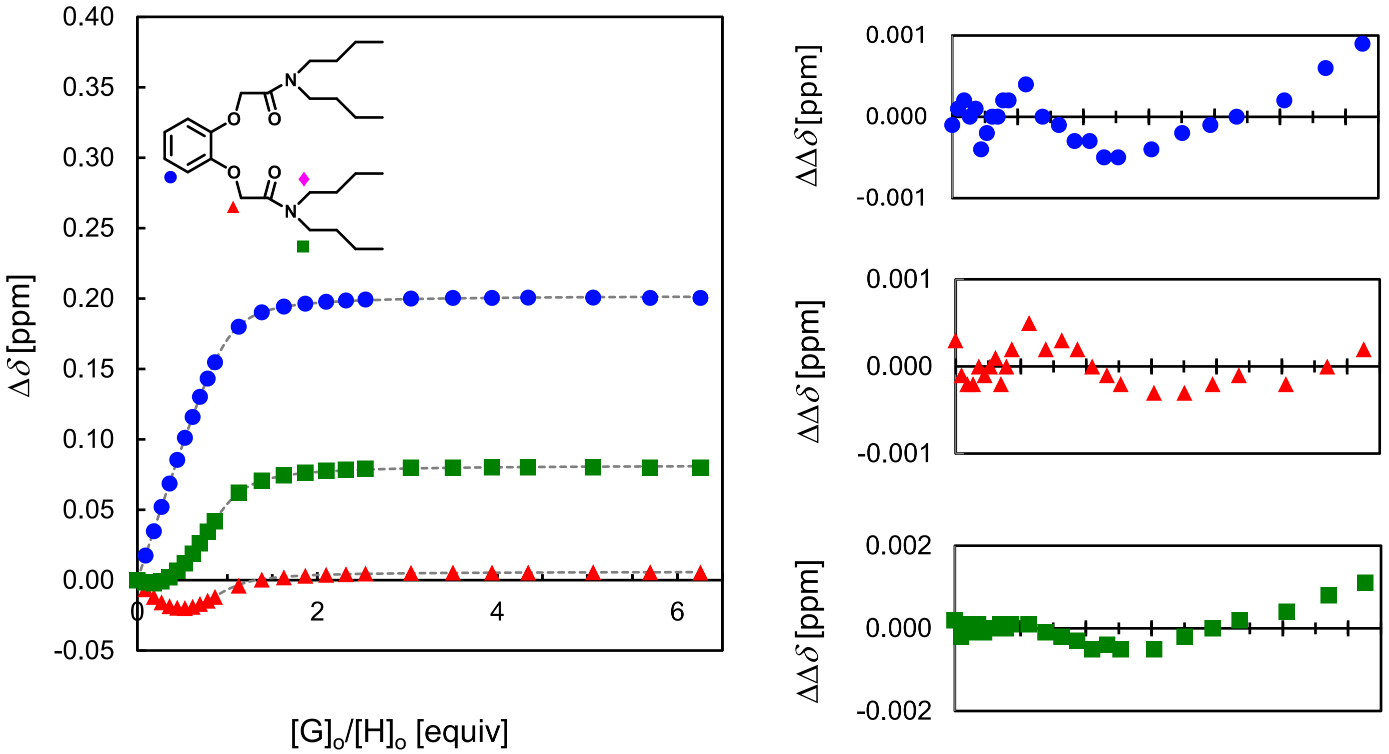

Figure S31. Experimental ^{1}H NMR chemical shift changes (symbols) and fitted binding isotherms (gray dashed lines) for titration of **1c** with NaOTf in 99.5% CD_3CN + 0.5% H_2O at 298 K (left), assuming 2:1 (host:guest) binding model; residual distribution for the corresponding shift (right).

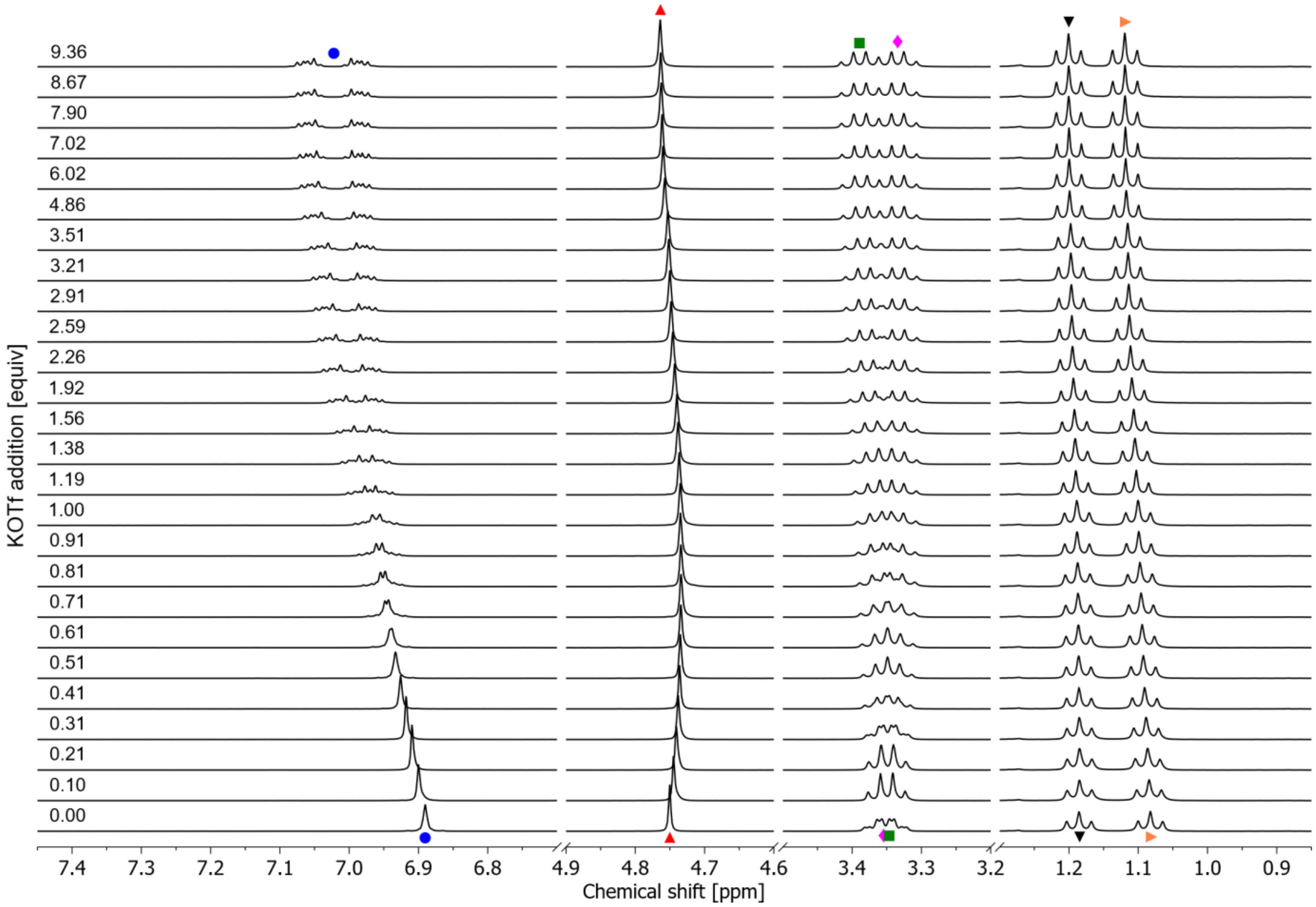

Figure S32. Stacked spectra from the ^{1}H NMR titration of **1c** with increasing amount of KOTf.

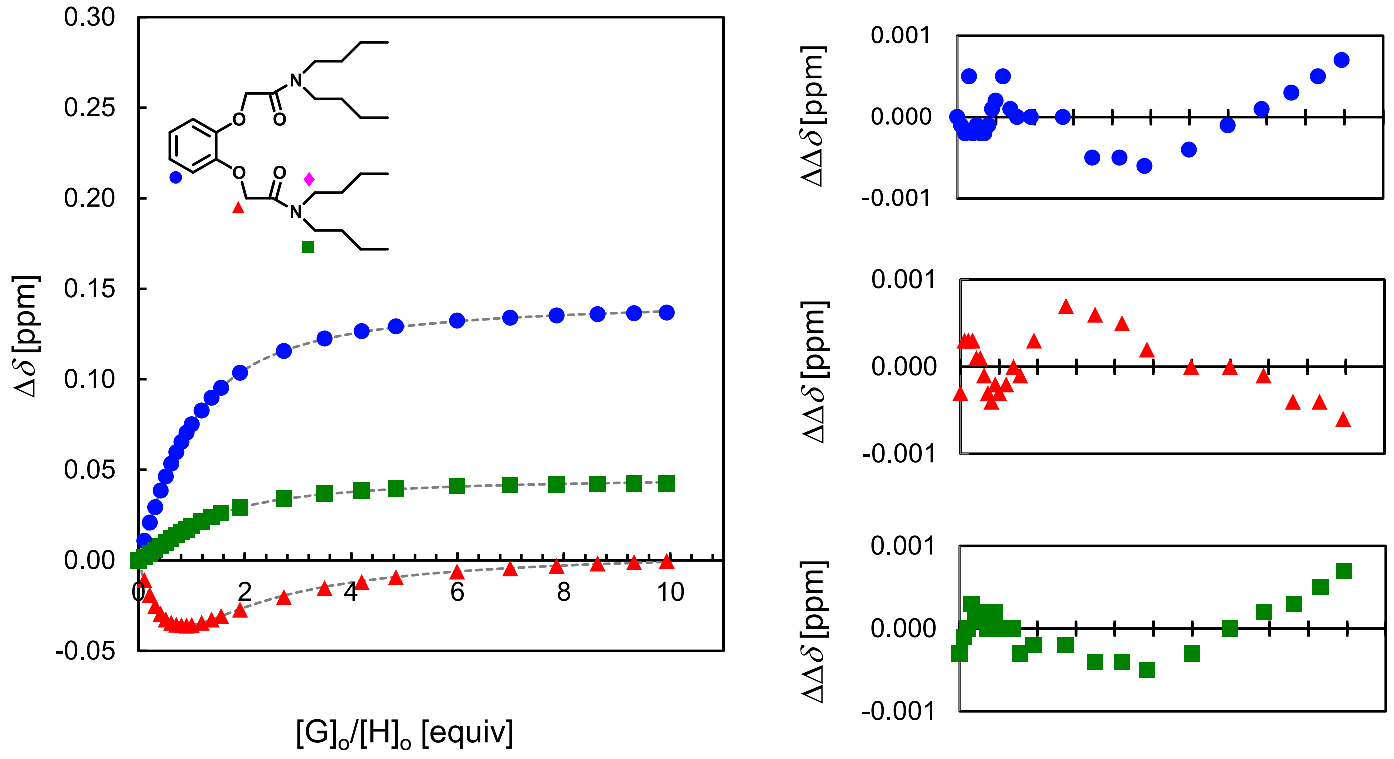

Figure S33. Experimental ^{1}H NMR chemical shift changes (symbols) and fitted binding isotherms (gray dashed lines) for titration of **1c** with KOTf in 99.5% CD_3CN + 0.5% H_2O at 298 K (left), assuming 2:1 (host:guest) binding model; residual distribution for the corresponding shift (right).

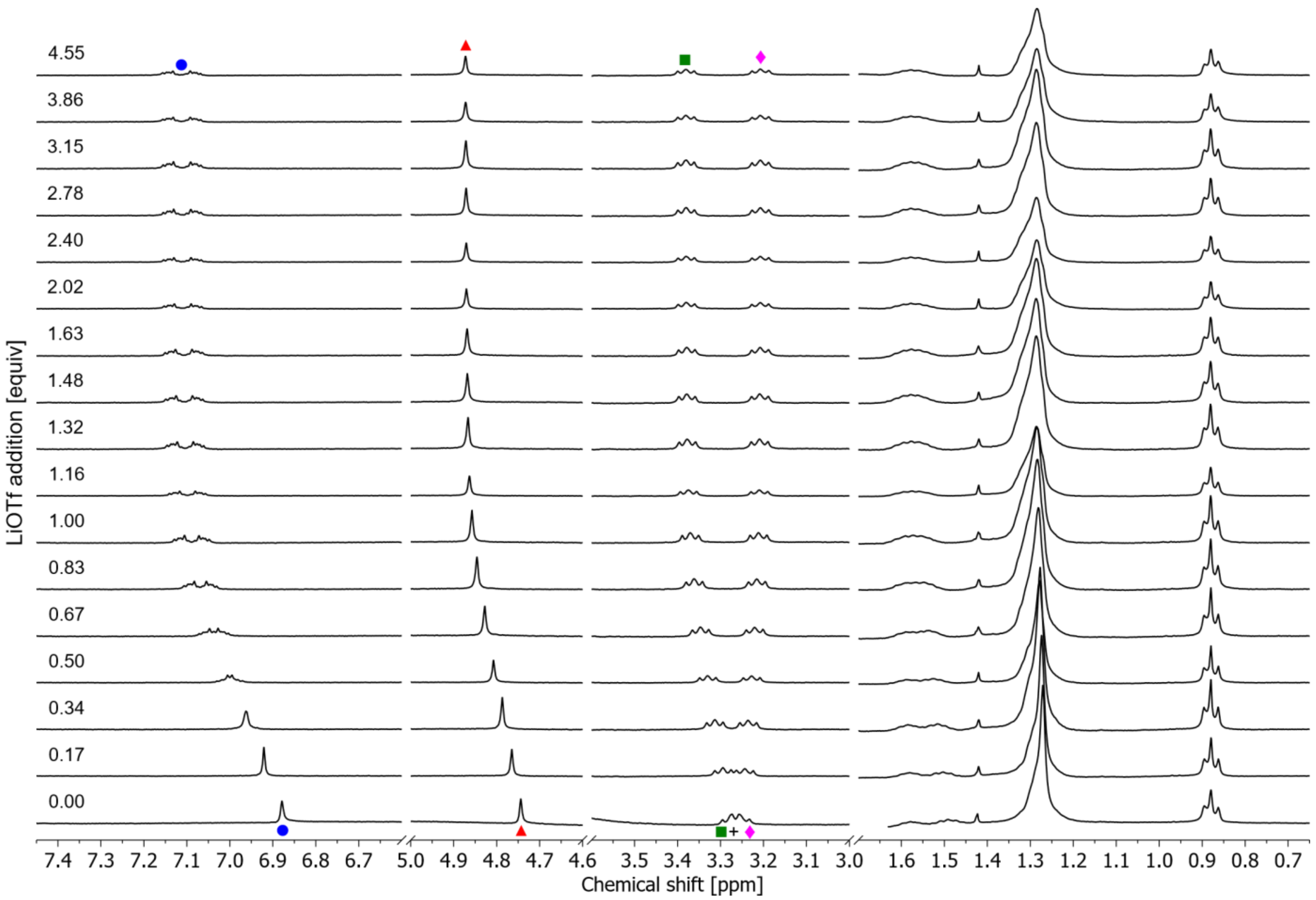

Figure S34. Stacked spectra from the ^{1}H NMR titration of **1d** with increasing amount of LiOTf.

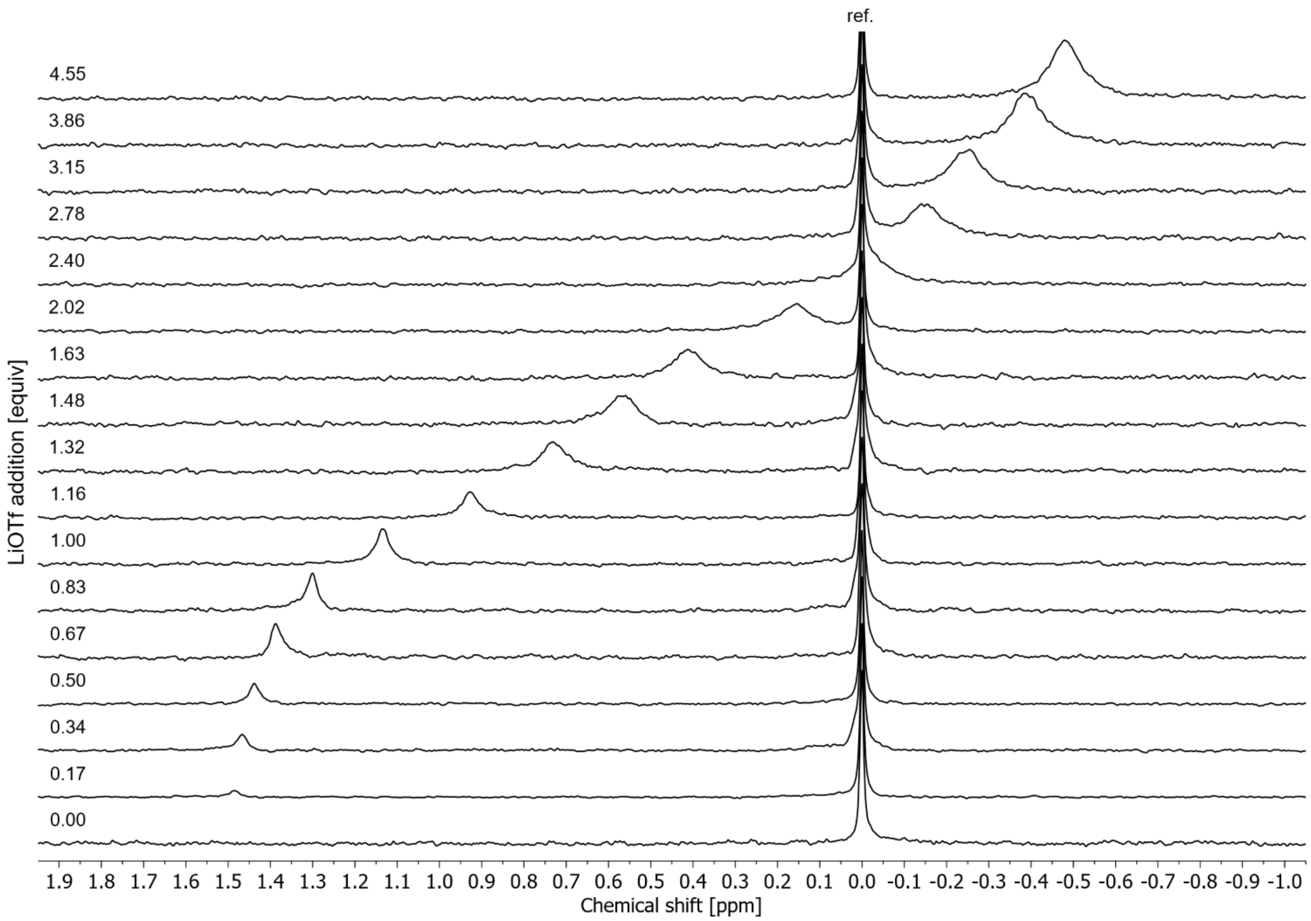

Figure S35. Stacked spectra from the ^{7}Li NMR titration of **1d** with increasing amount of LiOTf.

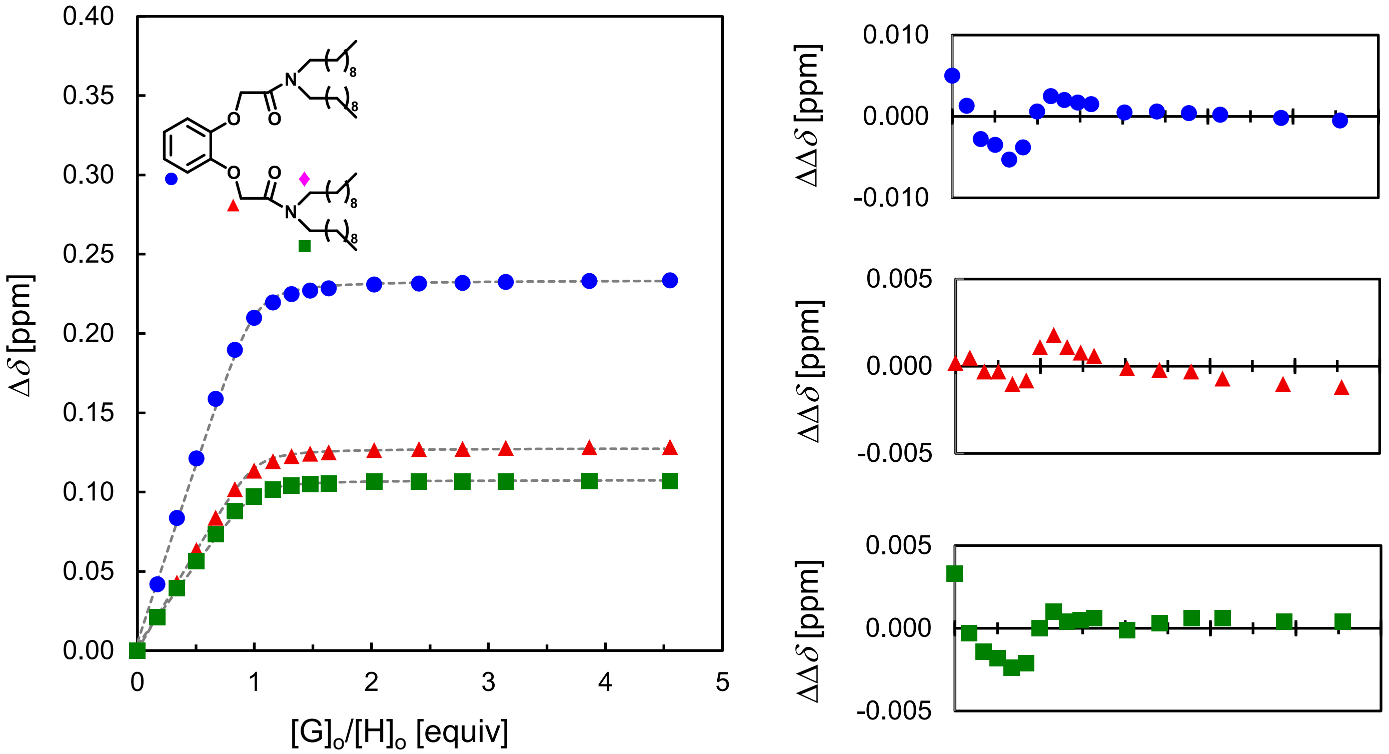

Figure S36. Experimental ^{1}H NMR chemical shift changes (symbols) and fitted binding isotherms (gray dashed lines) for titration of **1d** with LiOTf in 99.5% CD_3CN + 0.5% H_2O at 298 K (left), assuming 1:1 binding model; residual distribution for the corresponding shift (right).

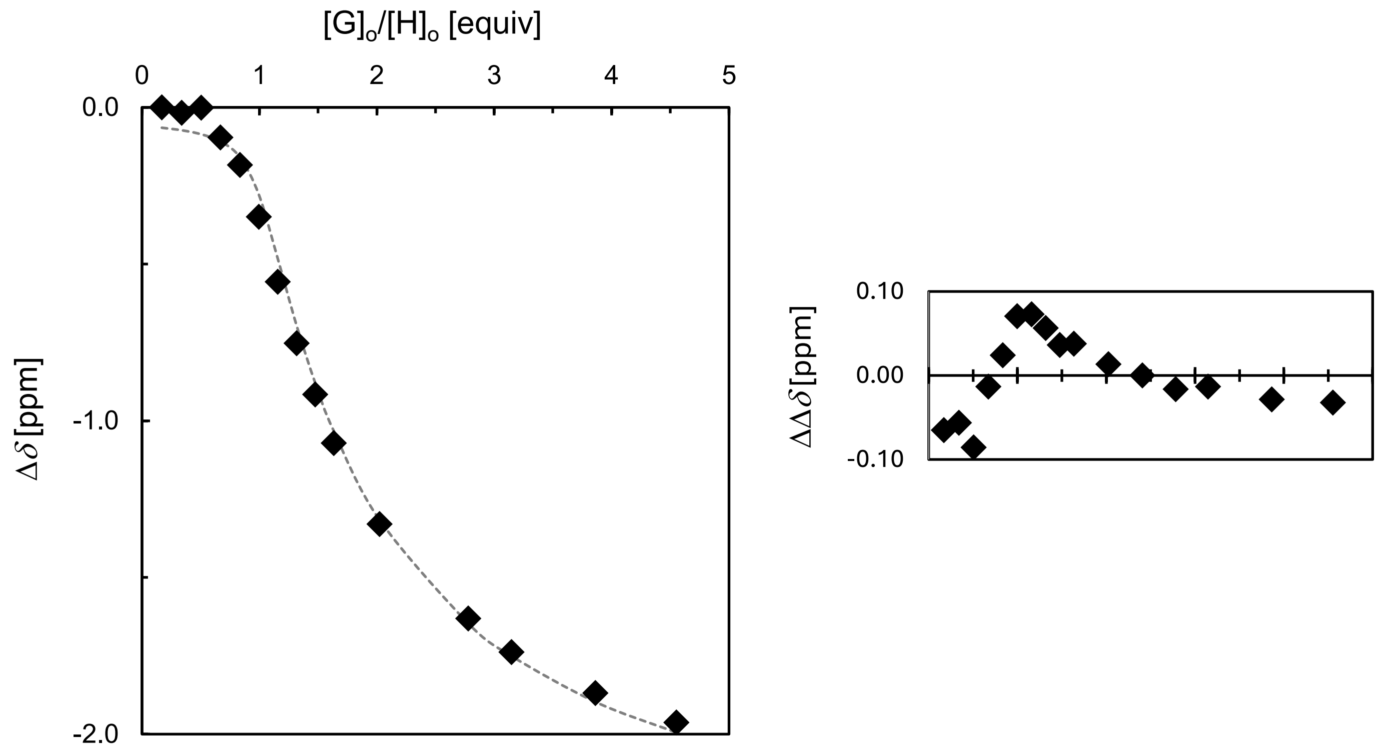

Figure S37. Experimental ^{7}Li NMR chemical shift changes (symbols) and calculated binding isotherms (gray dashed lines) for titration of **1d** with LiOTf in 99.5% CD_3CN + 0.5% H_2O at 298 K (left), assuming 1:1 binding model; residual distribution for the corresponding shift (right).

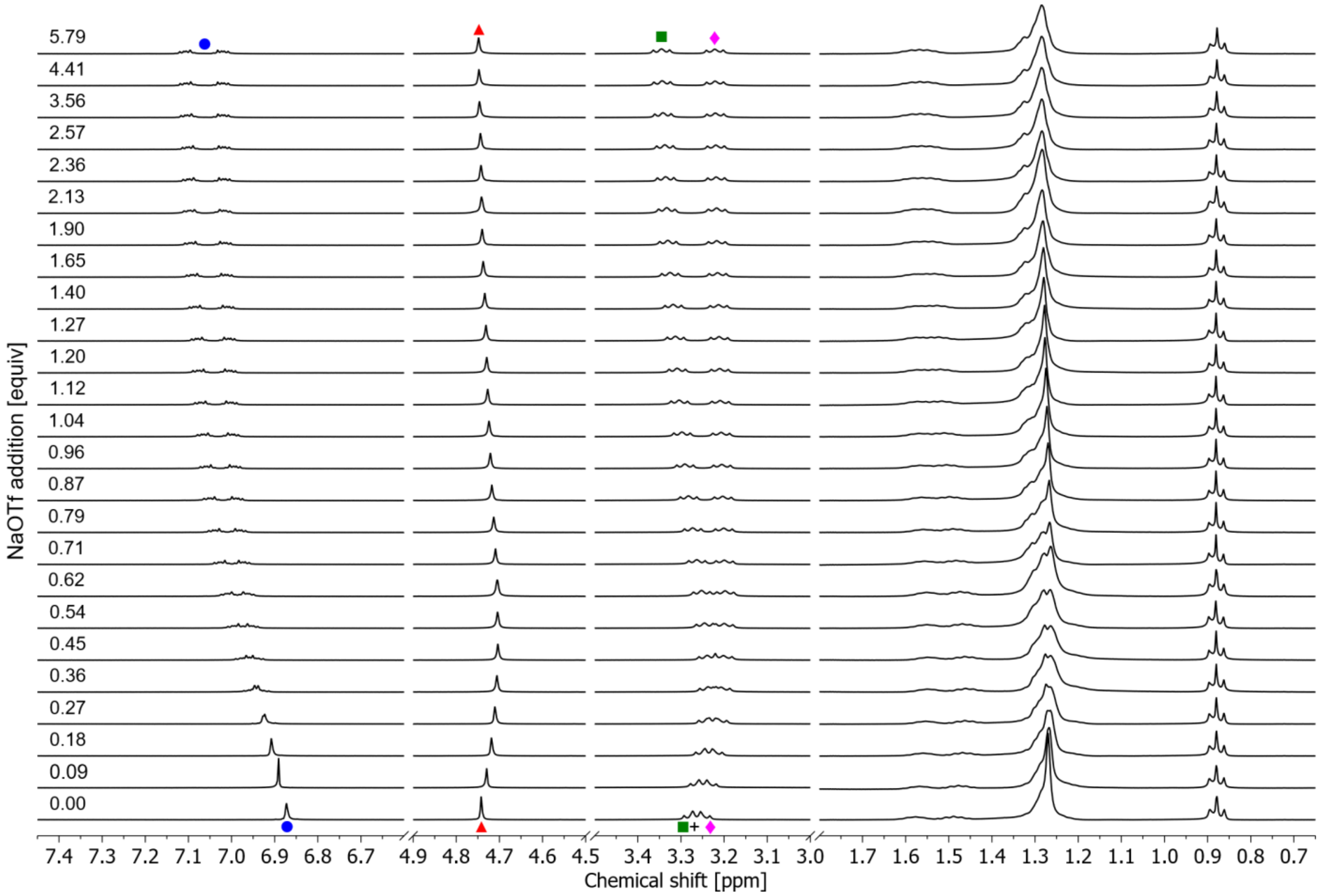

Figure S38. Stacked spectra from the ^{1}H NMR titration of **1d** with increasing amount of NaOTf.

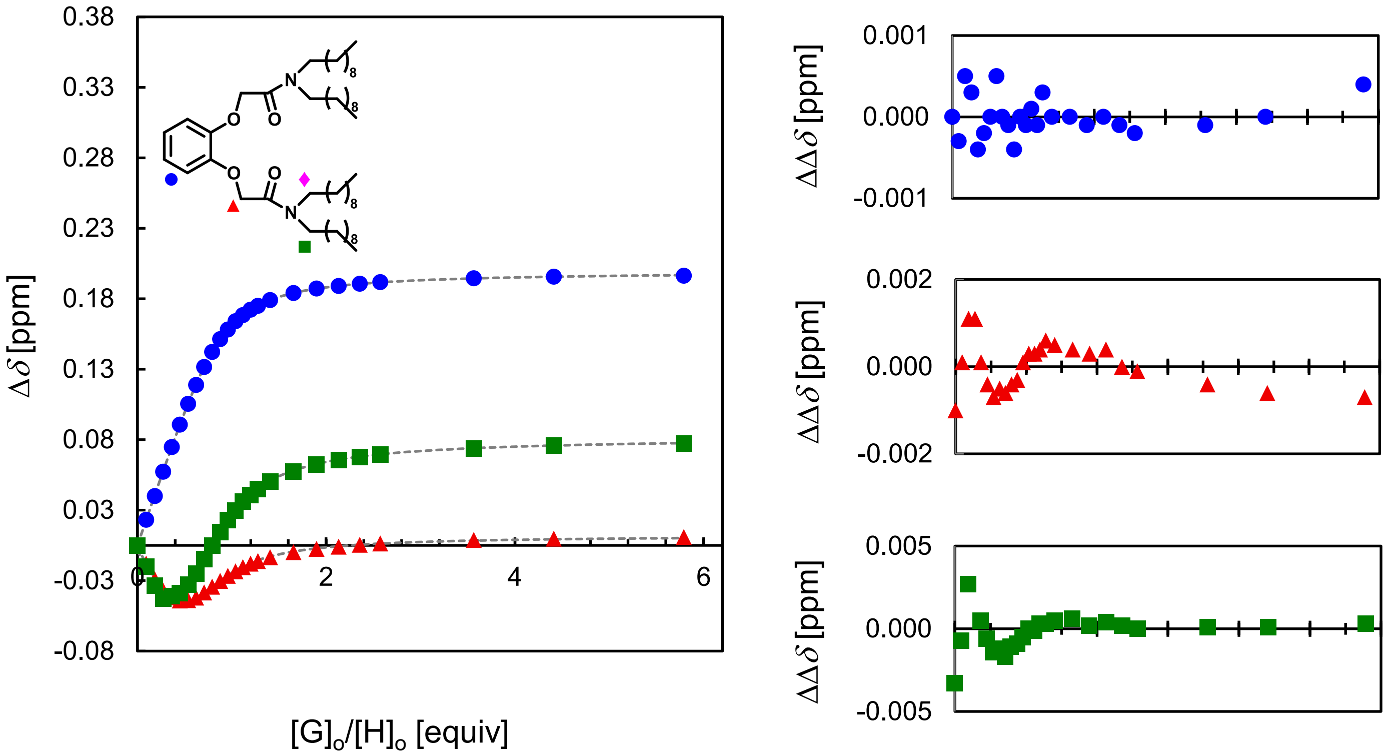

Figure S39. Experimental ^{1}H NMR chemical shift changes (symbols) and fitted binding isotherms (gray dashed lines) for titration of **1d** with NaOTf in 99.5% CD_3CN + 0.5% H_2O at 298 K (left), assuming 2:1 (host:guest) binding model; residual distribution for the corresponding shift (right).

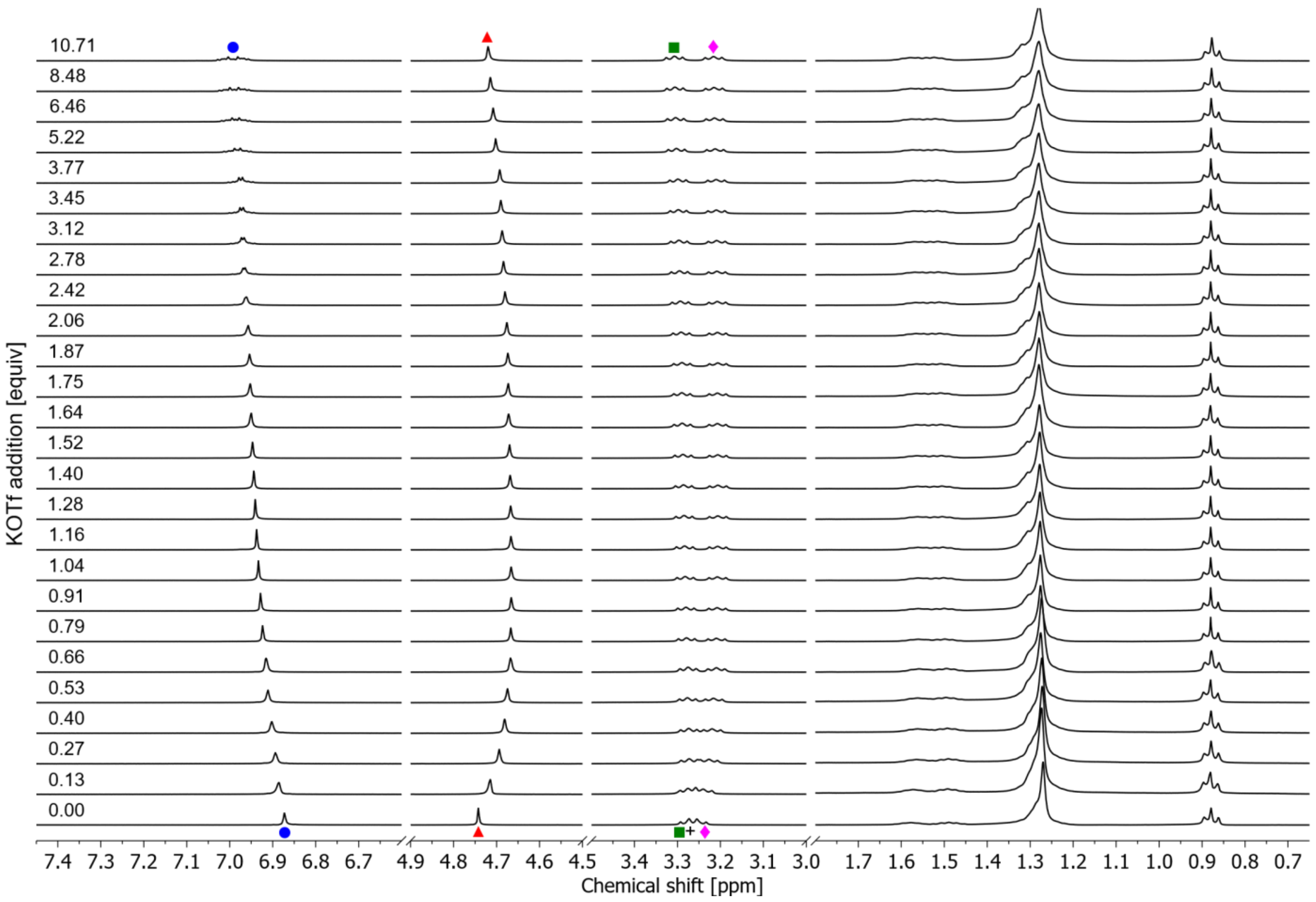

Figure S40. Stacked spectra from the ^{1}H NMR titration of **1d** with increasing amount of KOTf.

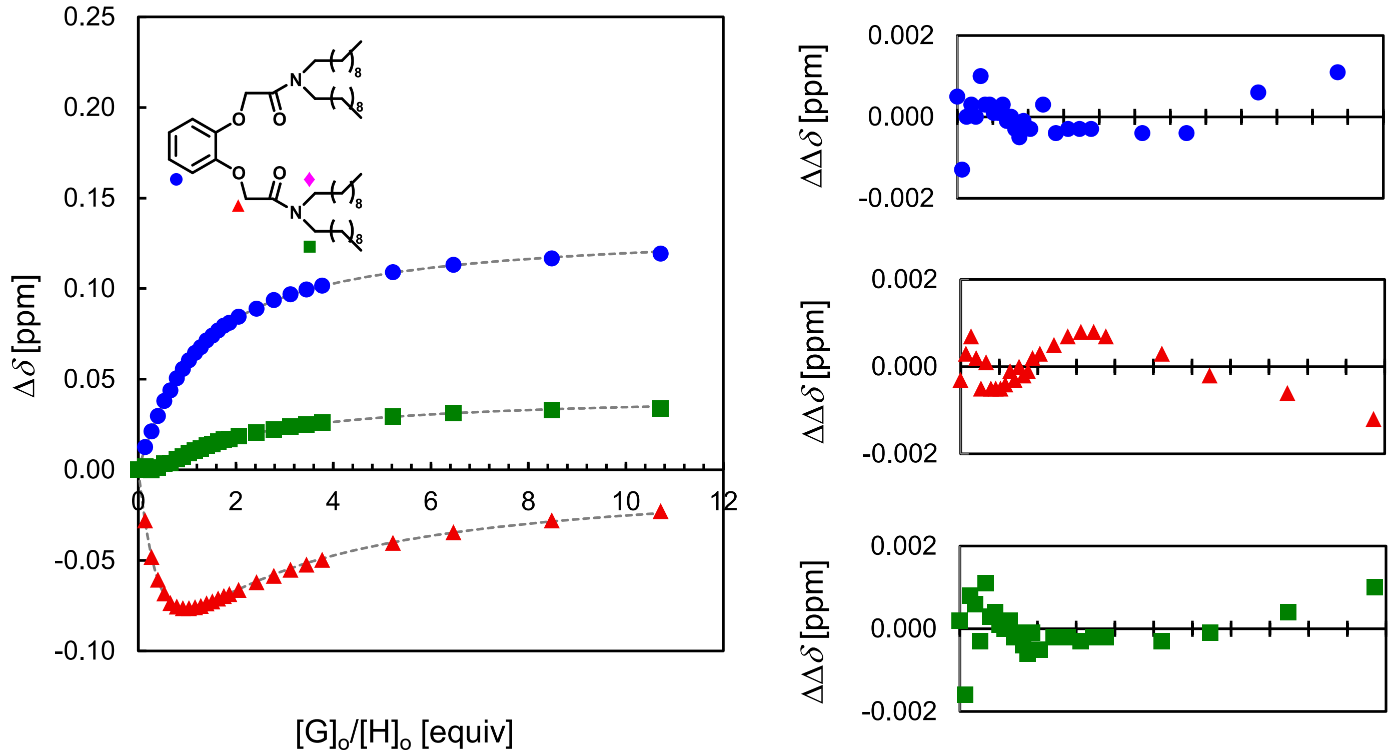

Figure S41. Experimental ^{1}H NMR chemical shift changes (symbols) and fitted binding isotherms (gray dashed lines) for titration of **1d** with KOTf in 99.5% CD_3CN + 0.5% H_2O at 298 K (left), assuming 2:1 (host:guest) binding model; residual distribution for the corresponding shift (right).

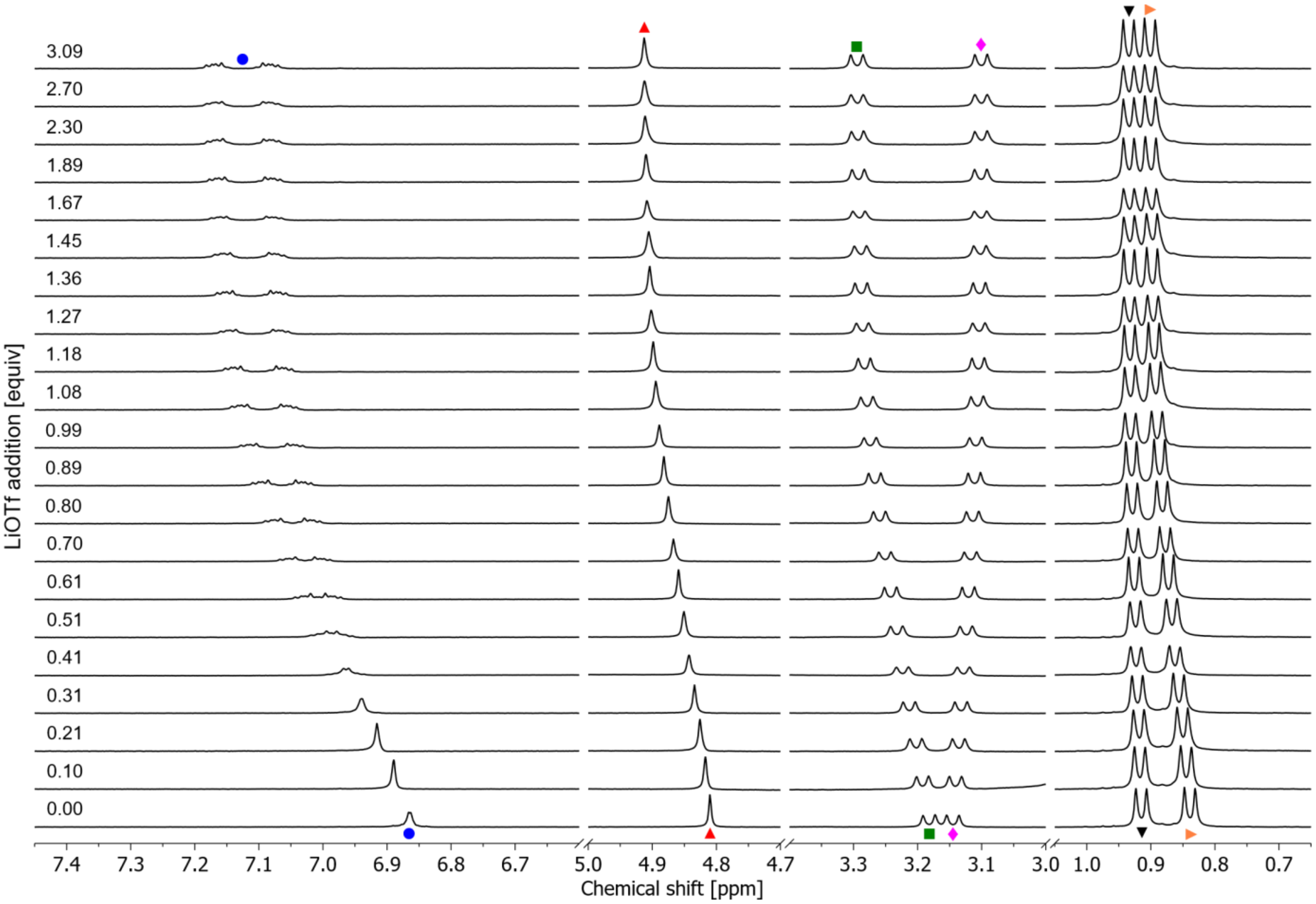

Figure S42. Stacked spectra from the ^{1}H NMR titration of **1e** with increasing amount of LiOTf.

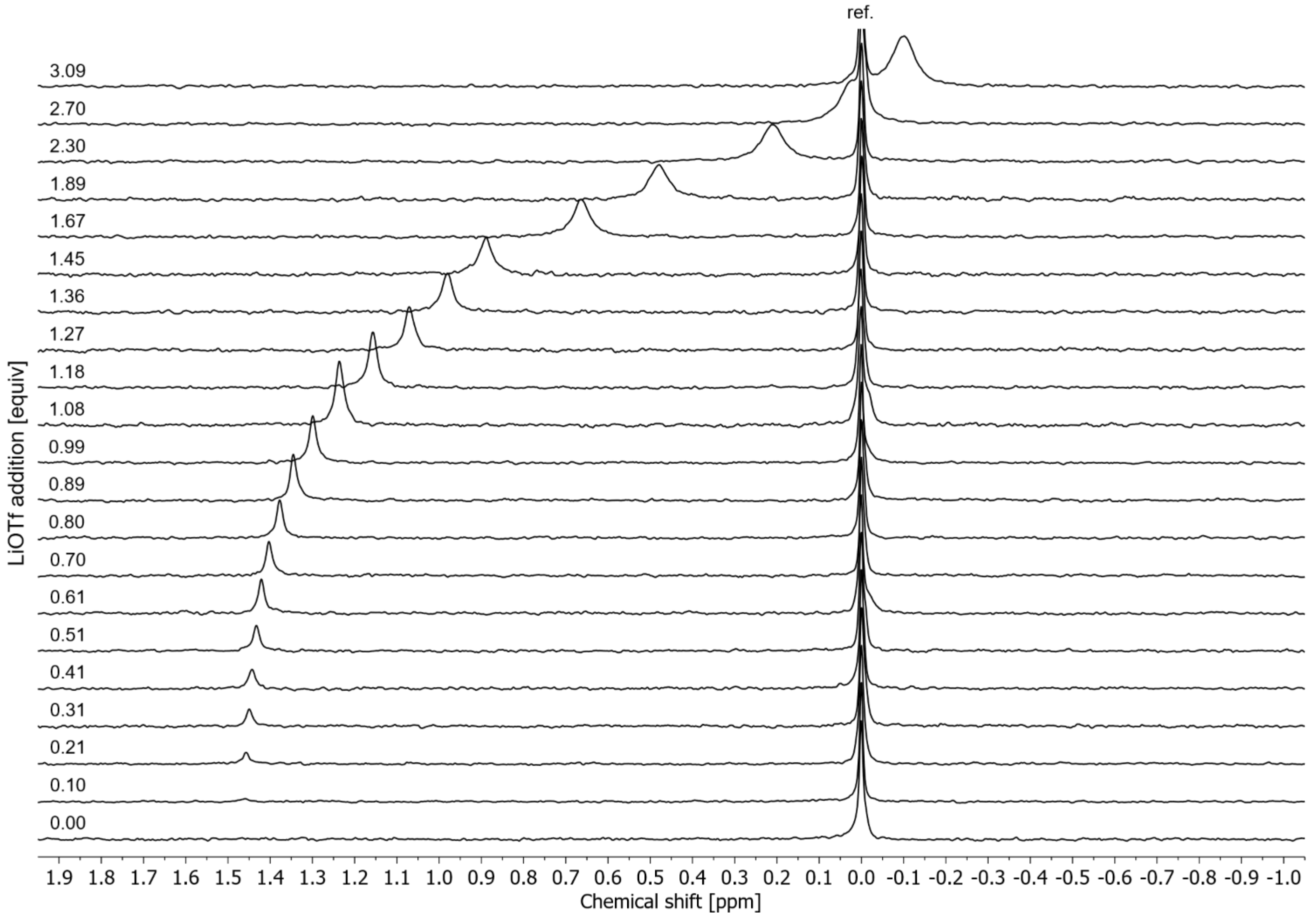

Figure S43. Stacked spectra from the ^{7}Li NMR titration of **1e** with increasing amount of LiOTf.

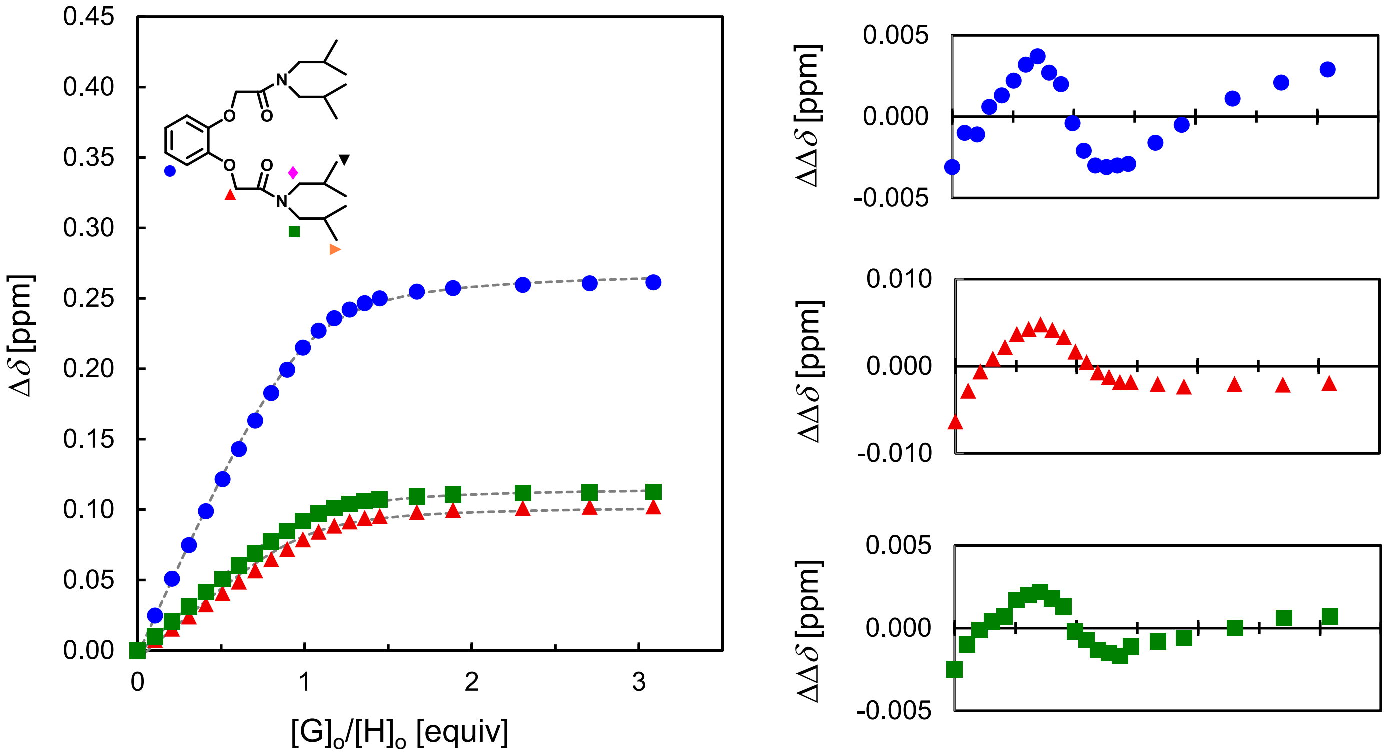

Figure S44. Experimental ^{1}H NMR chemical shift changes (symbols) and calculated binding isotherms (gray dashed lines) for titration of **1e** with LiOTf in 99.5% CD_3CN + 0.5% H_2O at 298 K (left), assuming 1:1 binding model; residual distribution for the corresponding shift (right).

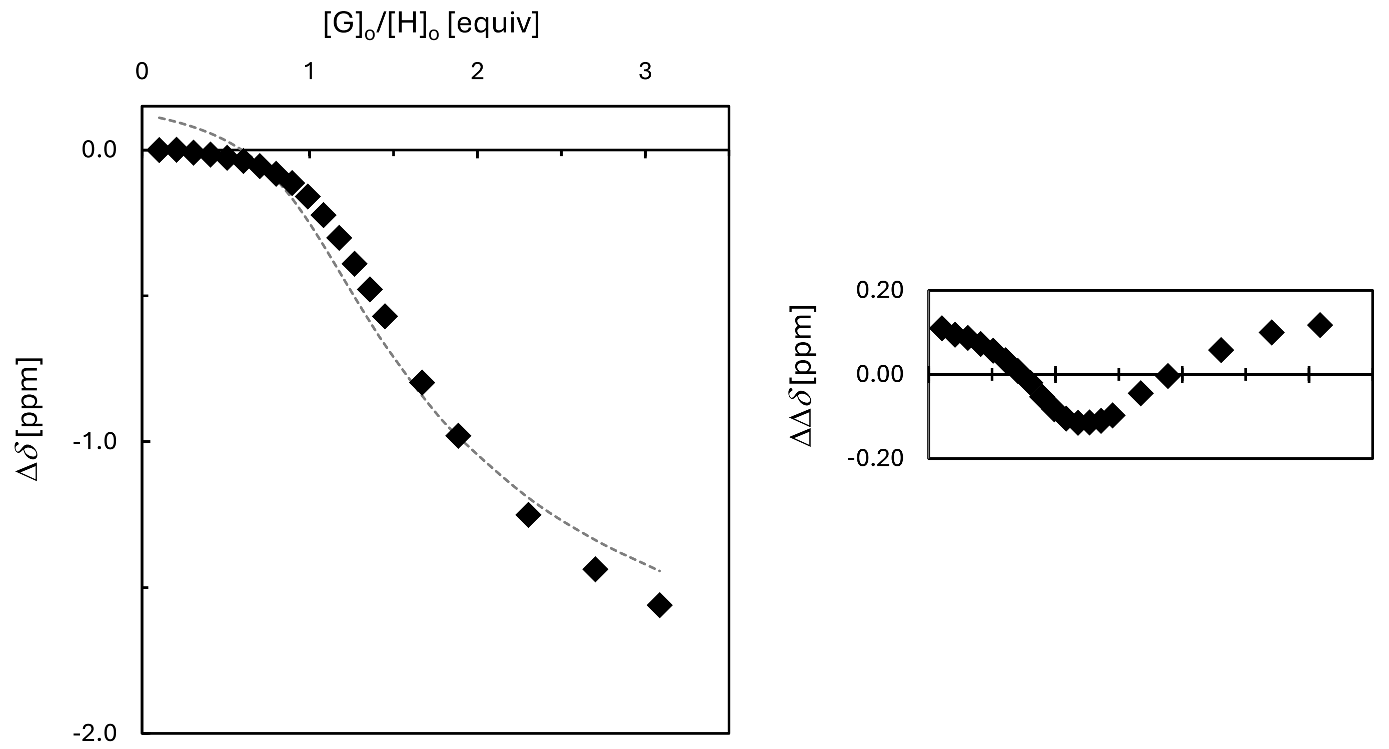

Figure S45. Experimental ^{7}Li NMR chemical shift changes (symbols) and calculated binding isotherms (gray dashed lines) for titration of **1e** with LiOTf in 99.5% CD_3CN + 0.5% H_2O at 298 K (left), assuming 1:1 binding model; residual distribution for the corresponding shift (right).

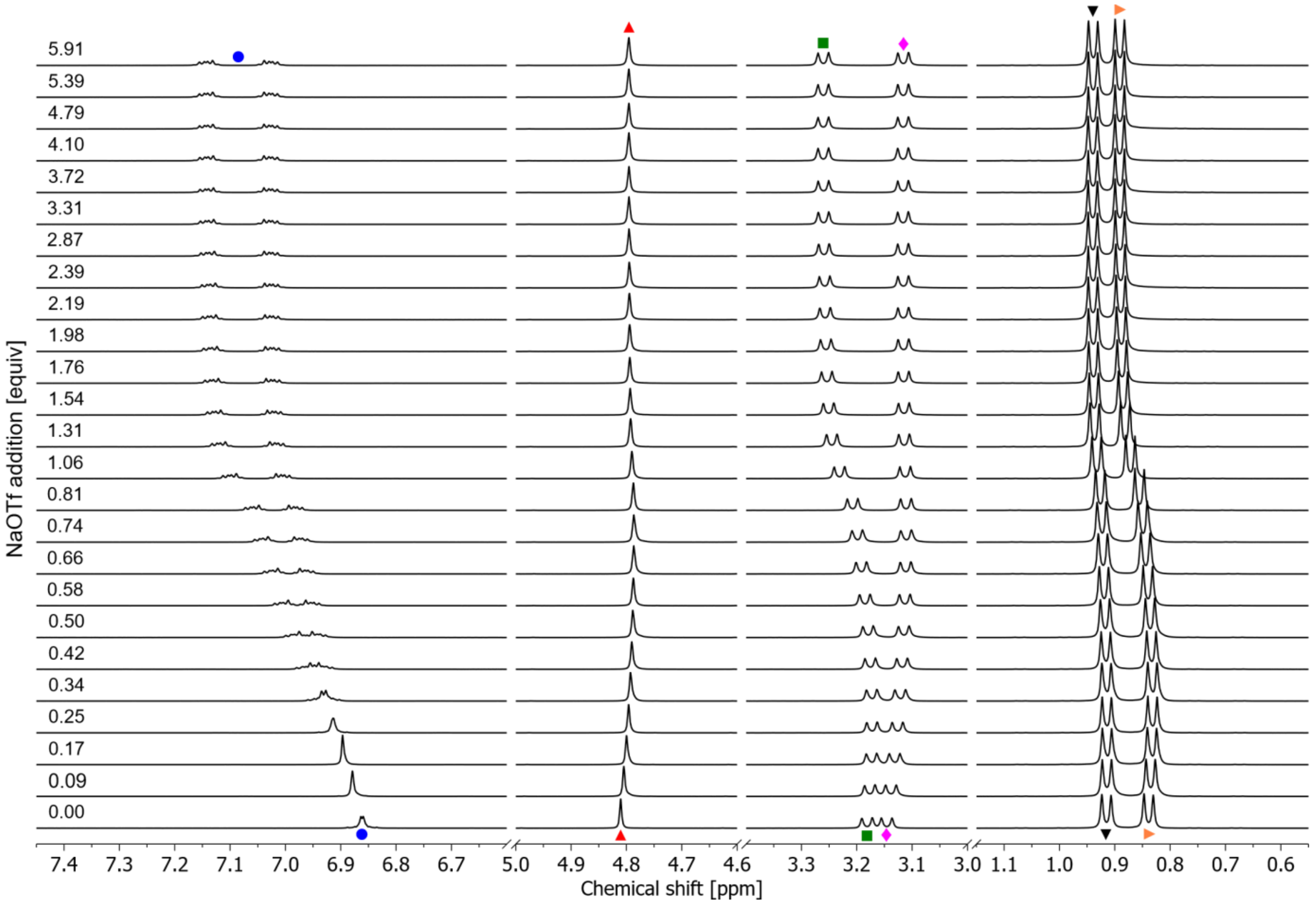

Figure S46. Stacked spectra from the ^{1}H NMR titration of **1e** with increasing amount of NaOTf.

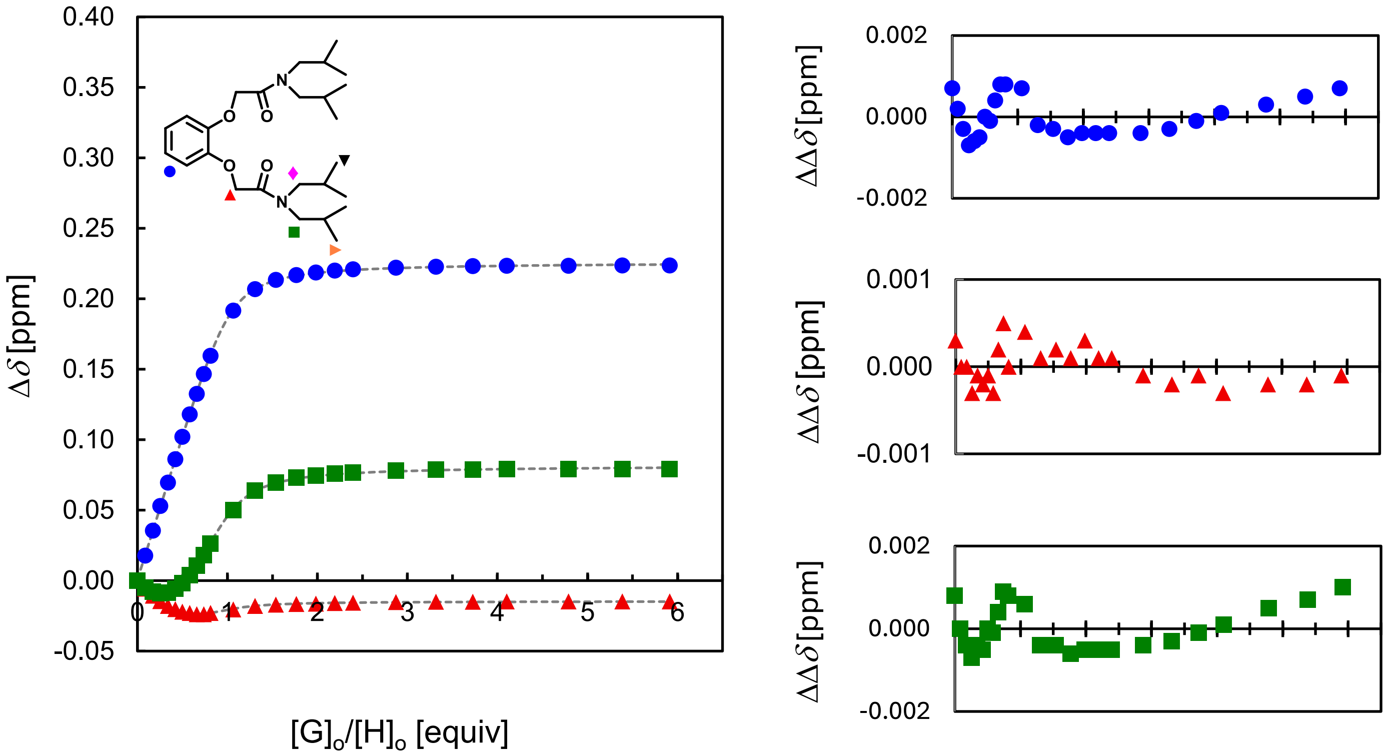

Figure S47. Experimental ^{1}H NMR chemical shift changes (symbols) and fitted binding isotherms (gray dashed lines) for titration of **1e** with NaOTf in 99.5% CD_3CN + 0.5% H_2O at 298 K (left), assuming 2:1 (host:guest) binding model; residual distribution for the corresponding shift (right).

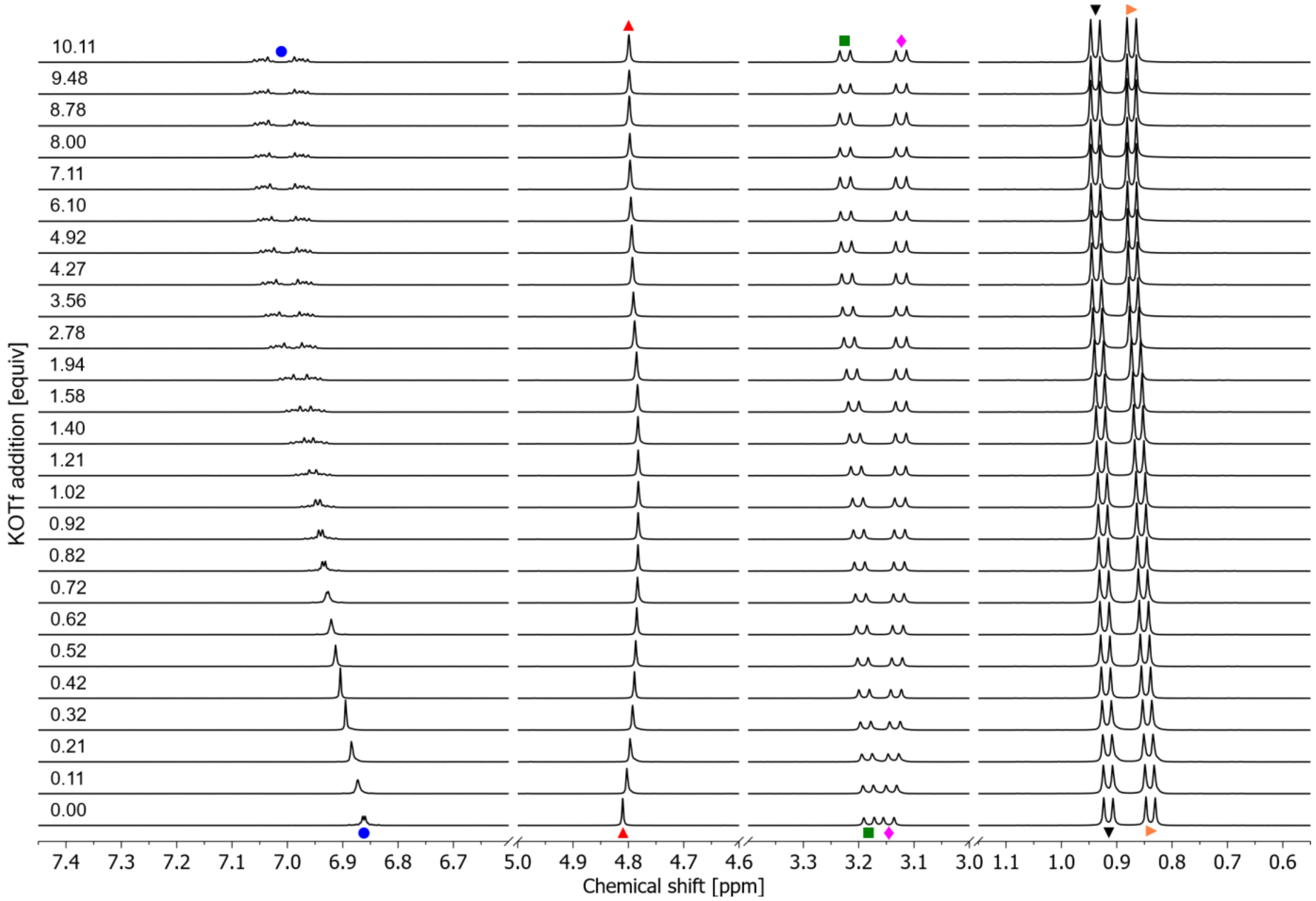

Figure S48. Stacked spectra from the ^{1}H NMR titration of **1e** with increasing amount of KOTf.

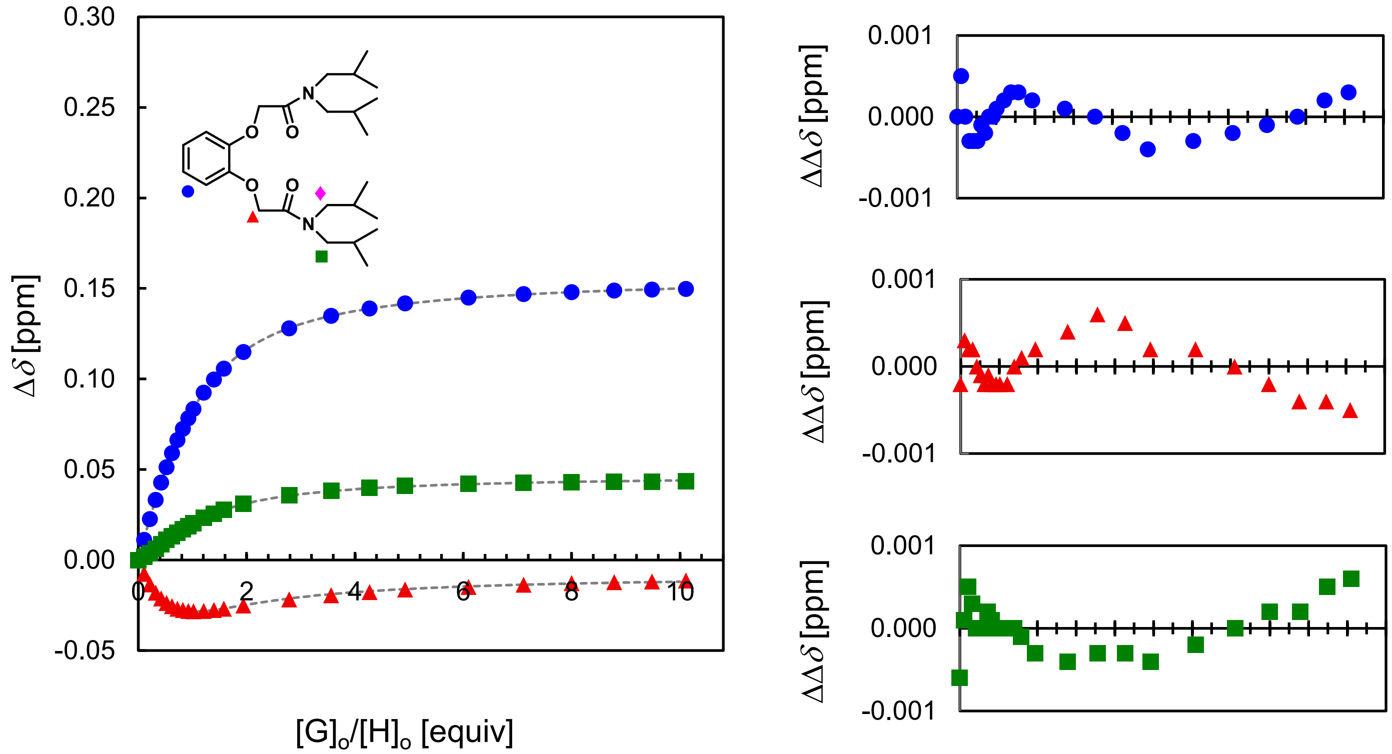

Figure S49. Experimental ^{1}H NMR chemical shift changes (symbols) and fitted binding isotherms (gray dashed lines) for titration of **1e** with KOTf in 99.5% CD_3CN + 0.5% H_2O at 298 K (left), assuming 2:1 (host:guest) binding model; residual distribution for the corresponding shift (right).

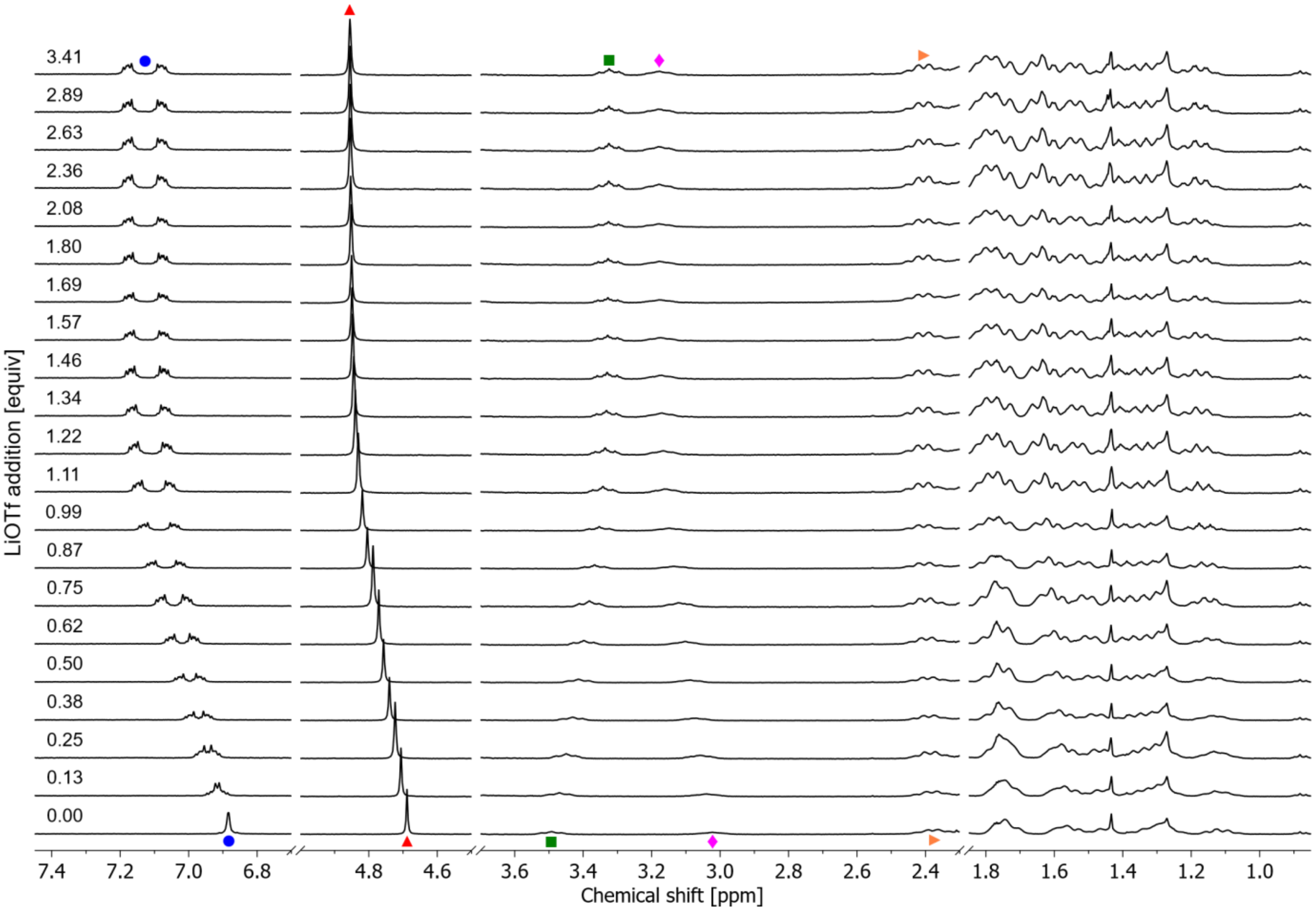

Figure S50. Stacked spectra from the ^{1}H NMR titration of **1f** with increasing amount of LiOTf.

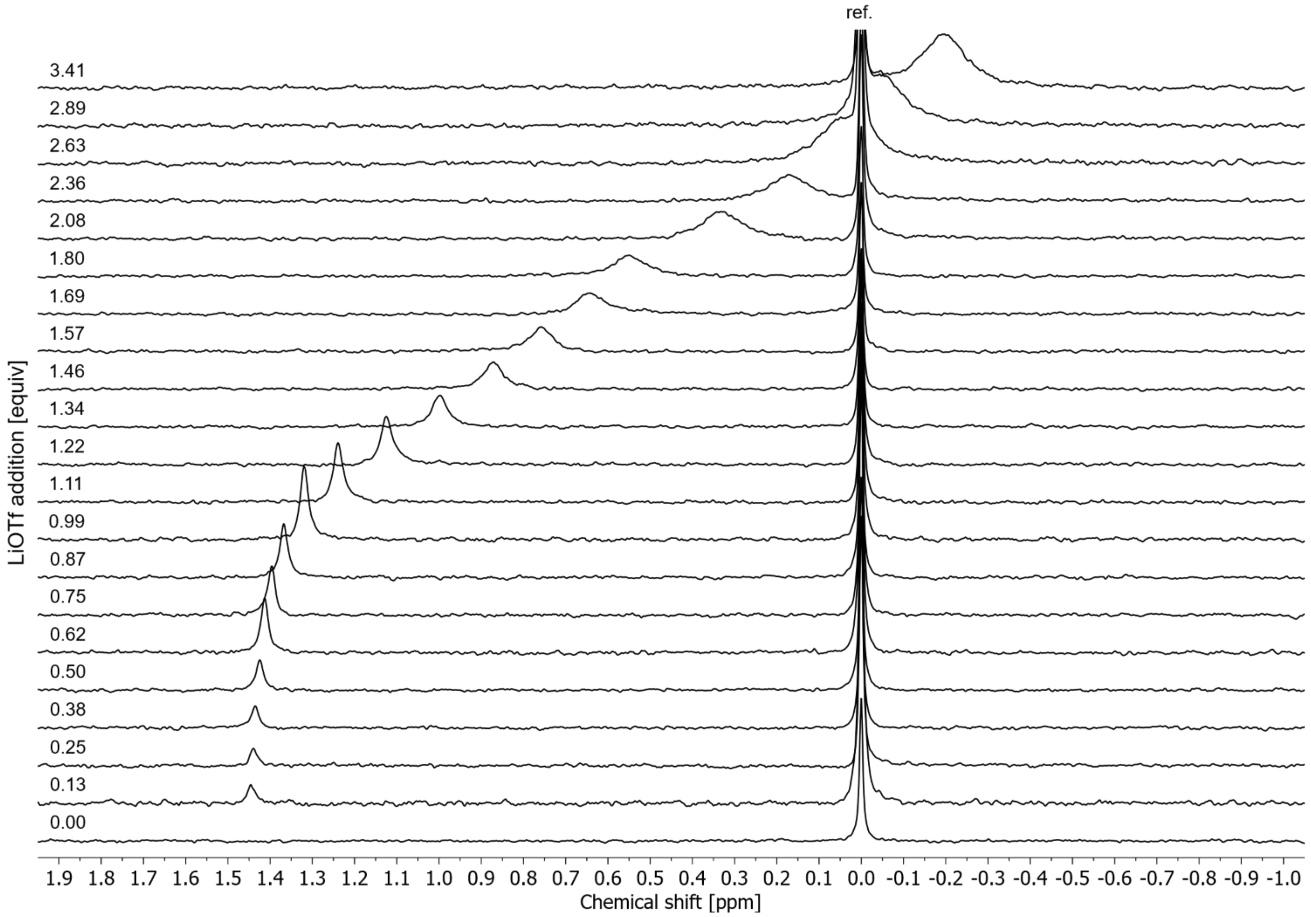

Figure S51. Stacked spectra from the ^{7}Li NMR titration of **1f** with increasing amount of LiOTf.

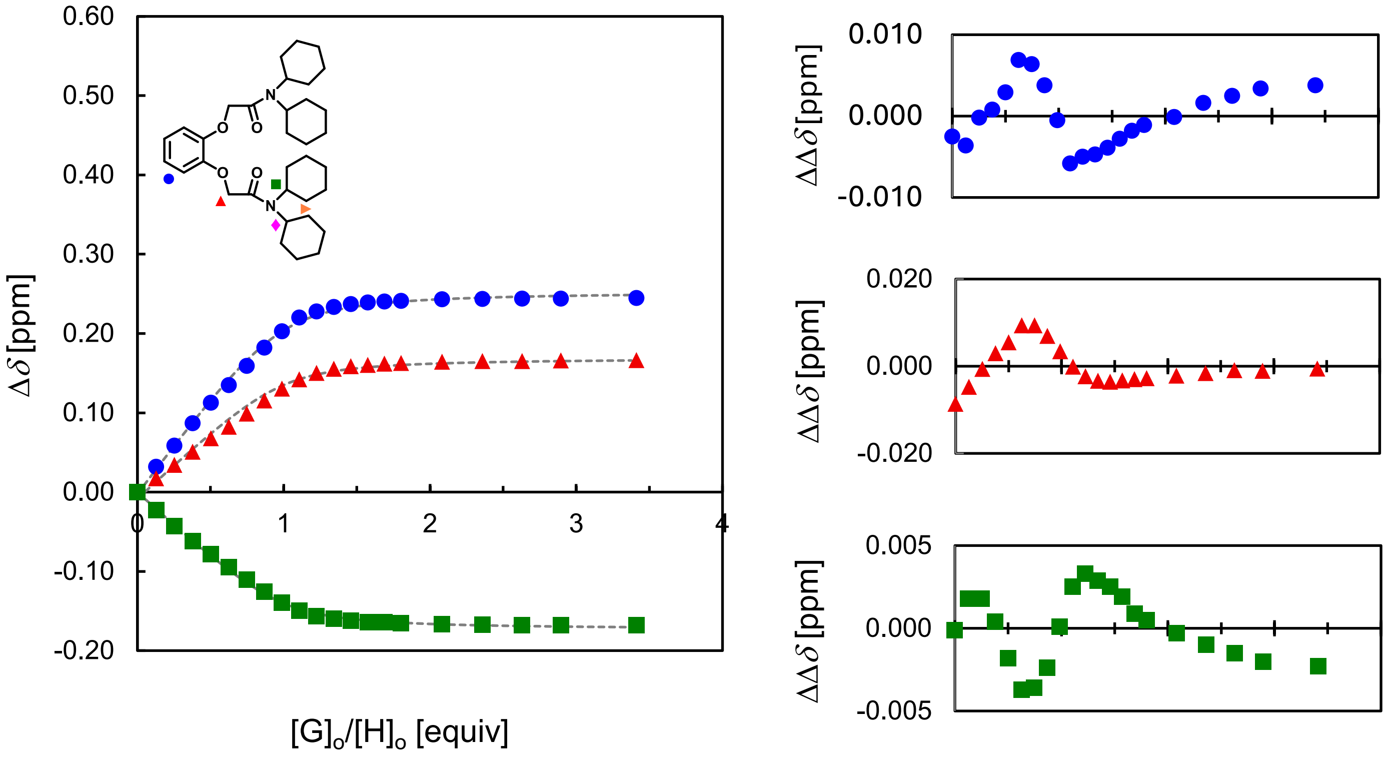

Figure S52. Experimental ^{1}H NMR chemical shift changes (symbols) and fitted binding isotherms (gray dashed lines) for titration of **1f** with LiOTf in 99.5% CD_3CN + 0.5% H_2O at 298 K (left), assuming 1:1 binding model; residual distribution for the corresponding shift (right).

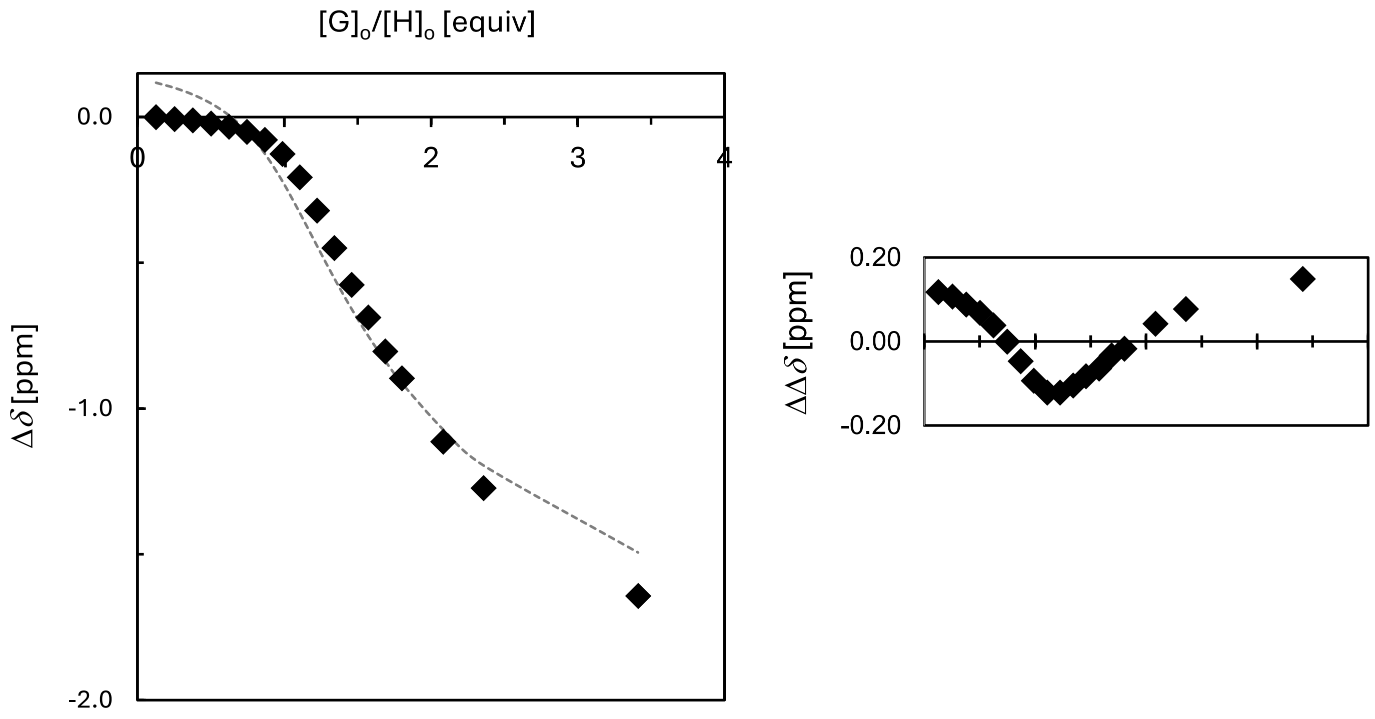

Figure S53. Experimental ^{7}Li NMR chemical shift changes (symbols) and calculated binding isotherms (gray dashed lines) for titration of **1f** with LiOTf in 99.5% CD_3CN + 0.5% H_2O at 298 K (left), assuming 1:1 binding model; residual distribution for the corresponding shift (right).

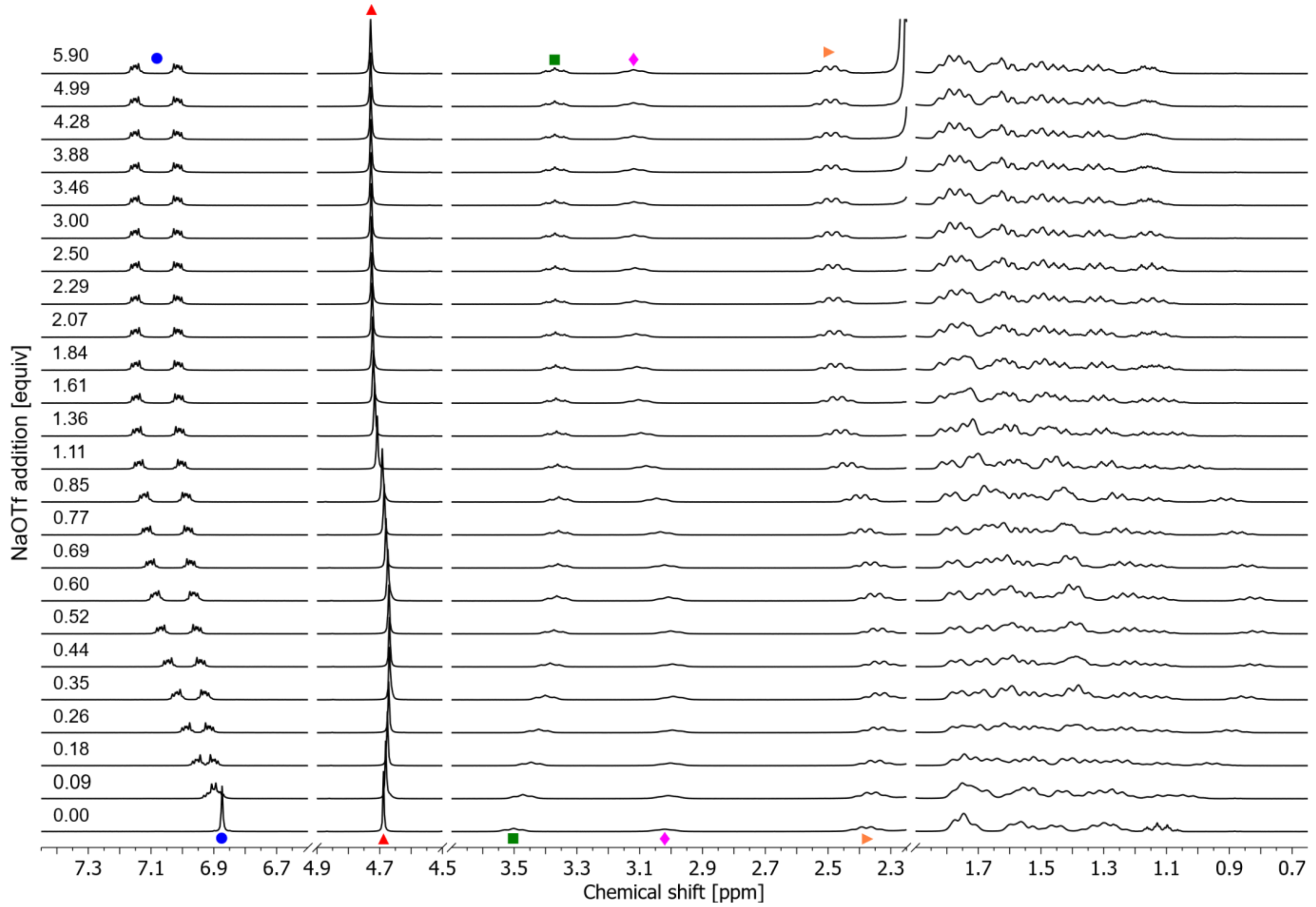

Figure S54. Stacked spectra from the ^{1}H NMR titration of **1f** with increasing amount of NaOTf.

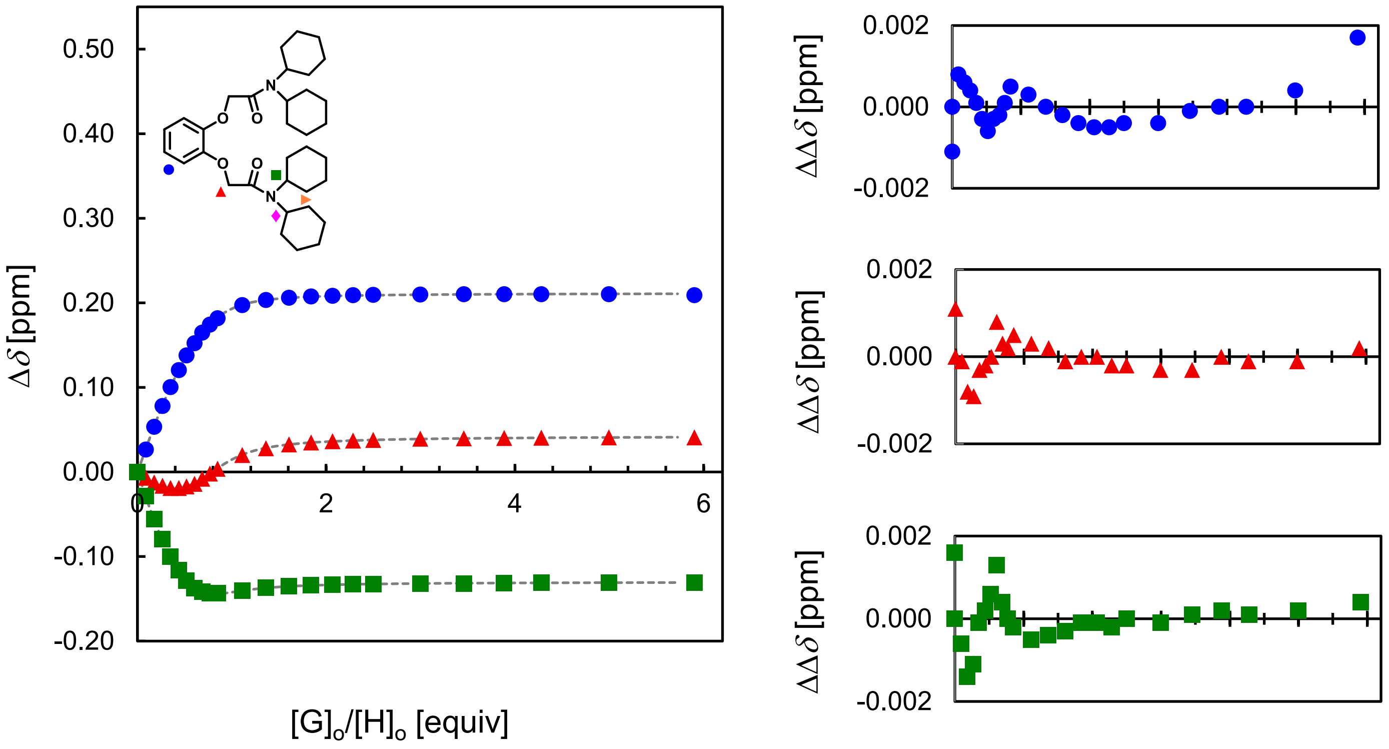

Figure S55. Experimental ^{1}H NMR chemical shift changes (symbols) and fitted binding isotherms (gray dashed lines) for titration of **1f** with NaOTf in 99.5% CD_3CN + 0.5% H_2O at 298 K (left), assuming 2:1 (host:guest) binding model; residual distribution for the corresponding shift (right).

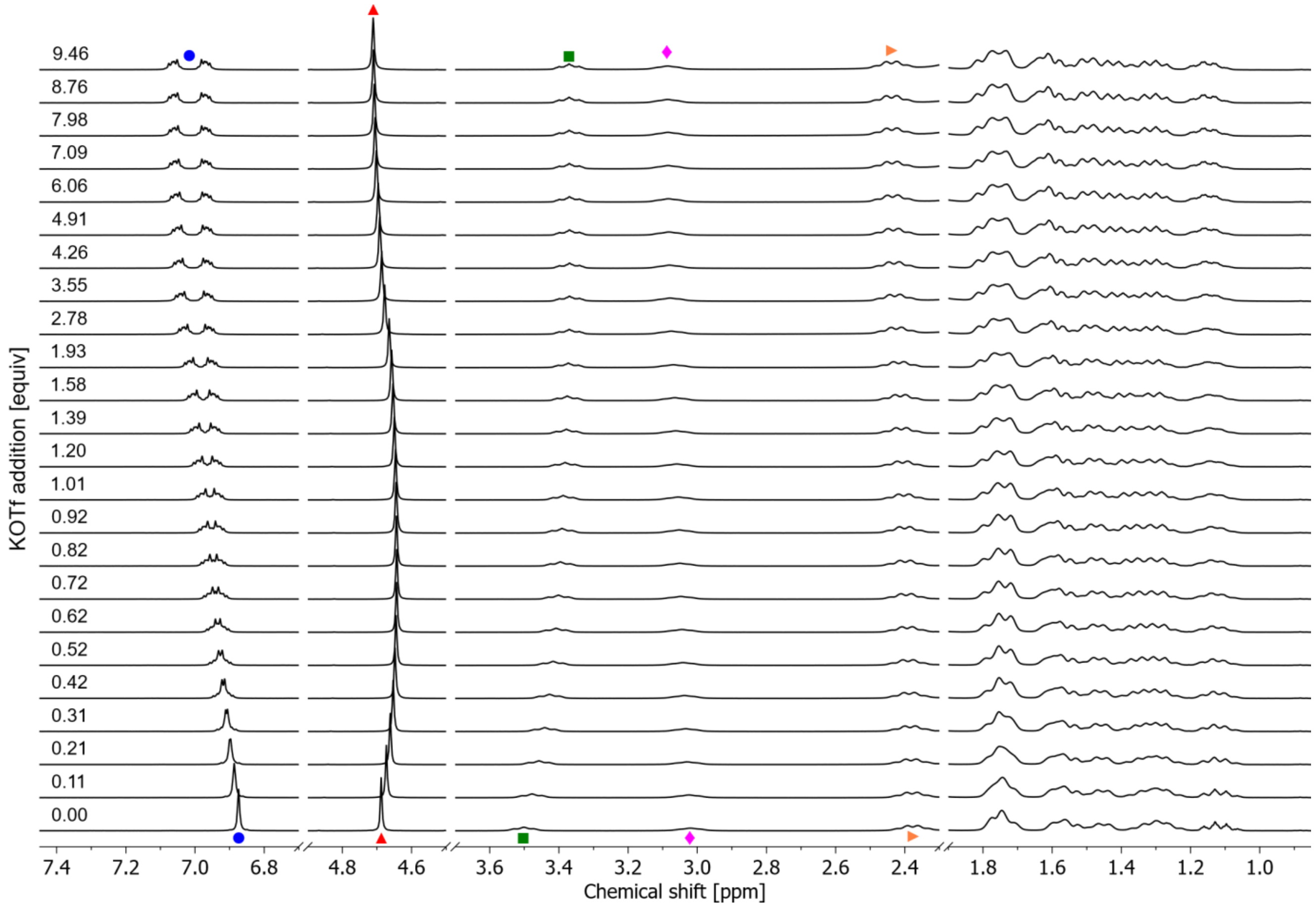

Figure S56. Stacked spectra from the ^{1}H NMR titration of **1f** with increasing amount of KOTf.

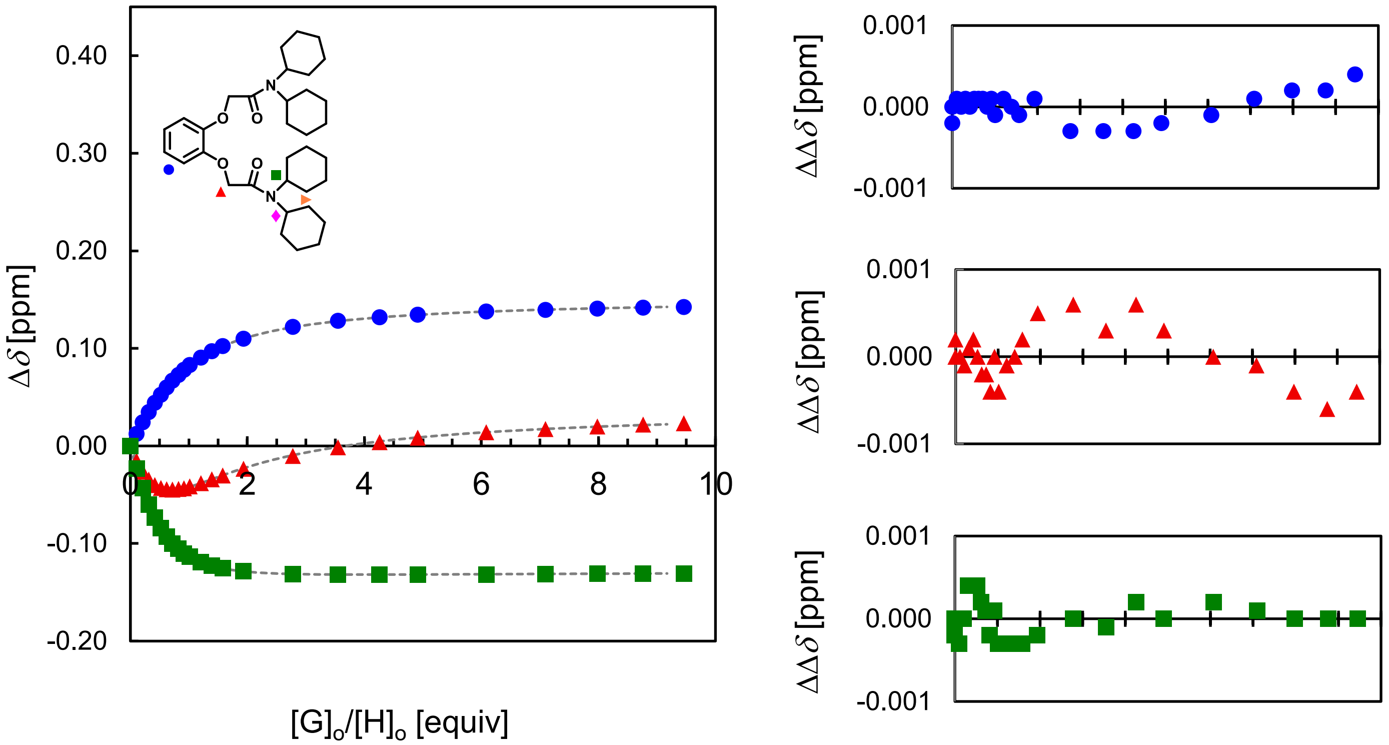

Figure S57. Experimental ^{1}H NMR chemical shift changes (symbols) and fitted binding isotherms (gray dashed lines) for titration of **1f** with KOTf in 99.5% CD_3CN + 0.5% H_2O at 298 K (left), assuming 2:1 (host:guest) binding model; residual distribution for the corresponding shift (right).

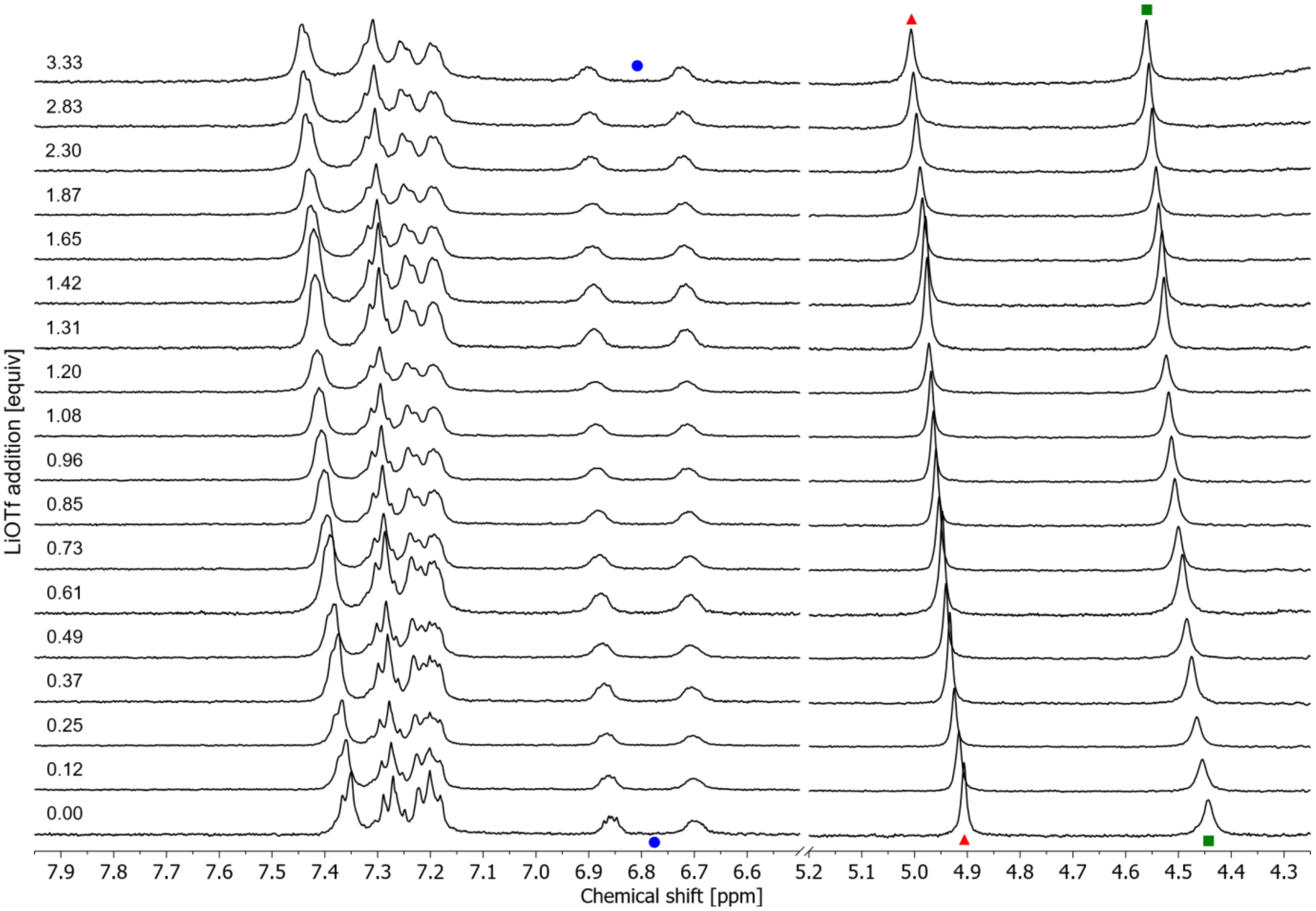

Figure S58. Stacked spectra from the ^{1}H NMR titration of **1g** with increasing amount of LiOTf.

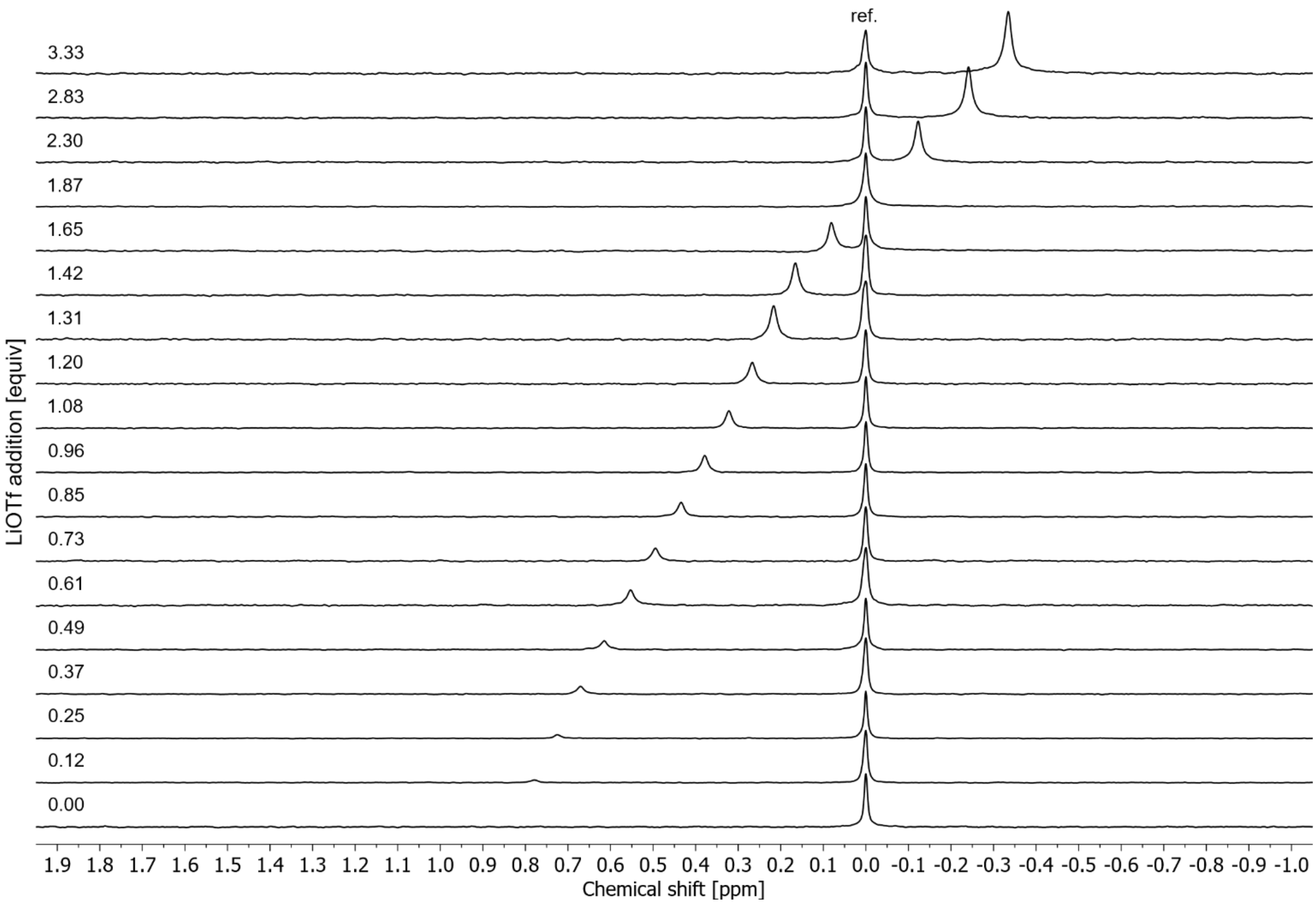

Figure S59. Stacked spectra from the ^{7}Li NMR titration of **1g** with increasing amount of LiOTf.

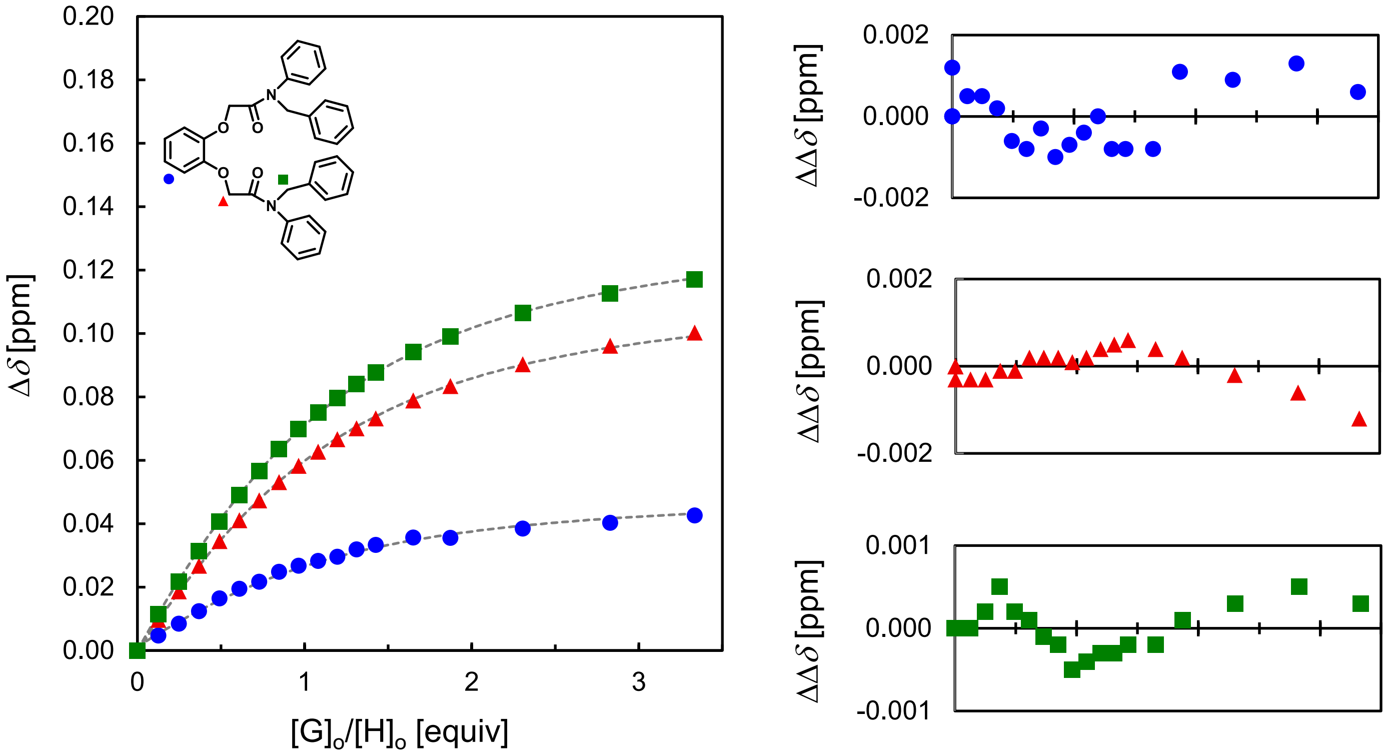

Figure S60. Experimental ^{1}H NMR chemical shift changes (symbols) and fitted binding isotherms (gray dashed lines) for titration of **1g** with LiOTf in 99.5% CD_3CN + 0.5% H_2O at 298 K (left), assuming 1:1 binding model; residual distribution for the corresponding shift (right).

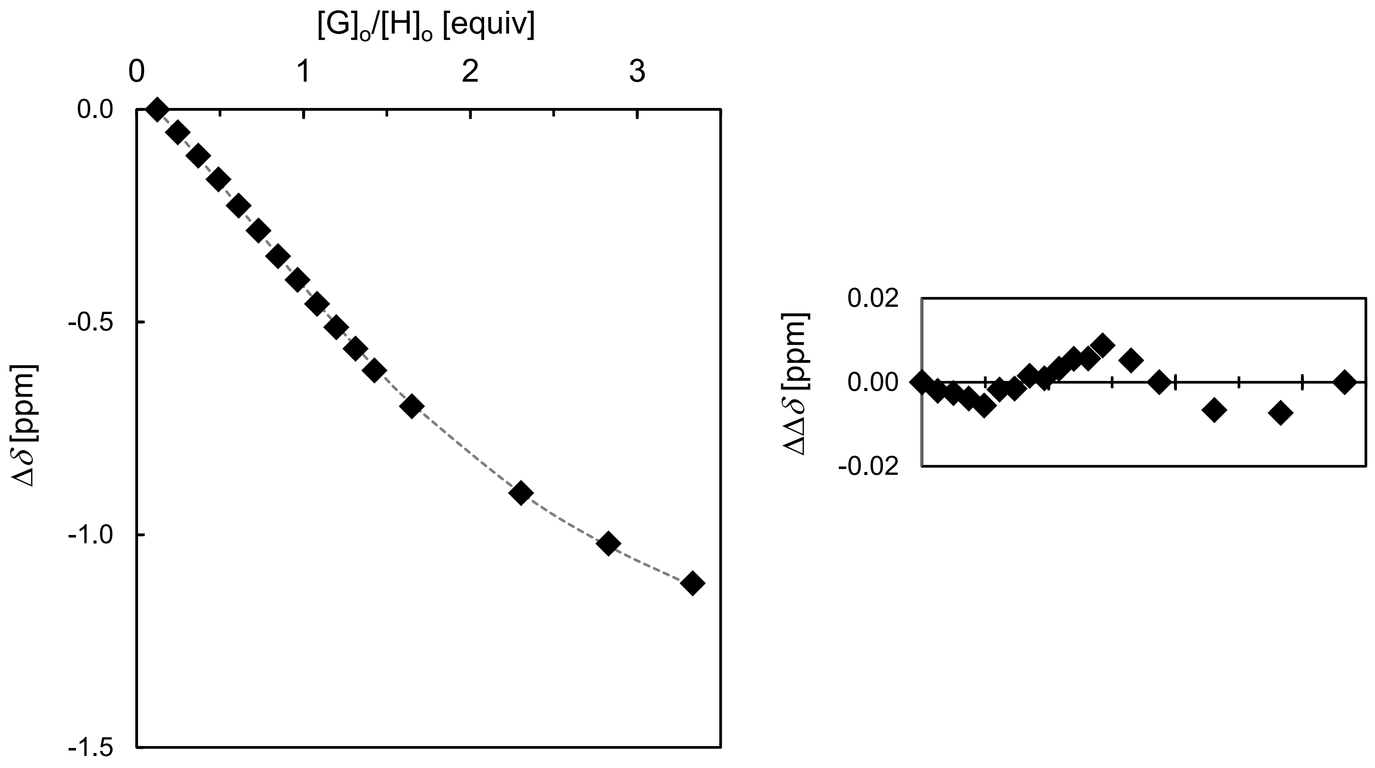

Figure S61. Experimental ^{7}Li NMR chemical shift changes (symbols) and calculated binding isotherms (gray dashed lines) for titration of **1g** with LiOTf in 99.5% CD_3CN + 0.5% H_2O at 298 K (left), assuming 1:1 binding model; residual distribution for the corresponding shift (right).

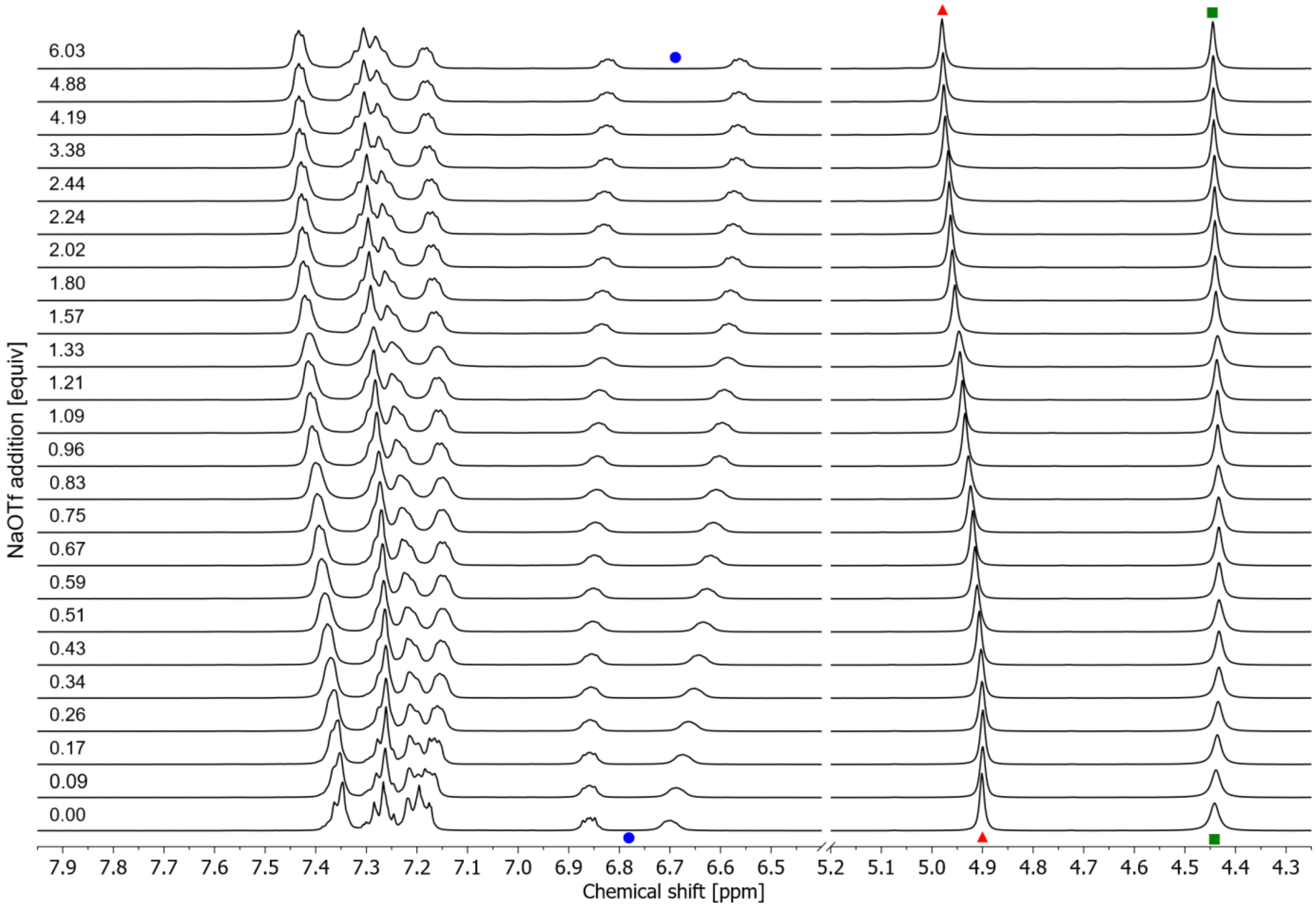

Figure S62. Stacked spectra from the ^{1}H NMR titration of **1g** with increasing amount of NaOTf.

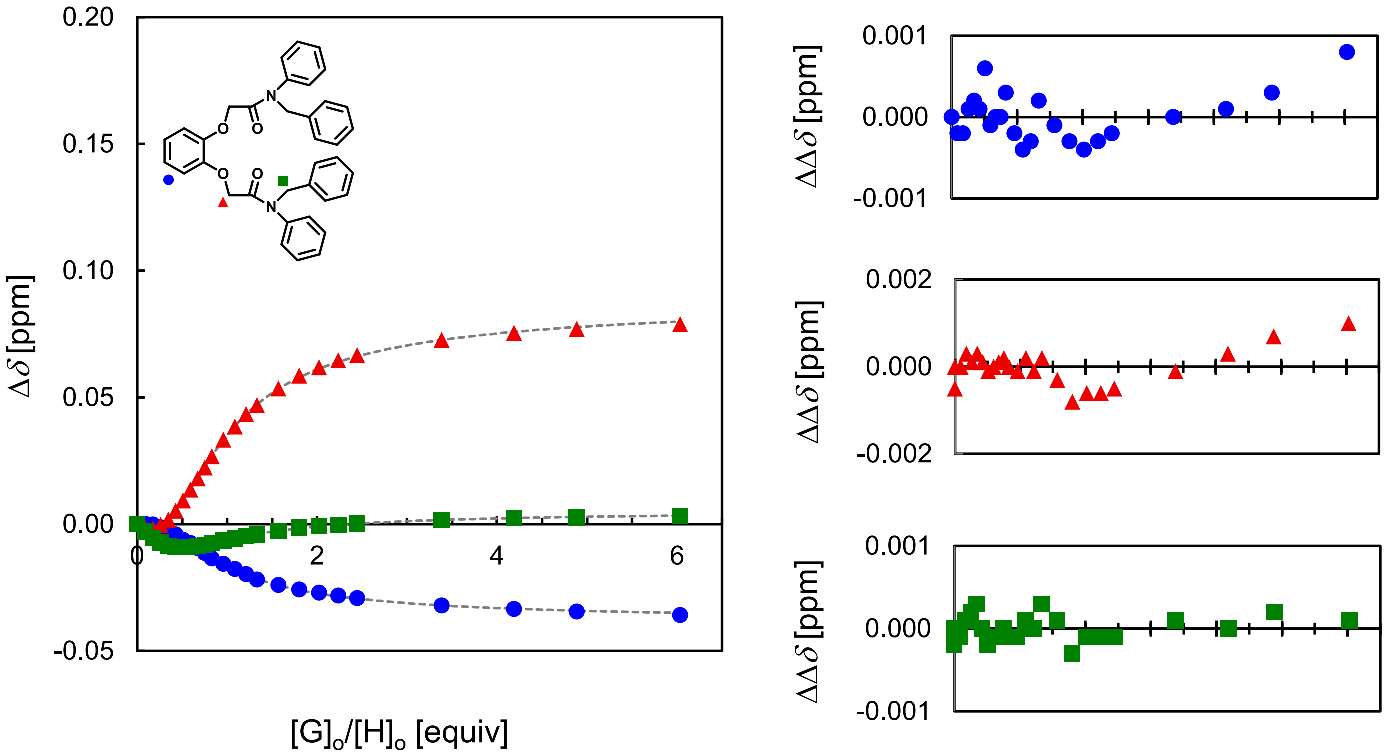

Figure S63. Experimental ^{1}H NMR chemical shift changes (symbols) and fitted binding isotherms (gray dashed lines) for titration of **1g** with NaOTf in 99.5% CD_3CN + 0.5% H_2O at 298 K (left), assuming 2:1 (host:guest) binding model; residual distribution for the corresponding shift (right).

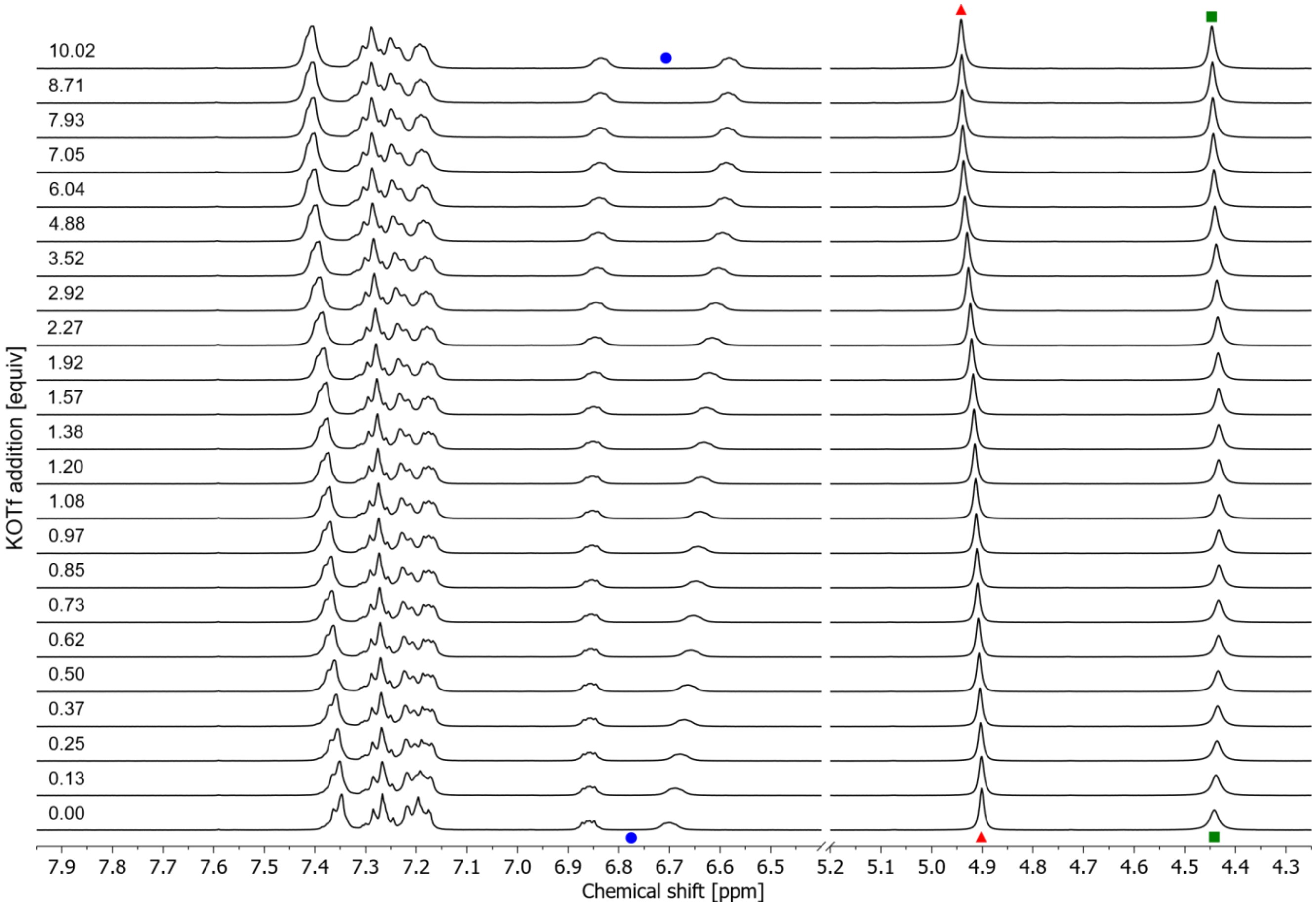

Figure S64. Stacked spectra from the ^{1}H NMR titration of **1g** with increasing amount of KOTf.

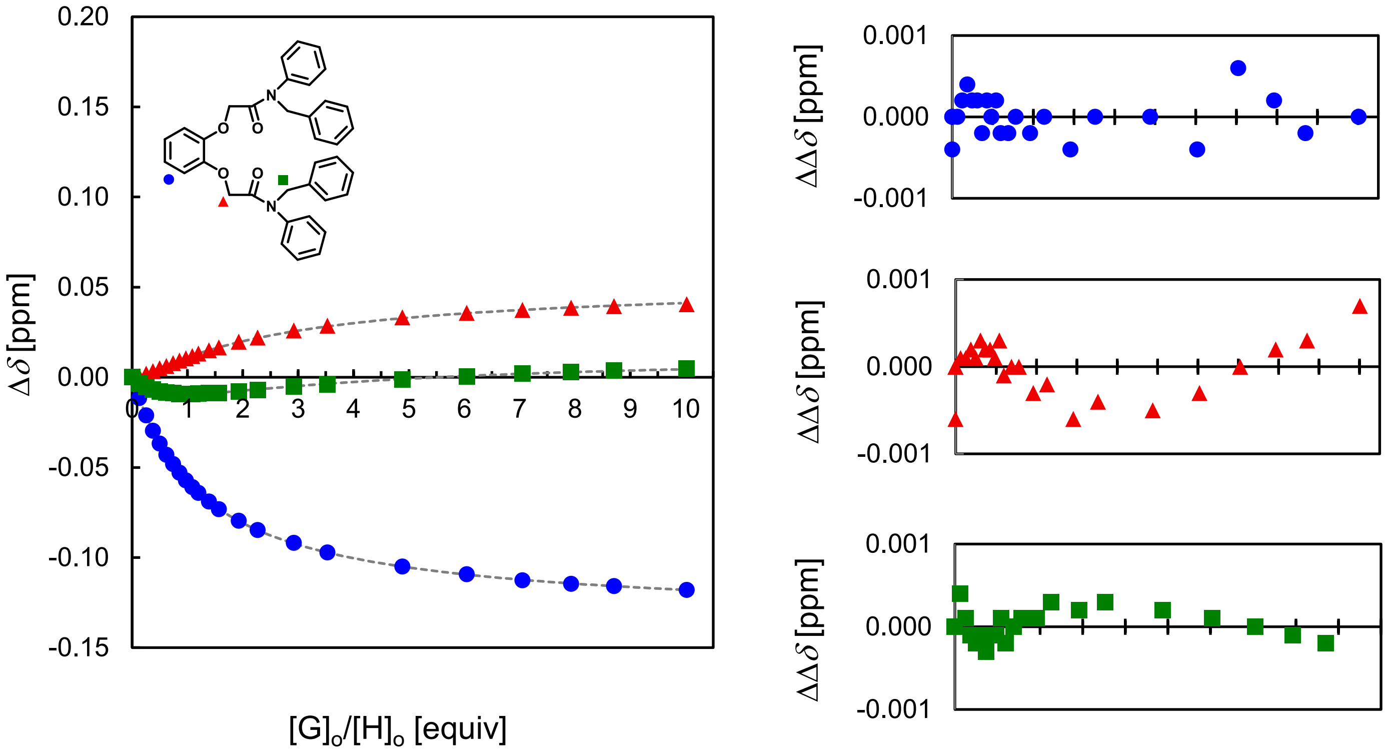

Figure S65. Experimental ^{1}H NMR chemical shift changes (symbols) and fitted binding isotherms (gray dashed lines) for titration of **1g** with KOTf in 99.5% CD_3CN + 0.5% H_2O at 298 K (left), assuming 2:1 (host:guest) binding model; residual distribution for the corresponding shift (right).

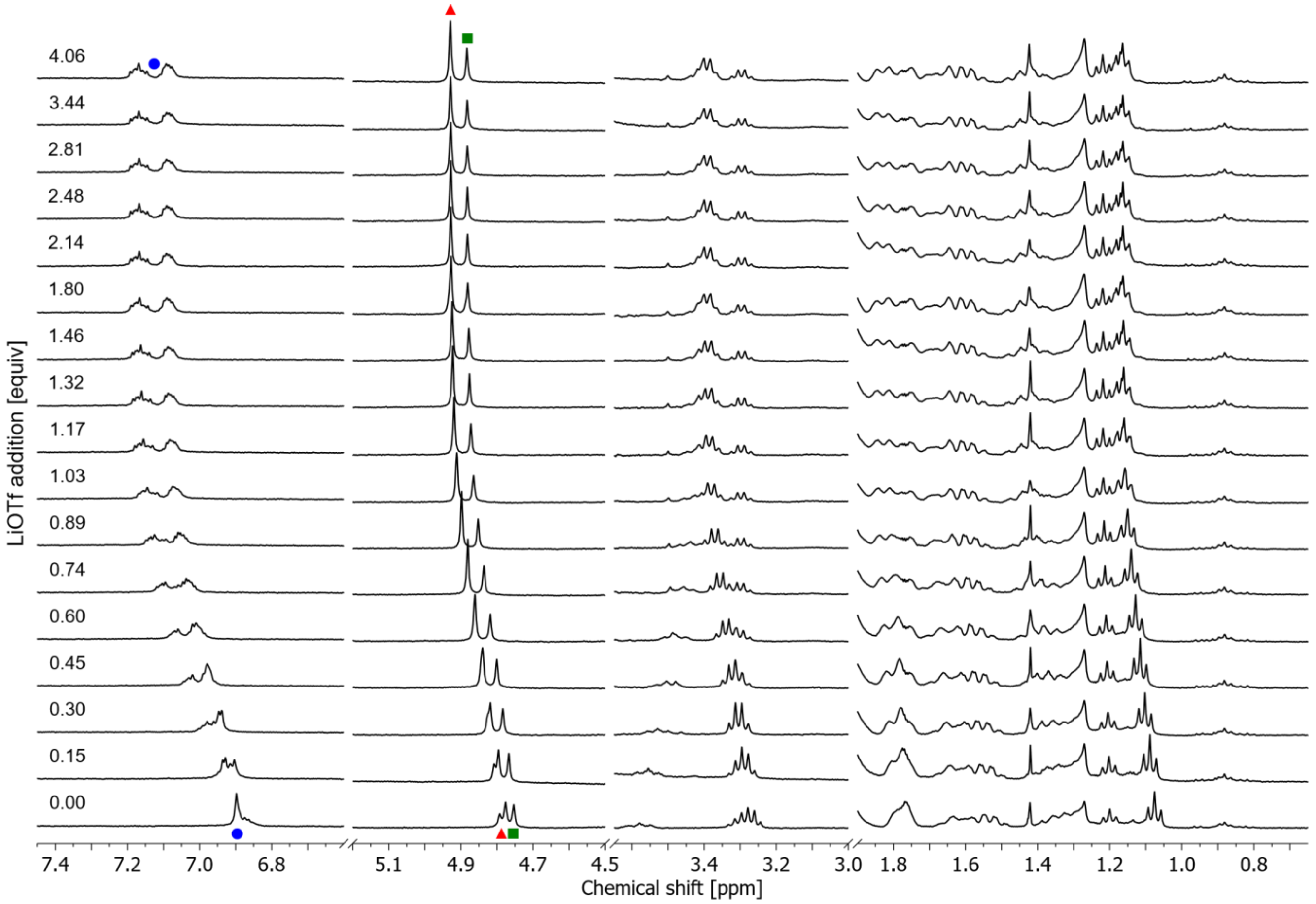

Figure S66. Stacked spectra from the ^{1}H NMR titration of **1h** with increasing amount of LiOTf.

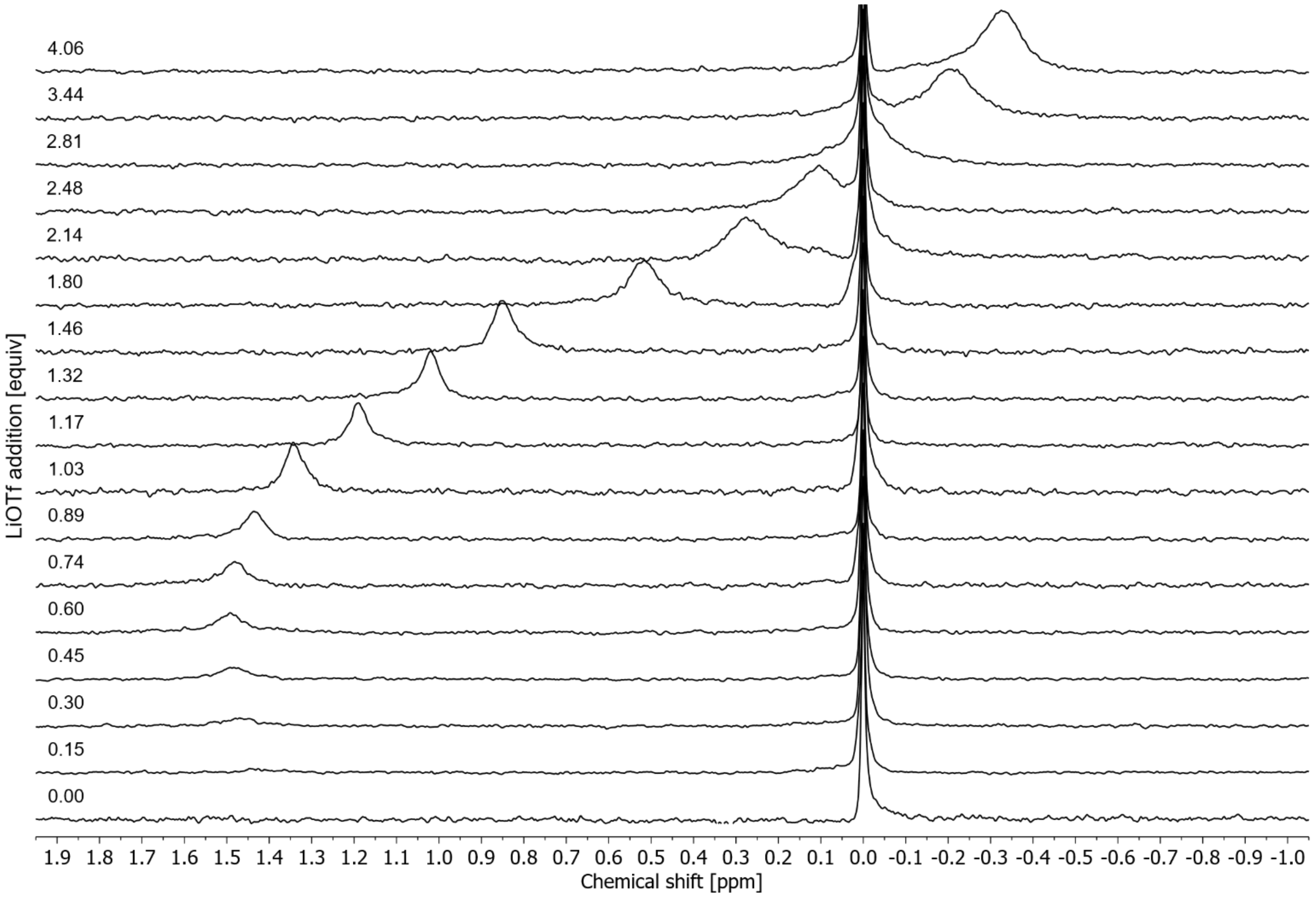

Figure S67. Stacked spectra from the ^{7}Li NMR titration of **1h** with increasing amount of LiOTf.

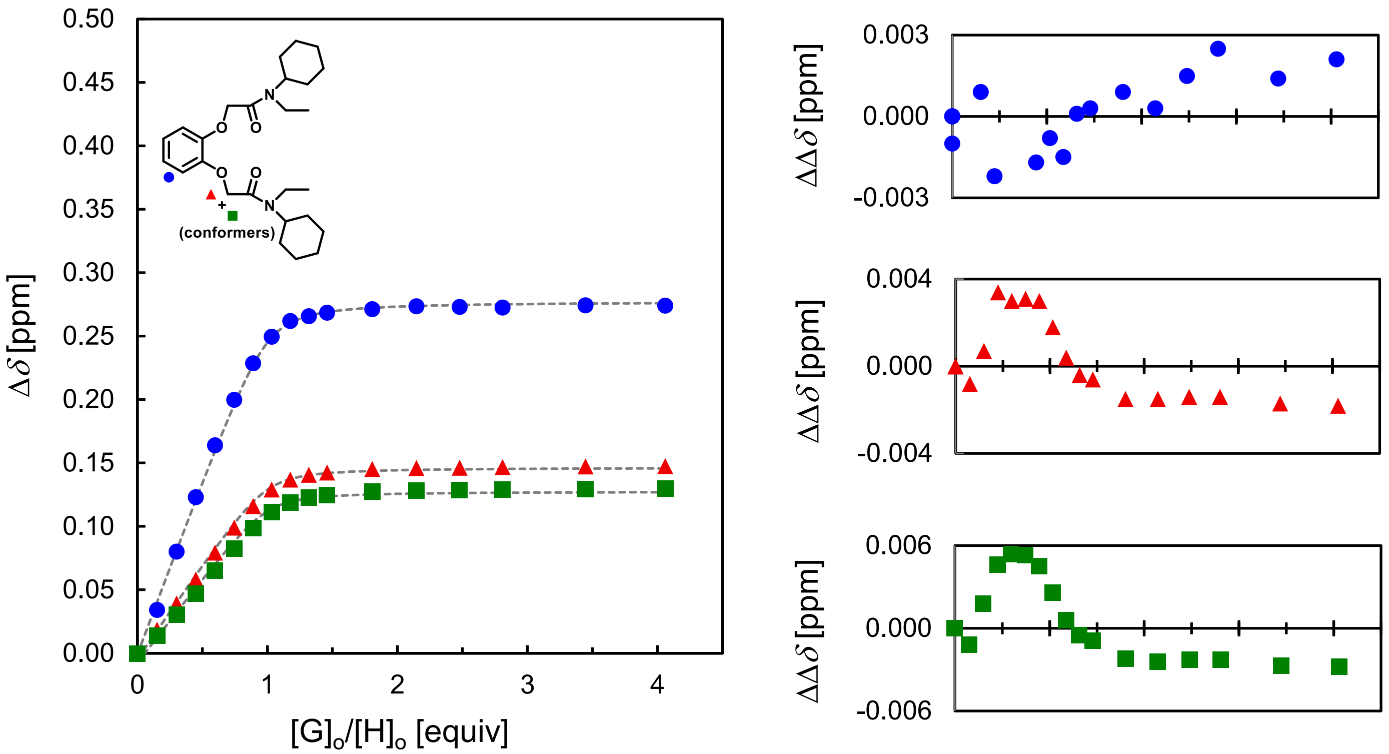

Figure S68. Experimental ^{1}H NMR chemical shift changes (symbols) and fitted binding isotherms (gray dashed lines) for titration of **1h** with LiOTf in 99.5% CD_3CN + 0.5% H_2O at 298 K (left), assuming 1:1 binding model; residual distribution for the corresponding shift (right).

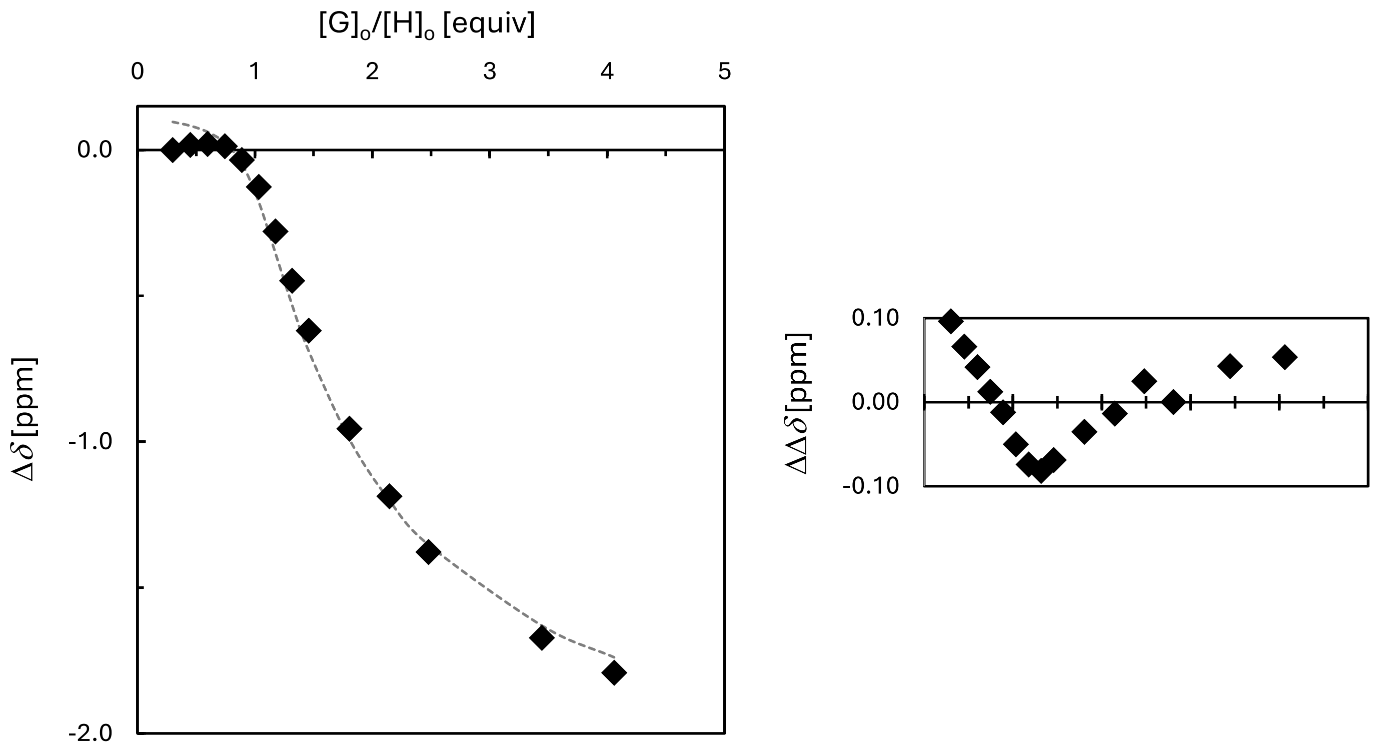

Figure S69. Experimental ^{7}Li NMR chemical shift changes (symbols) and calculated binding isotherms (gray dashed lines) for titration of **1h** with LiOTf in 99.5% CD_3CN + 0.5% H_2O at 298 K (left), assuming 1:1 binding model; residual distribution for the corresponding shift (right).

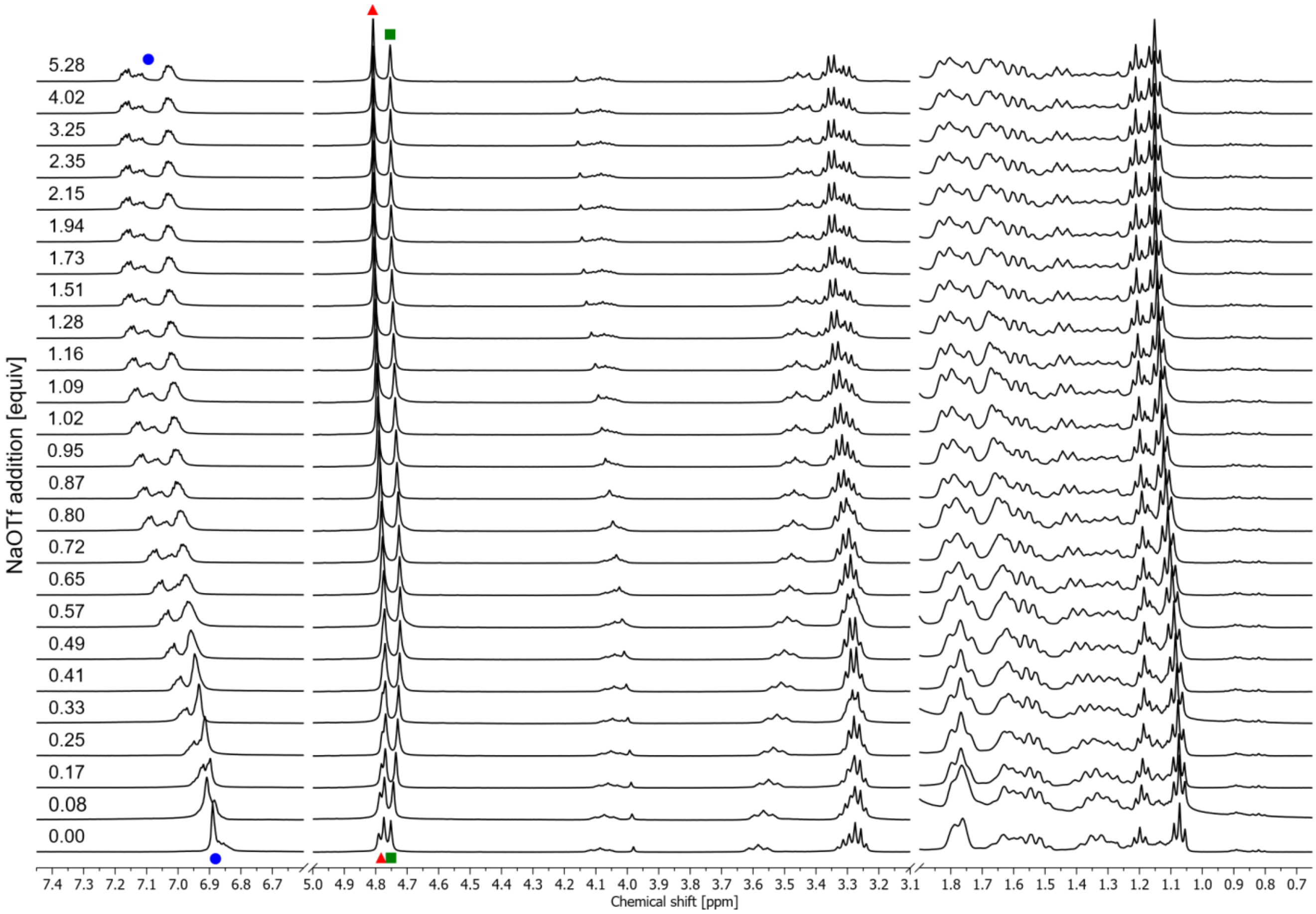

Figure S70. Stacked spectra from the ^{1}H NMR titration of **1h** with increasing amount of NaOTf.

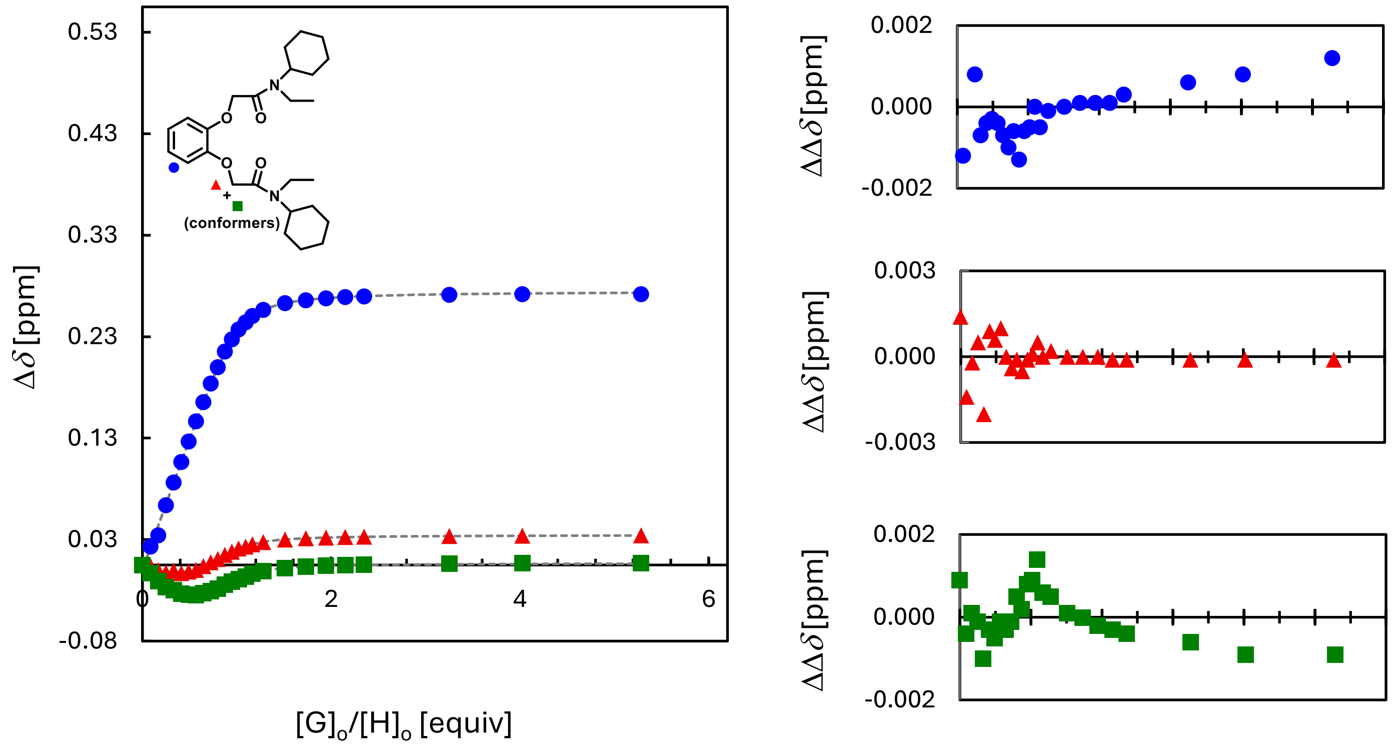

Figure S71. Experimental ^{1}H NMR chemical shift changes (symbols) and fitted binding isotherms (gray dashed lines) for titration of **1h** with NaOTf in 99.5% CD_3CN + 0.5% H_2O at 298 K (left), assuming 2:1 binding model; residual distribution for the corresponding shift (right).

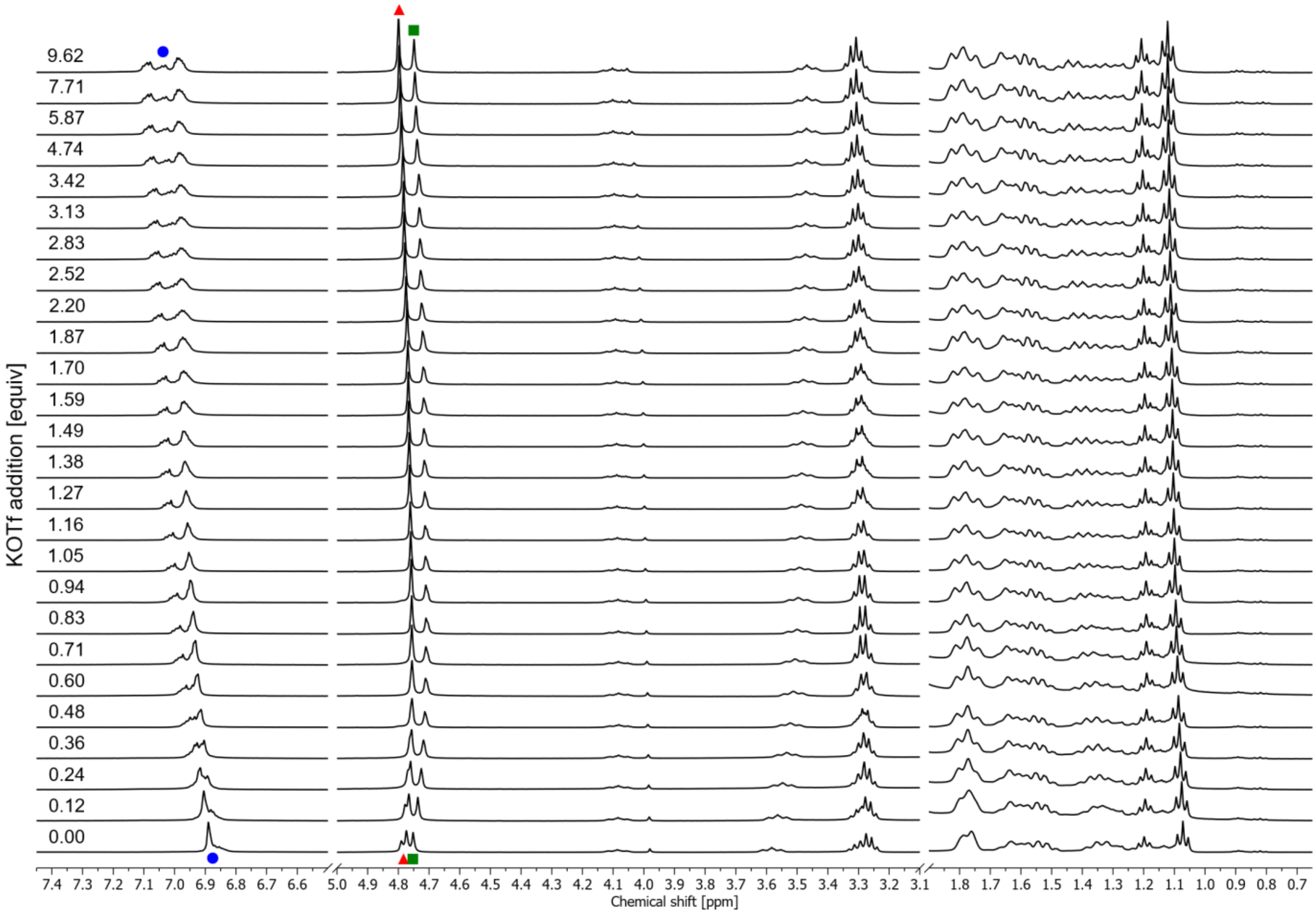

Figure S72. Stacked spectra from the ^{1}H NMR titration of **1h** with increasing amount of KOTf.

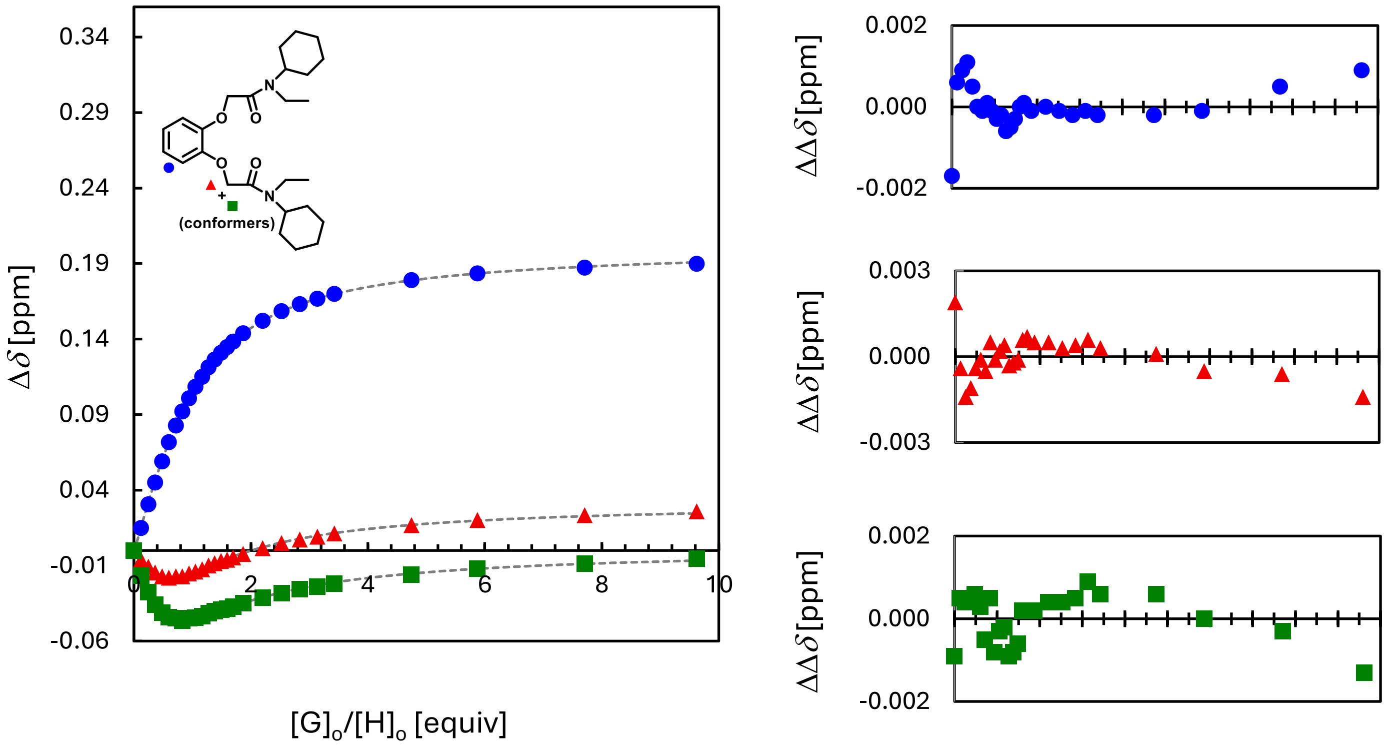

Figure S73. Experimental ^{1}H NMR chemical shift changes (symbols) and fitted binding isotherms (gray dashed lines) for titration of **1h** with KOTf in 99.5% CD_3CN + 0.5% H_2O at 298 K (left), assuming 2:1 binding model; residual distribution for the corresponding shift (right).

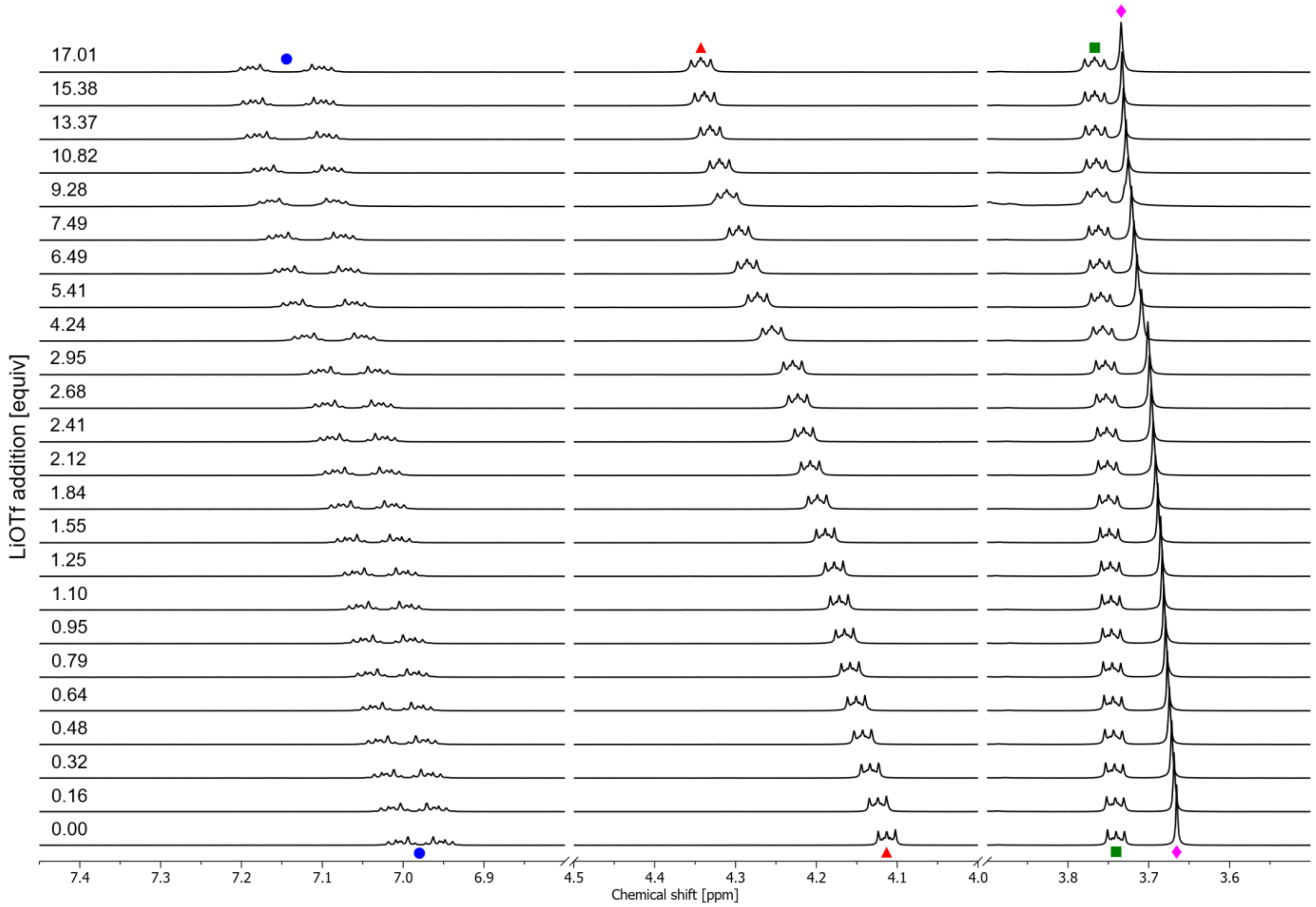

Figure S74. Stacked spectra from the ^{1}H NMR titration of **B12C4** with increasing amount of LiOTf.

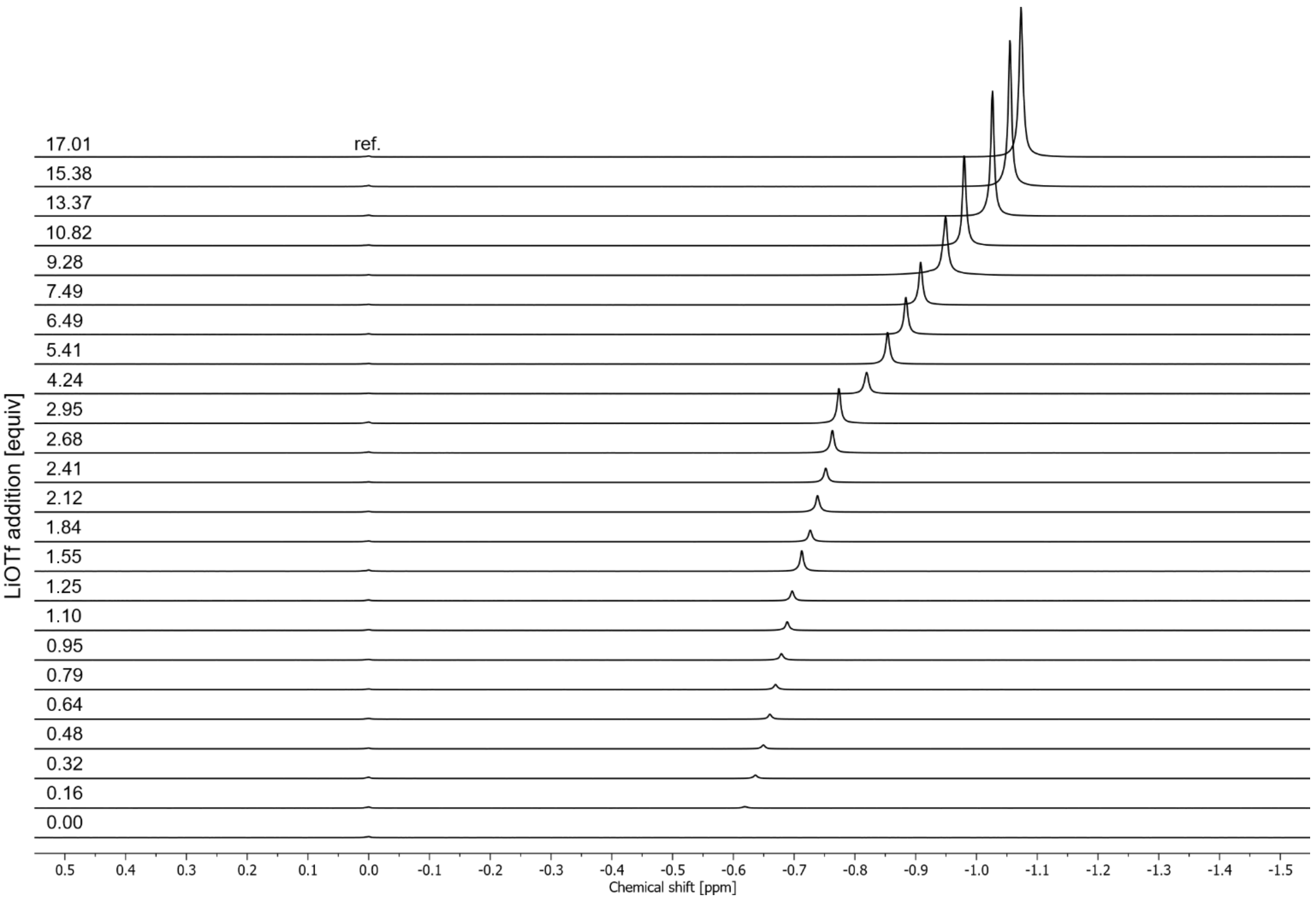

Figure S75. Stacked spectra from the ^{7}Li NMR titration of **B12C4** with increasing amount of LiOTf.

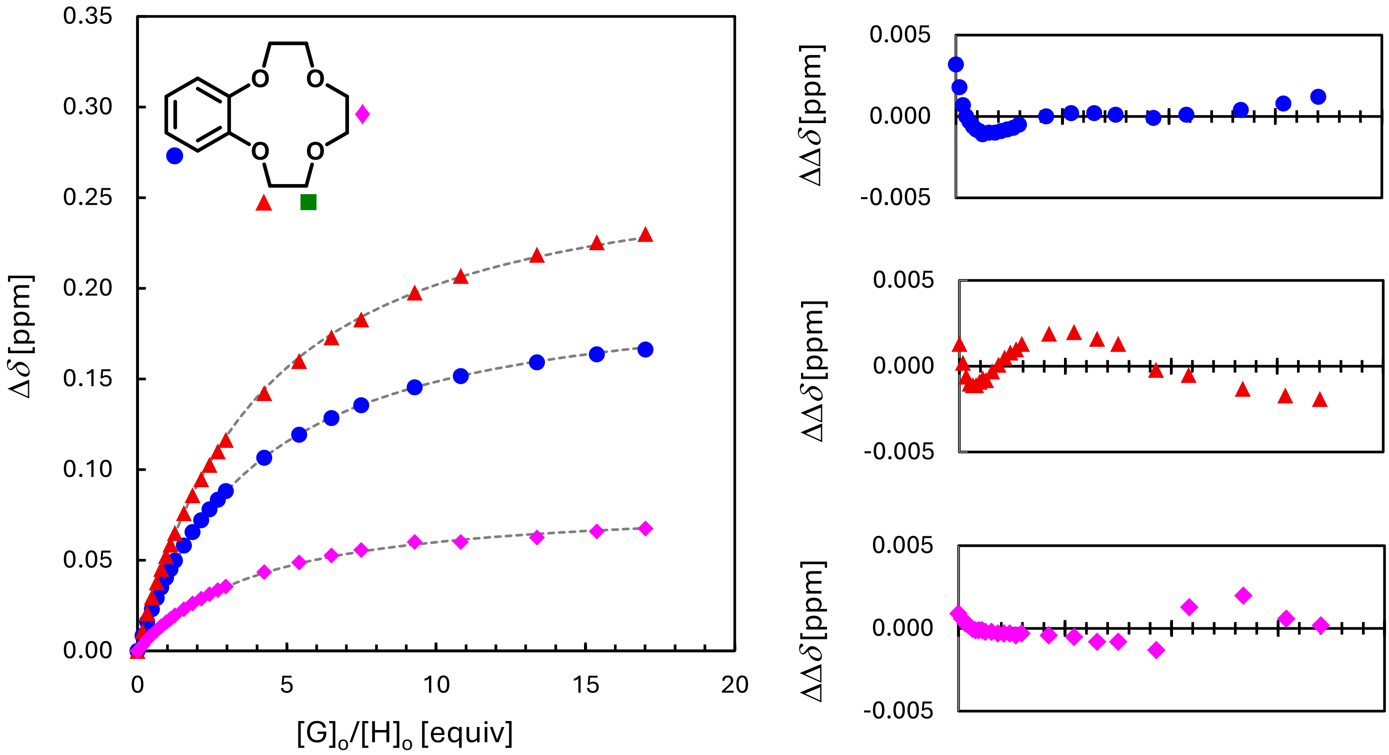

Figure S76. Experimental ^{1}H NMR chemical shift changes (symbols) and fitted binding isotherms (gray dashed lines) for titration of **B12C4** with LiOTf in 99.5% CD_3CN + 0.5% H_2O at 298 K (left), assuming 1:1 binding model; residual distribution for the corresponding shift (right).

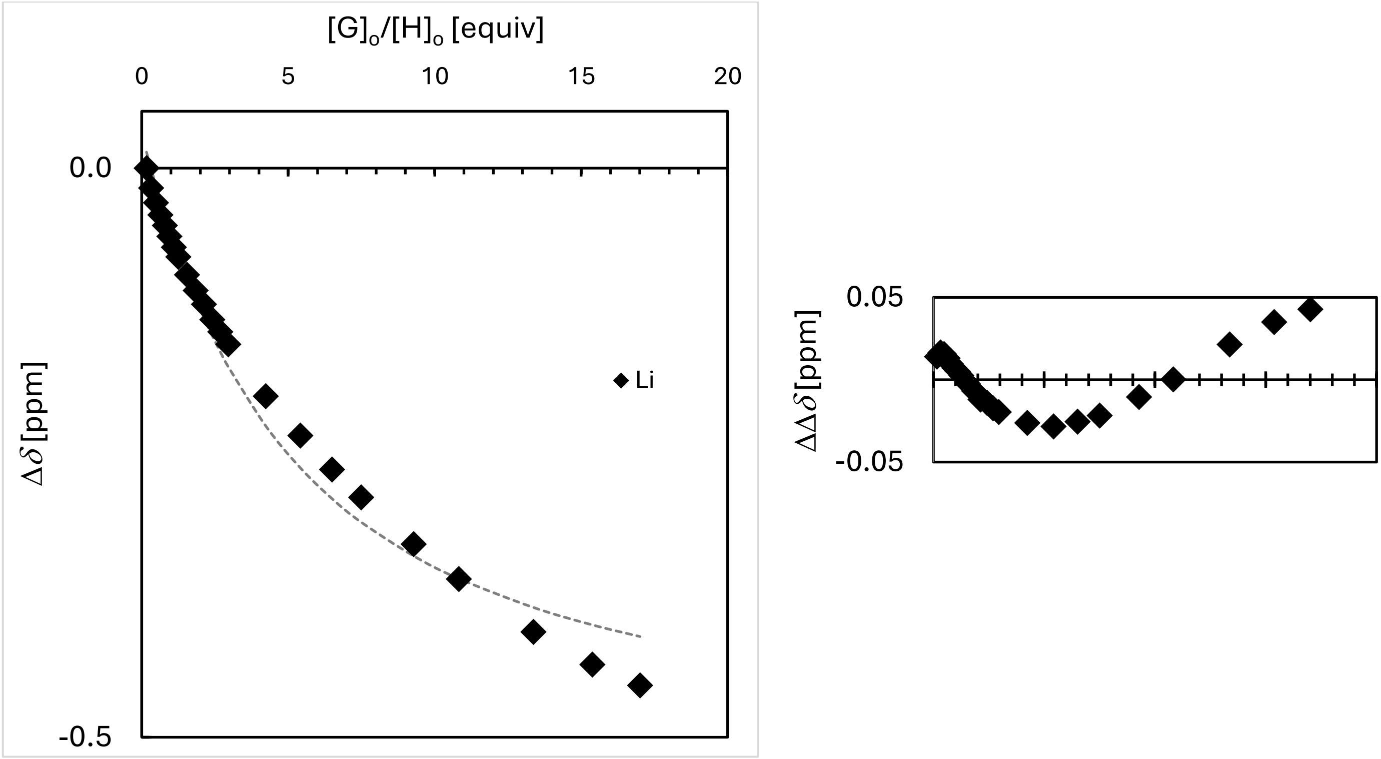

Figure S77. Experimental ^{7}Li NMR chemical shift changes (symbols) and calculated binding isotherms (gray dashed lines) for titration of **B12C4** with LiOTf in 99.5% CD_3CN + 0.5% H_2O at 298 K (left), assuming 1:1 binding model; residual distribution for the corresponding shift (right).

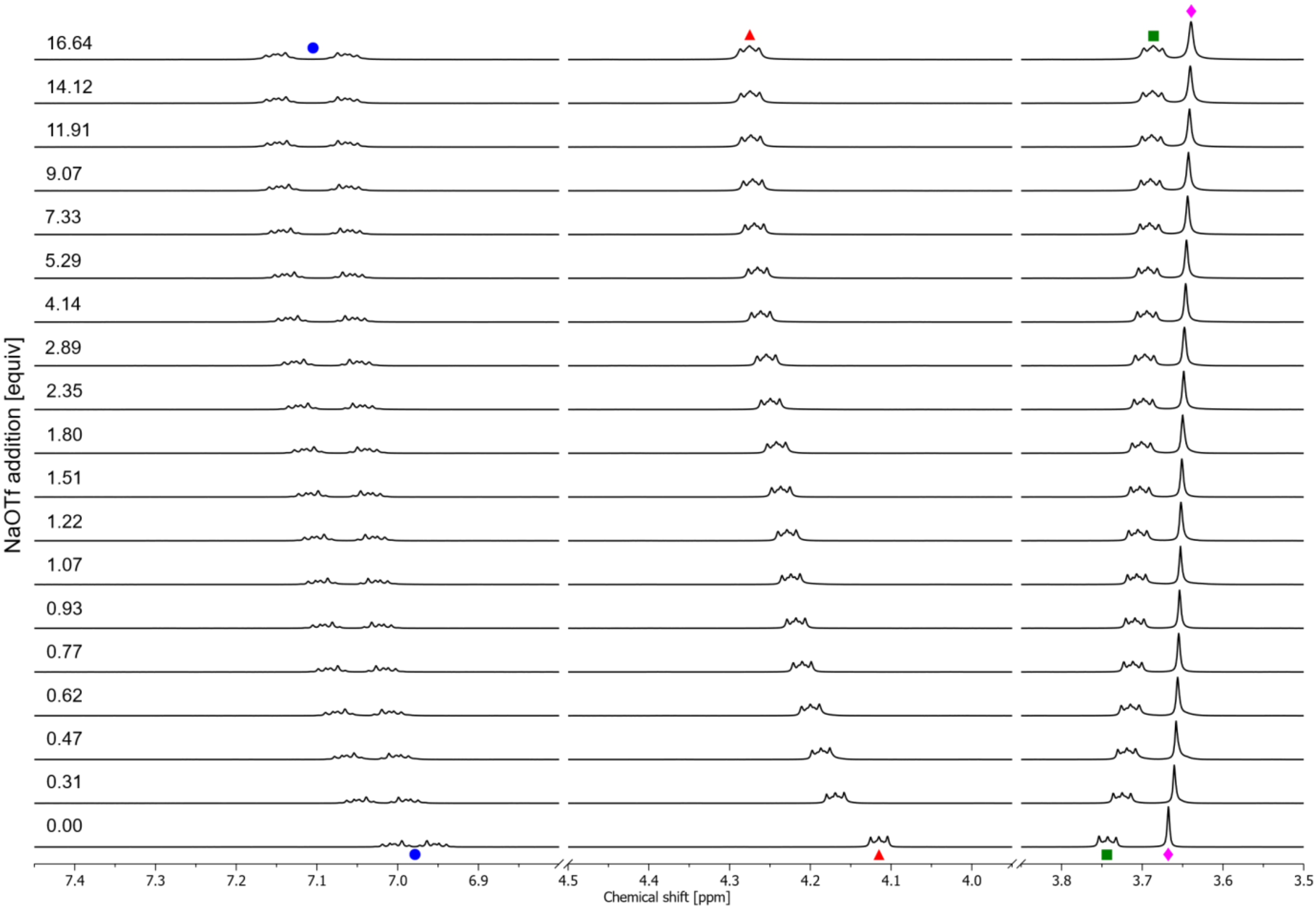

Figure S78. Stacked spectra from the ^{1}H NMR titration of **B12C4** with increasing amount of NaOTf.

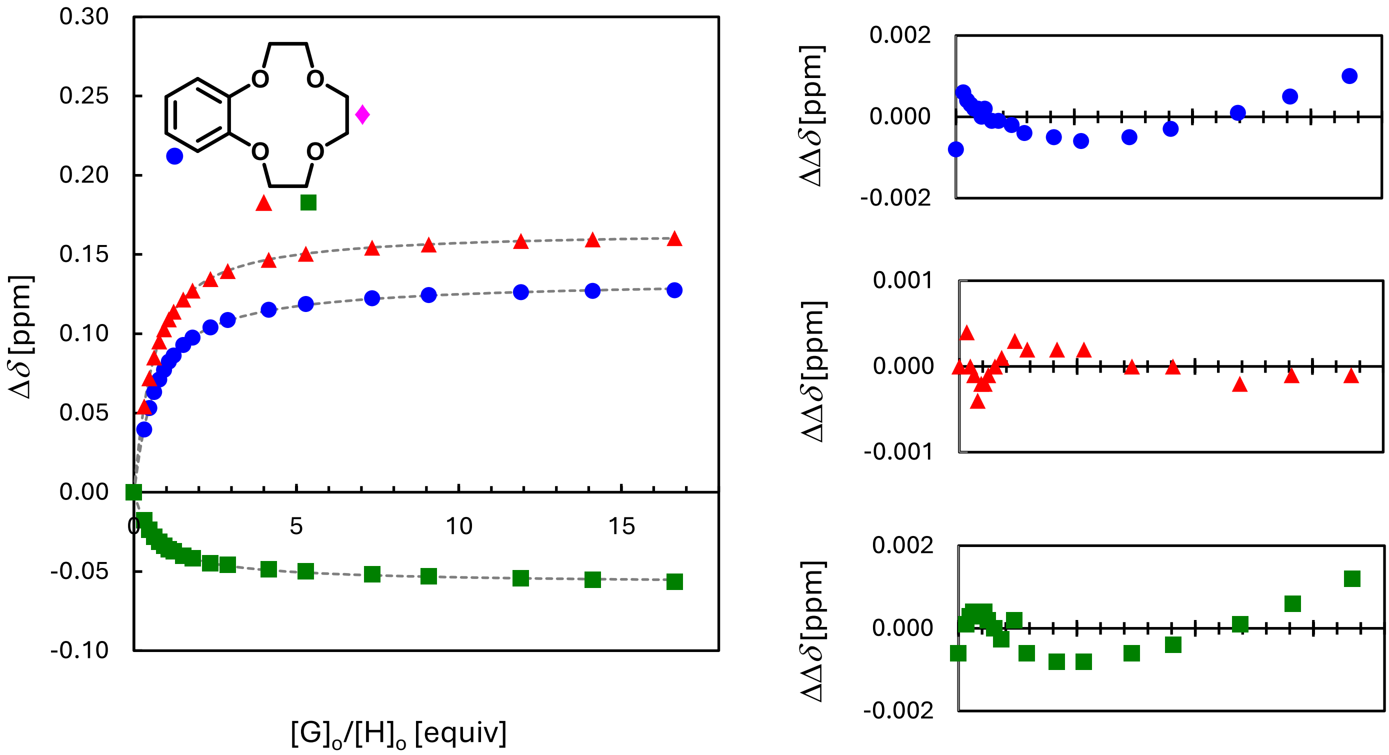

Figure S79. Experimental ^{1}H NMR chemical shift changes (symbols) and fitted binding isotherms (gray dashed lines) for titration of **B12C4** with NaOTf in 99.5% CD_3CN + 0.5% H_2O at 298 K (left), assuming 2:1 binding model; residual distribution for the corresponding shift (right).

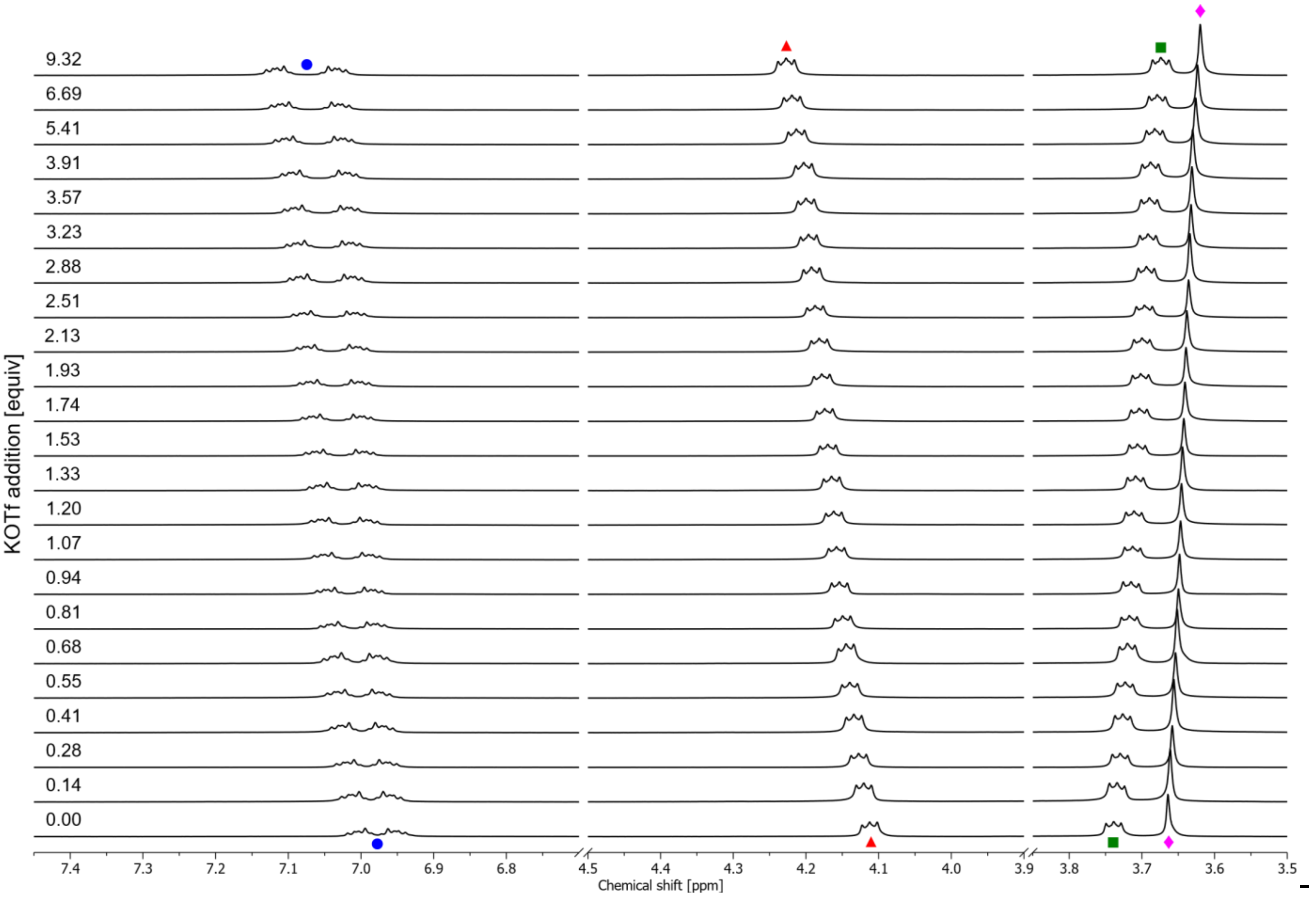

Figure S80. Stacked spectra from the ^{1}H NMR titration of **B12C4** with increasing amount of KOTf.

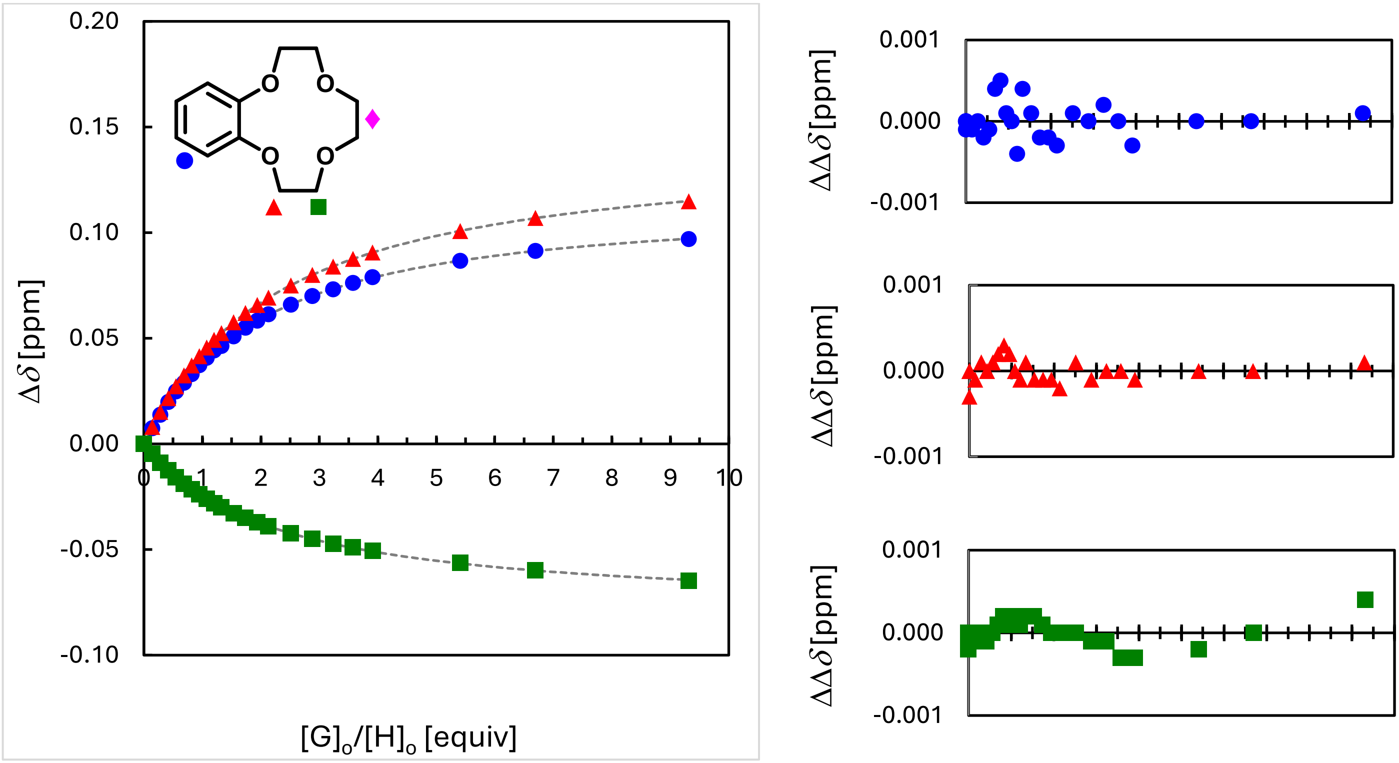

Figure S81. Experimental ^{1}H NMR chemical shift changes (symbols) and fitted binding isotherms (gray dashed lines) for titration of **B12C4** with KOTf in 99.5% CD_3CN + 0.5% H_2O at 298 K (left), assuming 2:1 binding model; residual distribution for the corresponding shift (right).

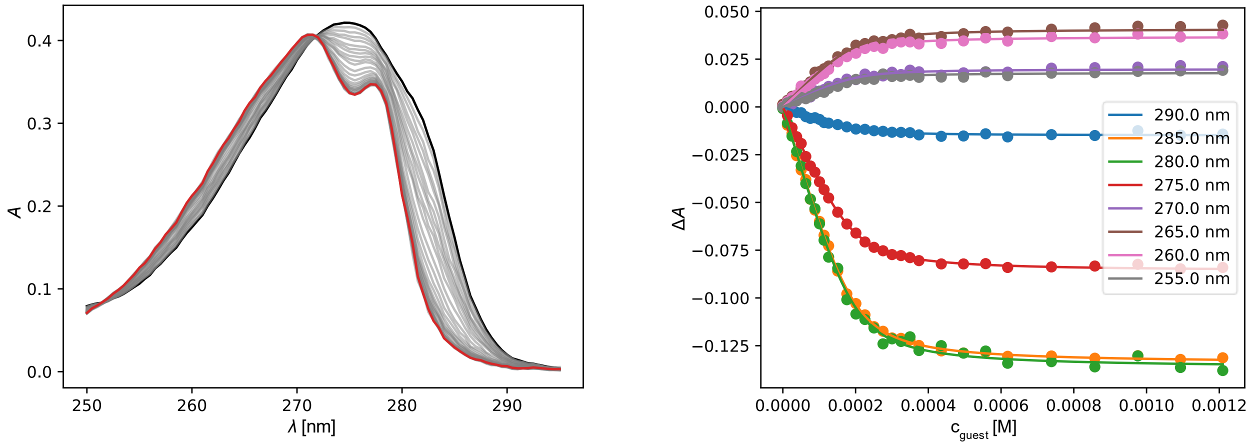

Figure S82. UV-Vis spectra recorded during UV-Vis titration of **1a** with LiOTf in 99.5% CH_3CN + 0.5% H_2O (black – first spectra, red – last spectra) (left); changes in absorbances (dots) at given wavelengths fitted to 1:1 model (solid lines) (right).

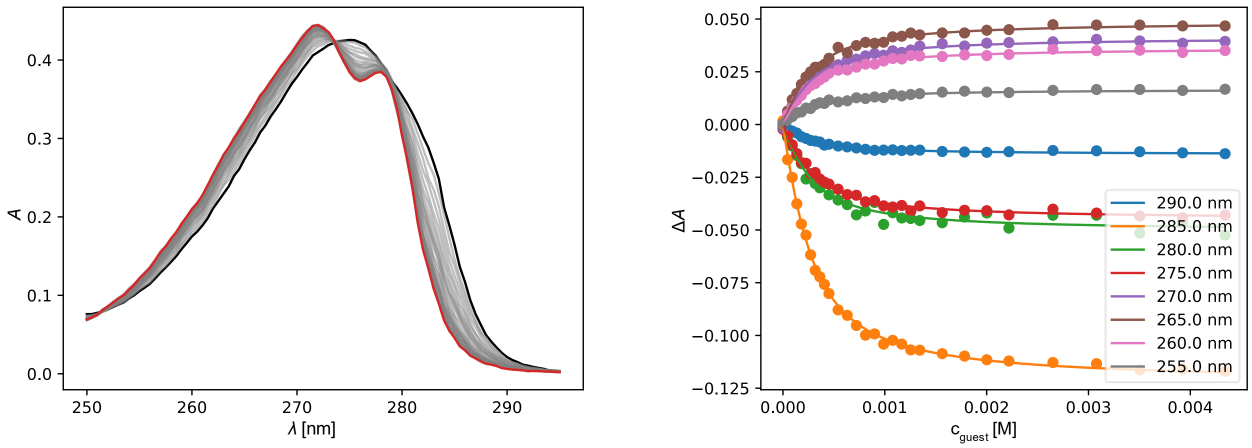

Figure S83. UV-Vis spectra recorded during UV-Vis titration of **1a** with NaOTf in 99.5% CH_3CN + 0.5% H_2O (black – first spectra, red – last spectra) (left); changes in absorbances (dots) at given wavelengths fitted to 1:1 model (solid lines) (right).

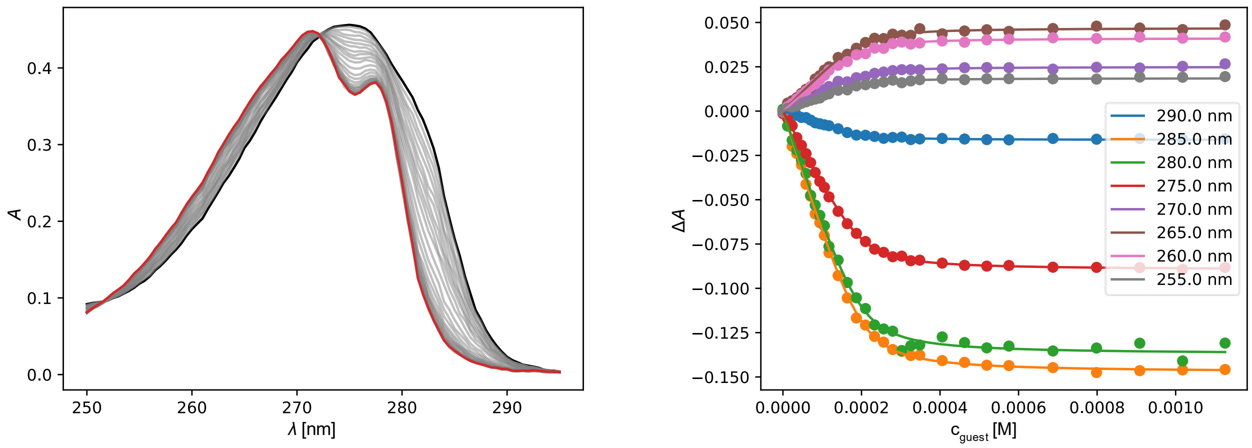

Figure S84. UV-Vis spectra recorded during UV-Vis titration of **1b** with LiOTf in 99.5% CH_3CN + 0.5% H_2O (black – first spectra, red – last spectra) (left); changes in absorbances (dots) at given wavelengths fitted to 1:1 model (solid lines) (right).

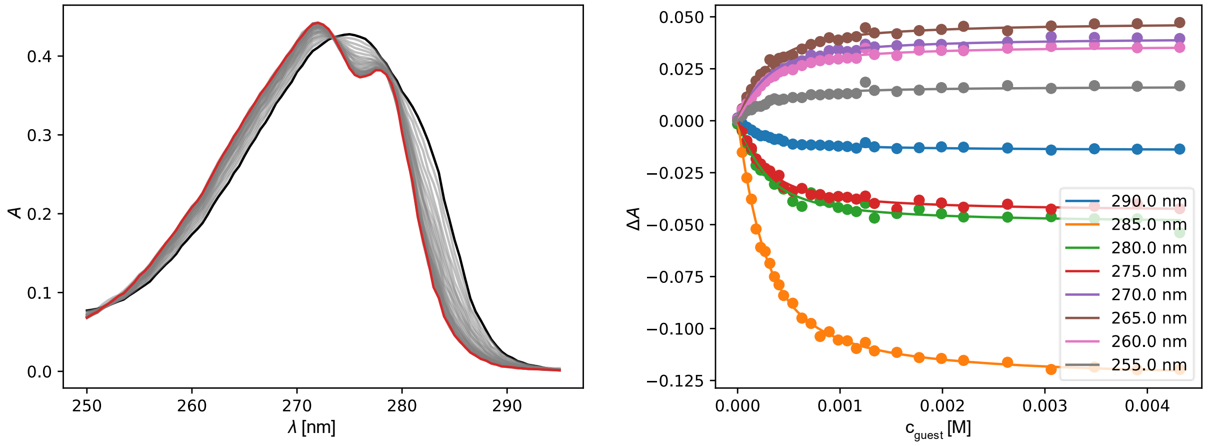

Figure S85. UV-Vis spectra recorded during UV-Vis titration of **1b** with NaOTf in 99.5% CH_3CN + 0.5% H_2O (black – first spectra, red – last spectra) (left); changes in absorbances (dots) at given wavelengths fitted to 1:1 model (solid lines) (right).

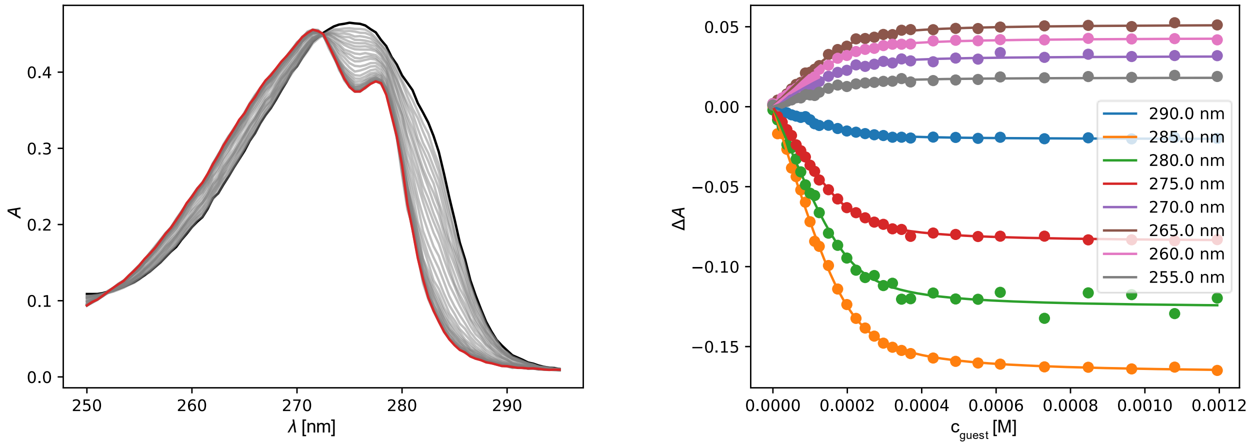

Figure S86. UV-Vis spectra recorded during UV-Vis titration of **1c** with LiOTf in 99.5% CH_3CN + 0.5% H_2O (black – first spectra, red – last spectra) (left); changes in absorbances (dots) at given wavelengths fitted to 1:1 model (solid lines) (right).

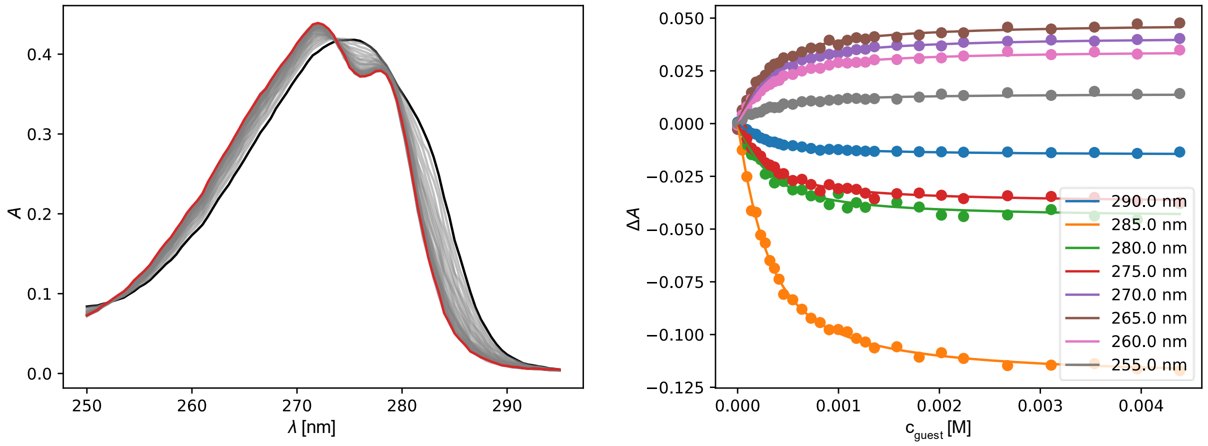

Figure S87. UV-Vis spectra recorded during UV-Vis titration of **1c** with NaOTf in 99.5% CH_3CN + 0.5% H_2O (black – first spectra, red – last spectra) (left); changes in absorbances (dots) at given wavelengths fitted to 1:1 model (solid lines) (right).

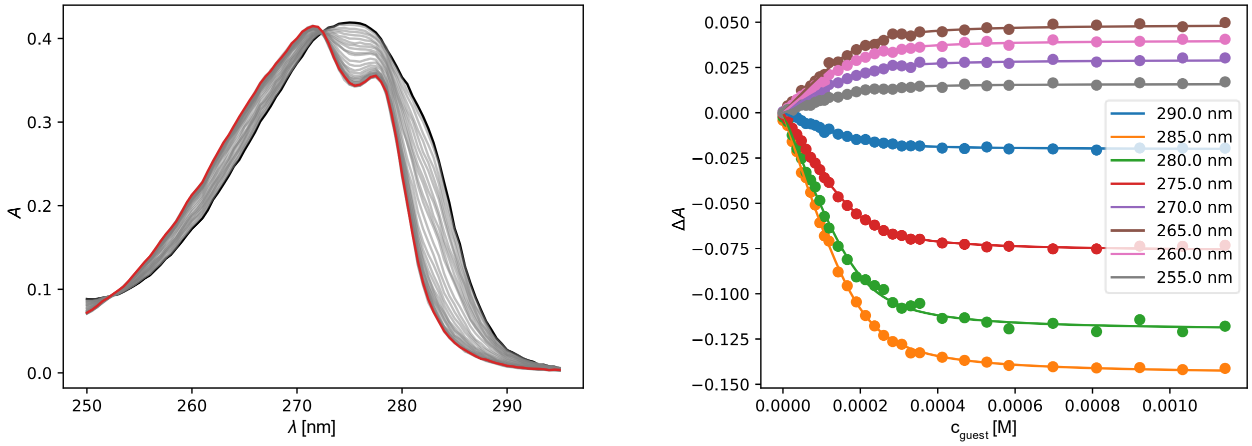

Figure S88. UV-Vis spectra recorded during UV-Vis titration of **1d** with LiOTf in 99.5% CH_3CN + 0.5% H_2O (black – first spectra, red – last spectra) (left); changes in absorbances (dots) at given wavelengths fitted to 1:1 model (solid lines) (right).

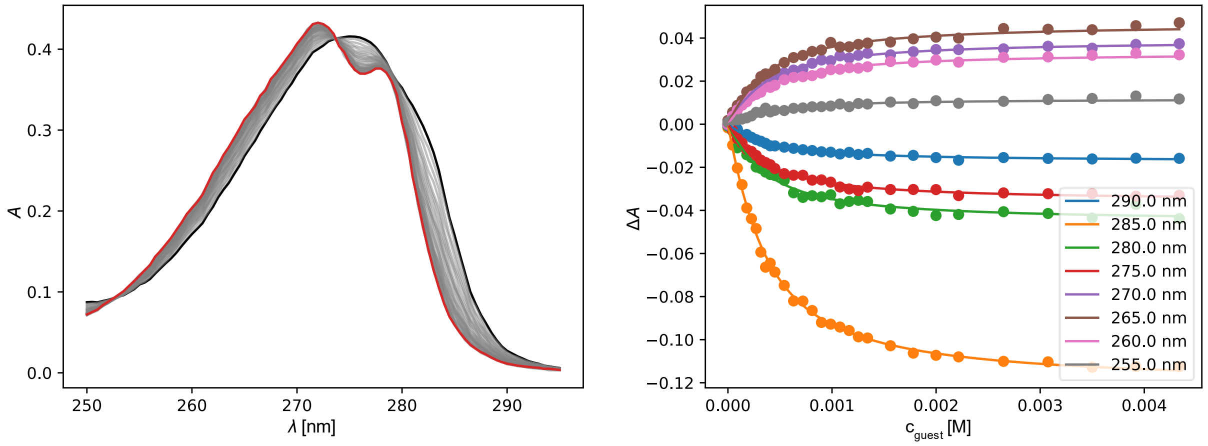

Figure S89. UV-Vis spectra recorded during UV-Vis titration of **1d** with NaOTf in 99.5% CH_3CN + 0.5% H_2O (black – first spectra, red – last spectra) (left); changes in absorbances (dots) at given wavelengths fitted to 1:1 model (solid lines) (right).

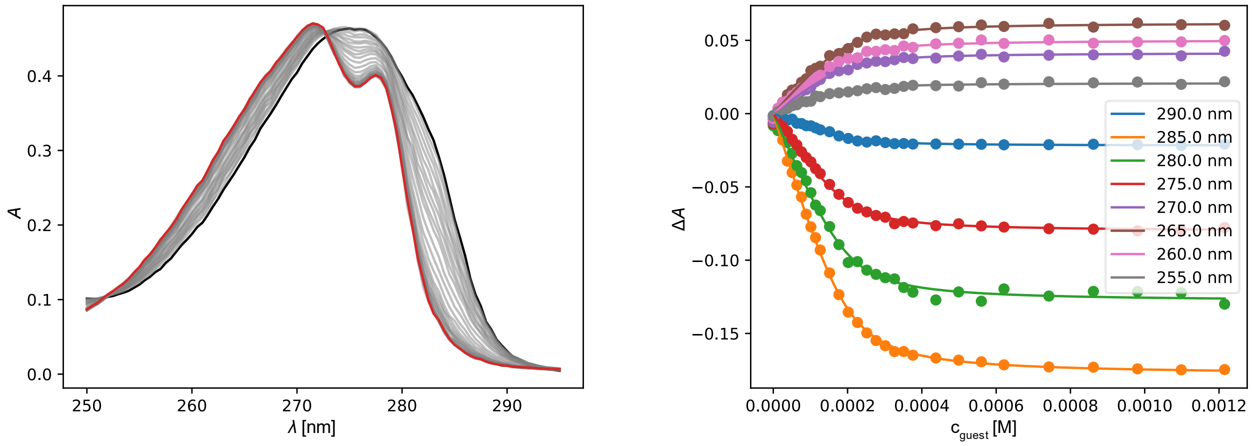

Figure S90. UV-Vis spectra recorded during UV-Vis titration of **1e** with LiOTf in 99.5% CH_3CN + 0.5% H_2O (black – first spectra, red – last spectra) (left); changes in absorbances (dots) at given wavelengths fitted to 1:1 model (solid lines) (right).

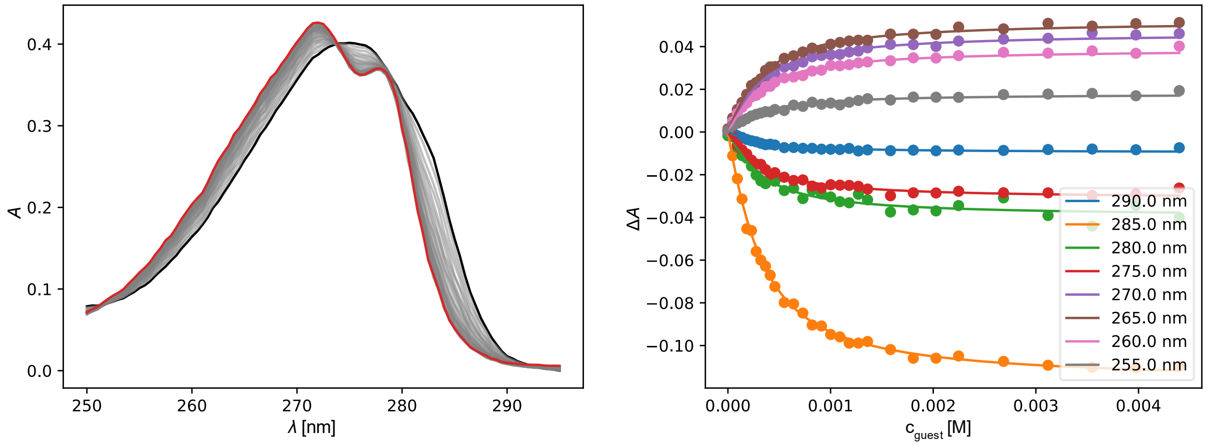

Figure S91. UV-Vis spectra recorded during UV-Vis titration of **1e** with NaOTf in 99.5% CH_3CN + 0.5% H_2O (black – first spectra, red – last spectra) (left); changes in absorbances (dots) at given wavelengths fitted to 1:1 model (solid lines) (right).

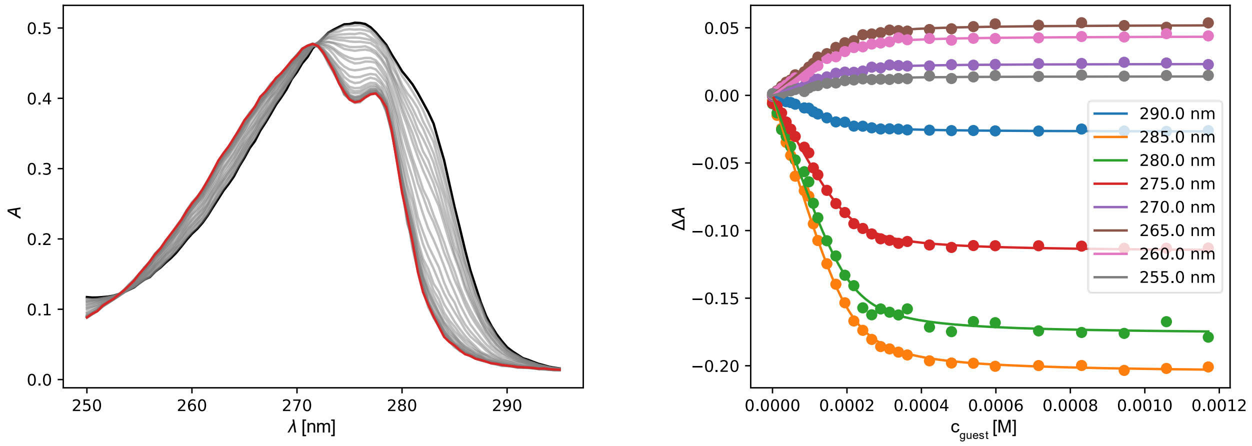

Figure S92. UV-Vis spectra recorded during UV-Vis titration of **1f** with LiOTf in 99.5% CH_3CN + 0.5% H_2O (black – first spectra, red – last spectra) (left); changes in absorbances (dots) at given wavelengths fitted to 1:1 model (solid lines) (right).

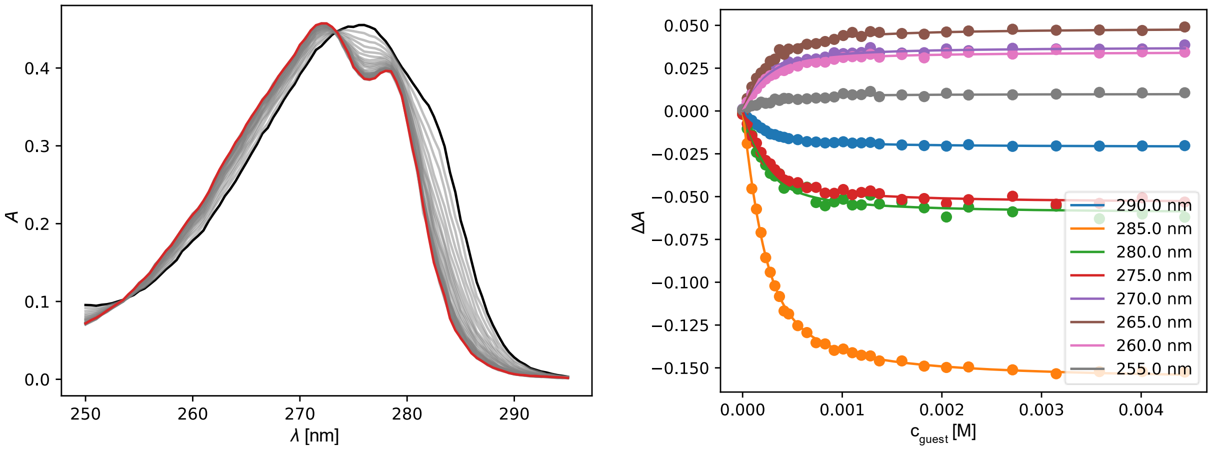

Figure S93. UV-Vis spectra recorded during UV-Vis titration of **1f** with NaOTf in 99.5% CH_3CN + 0.5% H_2O (black – first spectra, red – last spectra) (left); changes in absorbances (dots) at given wavelengths fitted to 1:1 model (solid lines) (right).

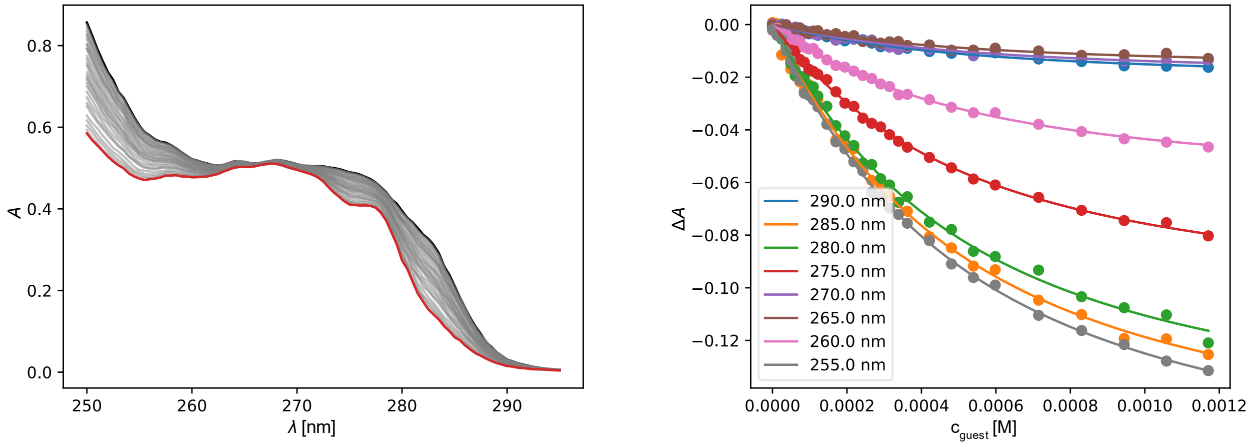

Figure S94. UV-Vis spectra recorded during UV-Vis titration of **1g** with LiOTf in 99.5% CH_3CN + 0.5% H_2O (black – first spectra, red – last spectra) (left); changes in absorbances (dots) at given wavelengths fitted to 1:1 model (solid lines) (right).

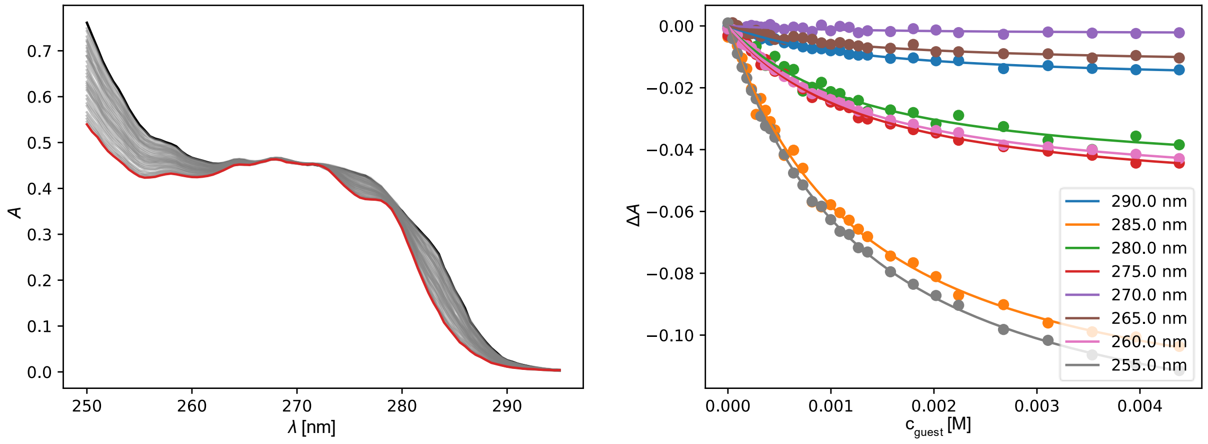

Figure S95. UV-Vis spectra recorded during UV-Vis titration of **1g** with NaOTf in 99.5% CH_3CN + 0.5% H_2O (black – first spectra, red – last spectra) (left); changes in absorbances (dots) at given wavelengths fitted to 1:1 model (solid lines) (right).

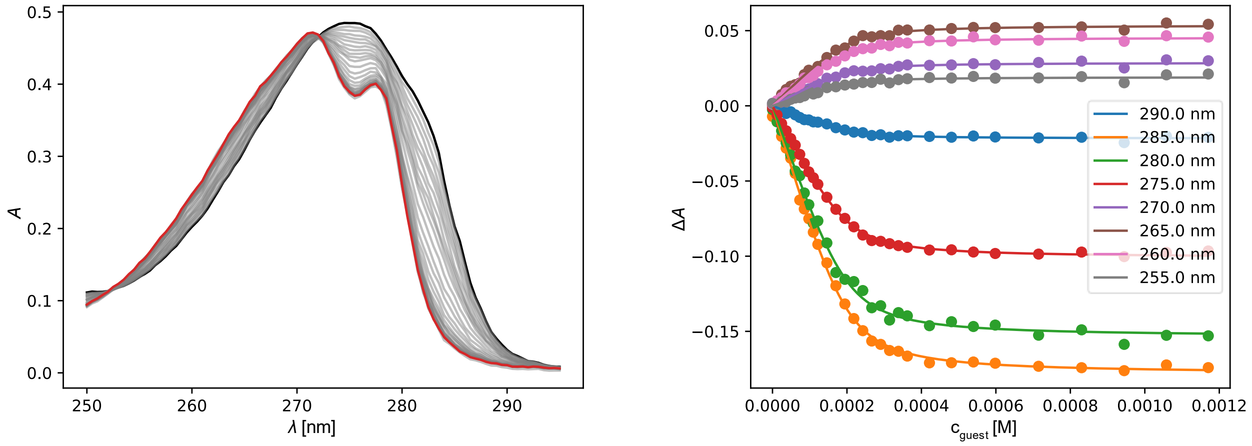

Figure S96. UV-Vis spectra recorded during UV-Vis titration of **1h** with LiOTf in 99.5% CH_3CN + 0.5% H_2O (black – first spectra, red – last spectra) (left); changes in absorbances (dots) at given wavelengths fitted to 1:1 model (solid lines) (right).

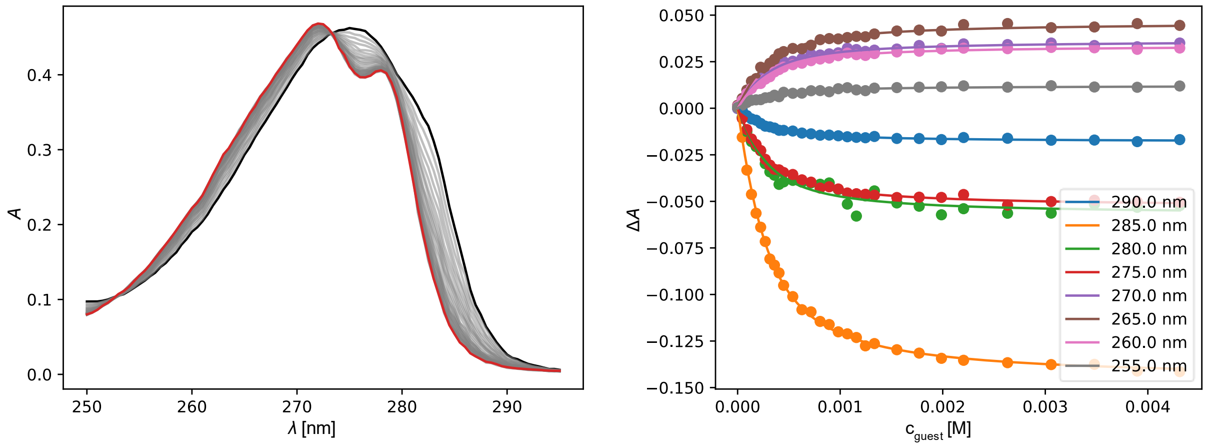

Figure S97. UV-Vis spectra recorded during UV-Vis titration of **1h** with NaOTf in 99.5% CH_3CN + 0.5% H_2O (black – first spectra, red – last spectra) (left); changes in absorbances (dots) at given wavelengths fitted to 1:1 model (solid lines) (right).

3. Crystallographic data

X-ray data for **1b**·LiBr·H_2O, **1f**, **1i,** and **1i**·LiCl·H_2O were collected on a SuperNova Agilent diffractometer using Cu Kα (λ = 1.54184 Å) radiation. X-ray data for **1b**·LiCl·H_2O and **1c**·LiCl·H_2O were collected on a XtaLAB Synergy, Dualflex, HyPix-Arc 150 diffractometer using Mo Kα (λ = 0.71073 Å) radiation. Data were processed using CrysAlisPro (Agilent Technologies, Version 1.171.35.21b).[36] X-ray data for **1f**·LiOTf were collected on a Bruker AXS D8 VENTURE (Bruker) using Mo Kα (λ = 0.71073 Å) radiation. The data processing and internal scaling were carried out with SAINT V8.40B (Bruker AXS Inc., 2019). The structures were solved by ShelXT and refined using ShelXL.[37,38]

Single crystals of **1b**·LiCl·H_2O, **1b**·LiBr·H_2O, and **1i**·LiCl·H_2O suitable for X-ray analysis were obtained by slow vapor diffusion of *n*-pentane into CH_2Cl_2 solutions of the corresponding compounds at ~5 °C. Single crystals of **1c**·LiCl·H_2O**, 1i,** and **1f**·LiOTf suitable for X-ray analysis were obtained by slow vapor diffusion of *n*-pentane into DCE solutions of the corresponding compounds at ~5 °C. Single crystals of **1f** suitable for X-ray analysis were obtained by slow cooling a hot solution of compound **1f** in ethyl acetate to room temperature.

ORTEP representations of all crystal structures are shown in Figures S98–S105. Crystal data and structure refinement details for all structures are summarized in Tables S6–S9.

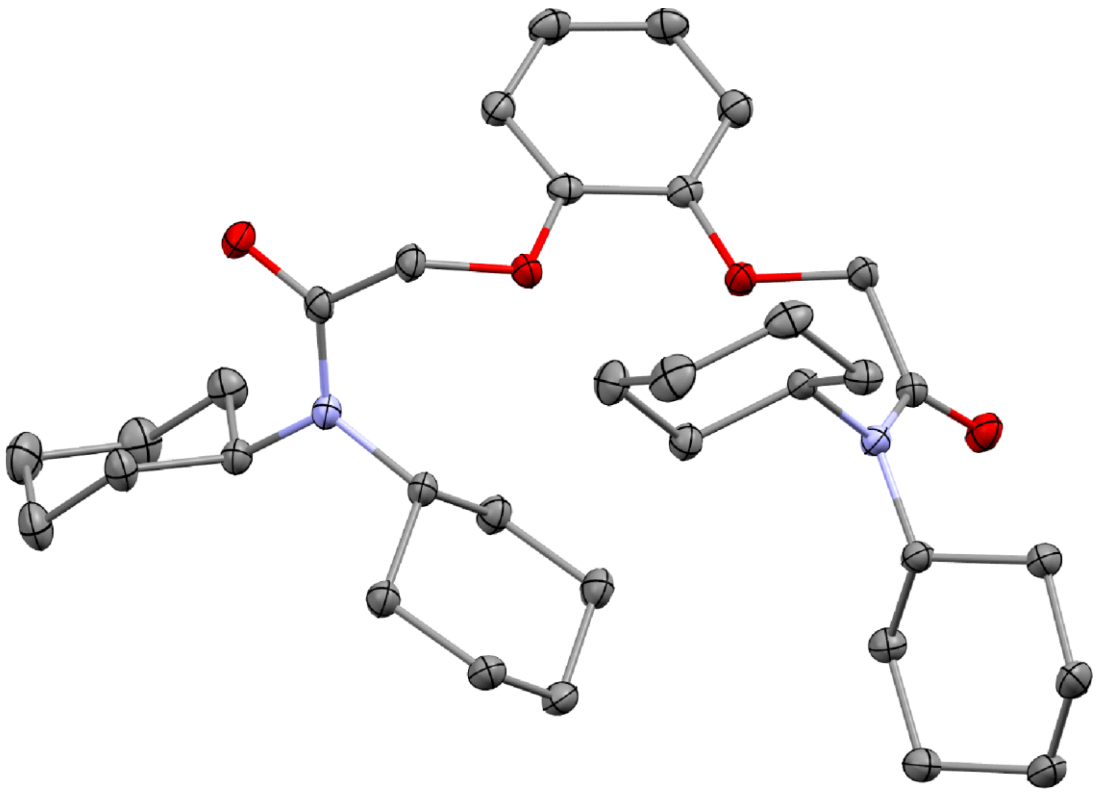

Figure S98. ORTEP view of **1f** with 50% probability displacement ellipsoids; non-acidic hydrogen atoms omitted for clarity.

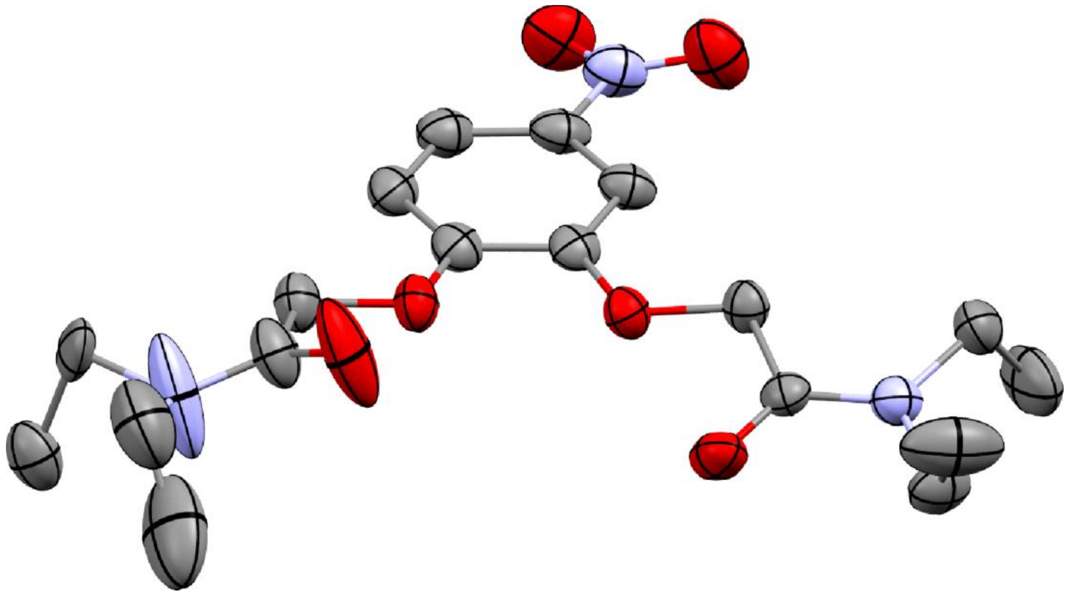

Figure S99. ORTEP view of **1i** with 50% probability displacement ellipsoids; non-acidic hydrogen atoms omitted for clarity.

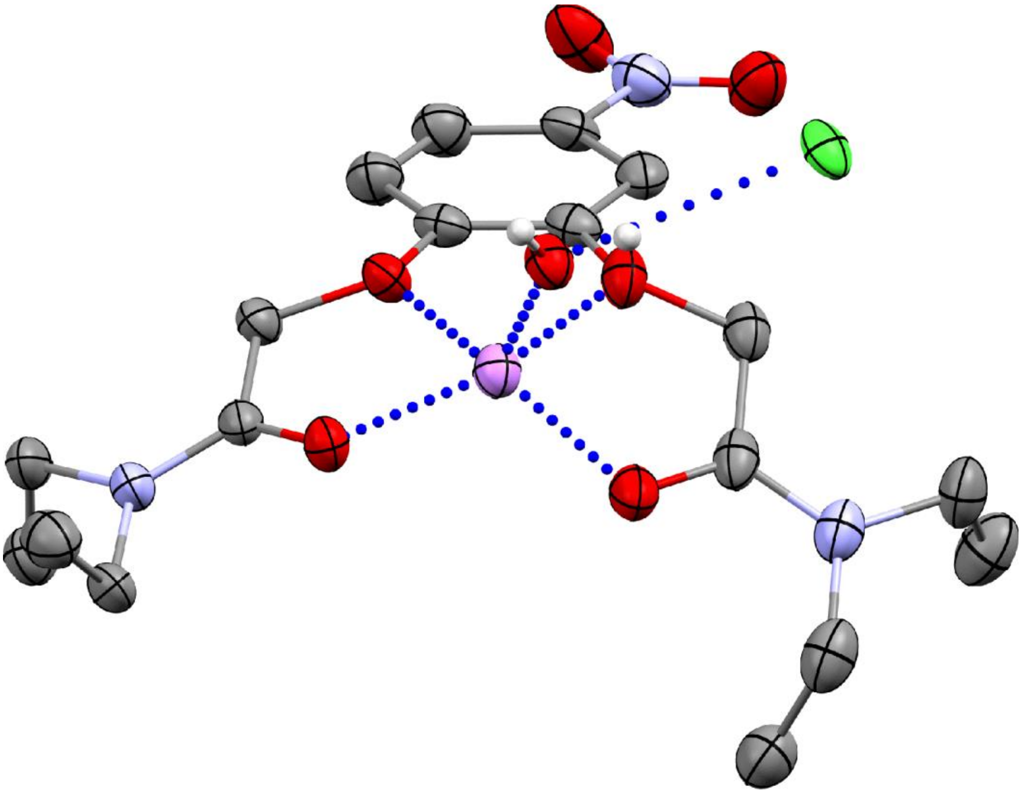

Figure S100. ORTEP view of complex **1i**·LiCl·H_2O, showing the Li^+ coordination environment; non-acidic hydrogen atoms omitted for clarity.

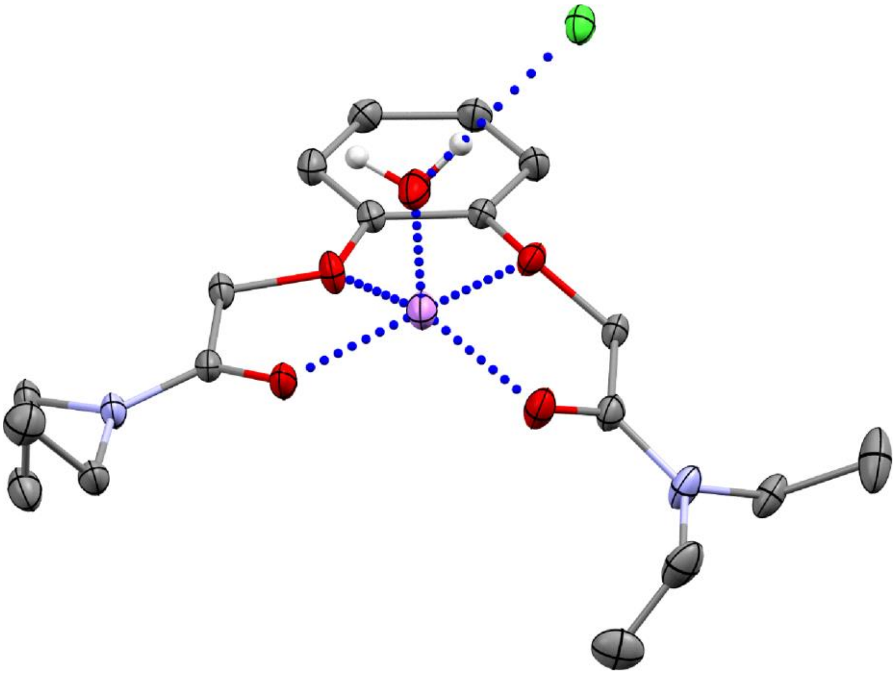

Figure S101. ORTEP view of complex **1b**·LiCl·H_2O, showing the Li^+ coordination environment; non-acidic hydrogen atoms omitted for clarity.

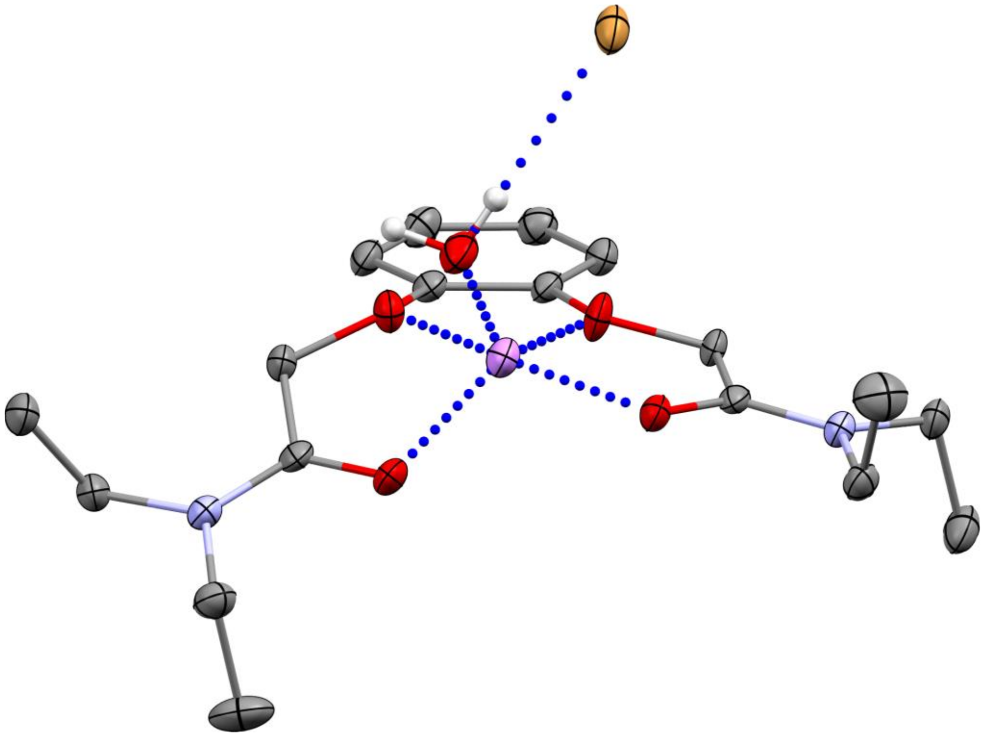

Figure S102. ORTEP view of complex **1b**·LiBr·H_2O, showing the Li^+ coordination environment; non-acidic hydrogen atoms omitted for clarity.

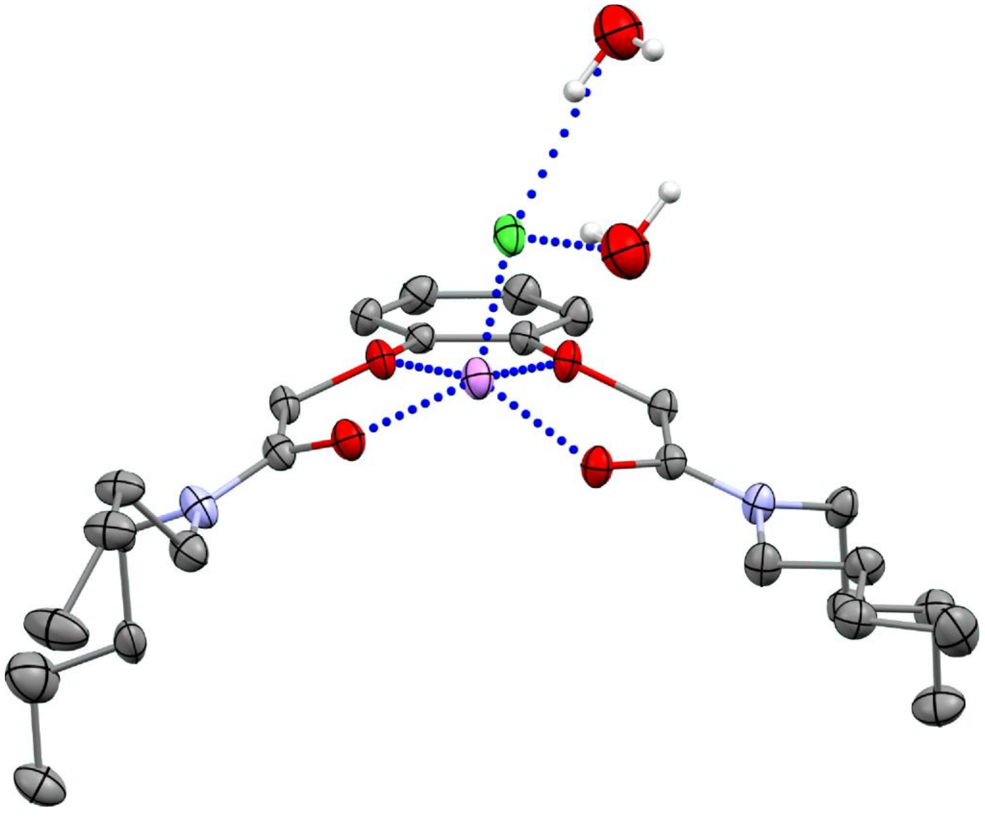

Figure S103. ORTEP view of complex **1c**·LiCl·H_2O, showing the Li^+ coordination environment; non-acidic hydrogen atoms omitted for clarity.

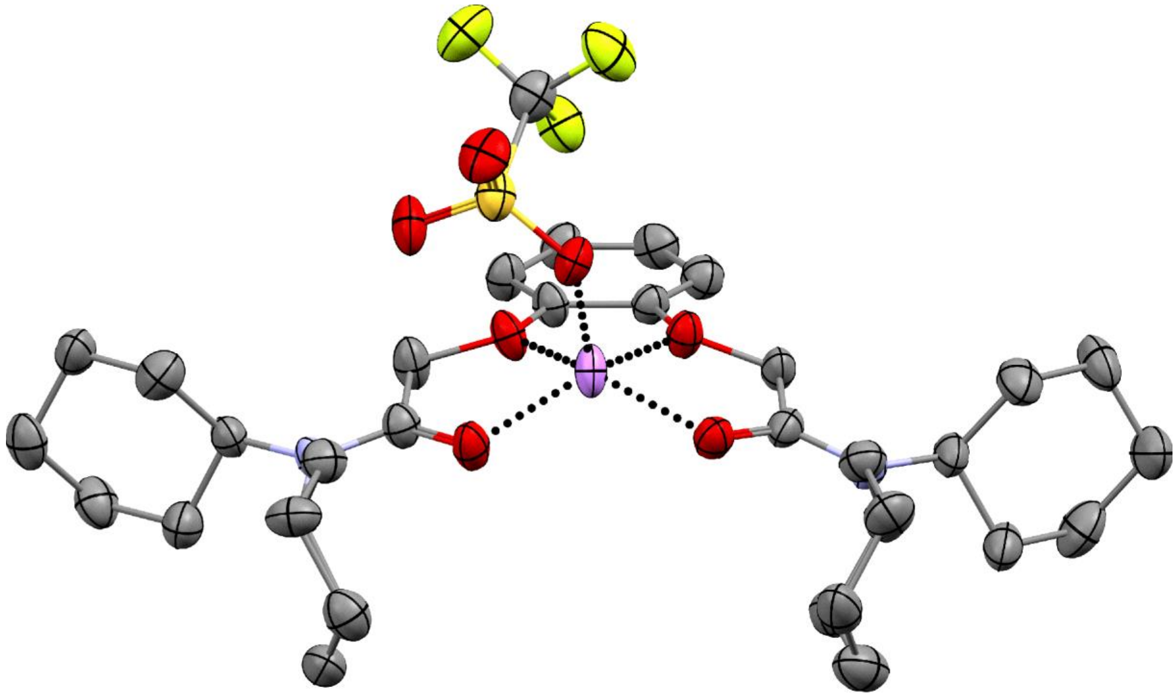

Figure S104. ORTEP view of complex **1f**·LiOTf, showing the Li^{+} coordination environment; non-acidic hydrogen atoms and disordered atoms omitted for clarity.

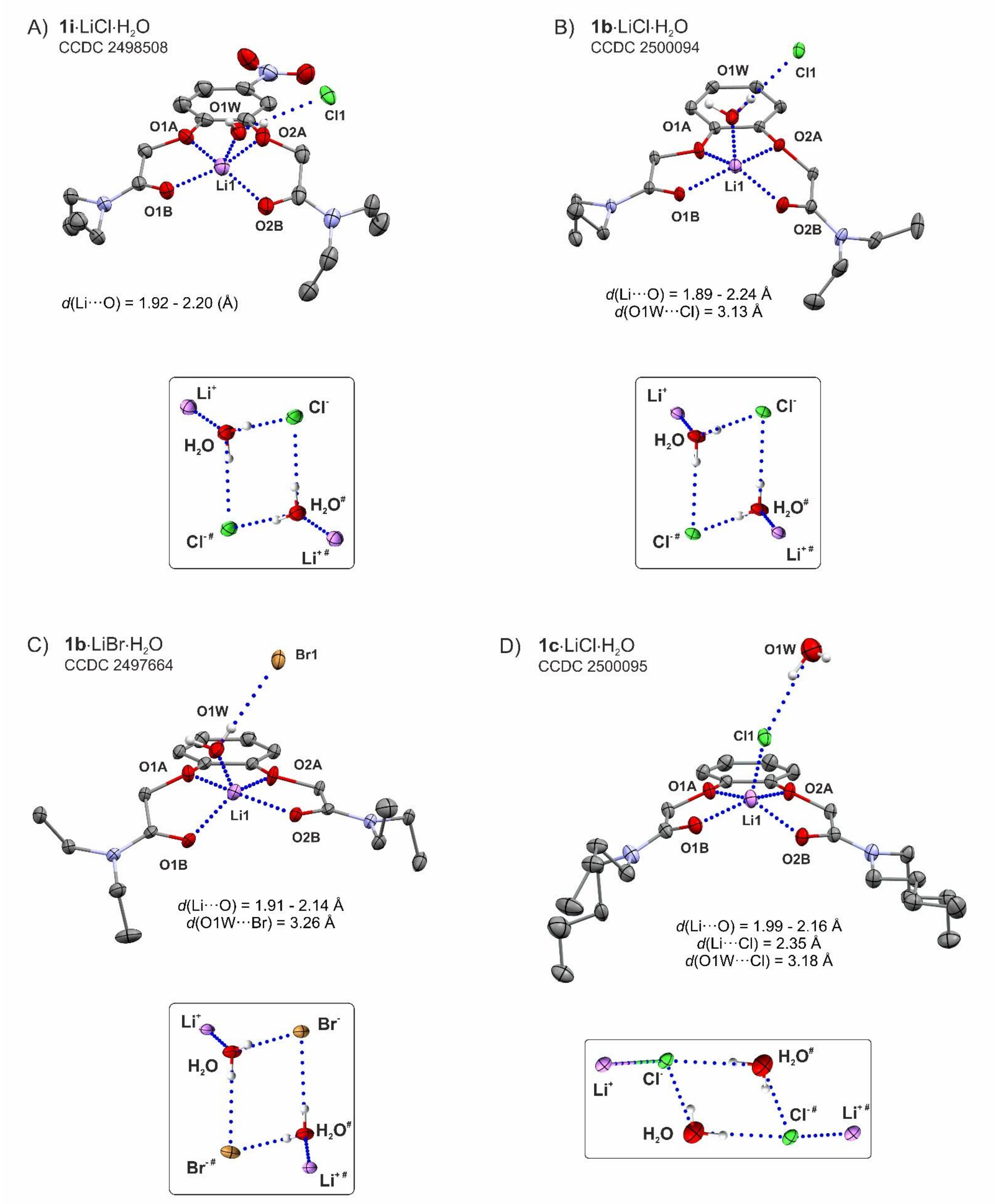

Figure S105. Supramolecular water–halide cluster motif observed in **1i**·LiCl·H_2O (A), **1b** LiCl·H_2O (B), **1b** LiBr·H_2O (C), and **1c**·LiCl·H_2O (D); non-acidic hydrogen atoms omitted for clarity; symmetry-equivalent atoms are denoted by the # symbol.

Table S6. Crystal data and structure refinement details for **1b**·LiBr·H_2O and **1b**·LiCl·H_2O.

Compound	**1b**·LiBr·H_2O	**1b**·LiCl·H_2O
Empirical formula	$C_{18}H_{30}N_2O_5LiBr$	$C_{18}H_{30}ClLiN_2O_5$
Moiety formula	$C_{18}H_{28}N_2O_4$·LiBr·H_2O	$C_{18}H_{28}N_2O_4$·LiCl·H_2O
Formula weight	441.29	396.83
CCDC No.	2497664	2500094
Wavelength	1.54184 Å	0.71073 Å
Crystal system	monoclinic	triclinic
Space group	*P*2$_1$*/n*	*P*-1
Unit cell dimensions	*a* = 10.5152(1) Å<br>*b* = 17.3115(2) Å<br>*c* = 23.9186(3) Å<br>β = 101.650 (1)°	*a* = 9.6950 (2) Å, α = 89.462 (1) °<br>*b* = 9.7566 (2) Å, β = 67.027(2) °<br>*c* = 11.9967 (2) Å, γ = 85.694 (1) °
Volume	4264.16(8) Å^3	1041.58(4) Å^3
Z	8	2
Density Calc.	1.375 g/cm^3	1.265 g/cm^3
Absorption coefficient	2.872 mm^{-1}	0.213 mm^{-1}
F(000)	1840	424
Crystal	colorless block	colorless plate
Crystal size	0.18 × 0.14 × 0.04 mm	0.219 × 0.178 × 0.114 mm
Index ranges	-12≤ h ≤ 9,<br>-21 ≤ k ≤ 20,<br>-25 ≤ l ≤ 29	-18≤ h ≤ 18,<br>-17 ≤ k ≤ 12,<br>-22 ≤ l ≤ 22
Reflections collected<br>(all / independent)	29355/8021	54675/11836
Absorption correction	multi-scan	Gaussian
Refinement method	Full-matrix least-squares on F^2	Full-matrix least-squares on F^2
Restraints / parameters	6/511	7/280
Goodness-of-fit on F^2	1.048	1.0072
Final R indices [$F^2 > 2\sigma(F^2)$]	*R* = 0.028, w*R* = 0.069	*R* = 0.046, w*R* = 0.127
R indices (all data)	*R* = 0.030, w*R* = 0.071	*R* = 0.066, w*R* = 0.138

Table S7. Crystal data and structure refinement details for **1f** and **1f**·LiOTf.

Compound	**1f**	**1f**·LiOTf	
Empirical formula	$C_{34}H_{52}N_2O_4$	$C_{76}H_{118}ClF_6Li_2N_4O_{14}S_2$	
Moiety formula	$C_{34}H_{52}N_2O_4$	$2[C_{34}H_{52}N_2O_4]\cdot 2CF_3LiO_3S\cdot$ $\cdot 0.5[C_2H_4Cl_2]\cdot C_5H_{12}$	
Formula weight	552.77	1535.16	
CCDC No.	2497716	2497721	
Wavelength	1.54184 Å	0.71073 Å	
Crystal system	orthorhombic	triclinic	
Space group	$Pca2_1$	$P1$	
Unit cell dimensions	a = 18.2011(2) Å	a = 9.4208(7) Å	α = 76.803(3) °
	b = 8.3430(1) Å	b = 15.3478(1) Å	β = 67.027(2) °
	c = 20.5757(2) Å	c = 20.5757(2) Å	γ = 72.840(3)°
Volume	3124.46(6) Å^3	2123.2(3) Å^3	
Z	4	1	
Density Calc.	1.175 g/cm^3	1.201 g/cm^3	
Absorption coefficient	0.596 mm^{-1}	0.166 mm^{-1}	
F(000)	1208	819	
Crystal	colorless block	colorless block	
Crystal size	0.14 × 0.10 × 0.03 nm	0.22 × 0.02 × 0.01 nm	
Index ranges	-22≤ h ≤ 22, -10 ≤ k ≤ 5, -24 ≤ l ≤ 24	-12≤ h ≤ 12, -20 ≤ k ≤ 20, -21 ≤ l ≤ 21	
Reflections collected (all / independent)	20232/5431	94717 /19640	
Absorption correction	multi-scan	multi-scan	
Refinement method	Full-matrix least-squares on F^2	Full-matrix least-squares on F^2	
Restraints / parameters	1/361	64/967	
Goodness-of-fit on F^2	1.038	1.034	
Final R indices [$F^2 > 2\sigma(F^2)$]	R = 0.023, wR = 0.060	R = 0.238, wR = 0.145	
R indices (all data)	R = 0.024, wR = 0.060	R = 0.240, wR = 0.200	

Table S8. Crystal data and structure refinement details for **1i** and **1i**·LiCl·H_2O.

Compound	**1i**		**1i**·LiCl·H_2O	
Empirical formula	$C_{18}H_{27}N_3O_6$		$C_{18}H_{29}ClLiN_3O_7$	
Moiety formula	$C_{18}H_{27}N_3O_6$		$C_{18}H_{27}N_3O_6$·LiCl·H_2O	
Formula weight	381.42		441.83	
CCDC No.	2498482		2498508	
Wavelength	1.54184 Å		1.54184 Å	
Crystal system	trigonal		monoclinic	
Space group	$P3_1$		$P2_1/c$	
Unit cell dimensions	a = 12.7588(3) Å	α = 90 °	a = 10.0981(4)Å	β = 99.107(3) °
	b = 12.7588(3) Å	β = 90 °	b = 16.8557(5) Å	
	c = 20.8043(6) Å	γ = 120 °	c = 13.1119(4) Å	
Volume	2932.9(2) Å^3		2203.7(1) Å^3	
Z	6		4	
Density Calc.	1.296		1.332	
Absorption coefficient	0.814		1.911	
F(000)	1224		936	
Crystal	Colorless needle		Colorless plate	
Crystal size	0.35 x 0.03 x 0.02		0.24 x 0.20 x 0.03	
Index ranges	-15≤ h ≤ 15,<br>-14 ≤ k ≤ 15,<br>-24 ≤ l ≤ 24		-12≤ h ≤ 9,<br>-13 ≤ k ≤ 20,<br>-13 ≤ l ≤ 15	
Reflections collected<br>(all / independent)	20125/6050		8151/4089	
Absorption correction	multi-scan		multi-scan	
Refinement method	Full-matrix least-squares on F^2		9/515	
Restraints / parameters	9/515		0/283	
Goodness-of-fit on F^2	1.059		1.041	
Final R indices [$F^2 > 2\sigma(F^2)$]	R = 0.070, wR = 0.192		R = 0.070, wR = 0.194	
R indices (all data)	R = 0.076, wR = 0.203		R = 0.074, wR = 0.198	

Table S9. Crystal data and structure refinement details for **1c**·LiCl·H_2O.

Compound	**1c**·LiCl·H_2O	
Empirical formula	$C_{26}H_{46}ClLiN_2O_5$	
Moiety formula	$C_{26}H_{44}N_2O_4$·LiCl·H_2O	
Formula weight	509.04	
CCDC No.	2500095	
Wavelength	0.71073 Å	
Crystal system	monoclinic	
Space group	$P2_1/c$	
Unit cell dimensions	a = 14.5238(4) Å	β = 98.965(3)°
	b = 19.8868(5) Å	
	c = 10.0302(3) Å	
Volume	2861.7(1) Å^3	
Z	4	
Density Calc.	1.182 g/cm^3	
Absorption coefficient	0.169 mm^{-1}	
F(000)	1104	
Crystal	colorless plate	
Crystal size	0.81 × 0.54 × 0.25 mm	
Index ranges	$-27 \leq h \leq 26$,<br>$-35 \leq k \leq 36$,<br>$-17 \leq l \leq 18$	
Reflections collected (all / independent)	92134/19017	
Absorption correction	Gaussian	
Refinement method	Full-matrix least-squares on F^2	
Restraints / parameters	14/350	
Goodness-of-fit on F^2	1.032	
Final R indices [$F^2 > 2\sigma(F^2)$]	R = 0.101, wR = 0.278	
R indices (all data)	R = 0.178, wR = 0.329	

4. Extraction Experiments

All extraction experiments were performed using model salt mixtures designed to reflect key ionic features of lithium brines, as the use and export of authentic lithium-bearing brines are subject to regulatory restrictions.

Chloroform was selected as a solvent for NMR-controlled extraction experiments as a practical, affordable benchmark solvent, widely used in the lithium molecular extractants literature, and therefore ensuring continuity with previous studies (see Table S1).

4.1 Preparation of solid mixtures M1-M3

Preparation of model salt mixture M1 (mimicking the Zabuye salt lake deposit)

LiCl (435 mg), KCl (3.35 g), and NaCl (46.22 g) were dissolved in ~100 mL of hot water. The solution was then concentrated on a rotary evaporator (~20 mbar, 100°C) to a solid residue, which was transferred to a mortar and ground. Lithium content in dry mass – 1425 ppm.

Preparation of model salt mixture M2 (mimicking Salar de Atacama salt deposit)

$LiCl{\cdot}H_2O$ (1.828 g), KCl (4.53 g), NaCl (12.78 g), and $MgCl_2{\cdot}6H_2O$ (9.82 g) were dissolved in ~50mL of hot water. The solution was then concentrated on a rotary evaporator (~20 mbar, 100°C) to a solid residue, which was transferred to a mortar and ground. Lithium content in dry mass – 6950 ppm.

Preparation of battery black mass M3

The spent $LiFePO_4$ batteries were first fully discharged to 0 V using resistors, then systematically disassembled into their constituent components, namely steel cases, electrode laminates, and the polymeric separator. The electrode laminate consisted of an aluminum foil bearing the $LiFePO_4$ cathode coating and a copper foil bearing the graphite anode coating. The Al and Cu foils were separated, cut into small pieces, and placed in Erlenmeyer flasks, where they were stirred neat with a cylindrical PTFE-coated stirring bar for 12–15 h (overnight). The stirring bar acted as an abrasive element and facilitated detachment of the active material from the foil surfaces.

The resulting black mass was passed through a set of stainless-steel sieves, with the finest fraction collected on a 200 μm sieve. This fine fraction was further processed by dispersing it in $CHCl_3$ (100 mL per ~10 g of black mass) under continuous magnetic stirring overnight. The suspension was then filtered through a Schott funnel and dried under vacuum to afford purified active material. The overall disassembly workflow and the isolated fractions are shown in Figure S106.

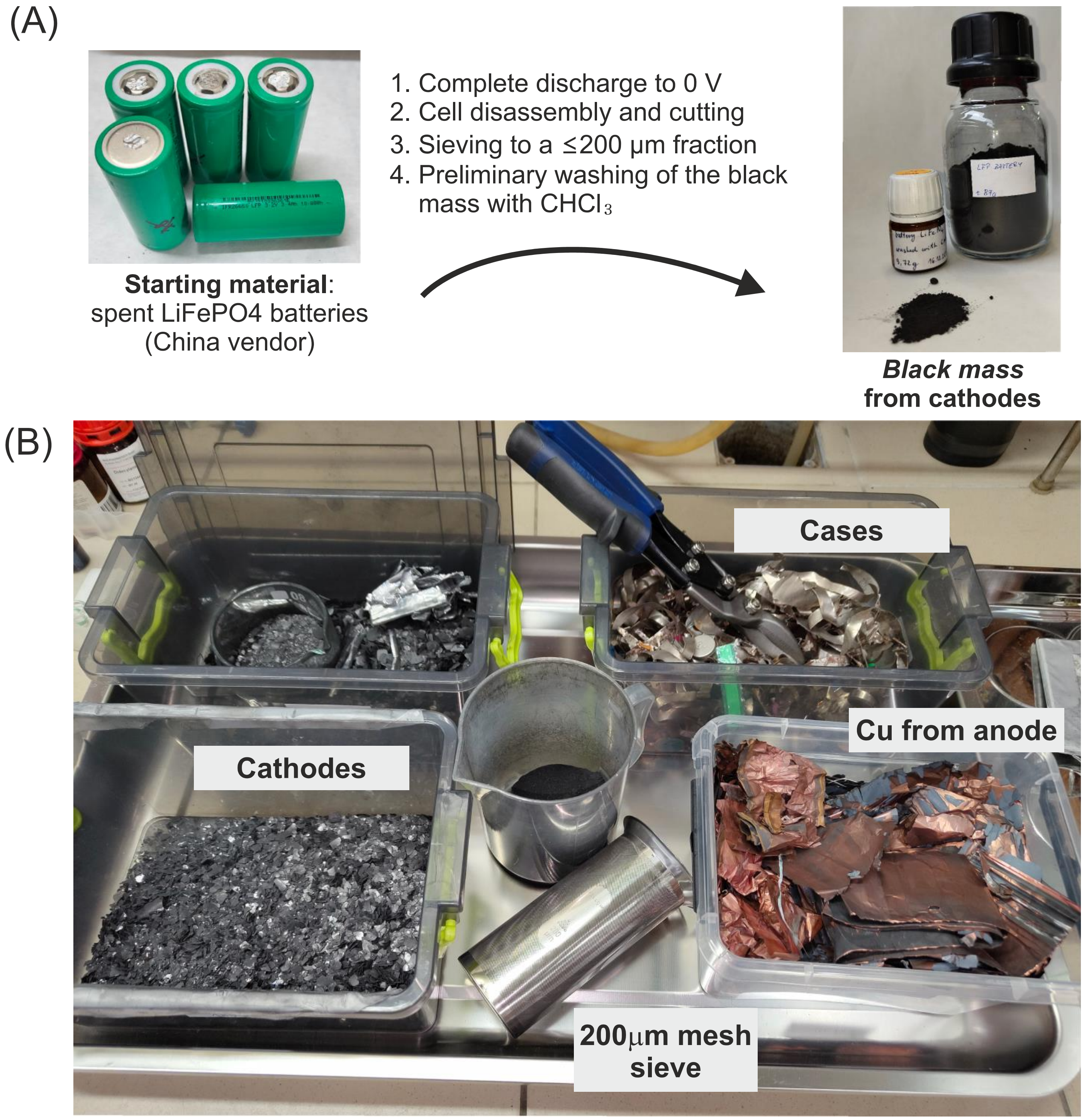

Figure S106. Preparation of black mass from spent $LiFePO_4$ batteries. (A) Starting cylindrical cells (China vendor) and black mass obtained from the cathode laminate. (B) Pictures of the disassembly workflow showing separated cathodes, steel cases, copper foils from the anode, and the 200 µm sieve used to collect the fine black-mass fraction.

4.2 Analytical scale SLE and LLE experiments

Determination of apparent solubility of LiCl and LiBr in $CDCl_3$

Two 2 mL vials were loaded with 3.6 mg of $LiCl{\cdot}H_2O$ and 5.2 mg of LiBr, respectively, and 0.7 mL of deacidified $CDCl_3$ was added. The samples were mixed for 72 h at 40 rpm on a SU1500 Sunlab Disk Rotator (vs the standard 2 h used for SLE experiments), then filtered through a 0.45 µm PTFE syringe filter into NMR tubes, and ^{7}Li NMR spectra were recorded (Figure S107). No signals attributable to dissolved lithium salts were observed, indicating that LiCl and LiBr remain essentially insoluble in $CDCl_3$ under these conditions.

(A)

(B)

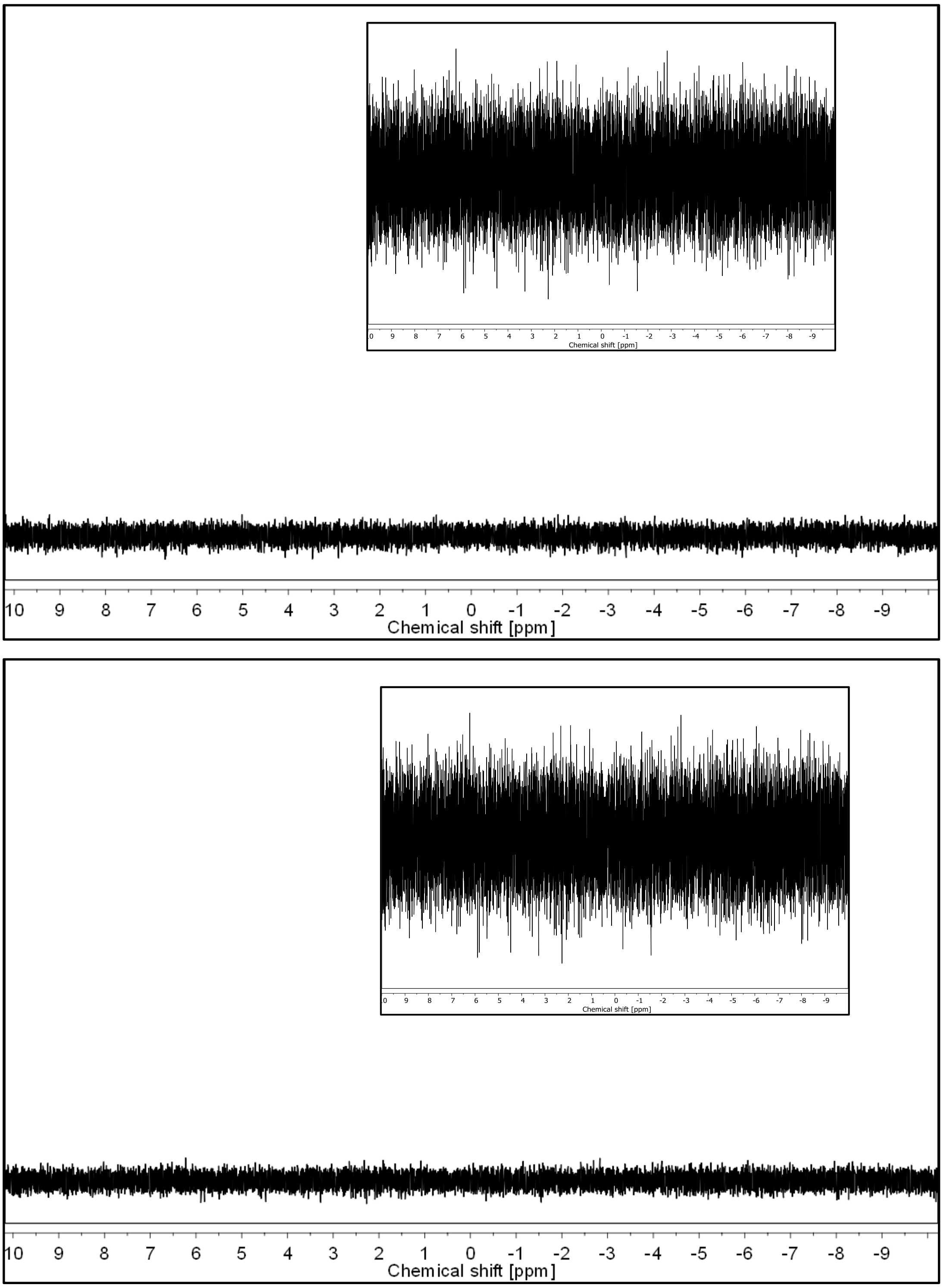

Figure S107. ^{7}Li NMR spectra of $CDCl_3$ solutions obtained after 72 h contact of $LiCl{\cdot}H_2O$ (A) and LiBr (B) with $CDCl_3$ in the absence of extractant **1**, showing no detectable dissolved lithium salts.

Protocol for SLE experiments for 1a–h

2 mL-vials were loaded with a weighted amount of individual salts: 3.6 mg (0.06 mmol) of $LiCl \cdot H_2O$, 3.5 mg (0.06 mmol) of NaCl, 4.5 mg (0.06 mmol) of KCl (~10 molar excess per corresponding host), salt mixtures **M1** (100 mg), **M2** (100 mg), and **M3** (300 mg). For the vials containing salts and their mixtures, 0.7 mL of a 10.0 mM solution of **1a–h** in deacidified $CDCl_3$ was added, while for the vial containing **M3**, 1.0 mL of the same solution was added. The samples were mixed for 2 hours at 40 rpm on the SU1500 Sunlab LCD Disk Rotator. The vial contents were then drawn into a syringe and filtered through a 0.45 μm PTFE syringe filter into NMR tubes, and the spectra were recorded (Figures S108-125)

Protocol for LLE experiments with 1b

4 mL-vials equipped with stirring bars were loaded with 1.5 mL of aqueous 1 M solutions of LiCl, LiBr, LiOTf, mixture of LiCl, NaCl, KCl, $MgCl_2$, and $CaCl_2$ (1M each, 5M total), and $LiNTf_2$, and 1.5 mL of a 10.0 mM solution of **1b** in deacidified $CDCl_3$ was added. The biphasic mixture was vigorously stirred for 16 h at 1500 rpm. Stirring was then turned off, and the mixtures were left to separate into phases. The organic phases were transferred into NMR tubes, and their spectra were recorded (Figures S127-128).

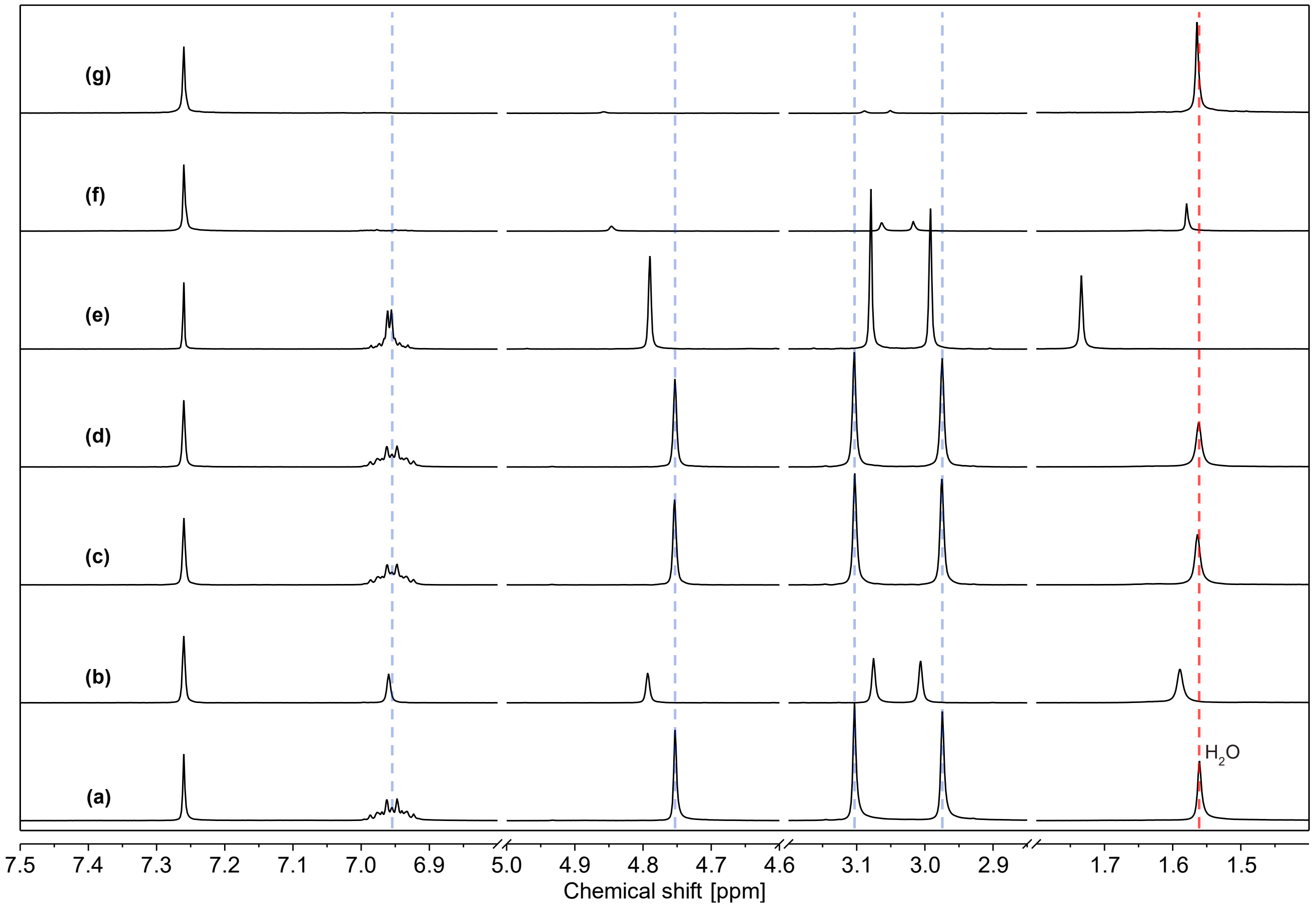

Figure S108. Selected regions of ^{1}H NMR spectra of solutions obtained after SLE into **1a** solution in $CDCl_3$, **(a)** stock **1a** solution, **(b)** $LiCl{\cdot}H_2O$, **(c)** NaCl, **(d)** KCl, **(e) M1**, **(f) M2**, **(g) M3**.

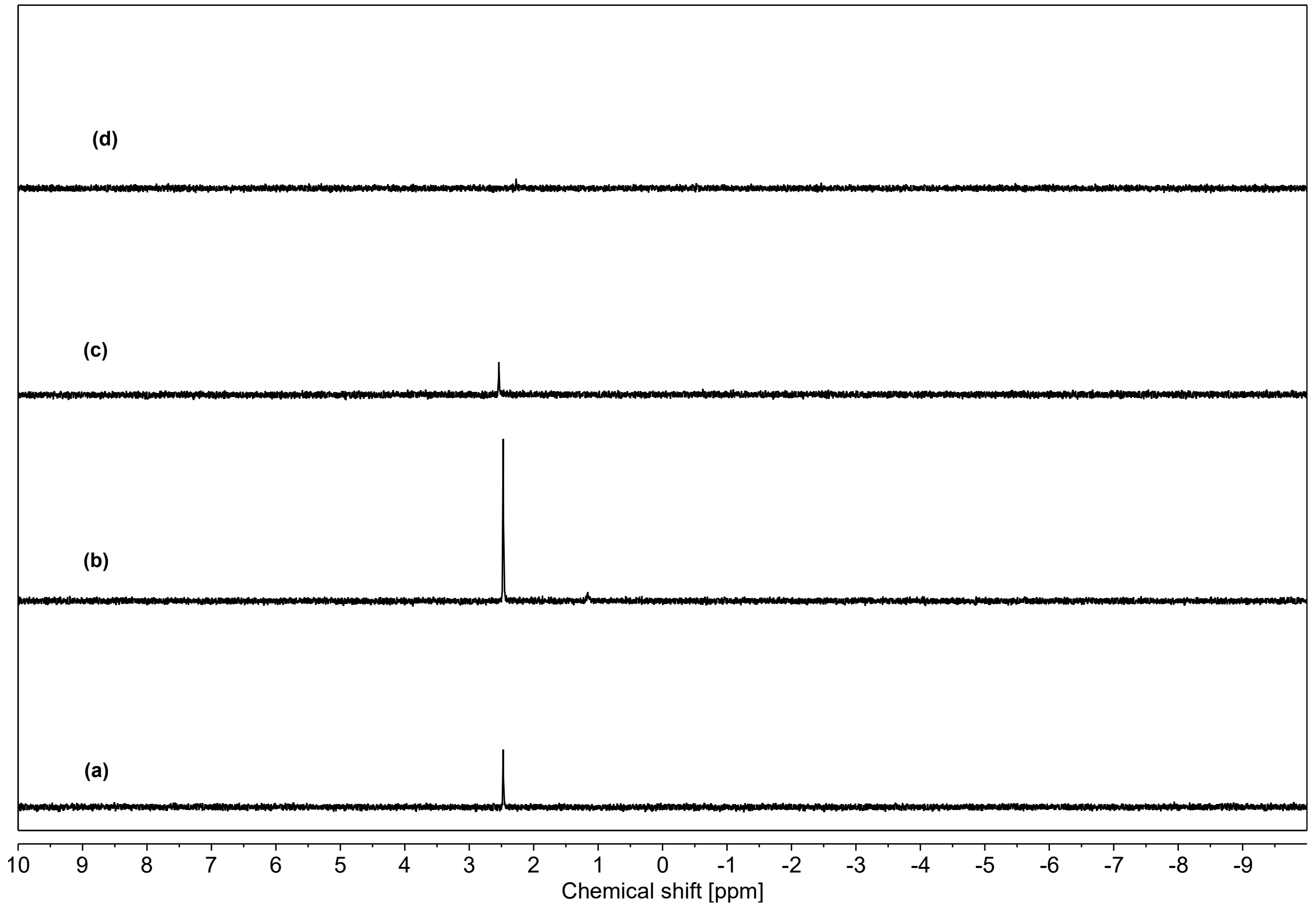

Figure S109. ^{7}Li NMR spectra of solutions obtained after SLE into **1a** solution in $CDCl_3$, **(a)** $LiCl{\cdot}H_2O$, **(b) M1**, **(c) M2**, **(d) M3**.

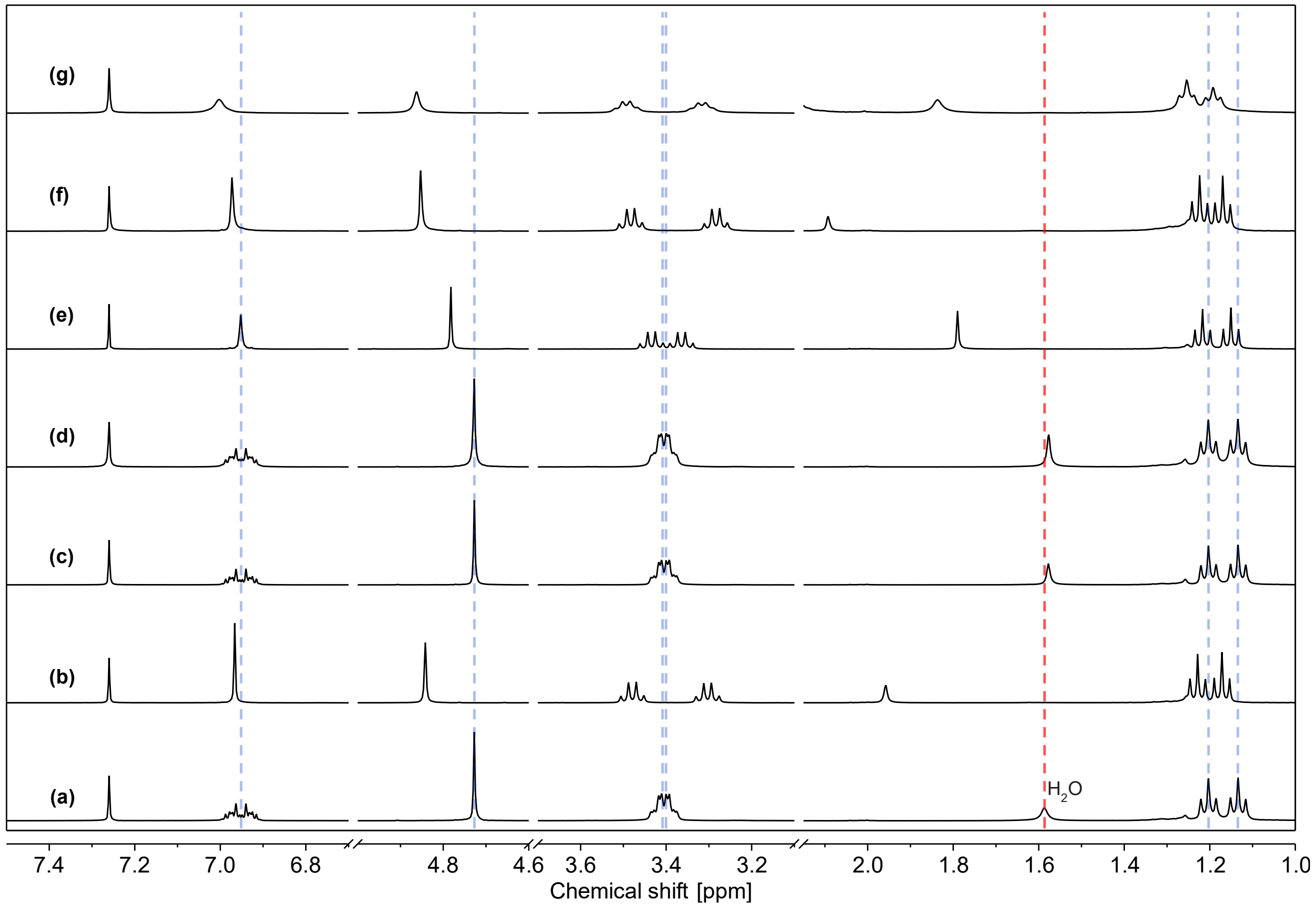

Figure S110. Selected regions of ^{1}H NMR spectra of solutions obtained after SLE into **1b** solution in $CDCl_3$, **(a)** stock **1b** solution, **(b)** $LiCl{\cdot}H_2O$, **(c)** NaCl, **(d)** KCl, **(e) M1**, **(f) M2**, **(g) M3**.

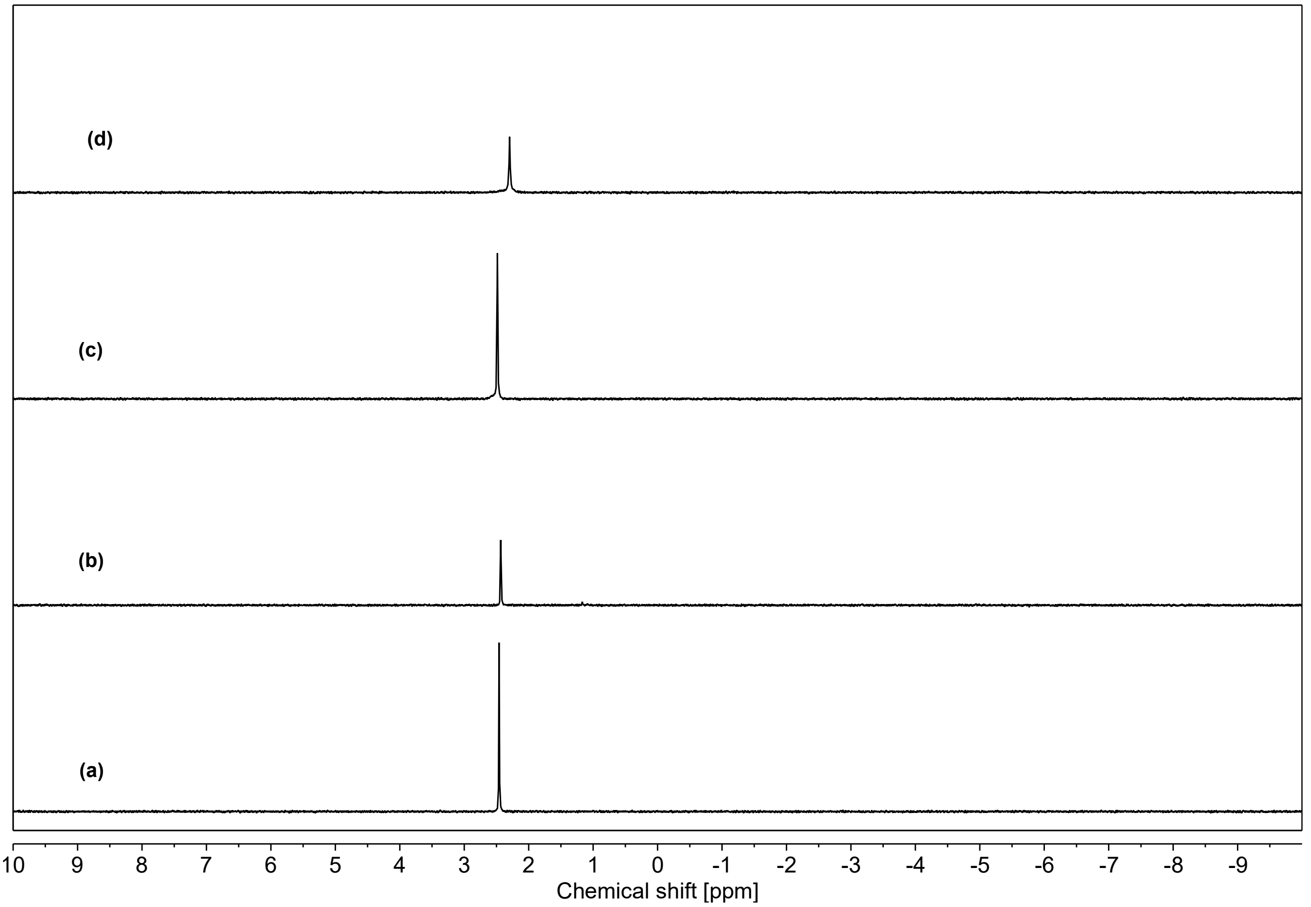

Figure S111. ^{7}Li NMR spectra of solutions obtained after SLE into **1b** solution in $CDCl_3$ **(a)** $LiCl{\cdot}H_2O$, **(b) M1**, **(c) M2**, **(d) M3**.

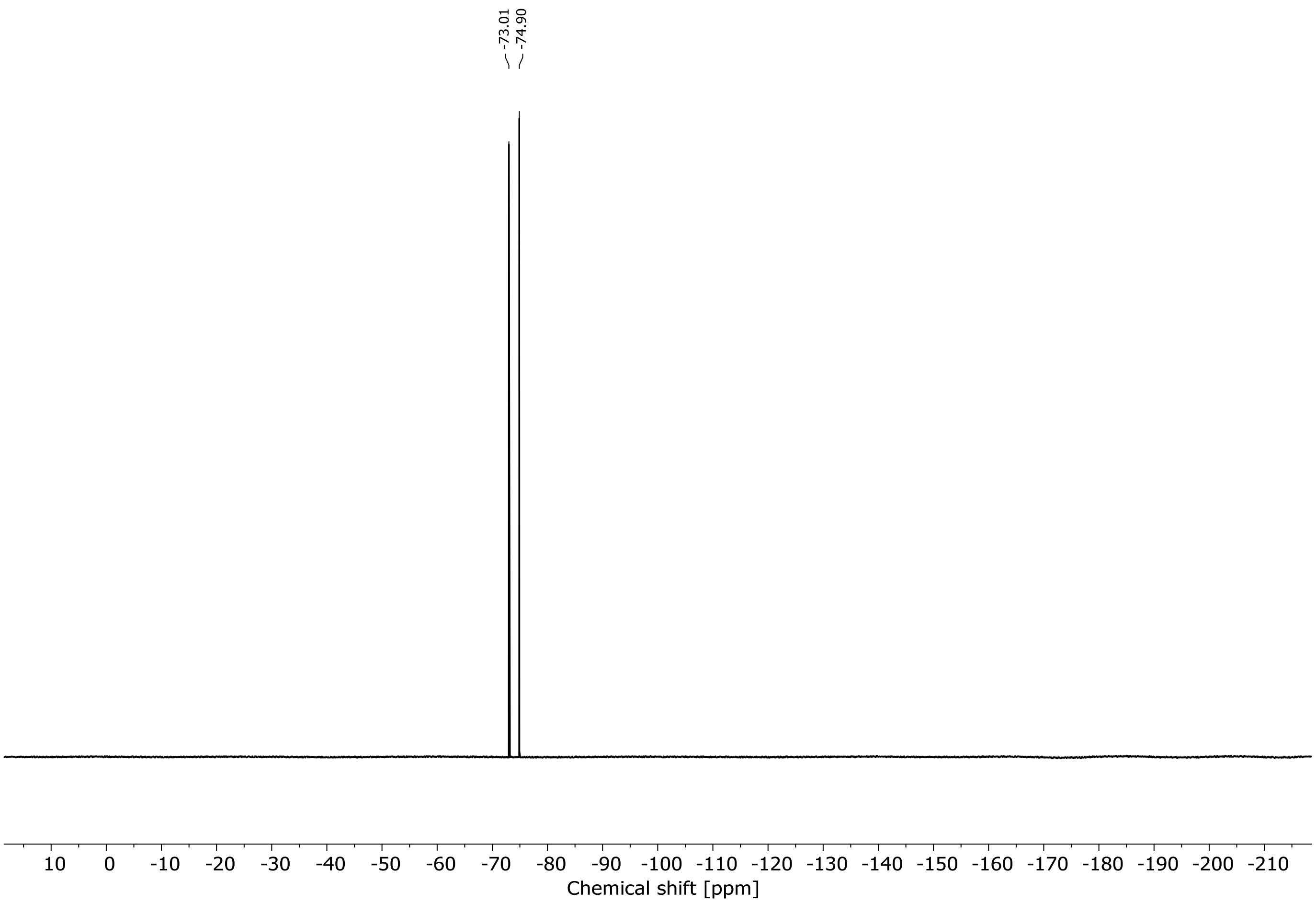

Figure S112. ^{19}F NMR spectra of the solution obtained after SLE from **M3** into **1b** solution in $CDCl_3$.

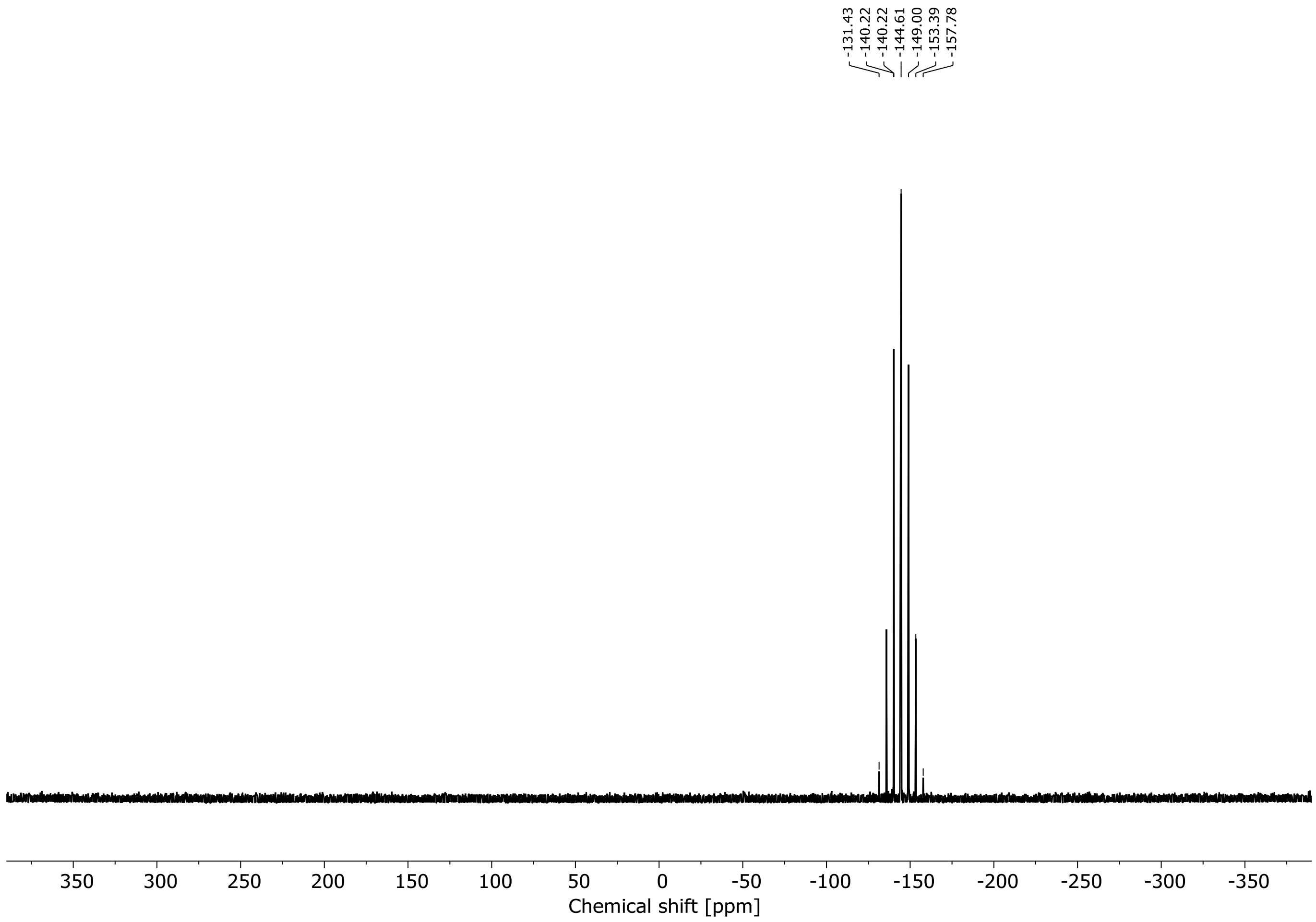

Figure S113. ^{31}P NMR spectra of the solution obtained after SLE from **M3** into **1b** solution in $CDCl_3$.

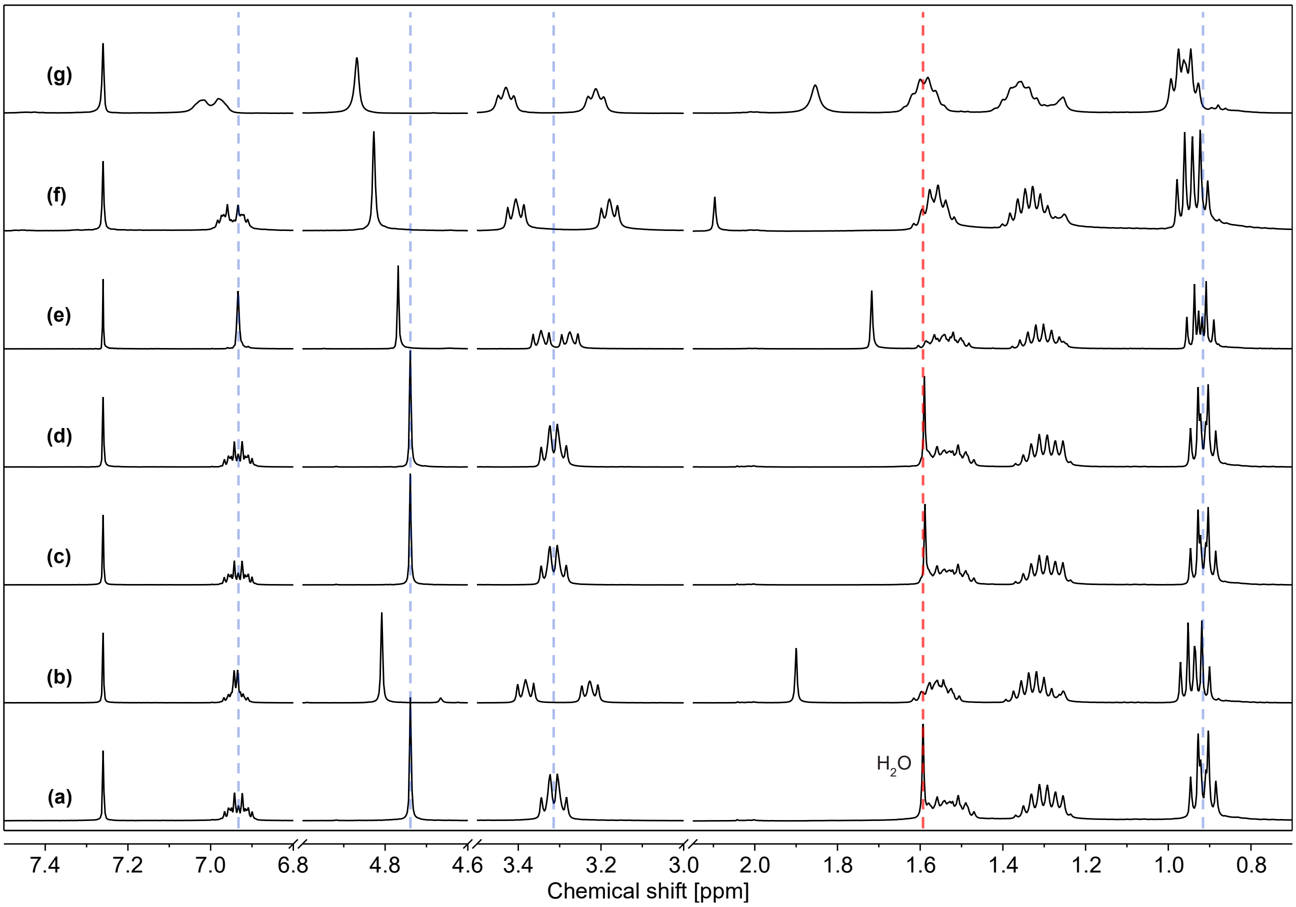

Figure S114. Selected regions of ^{1}H NMR spectra of solutions obtained after SLE into **1c** solution in $CDCl_3$, **(a)** stock **1c** solution, **(b)** $LiCl{\cdot}H_2O$, **(c)** NaCl, **(d)** KCl, **(e)** **M1**, **(f)** **M2**, **(g)** **M3**.

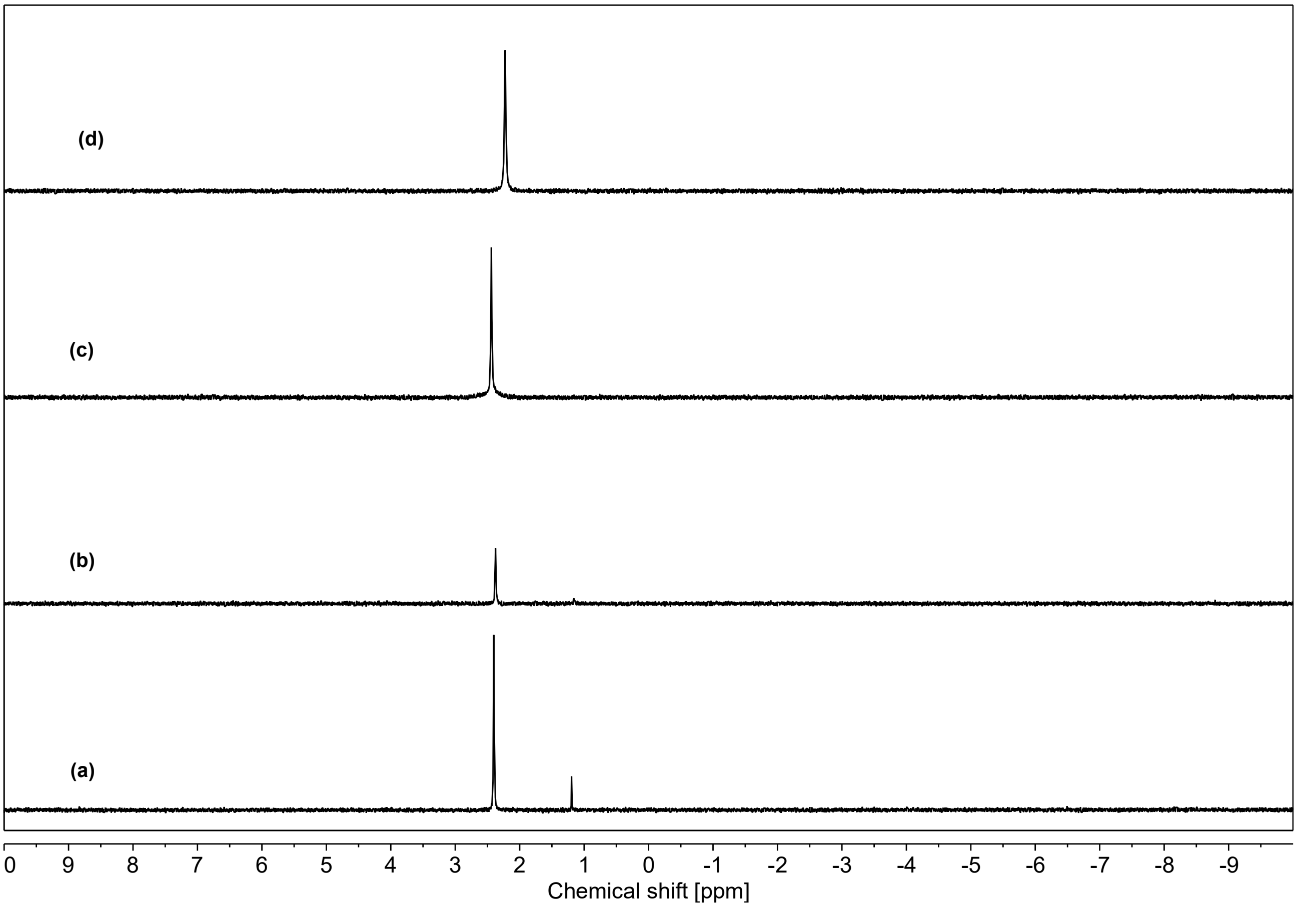

Figure S115. ^{7}Li NMR spectra of solutions obtained after SLE into **1c** solution in $CDCl_3$ **(a)** $LiCl{\cdot}H_2O$, **(b)** **M1**, **(c)** **M2**, **(d)** **M3**.

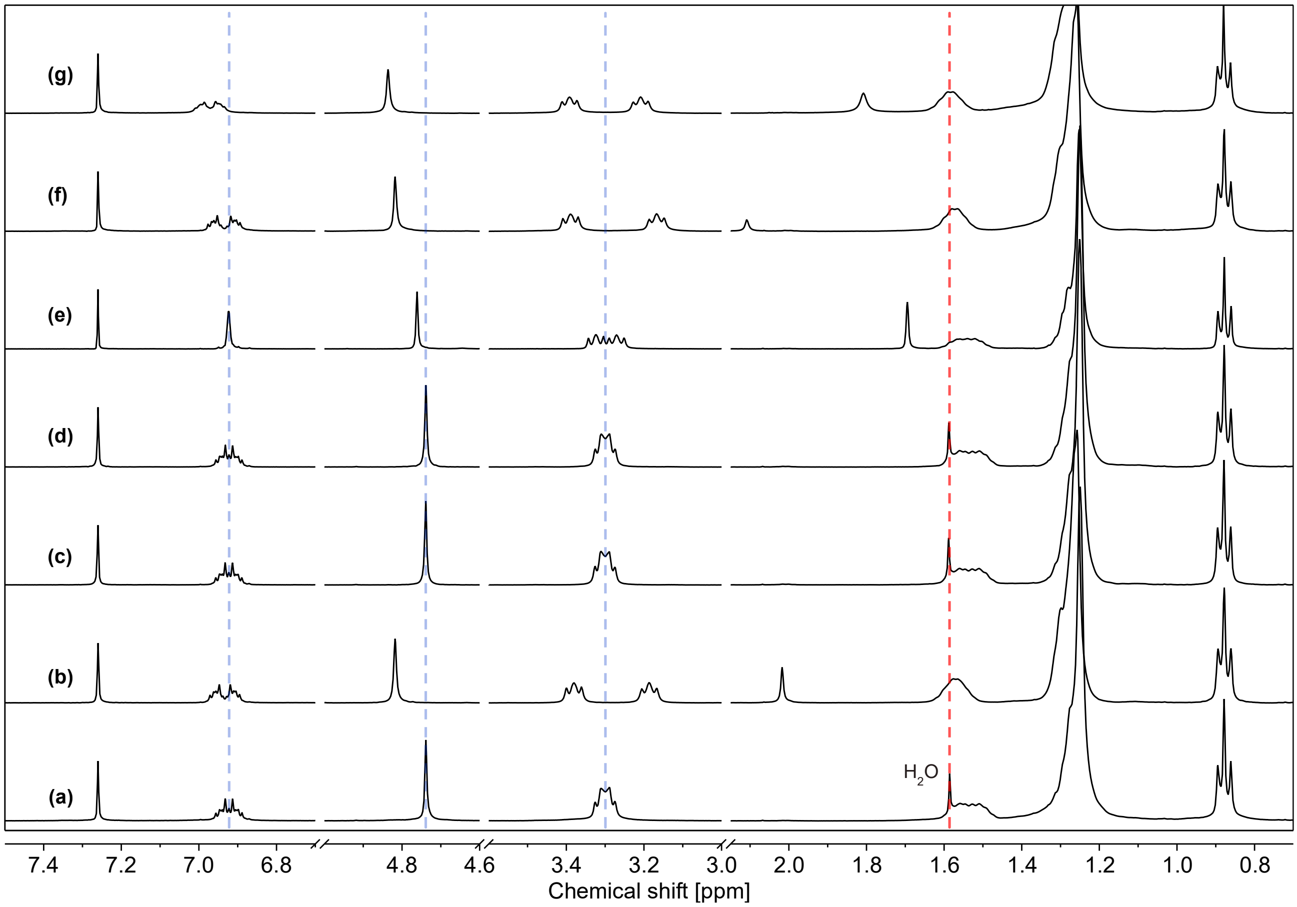

Figure S116. Selected regions of ^{1}H NMR spectra of solutions obtained after SLE into **1d** solution in $CDCl_3$, **(a)** stock **1d** solution, **(b)** $LiCl{\cdot}H_2O$, **(c)** NaCl, **(d)** KCl, **(e) M1**, **(f) M2**, **(g) M3**.

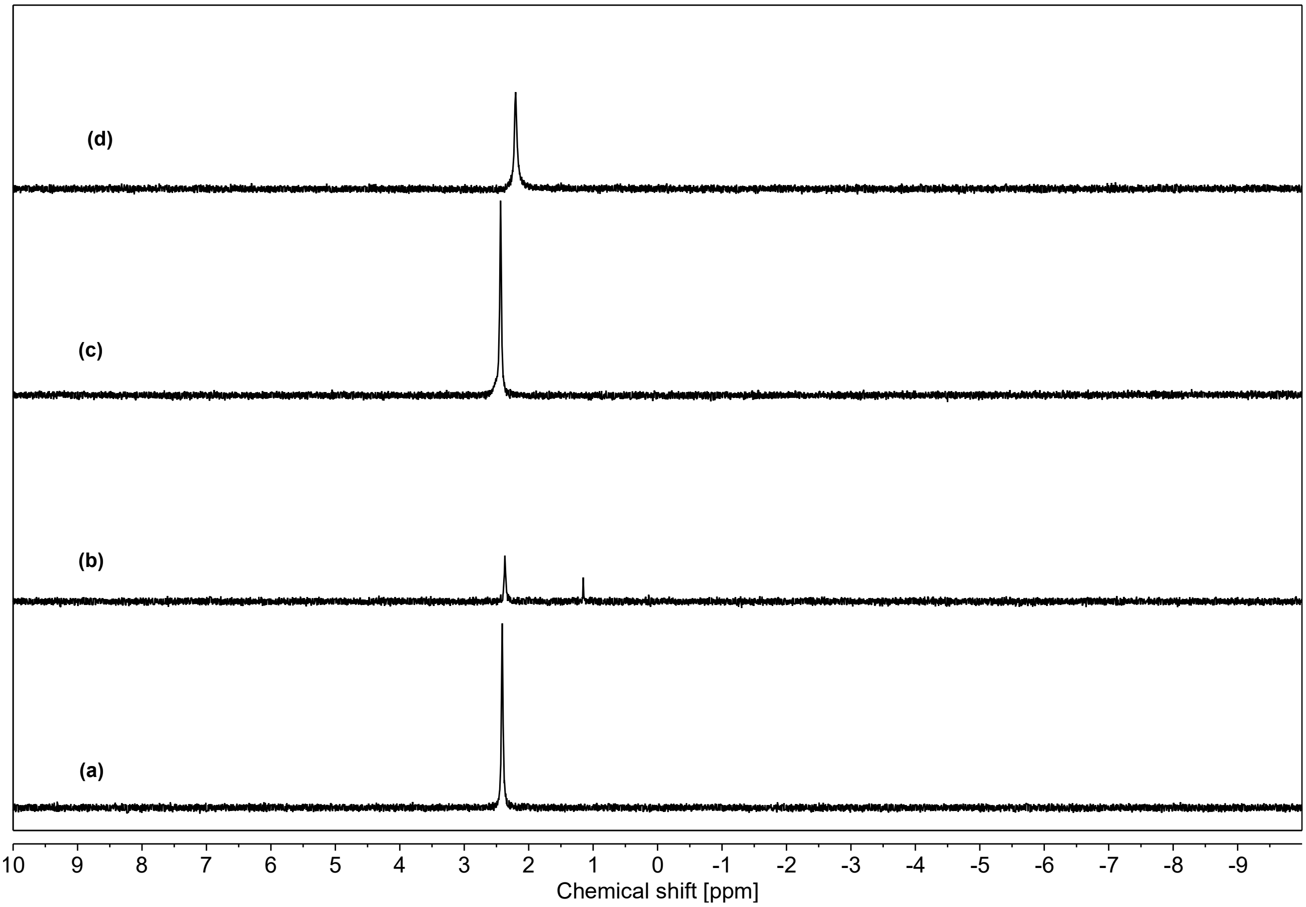

Figure S117. ^{7}Li NMR spectra of solutions obtained after SLE into **1d** solution in $CDCl_3$ **(a)** $LiCl{\cdot}H_2O$, **(b) M1**, **(c) M2**, **(d) M3**.

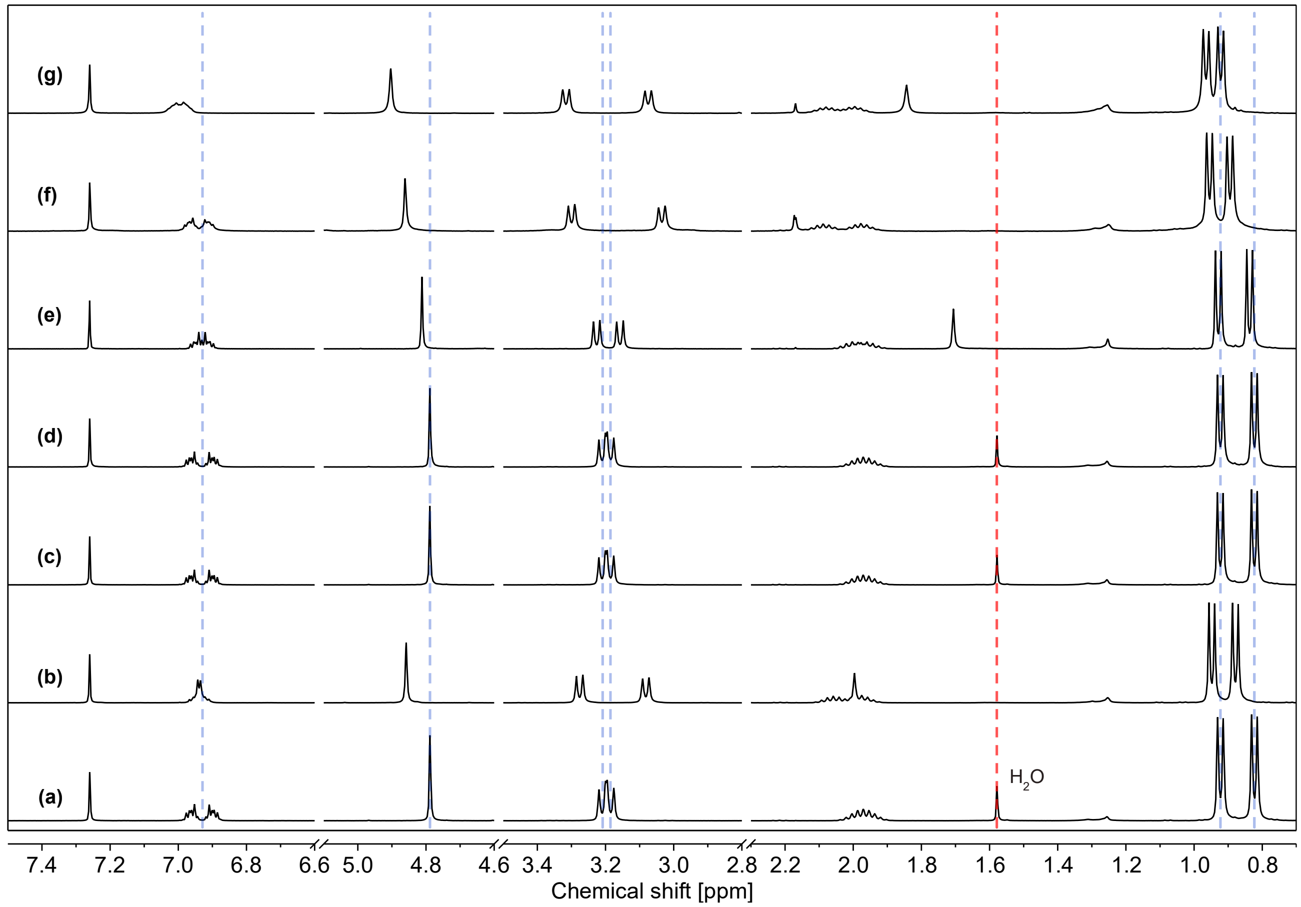

Figure S118. Selected regions of ^{1}H NMR spectra of solutions obtained after SLE into **1e** solution in $CDCl_3$, **(a)** stock **1e** solution, **(b)** $LiCl·H_2O$, **(c)** NaCl, **(d)** KCl, **(e) M1**, **(f) M2**, **(g) M3**.

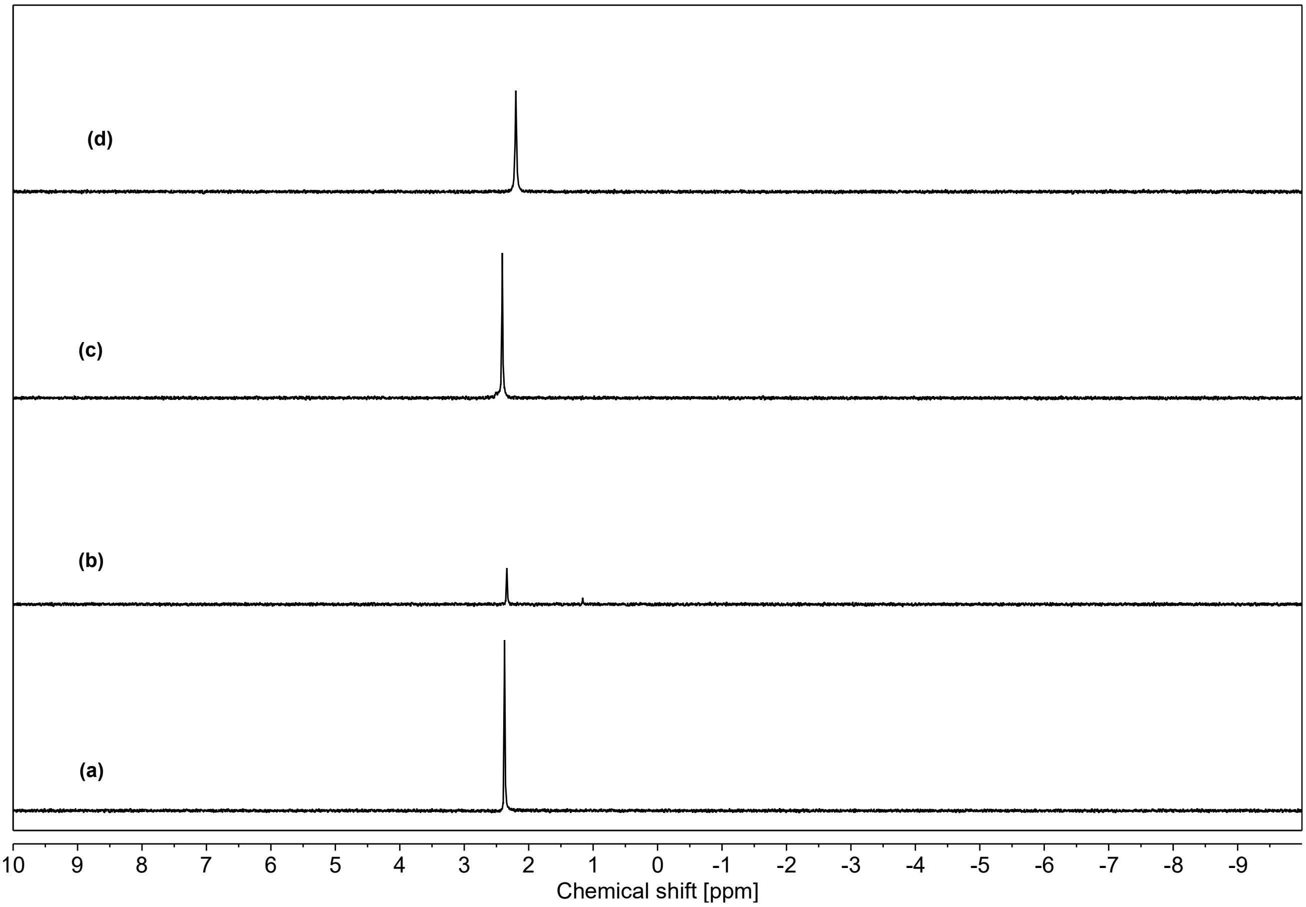

Figure S119. ^{7}Li NMR spectra of solutions obtained after SLE into **1e** solution in $CDCl_3$ **(a)** $LiCl·H_2O$, **(b) M1**, **(c) M2**, **(d) M3**.

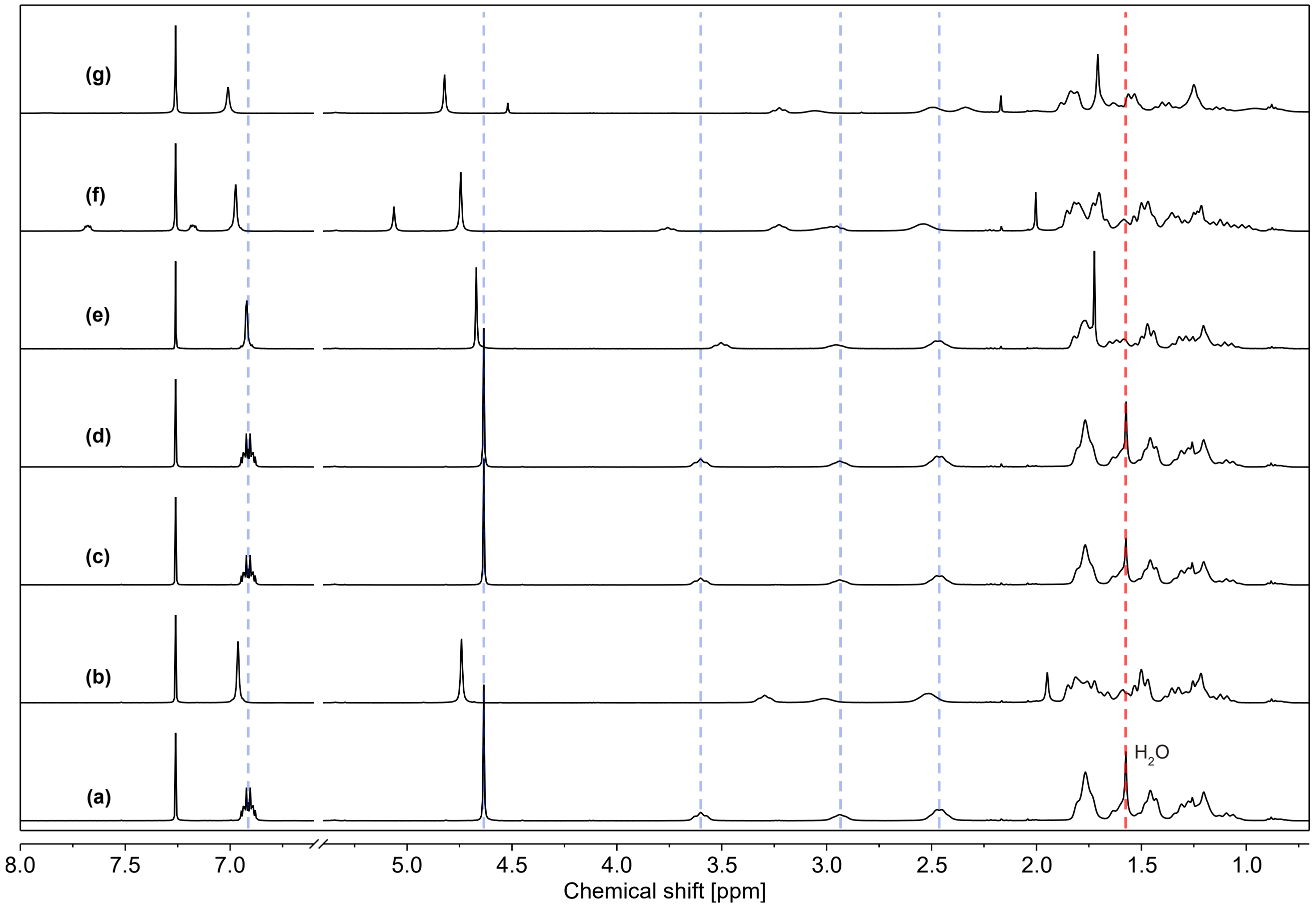

Figure S120. Selected regions of ^{1}H NMR spectra of solutions obtained after SLE into **1f** solution in $CDCl_3$, **(a)** stock **1f** solution, **(b)** $LiCl·H_2O$, **(c)** NaCl, **(d)** KCl, **(e) M1**, **(f) M2**, **(g) M3**.

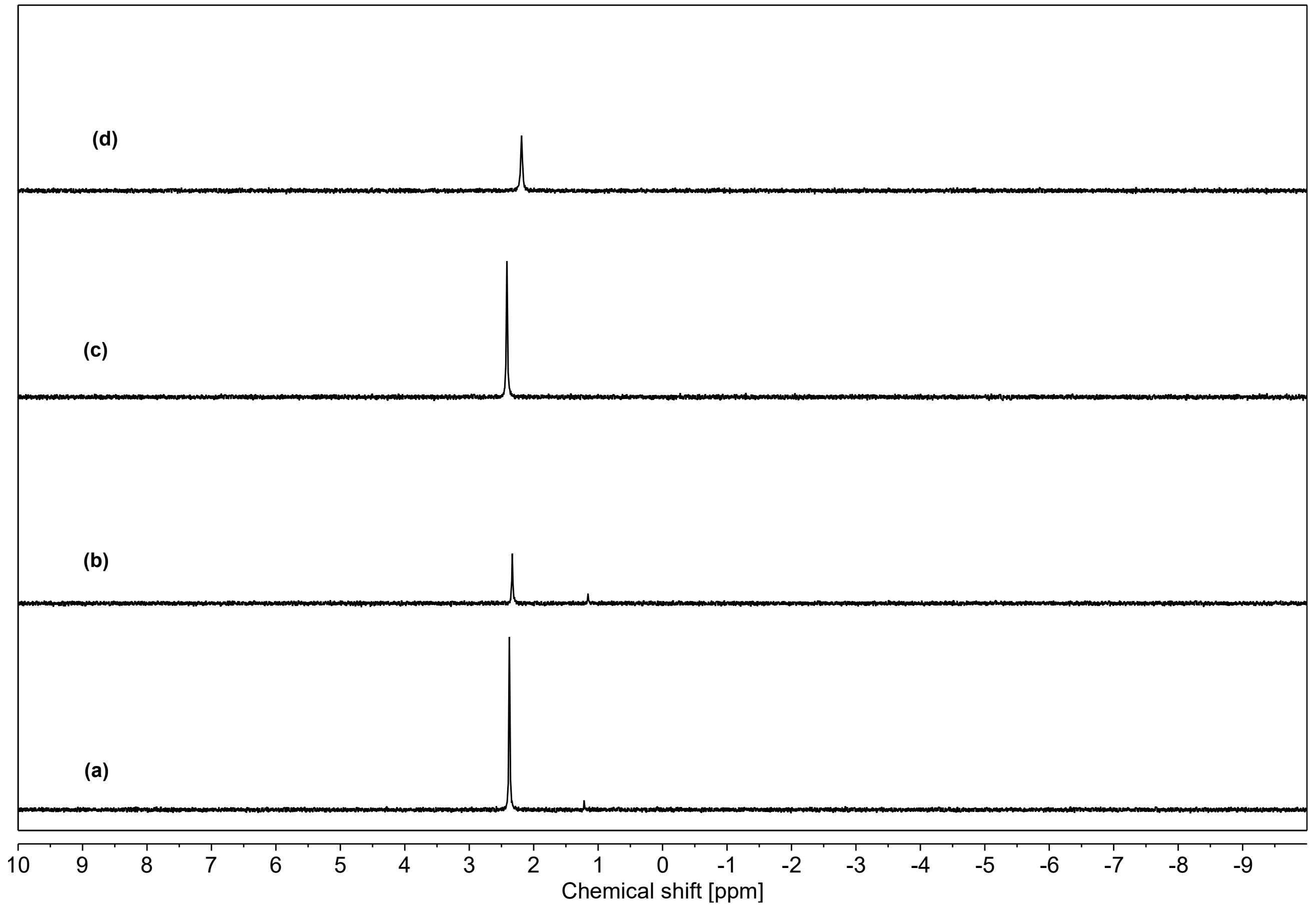

Figure S121. ^{7}Li NMR spectra of solutions obtained after SLE into **1f** solution in $CDCl_3$ **(a)** $LiCl·H_2O$, **(b) M1**, **(c) M2**, **(d) M3**.

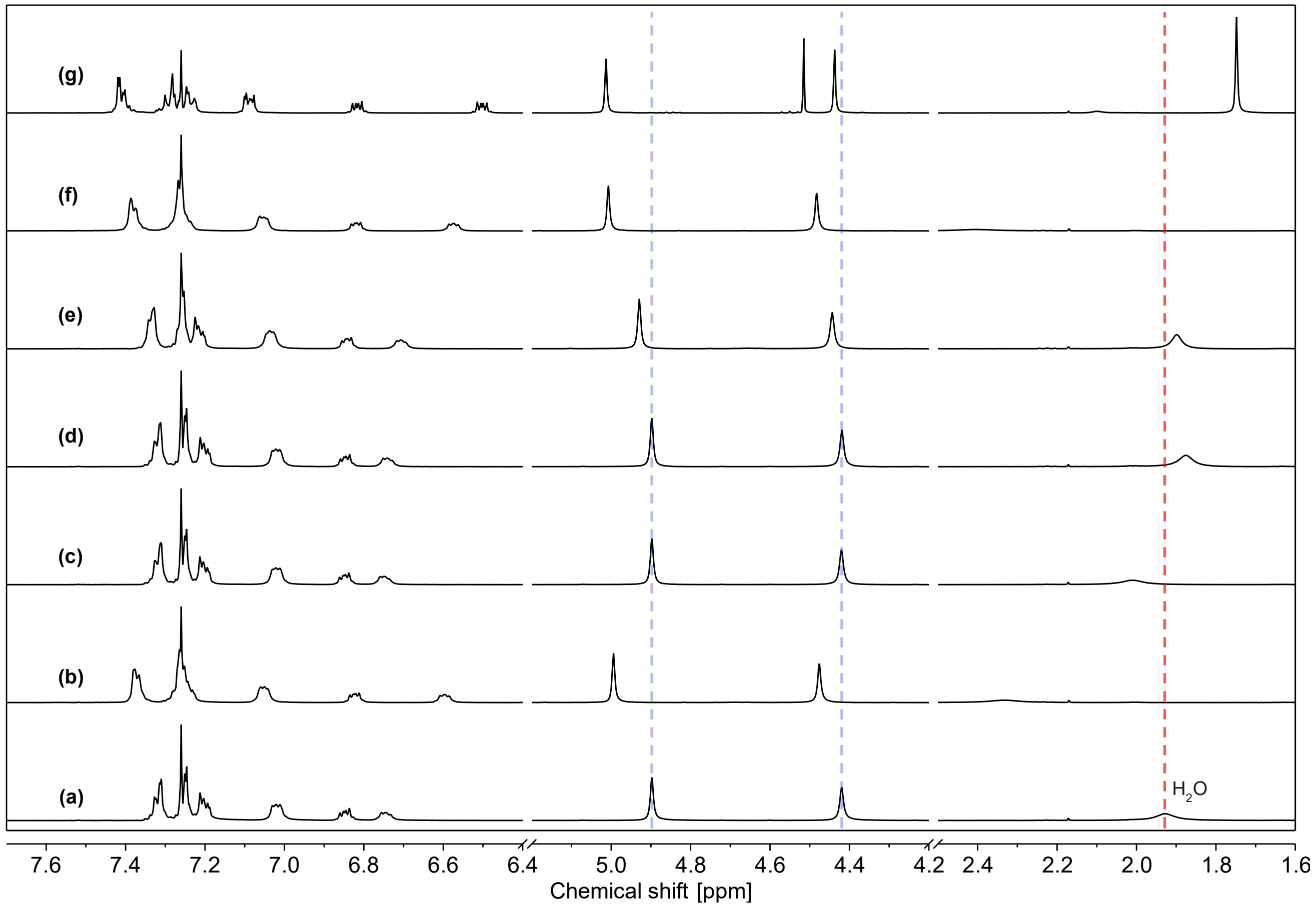

Figure S122. Selected regions of ^{1}H NMR spectra of solutions obtained after SLE into **1g** solution in $CDCl_3$, **(a)** stock **1g** solution, **(b)** $LiCl{\cdot}H_2O$, **(c)** NaCl, **(d)** KCl, **(e) M1**, **(f) M2**, **(g) M3**.

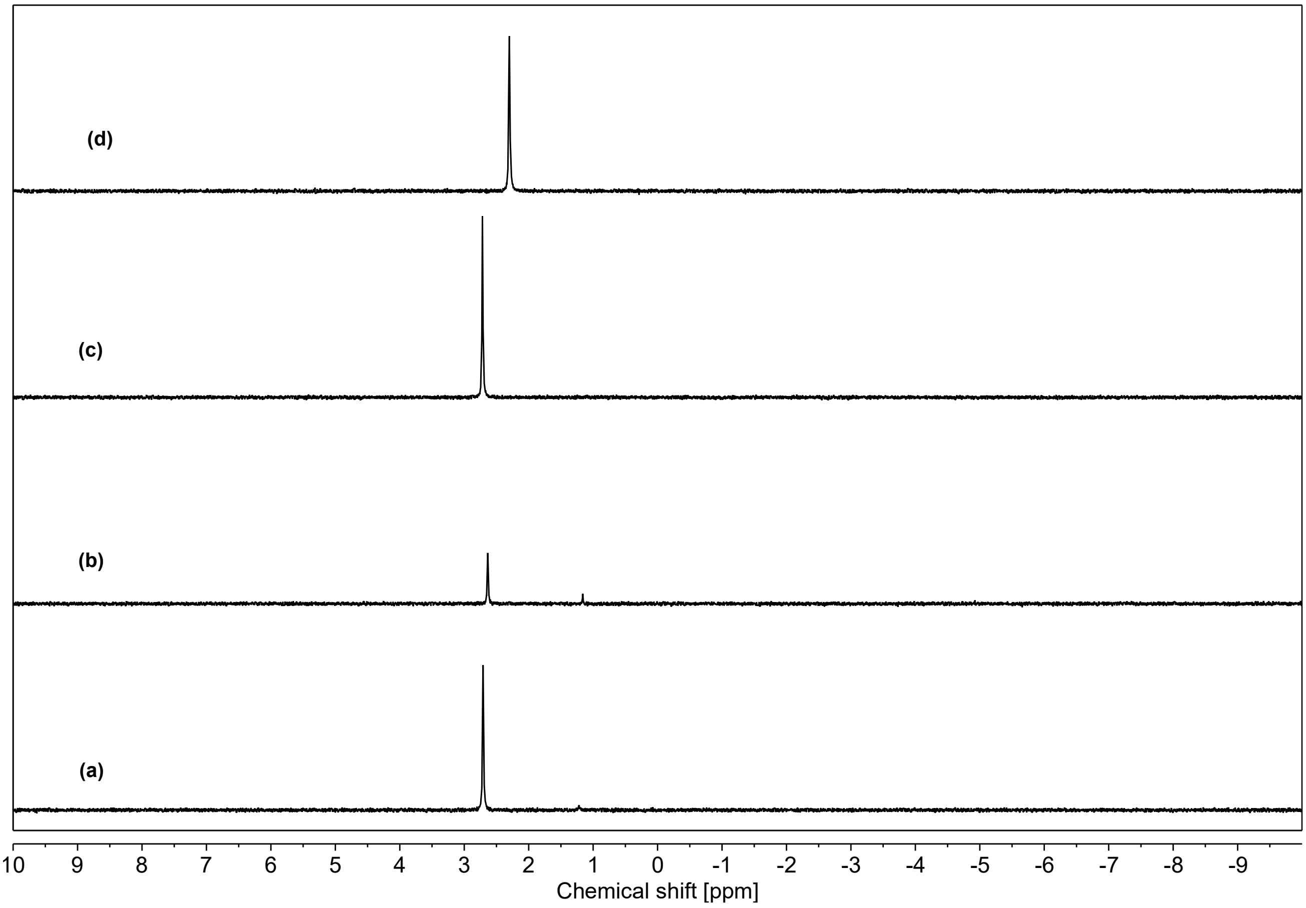

Figure S123. ^{7}Li NMR spectra of solutions obtained after SLE into **1g** solution in $CDCl_3$ **(a)** $LiCl{\cdot}H_2O$, **(b) M1**, **(c) M2**, **(d) M3**.

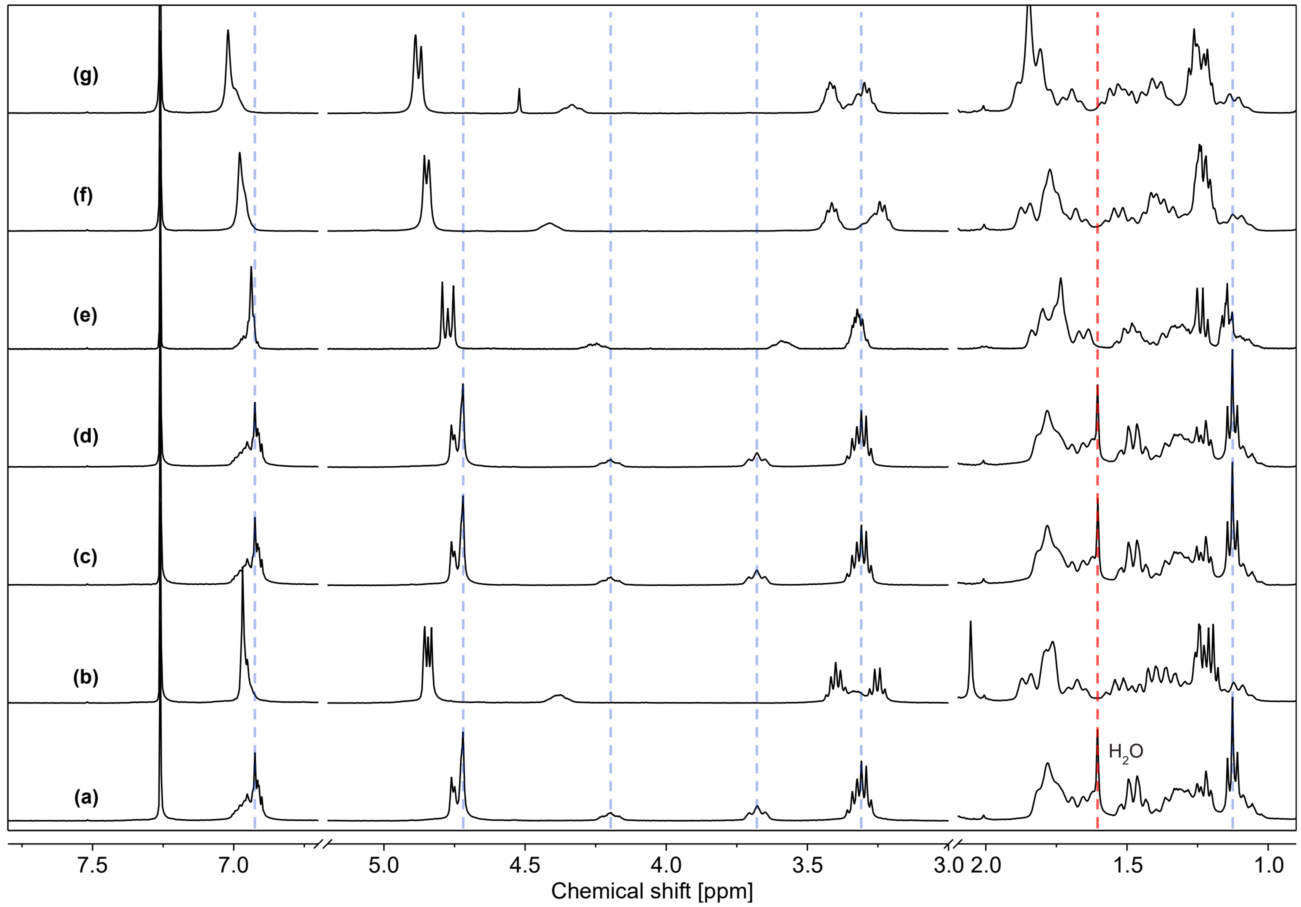

Figure S124. Selected regions of ^{1}H NMR spectra of solutions obtained after SLE into **1h** solution in $CDCl_3$, **(a)** stock **1h** solution, **(b)** $LiCl{\cdot}H_2O$, **(c)** NaCl, **(d)** KCl, **(e) M1**, **(f) M2**, **(g) M3**.

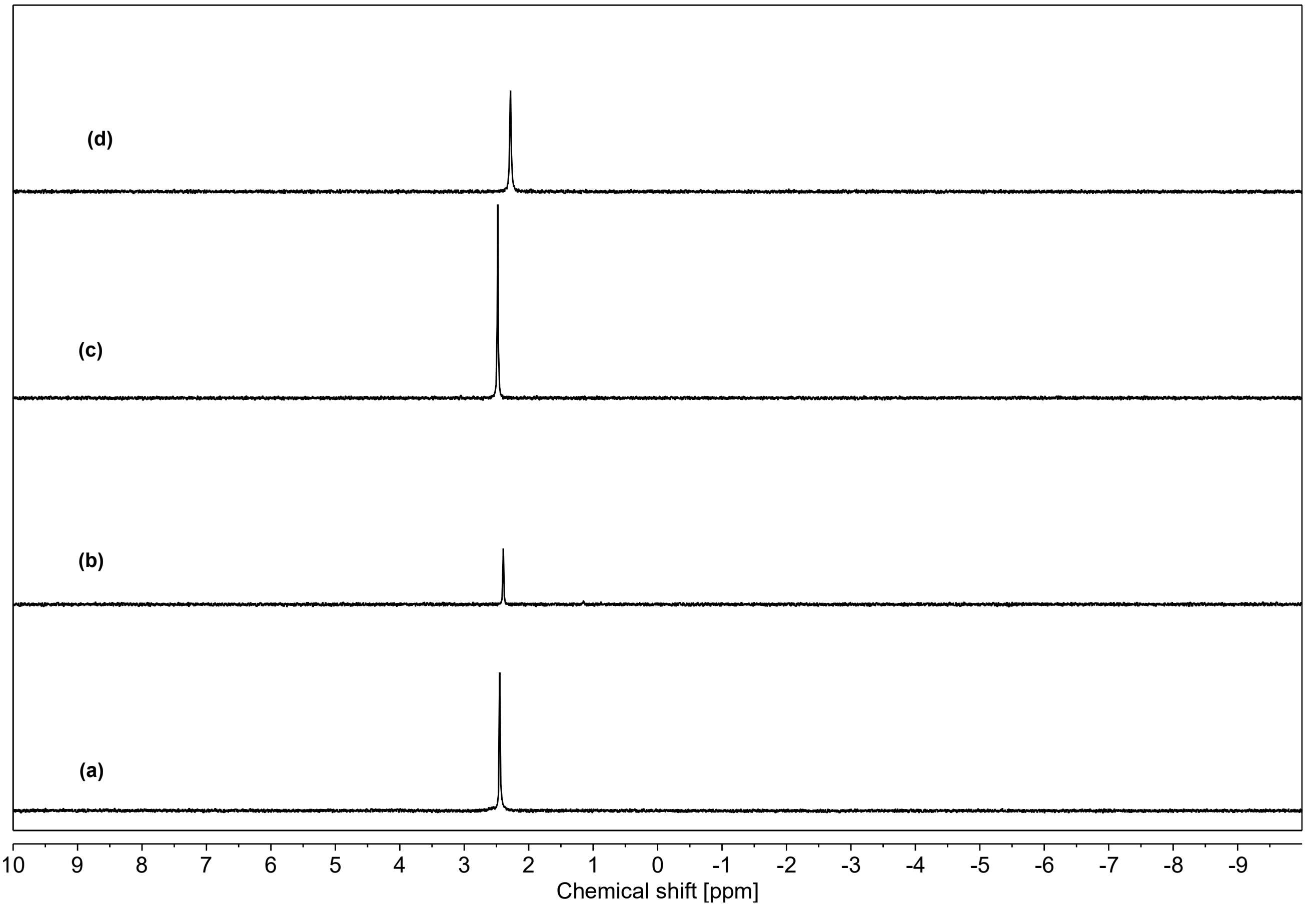

Figure S125. ^{7}Li NMR spectra of solutions obtained after SLE into **1h** solution in $CDCl_3$ **(a)** $LiCl{\cdot}H_2O$, **(b) M1**, **(c) M2**, **(d) M3**.

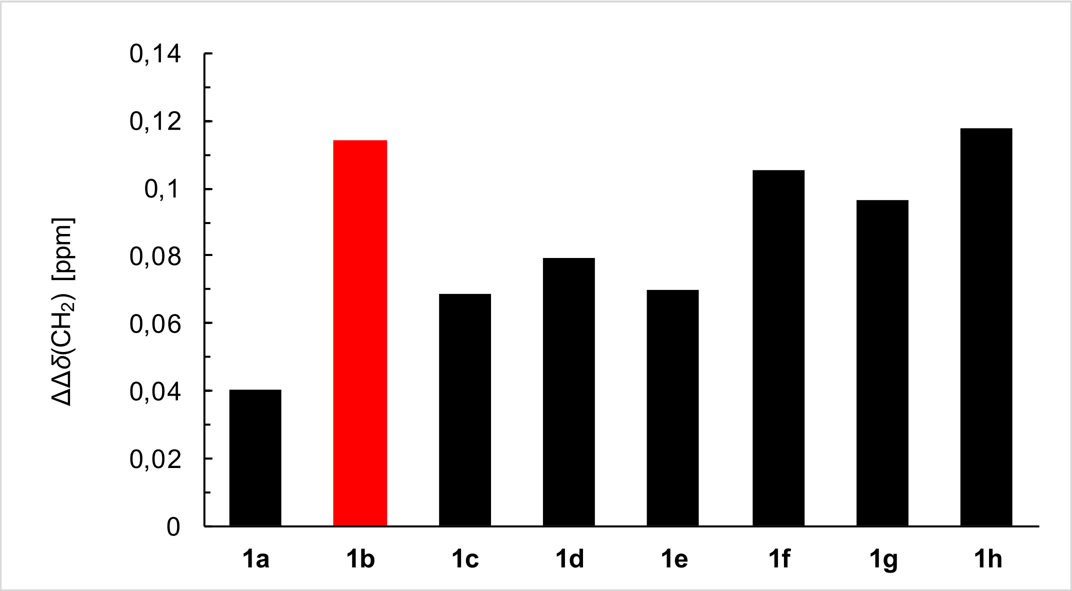

Figure S126. Comparison of ^{1}H NMR chemical-shift changes (Δδ) of methylene OCH_2 protons for **1a**–**h** after SLE of $LiCl{\cdot}H_2O$ under standard conditions (data extracted from individual SLE experiments).

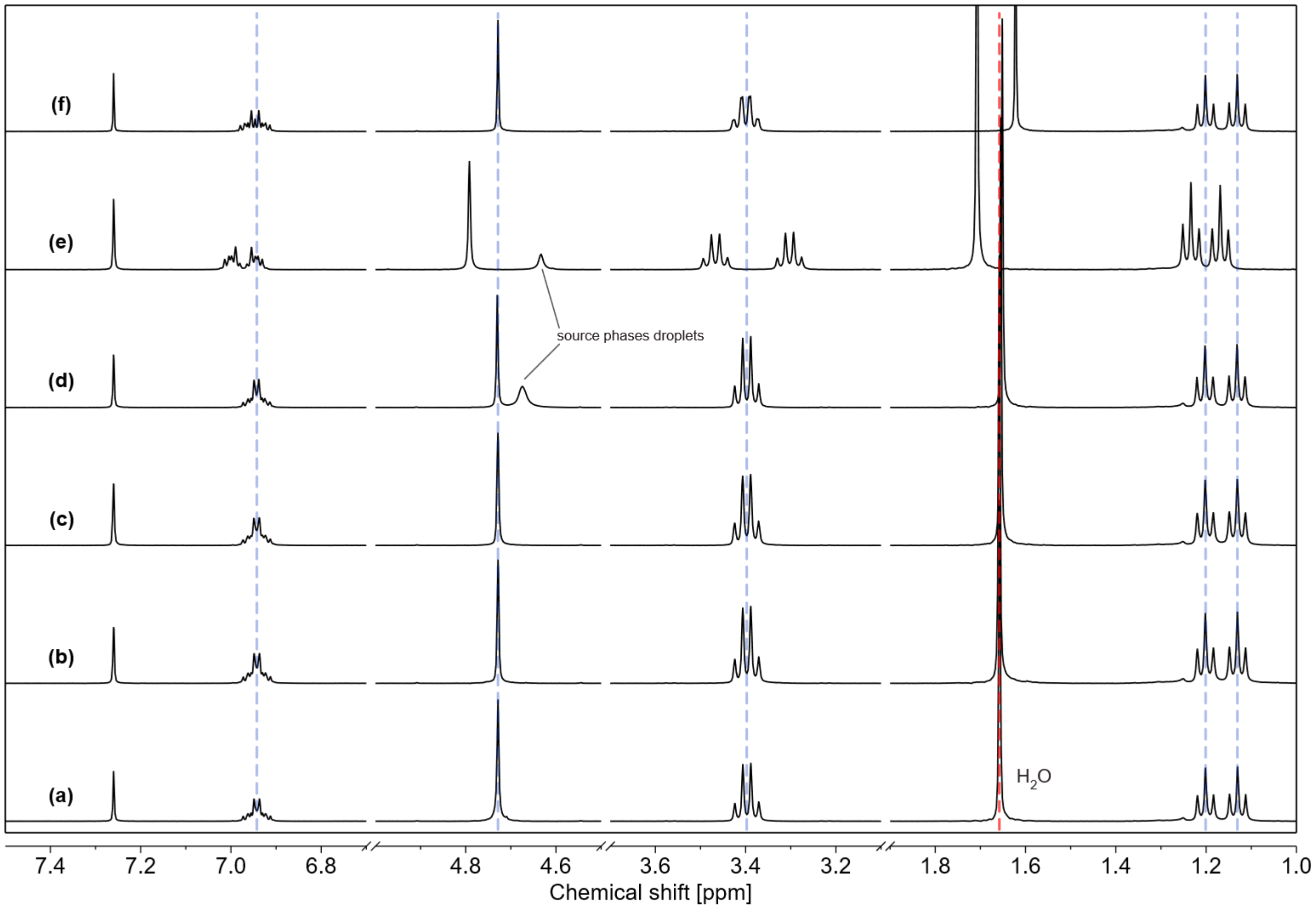

Figure S127. Selected regions of ^{1}H NMR spectra of solutions obtained after LLE into **1b** solution in $CDCl_3$, **(a)** water, **(b)** 1.0 M LiCl, **(c)** 1.0 M LiBr, **(d)** 1.0 M LiOTf, **(e)** 1.0 M $LiNTf_2$, **(f)** LiCl + NaCl + KCl + $MgCl_2$ + $CaCl_2$ (1.0 M each).

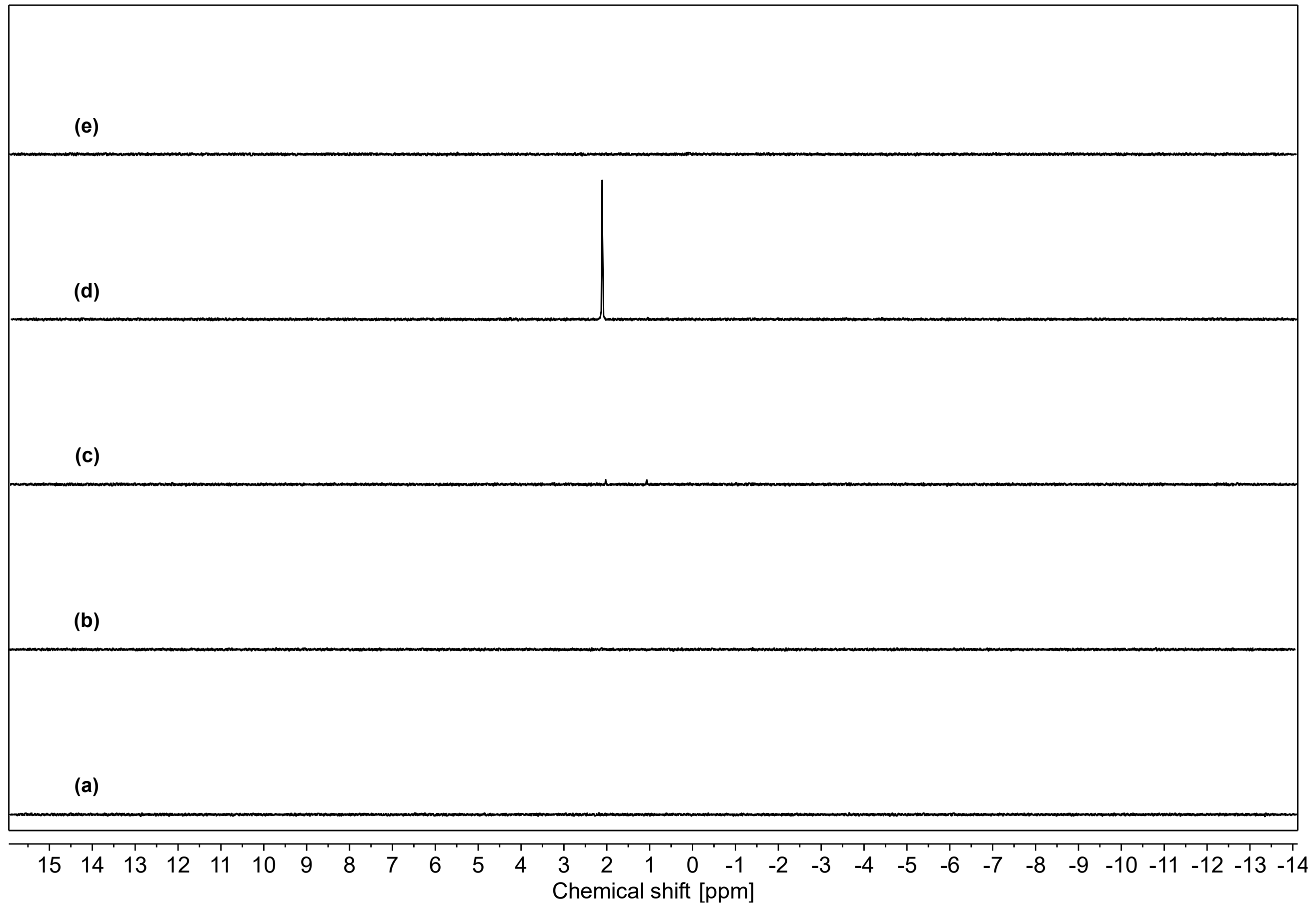

Figure S128. ^{7}Li NMR spectra of solutions obtained after LLE into **1h** solution in $CDCl_3$ **(a)** 1.0 M LiCl, **(b)** 1.0 M LiBr, **(c)** 1.0 M LiOTf, **(d)** 1.0 M $LiNTf_2$, **(e)** LiCl + NaCl + KCl + $MgCl_2$ + $CaCl_2$ (1.0 M each).

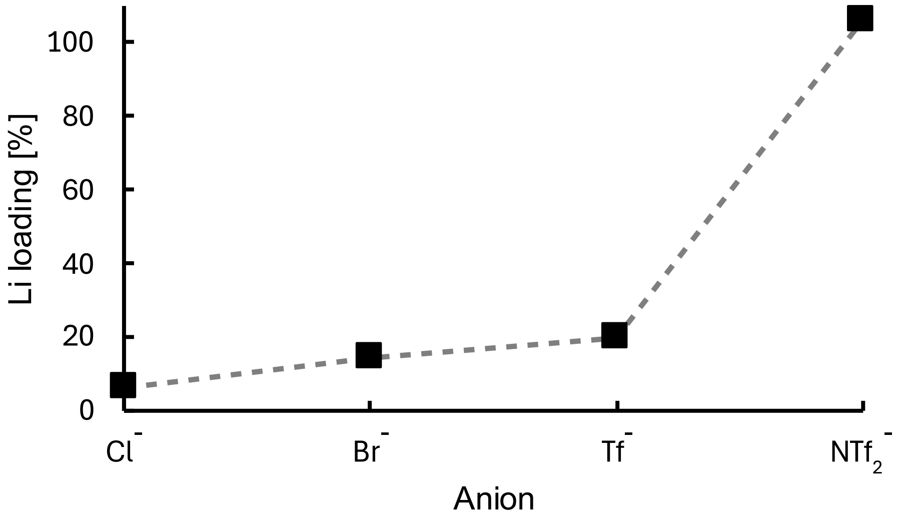

Figure S129. Lithium loading (ICP-MS/MS) of 10 mM **1b** solutions in $CDCl_3$ after LLE from 1 M aqueous solutions of LiCl, LiBr, LiOTf, and $LiNTf_2$.

4.3 DOSY ^{1}H NMR measurements

For DOSY measurements of **1b-d** and their complexes with LiCl, 0.50 mL of solutions prepared as described in section 4.2 (Protocol for SLE experiments for **1a**–**h**) were placed in NMR tubes, and DOSY spectra were recroded at 600 MHz (Figures S130-138). The obtained diffusion coefficients are given in Table S10.

Table S10. Diffusion coefficients of selected species in $CDCl_3$, 10.0 mM solutions of **1b–d** in $CDCl_3$ before and after SLE from $LiCl \cdot H_2O$, and 10.0 mM solutions of TBACl and TPPCl in $CDCl_3$.[a]

Sample	$D_{CHCl3} \cdot 10^6$ [$cm^2 \cdot s^{-1}$]	D_{H2O}[b] $\cdot 10^6$ [$cm^2 \cdot s^{-1}$]	D_{host}[c] $\cdot 10^6$ [$cm^2 \cdot s^{-1}$]
**$CDCl_3$**	24	50	-
**1b**	24	47	9.9
**1b·LiCl**	22	39	7.6
**1c**	24	41	7.7
**1c·LiCl**	23	40	6.9
**1d**	24	28	7.1
**1d·LiCl**	24	40	5.8
**TBACl**	24	44	-
**TPPCl**	23	44	-

[a] Estimated uncertainties for diffusion coefficients *D* are ±10%. [b] Water concentration was ~ 10 mM in all cases, as determined by spectra integration. [c] Determined for the peak of the $-OCH_2CO-$ group.

In the presence of the free hosts **1b**–**d**, the water signal shows reduced diffusion relative to residual water in neat $CDCl_3$, indicating host-water interaction. The magnitude of this effect increases along the series **1b** < **1c** < **1d**, consistent with increasing alkyl chain length. Despite the substantial decrease in diffusion coefficient, reaching up to ca. 40% for **1d**, the chemical shift of water peak remains essentially unchanged. Similar effects of supramolecular host–water interactions on D_{H2O} were previously observed.[39] Following extraction of $LiCl \cdot H_2O$, the water signal shifts markedly downfield and still exhibits reduced diffusion, with D_{H2O} values remaining ca. 20% lower than for residual water in neat $CDCl_3$, while interaction with Cl^- (TBACl and TPPCl) alone caused only ca. 10% decrease in D_{H2O} compared to neat $CDCl_3$. This behavior is consistent with labile, yet persistent association of water with the extracted supramolecular assembly on the NMR timescale, supporting a solution-state picture in which water remains an integral component of the complex. Notably, the diffusion coefficients of water in the extracted complexes are similar across the **1b**–**d** series. This likely reflects two opposing effects: the increasing molecular weight of the extractant, which would tend to slow diffusion, and changes in the balance between contact ion pairs (CIP, Li-Cl-H_2O) and water-bridged (Li-OH_2-Cl) ion pairs. X-ray data for **1b**·Li·OH_2·Cl, representing a water-bridged ion pair (Figure S101), and **1c**·LiCl·H_2O (Figure S103), representing a contact ion pair, together with DFT calculations at the M06-2X/def2-TZVP/CPCM($CHCl_3$) level (Figure S151), indicate a slight energetic preference for the CIP motif. However, the calculated energy differences are small ($\leq$ 4 $kJ \cdot mol^{-1}$) and within the uncertainty of the DFT method, suggesting that both motifs may coexist and interconvert rapidly in solution. The CIP arrangement is expected to permit faster water exchange, whereas the water-bridged ion pair should retain water more strongly. The interplay of these effects likely accounts for the similar diffusion coefficients observed across the series.

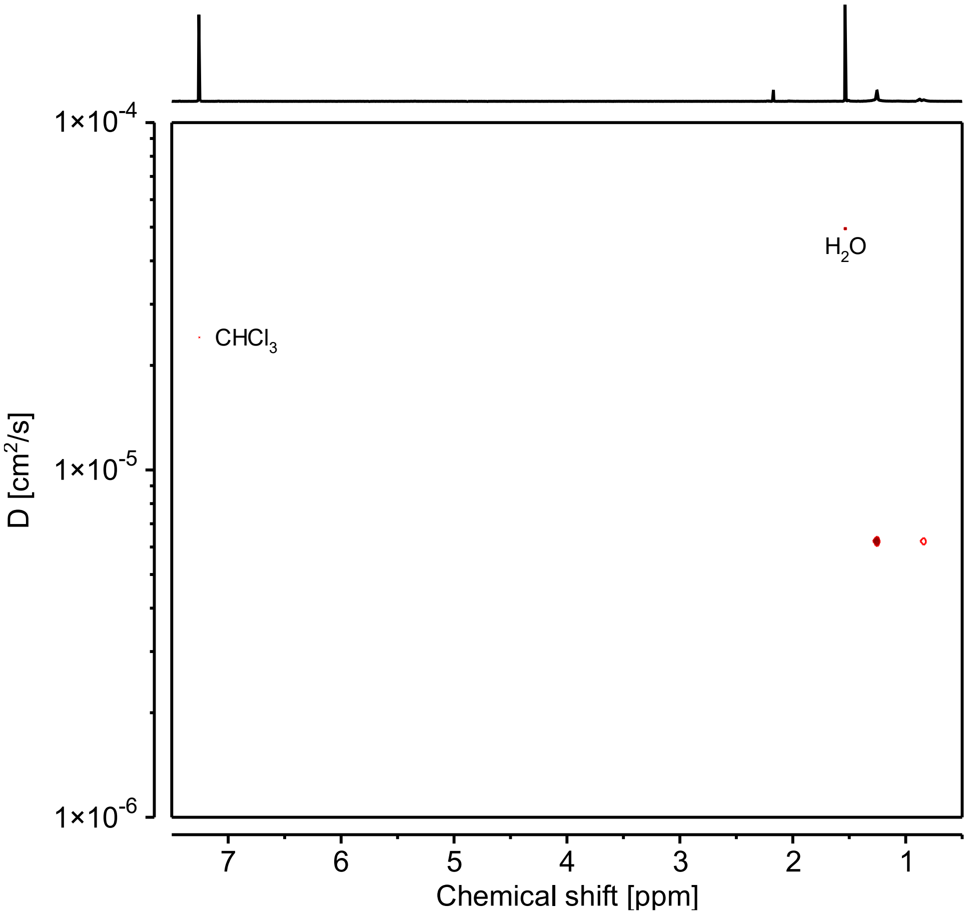

Figure S130. DOSY ^{1}H NMR spectra of $CDCl_3$ used for DOSY experiments.

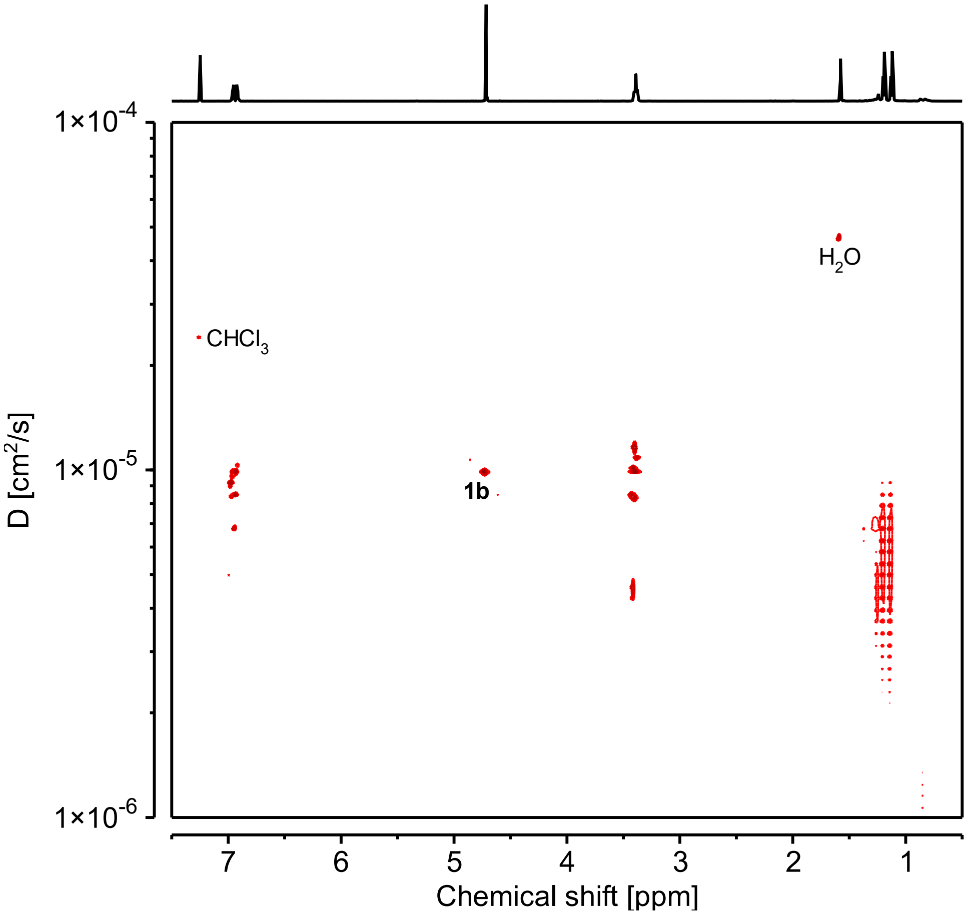

Figure S131. DOSY ^{1}H NMR spectra of 10.0 mM solution of **1b** in $CDCl_3$.

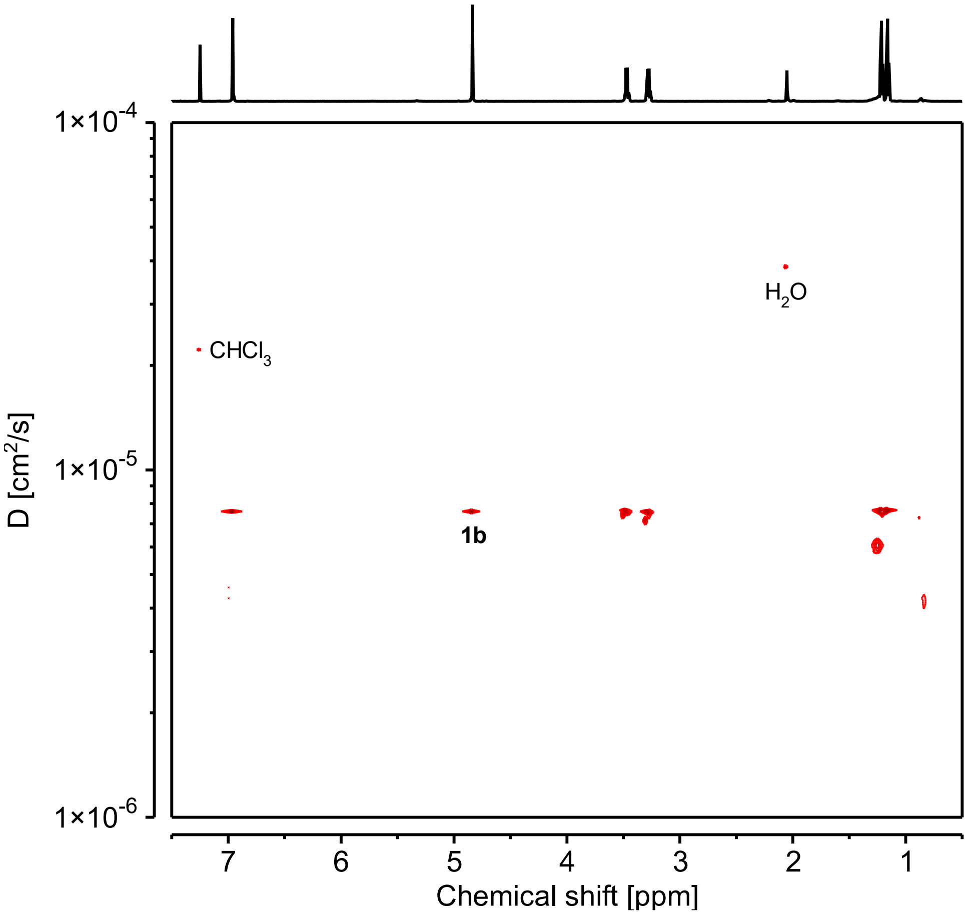

Figure S132. DOSY ^{1}H NMR spectra of 10.0 mM solution of **1b** in $CDCl_3$ after SLE from $LiCl \cdot H_2O$.

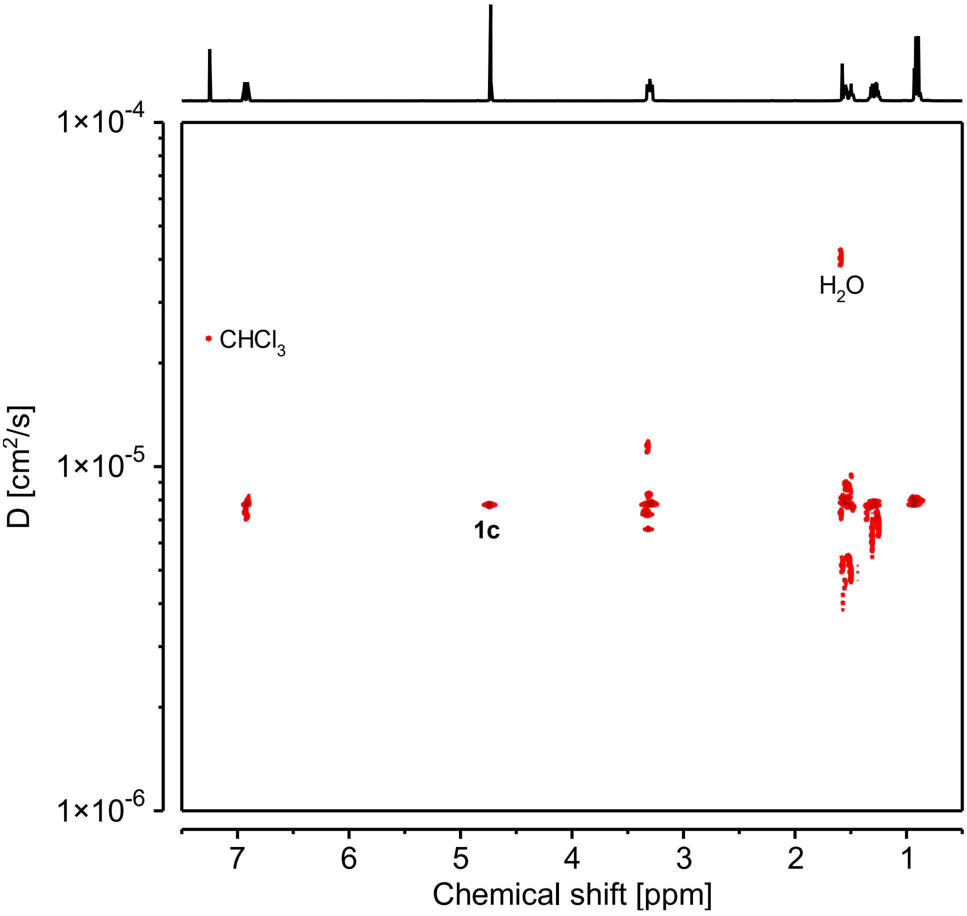

Figure S133. DOSY ^{1}H NMR spectra of 10.0 mM solution of **1c** in $CDCl_3$.

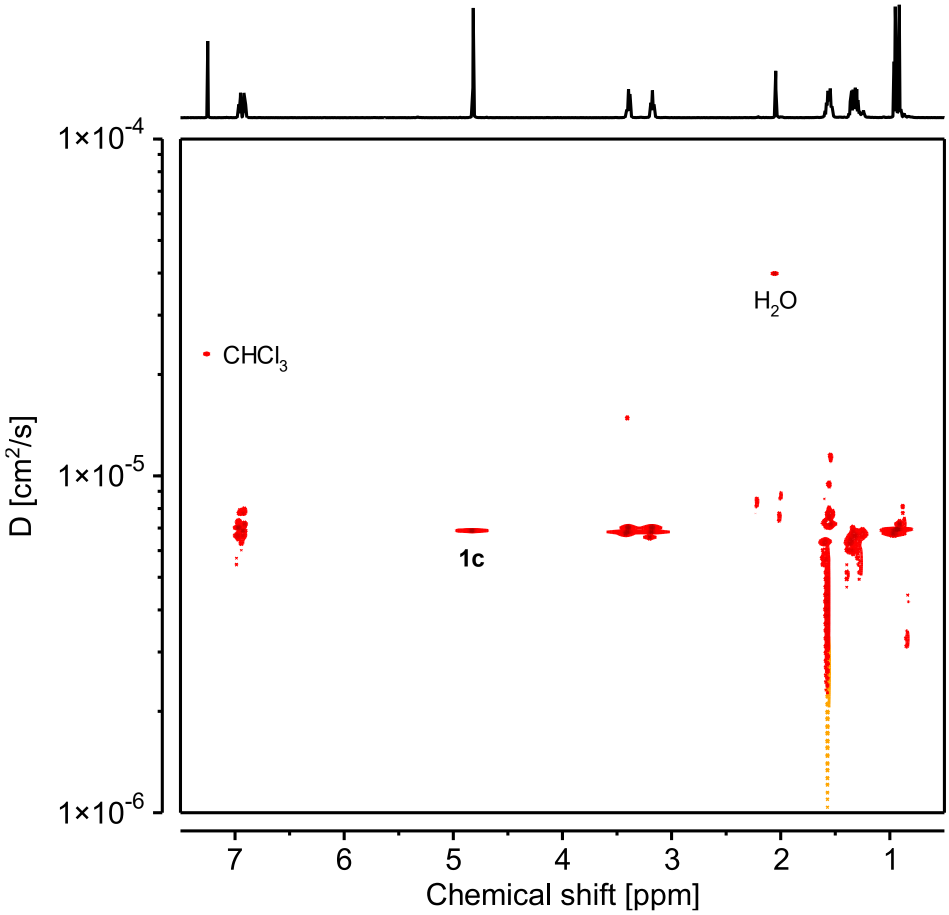

Figure S134. DOSY ^{1}H NMR spectra of 10.0 mM solution of **1c** in $CDCl_3$ after SLE from $LiCl{\cdot}H_2O$.

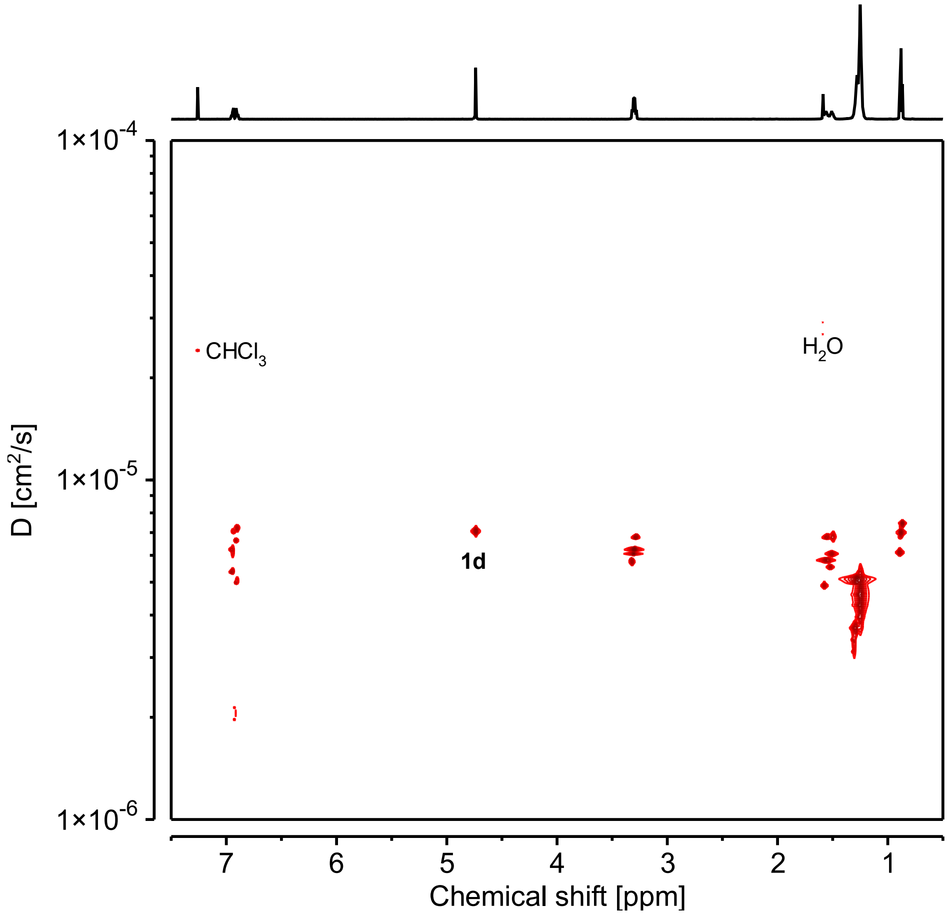

Figure S135. DOSY ^{1}H NMR spectra of 10.0 mM solution of **1d** in $CDCl_3$.

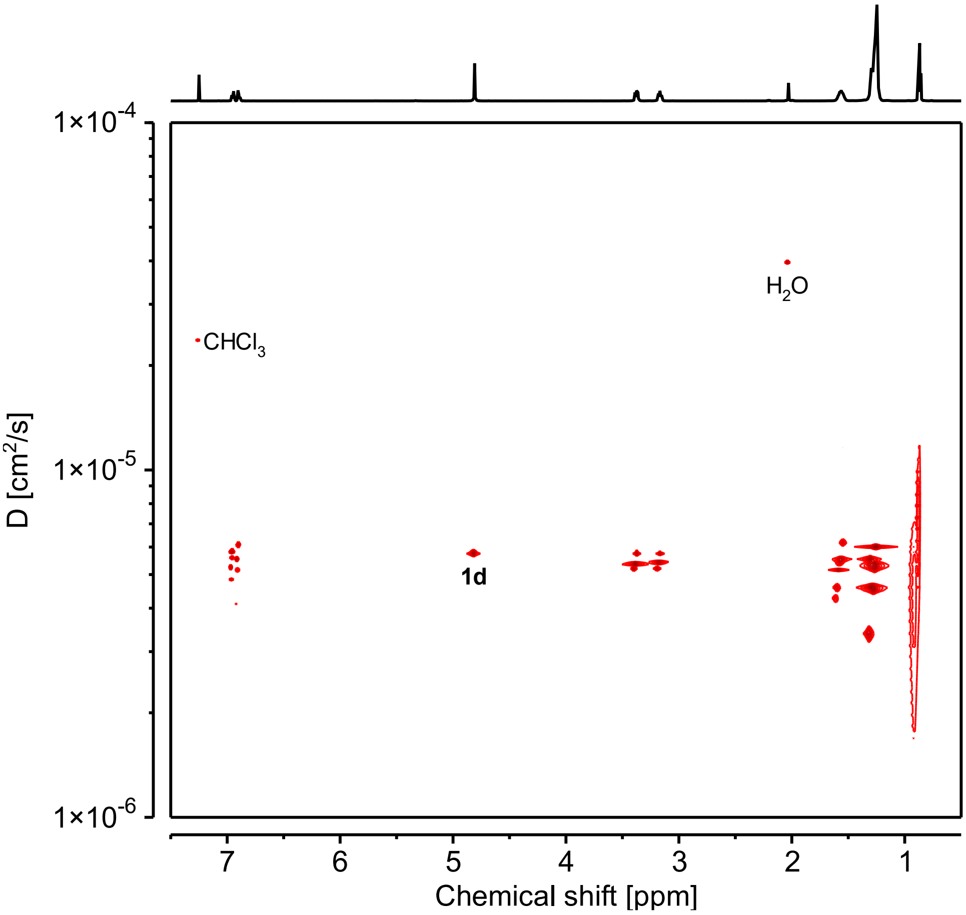

Figure S136. DOSY ^{1}H NMR spectra of 10.0 mM solution of **1d** in $CDCl_3$ after SLE from $LiCl{\cdot}H_2O$.

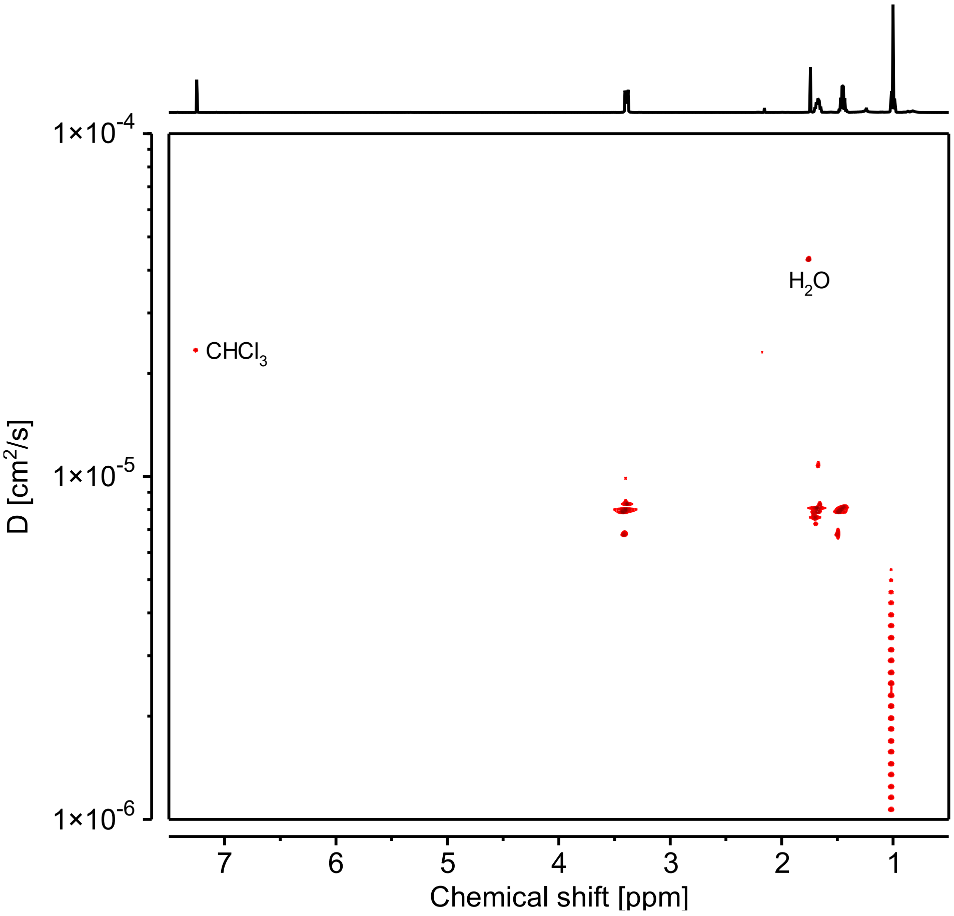

Figure S137. DOSY ^{1}H NMR spectra of 10.0 mM solution of TBACl in $CDCl_3$.

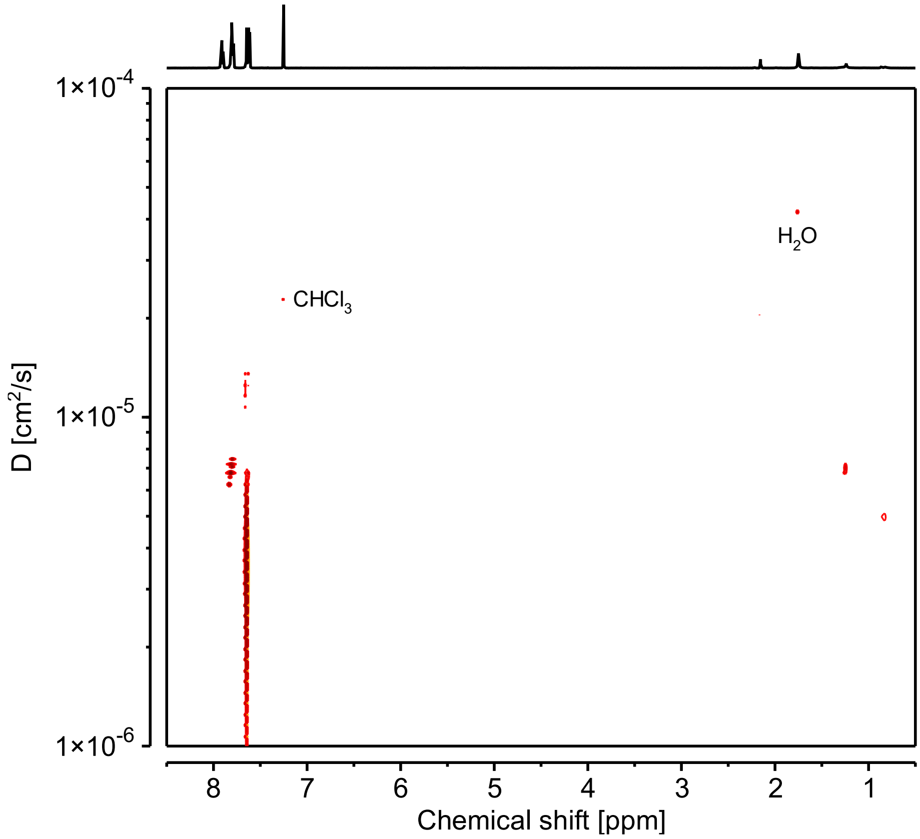

Figure S138. DOSY ^{1}H NMR spectra of 10.0 mM solution of TPPCl in $CDCl_3$.

4.4 Kinetics of $LiCl{\cdot}H_2O$ solubilization by 1b

Six 2 mL vials were loaded with 4.2 mg (0.070 mmol, 10 equiv) of $LiCl{\cdot}H_2O$. Then, 0.7 mL of a 10.0 mM solution of **1b** in deacidified $CDCl_3$ was simultaneously added to all vials, and mixing (40 rpm, SU1500 Sunlab LCD Disk Rotator) was turned on. After 5, 15, 30, 60, and 240 minutes, individual vials were taken off the rotator, filtered via a 0.45 µm PTFE syringe into NMR tubes, and the spectra were recorded (Figure S139). As a 0-minute datapoint, a 10.0 mM solution of **1b** in deacidified $CDCl_3$ not contacted with $LiCl{\cdot}H_2O$ was used.

(A)

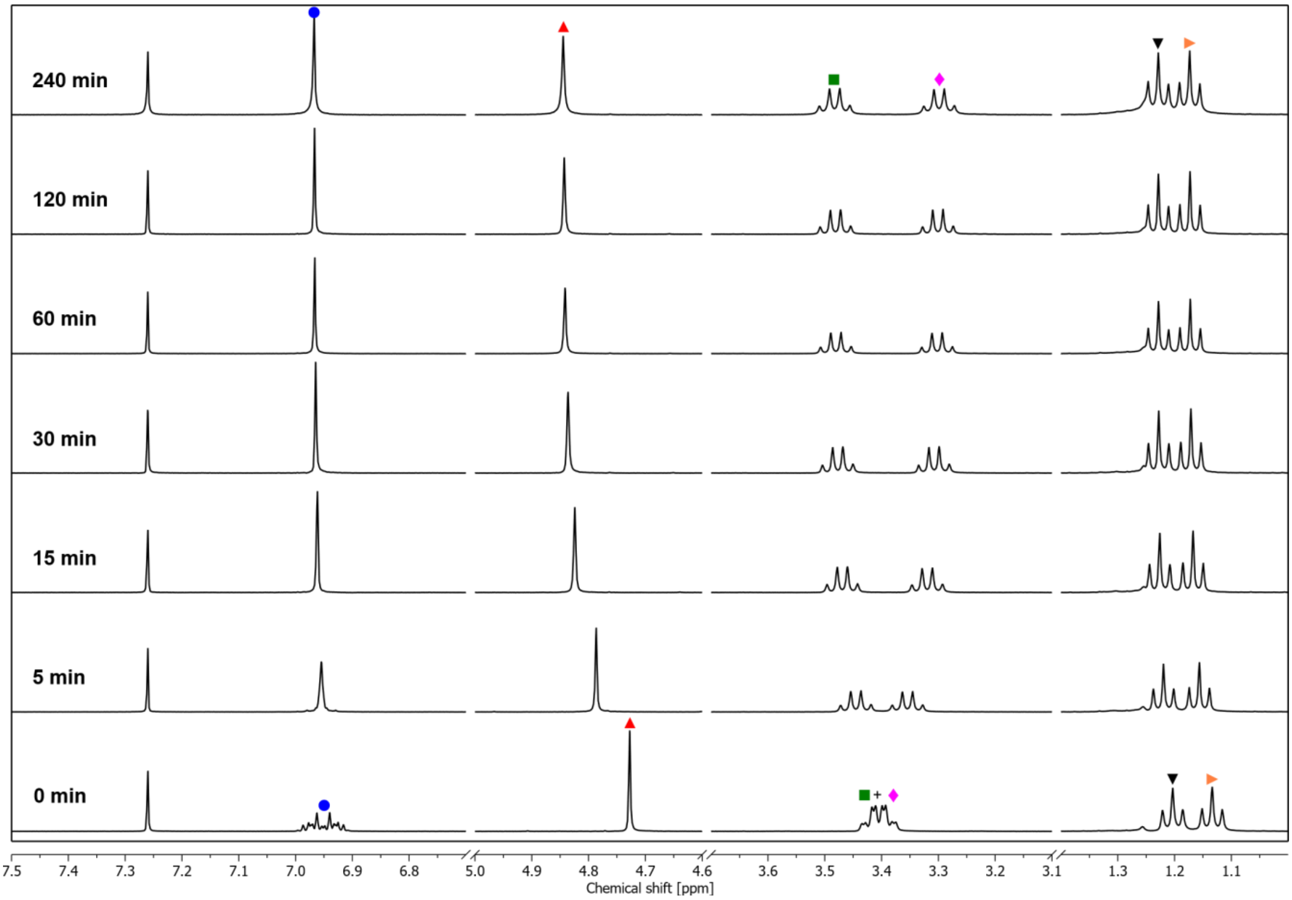

(B)

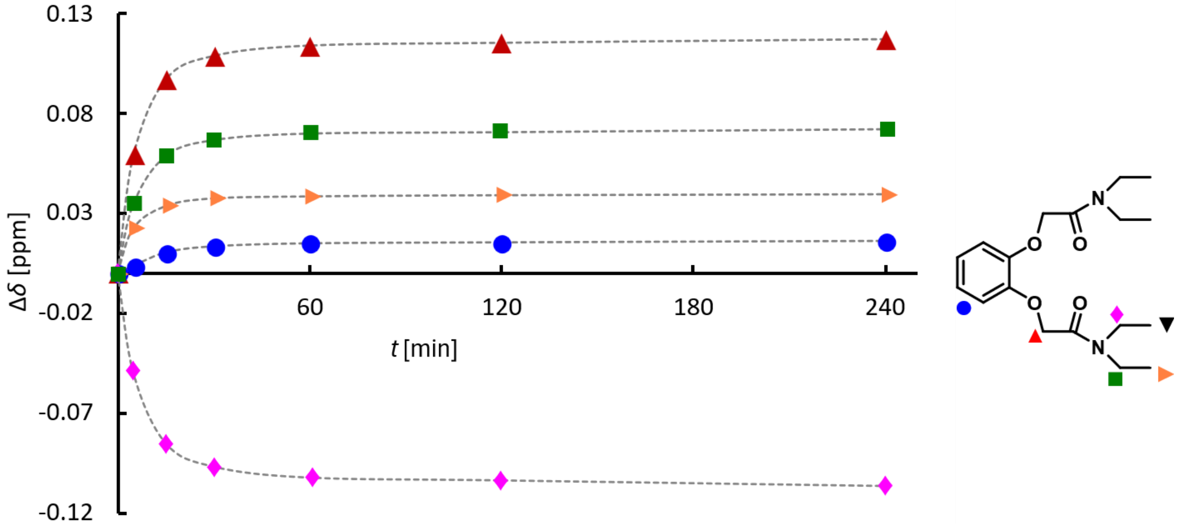

Figure S139. (A) Selected regions of ^{1}H NMR spectra of solutions obtained after SLE of $LiCl \cdot H_2O$ into **1b** in $CDCl_3$ at different extraction times at 298 K. (B) Kinetic plot of ^{1}H NMR chemical-shift changes for SLE of $LiCl \cdot H_2O$ into **1b** in $CDCl_3$.

4.5 ICP-MS/MS analysis of samples after analytical scale SLE with 1b

$CDCl_3$ solutions (0.5 mL each) after SLE with **1b** from $LiCl{\cdot}H_2O$, NaCl, KCl, **M1**, **M2**, and **M3**, or (1.0 mL each) after LLE with **1b** from 1 M aqueous LiCl, LiBr, LiOTf, and $LiNTf_2$ were back-extracted to distilled water (2 mL), diluted adequately (from 5- to 10000-times) in 2% (v/v) nitric acid (69%, Sigma-Aldrich, trace metal basis), and yttrium as internal standard was added (2 μg/L final Y concentration, Merck). As a control experiments, SLE from **M2** and LLE from 1 M aqueous $LiNTf_2$ solution were performed with sole $CDCl_3$, and the obtained solutions were processed as described above. The concentrations of lithium, sodium, magnesium, and potassium in control sample and samples after SLE from $LiCl{\cdot}H_2O$, **M1**, **M2**, and, additionally, aluminium, manganese, iron, cobalt, nickel, and copper in sample after SLE from **M3** were determined using an inductively coupled plasma tandem mass spectrometer working as an element-specific detector (each sample was measured in 20 repetitions). The Agilent 8900 ICP Triple Quadrupole Mass Spectrometer was equipped with a 2.5 mm quartz torch and the Pt-cones in the interface. The position of the torch and the nebulizer gas flow were adjusted daily, with emphasis paid to the increase in the signal-to-noise ratio using a 1 μg/L solution of Co, Y, Ce, and Tl in 2% (v/v) HNO_3 and 2% (v/v) HNO_3, respectively. The RF power was 1430 W, the nebulizer gas flow was 1.07 L/min, and the reaction gas flow (hydrogen in ICP-MS/MS) was 5.5 mL/min. The total concentrations of selected metals in measured samples were calculated automatically as a result of monitoring the singly-positively charged ions with the specified mass/charge ratios: 7 (^{7}Li), 23 (^{23}Na), 24(^{24}Mg), 39 (^{39}K), 27 (^{27}Al), 55 (^{55}Mn), 56 (^{56}Fe), 59 (^{59}Co), 60 (^{60}Ni), 63 (^{63}Cu), registered in the on-mass mode after the production in the collision-reaction cell, and normalization (^{89}Y) after daily external calibration against 15-point calibration curve (0–800 μg/L, R^2 > 0.9995). The obtained limits of detection of analytes were: Li 0.0526, Na 0.6144, Mg 0.0578, K 0.2571, Al 0.0667, Mn 0.0031, Fe 0.0373, Co 0.0009, Ni 0.0087, Cu 0.0187 [μg/L]. The elements' concentrations in liquid samples were calculated by multiplying the values generated in the software by the samples' dilution factor.

Table S11. Loading [%] of **1b** with lithium, sodium, potassium, and magnesium, measured with ICP-MS/MS from samples after SLE with a 10.0 mM solution of **1b** in $CDCl_3$ from $LiCl{\cdot}H_2O$, **M1**, **M2,** and **M3.** For the control sample, loading was calculated per 10.0 mM of virtual extractant.

Sample	Li	Na	K	Mg
$LiCl{\cdot}H_2O$	61	<0.005	<0.0004	<0.0002
**M1**	26	<0.005	<0.0004	<0.0002
**M2**	78	<0.005	<0.0004	0.63
**M3**	67	<0.005	<0.0004	<0.0002
Control	0.004	0.216	0.04	<0.0002

Table S12. Loading [%] of **1b** with aluminium, manganese, iron, cobalt, nickel, and copper, measured with ICP-MS/MS from samples after SLE with a 10.0 mM solution of **1b** in $CDCl_3$ from $LiCl \cdot H_2O$, **M1**, **M2,** and **M3.** For the control sample, loading was calculated per 10.0 mM of virtual extractant.

Sample	Al	Mn	Fe	Co	Ni	Cu
**M3**	0.34	0.88	0.000	0.001	0.000	32[a]

[a] As copper is not present in active cathode material ($LiFePO_4$), it probably comes from oxidized current collectors or other parts of the cell casing.

Table S13. Loading [%] of **1b** with lithium measured with ICP-MS/MS from samples after LLE with a 10.0 mM solution of **1b** in $CDCl_3$ from 1M aqueous solutions of LiCl, LiBr, LiOTf, and $LiNTf_2$. For the control sample, loading was calculated per 10.0 mM of virtual extractant.

Sample	Control	LiCl	LiBr	LiOTf	$LiNTf_2$
**Loading [%]**	1.3	7.4	15.5	21	107

4.6 Large-scale experiments

Solvent, extraction time, and stoichiometry selection

For a large-scale experiment, we decided not to pursue chloroform, used for NMR studies, due to its toxicity and limited chemical stability, possibly resulting in the formation of hydrogen chloride and phosgene. Dichloromethane was selected instead because it combines a low boiling point, limited miscibility with water, and reliable phase separation, while remaining similar in polarity to chloroform, and less problematic than other non-coordinating solvents commonly used in SLE/LLE studies, such as nitrobenzene. Oxygen-donor solvents (e.g., Et_2O, 2-MeTHF) were excluded due to potential competition for Li^+ binding and peroxide formation. Hydrocarbon solvents were not explored at this stage, as the deuterated analogues required for NMR-based studies are comparatively expensive and less practical for method development.

Extraction time of 24 h, much longer than the time required for equilibration in the case of a kinetic NMR experiment with excess of LiCl alone (~ 1 h), was chosen to secure full equilibration of the system, preventing any kinetic phenomenon affecting selectivity and therefore assuring reproducibility.

Aiming at the evaluation of the practicality of the system, we decided to use a stoichiometric amount of **1b** and LiCl. Notably, in most cases reported in the literature, an excess of lithium salt is used, and, as a consequence, only host loading, not lithium extraction yield, is typically determined. Due to the use of stoichiometric amounts of **1b** and LiCl in this study, we were able to determine both (Table S14).

Cationic composition of the model salt mixture was designed to reflect the average reported metal composition at Salar de Atacama. Chlorides were used, as they are the main component of most salt brines. Calcium chloride was added to more accurately represent the Salar de Atacama composition.

Preparation of model salt mixture modified M2 for large-scale reusability experiments

Dry LiCl (4.24 g, 0.100 mol), NaCl (42.56 g, 0.7282 mol), KCl (15.03 g, 0.2016 mol), $MgCl_2 \cdot 6H_2O$ (32.48 g, 0.1598 mol), and $CaCl_2$ (274 mg, 2.47 mmol) were dissolved in ~300 mL of hot water. The solution was then concentrated on a rotary evaporator (~20 mbar, 100°C) to a solid residue, which was transferred to a mortar and ground, yielding 97.47 g of modified **M2** containing 0.100 mol of lithium.

Reusability evaluation

To assess **1b** reusability, three cycles of consecutive extractions were performed under two different scenarios, described below. Briefly, in the first scenario, all extractions were performed from the same re-used portion of modified **M2**, probing the influence of decreasing lithium content on **1b** retention. In the second scenario, new portions of the modified **M2** were used for every cycle, probing the influence of higher lithium content on the **1b** retention over its use.

Three cycles of extraction from one portion of modified M2

In a 250 mL flask, 9.75 g of modified **M2** (containing ~0.01 mol of lithium) was placed. Subsequently, a stirring bar and a solution of 3.363 g (0.0100 mol) of **1b** in 100 mL of CH_2Cl_2 were added, and the suspension was stirred at 700 rpm at room temperature for 24 h. Then, the solids were filtered on a G3 Schott funnel, washed with 2 × 20 mL of CH_2Cl_2, transferred back to a 250 mL round-bottom flask, and dried on a rotary evaporator. The organic solution was transferred to a separatory funnel and washed with 2 × 25 mL of distilled water. The aqueous phases containing lithium chloride were combined and washed with 25 mL of CH_2Cl_2, then concentrated, dried (100°C, vacuum pump), and transferred to a 5 mL volumetric flask with CD_3OD. Spectrophotometric analysis of lithium concentrations was performed, and a ^1H NMR spectrum of this solution with the addition of pentachloroethane as a reference was measured. Organic phases, containing **1b**, were also combined, concentrated, and vacuum-dried. The recovered **1b** was weighed, and a ^1H NMR spectrum was recorded (with the NMR sample being re-transferred to the rest of the **1b** portion after measurement). Retained **1b** was again dissolved in 100 mL of CH_2Cl_2, and the whole extraction procedure was repeated twice with the same portion of modified **M2**. Full results of this experiment are collected in Table S14 and in Figures S140 and S141.

Table S14. **1b** retention and losses, and lithium loading in the three consecutive extractions from the same portion of modified **M2**.

	1st extraction	2nd extraction	3rd extraction
**1b** recovery	3.350 g (99.6%)[a]	3.347 g (99.5%)[a]	3.341 g (99.3%)[a]
**1b** loss	0.013 g (0.39%)	0.003 g (0.12%)	0.006 g (0.18%)
**1b** lithium loading [%]	37 ± 4[b]	18 ± 2[b]	16 ± 2[b]

[a] In respect to the starting mass of **1b** (3.363 g). [b] Determined spectrophotometrically.

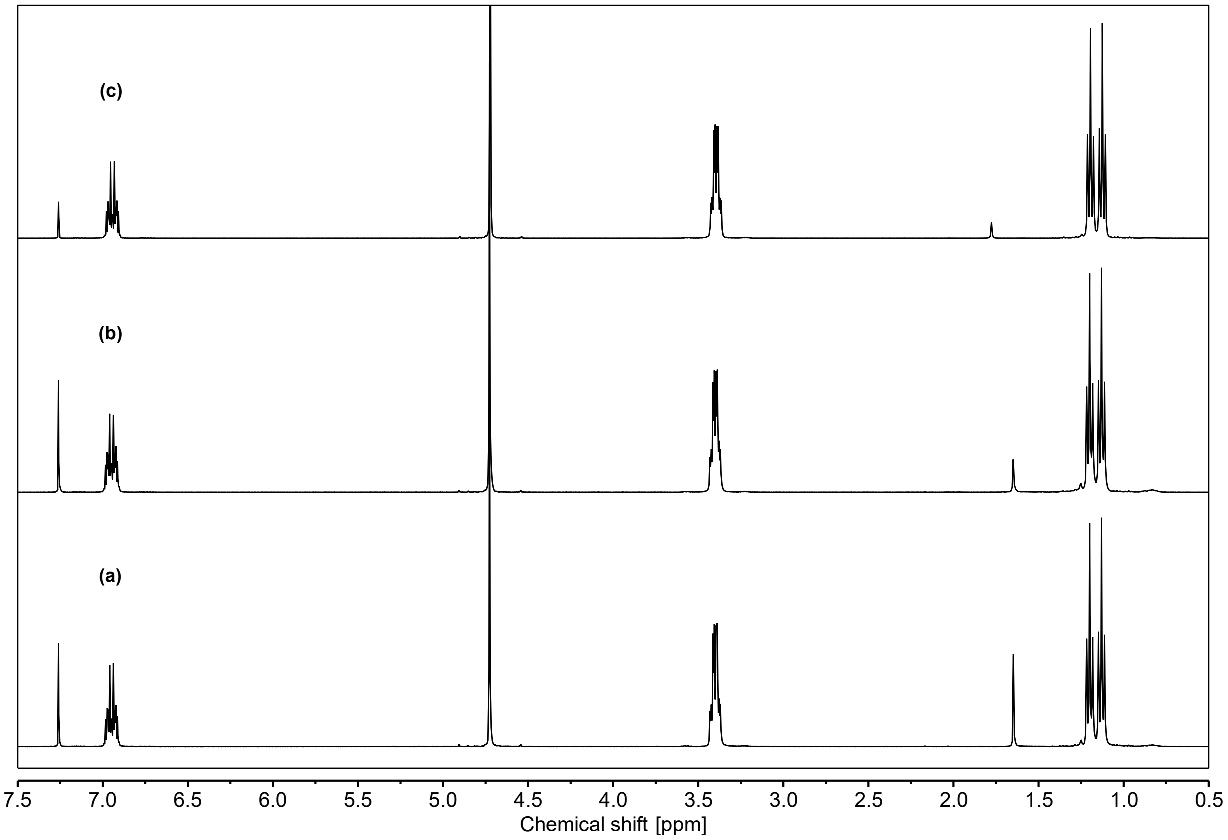

Figure S140. ^{1}H NMR spectra of **1b** recovered after first (a), second (b), and third (c) extraction cycle from the re-used portion of modified **M2**.

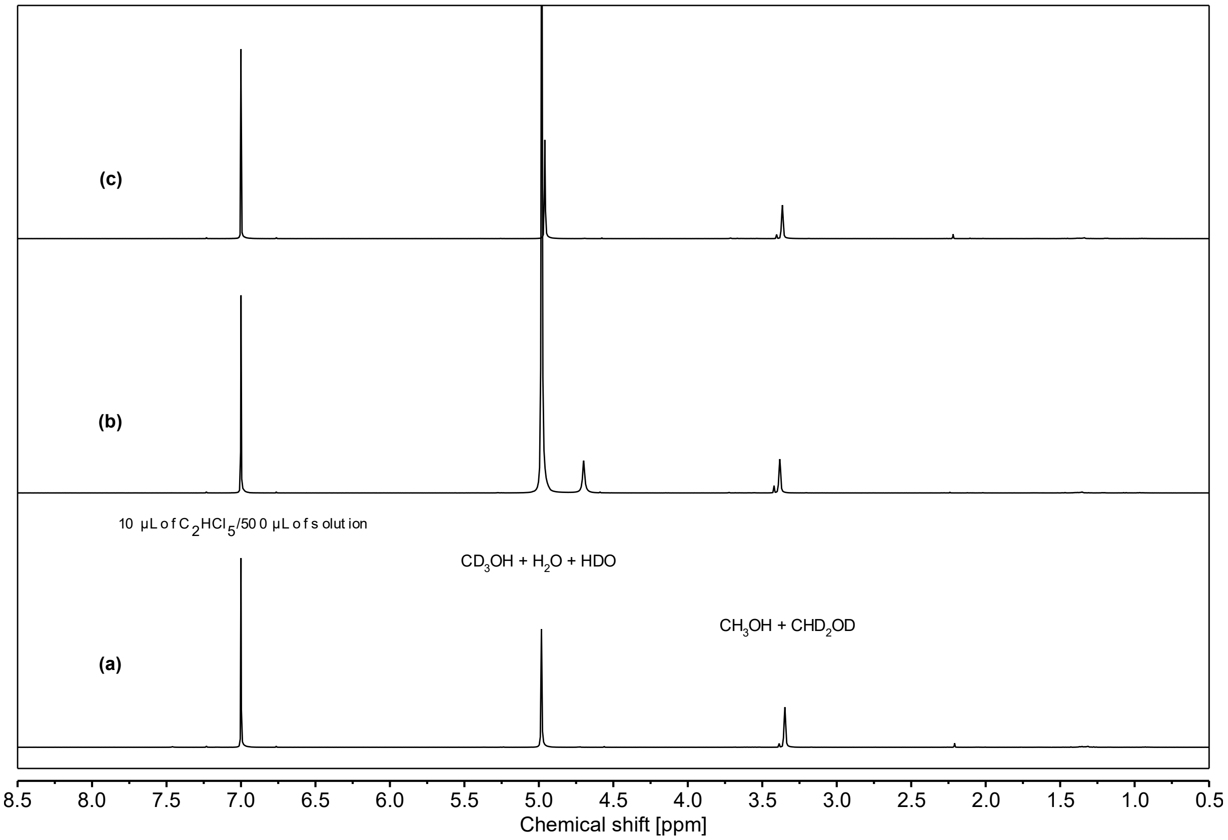

Figure S141. ^{1}H NMR spectra of the solutions of LiCl obtained after first (a), second (b), and third (c) extraction cycle from the re-used portion of modified **M2**, measured in CD_3OD with the addition of 10 μL of internal standard (C_2HCl_5) per 500 μL of the solution, showing no sign of **1b** presence.

Three cycles of extraction from three independent portions of modified **M2**

In a 250 mL flask, 9.75 g of modified **M2** (containing ~0.01 mol of lithium) was placed. Subsequently, a stirring bar and a solution of 3.363 g (0.0100 mol) of **1b** in 100 mL of CH_2Cl_2 were added, and the suspension was stirred at 700 rpm at room temperature for 24 h. Then, the solids were filtered on a G3 Schott funnel, washed with 2 × 20 mL of CH_2Cl_2, and discarded. The organic solution was transferred to a separatory funnel and washed with 2 × 25 mL of distilled water. The aqueous phases containing lithium chloride were combined and washed with 25 mL of CH_2Cl_2, then concentrated, dried (100°C, vacuum pump), and transferred to a 5 mL volumetric flask with CD_3OD. Spectrophotometric analysis of lithium concentrations was performed, and a ^1H NMR spectrum of this solution with the addition of pentachloroethane as a reference was measured. Organic phases, containing **1b**, were also combined, concentrated, and vacuum-dried. The recovered **1b** was weighed, and a ^1H NMR spectrum was recorded (with the NMR sample being re-transferred to the rest of the **1b** portion after measurement). Retained **1b** was again dissolved in 100 mL of CH_2Cl_2, and the whole extraction procedure was repeated twice with a new portion of modified **M2** in each extraction. Full results of this experiment are collected in Table S15 and in the Figures S142 and S143.

Table S15. 1b retention and losses, and lithium loading in the three consecutive extractions from three independent portions of modified **M2**.

	1st extraction	2nd extraction	3rd extraction
**1b** recovery	3.358 g (99.9%)[a]	3.353 g (99.7%)[a]	3.346 g (99.5%)[a]
**1b** loss	0.004 g (0.12%)	0.005 g (0.15%)	0.007 g (0.21%)
**1b** lithium loading [%]	41 ± 4[b]	38 ± 4[b]	40 ± 4[b]

[a] In respect to the starting mass of **1b** (3.362 g). [b] Determined spectrophotometrically.

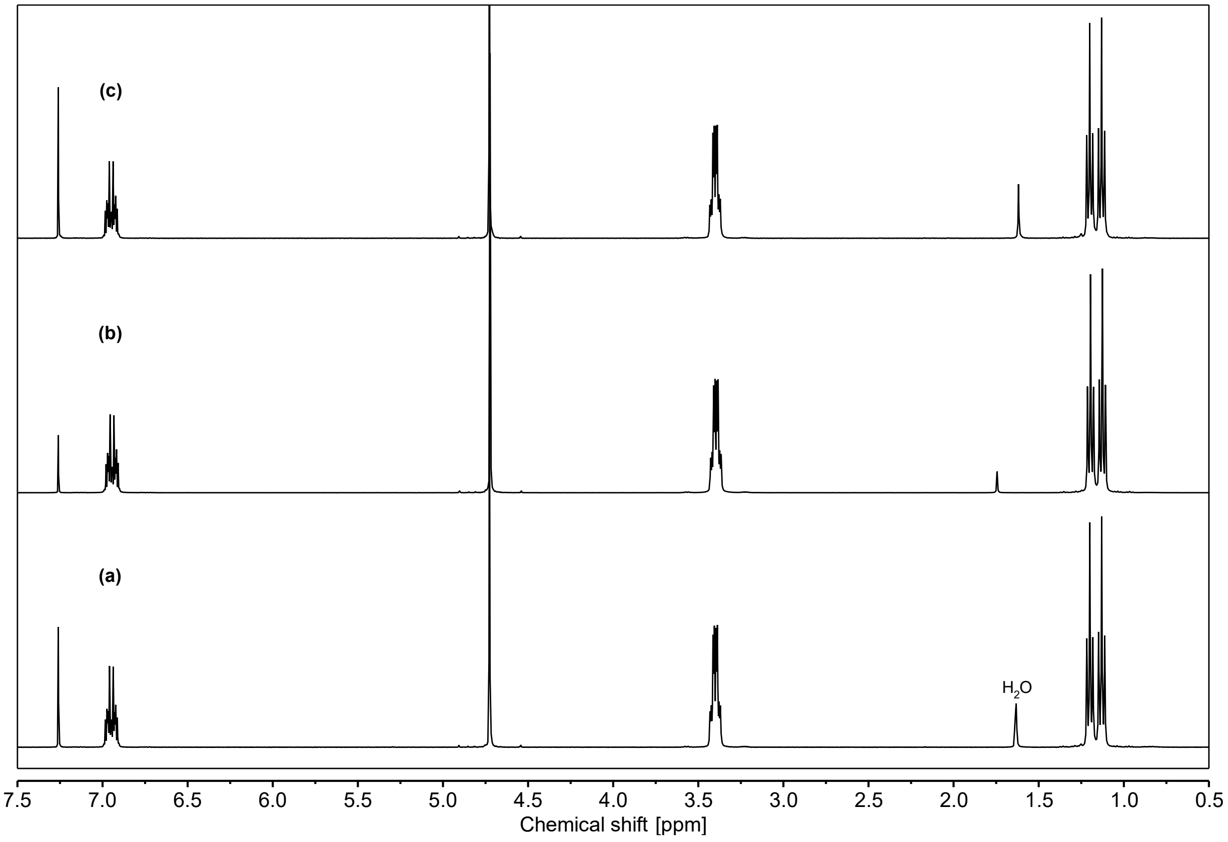

Figure S142. ^{1}H NMR spectra of **1b** recovered after first (a), second (b), and third (c) extraction cycle from independent portions of modified **M2**.

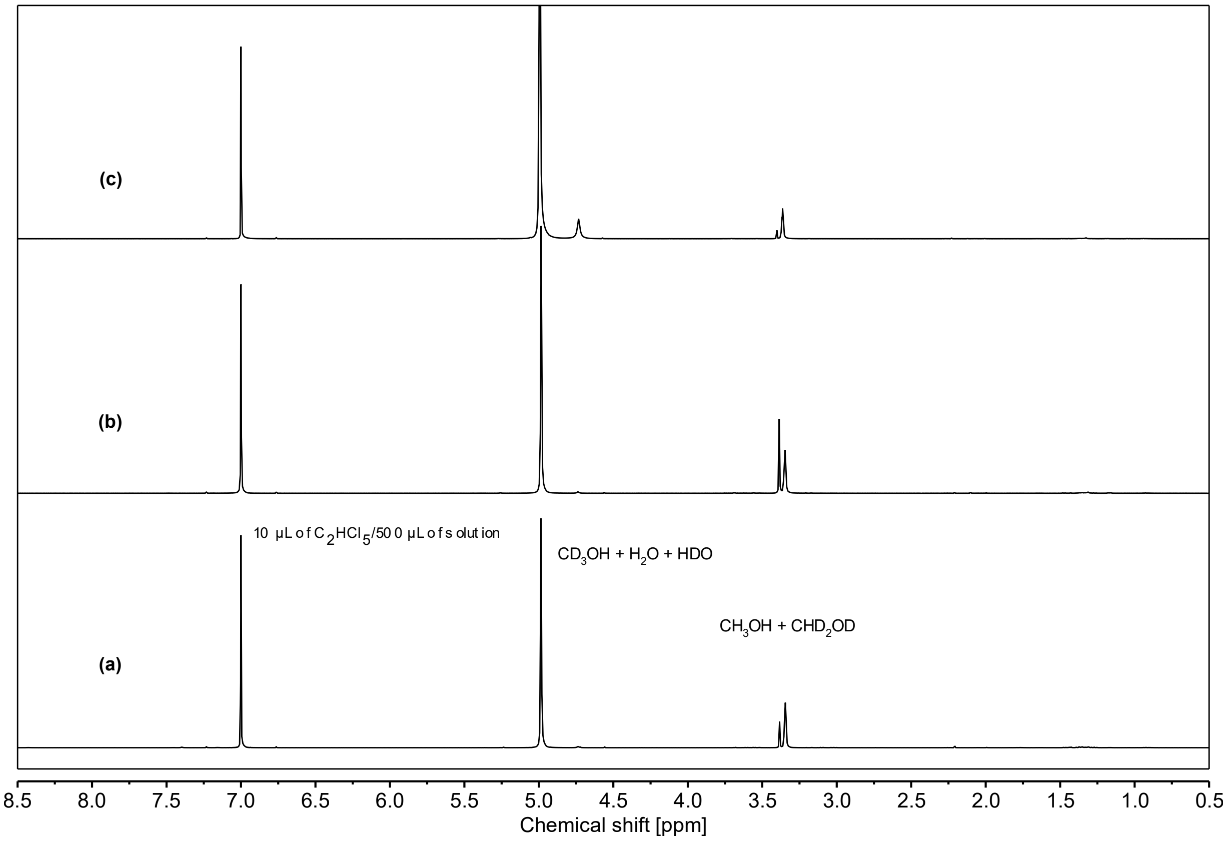

Figure S143. ^{1}H NMR spectra of the solutions of LiCl obtained after first (a), second (b), and third (c) extraction cycle from three independent portions of modified **M2**, measured in CD_3OD with the addition of 10 µL of internal standard (C_2HCl_5) per 500 µL of the solution, showing no sign of **1b** presence.

Generally, excellent reusability and activity retention of **1b** were observed across three cycles in both scenarios. Additionally, based on the fact that spectra of CD_3OD solutions of concentrated aqueous phases lack meaningful signals of **1b**, we conclude that the main reason for observed minor losses of **1b** is operational (losses associated with solution transfers, retention of solutions on the solid phase during filtration, etc.), rather than partition into the aqueous phase during the extraction steps. Taking into account the value of $P_{CH_2Cl_2/H_2O}$, we suspect that washing the aqueous phase with CH_2Cl_2 at the end of the cycle is an important step for keeping the retention of **1b** during the process at a very high level.

Large-scale extraction from the model of the Salar de Atacama salts deposit

In a 2 L round-bottom flask, 4.24 g (0.100 mol) of dry LiCl, 42.56 g (0.7282 mol) of NaCl, 15.03 g (0.2016 mol) of KCl, 32.48 g (0.1598 mol) of $MgCl_2 \cdot 6H_2O$, and 274 mg (2.47 mmol) of $CaCl_2$ (total mass 94.59 g, Li:Na:K:Mg:Ca molar ratio 1:7.3:2.0:1.6:0.025) were dissolved in hot water and evaporated (20 mbar, 90 °C) to a slightly wet white solid modified **M2** (mass ~100 g). Subsequently, a stirring bar and a solution of 33.62 g (0.100 mol) of **1b** in 1L of CH_2Cl_2 were added, and the suspension was stirred at 700 rpm at room temperature for 24 h. Then, the solids were filtered on a G3 Schott funnel, washed with 2 × 100 mL of CH_2Cl_2, transferred back to a 2 L round-bottom flask, and dried on a rotary evaporator. The organic solution was transferred to a separatory funnel and washed with 2 × 250 mL of distilled water. The aqueous phases containing lithium chloride were combined and washed with 50 mL of CH_2Cl_2, then concentrated, transferred to a 100 mL volumetric flask, and made up to volume for analysis. Organic phases, containing **1b**, were also combined, concentrated, and vacuum-dried. The recovered **1b** was again dissolved in 1 L of CH_2Cl_2, and the whole extraction procedure was repeated. After both extractions, 33.39 g (99.3%) of pure, salt-free **1b** was recovered. Representative photographs of the large-scale SLE process are shown in Figure S144.

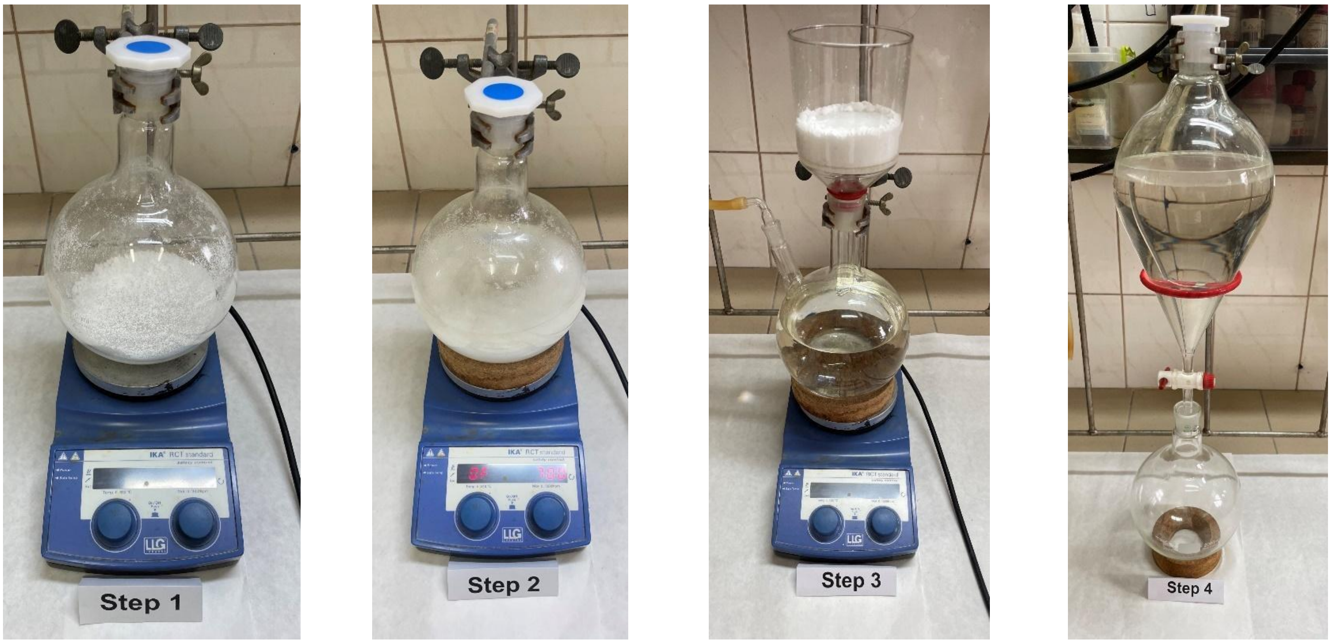

Figure S144. Photographs of large-scale SLE from **modified M2** at subsequent stages: Step 1 – starting modified **M2**, Step 2 – stirring with 1 L of a solution of **1b**, Step 3 – filtration, Step 4 – back-extraction to water.

Calibration of the spectrophotometric method for the determination of lithium concentration

The procedure was adapted from Thomason[40] with modifications. To six separate 10 mL volumetric flasks, consecutively 0, 10, 20, 30, 40, and 50 µL of 5.30 mM Li_2SO_4 solution were added, followed by 0.2 mL of 20% $KOH_{(aq)}$, 7 mL of acetone, and 200 µL of aqueous Thorin solution (2 mg/mL). The solutions were made up to volume with distilled water, shaken vigorously, and left for 30 minutes for the colour to develop. For the content of every flask, the spectra were measured three times and then averaged. An excellent linear correlation between the ratio of absorbances at 480 nm and 390 nm was observed and therefore used for the construction of the calibration curve (Figure S145).

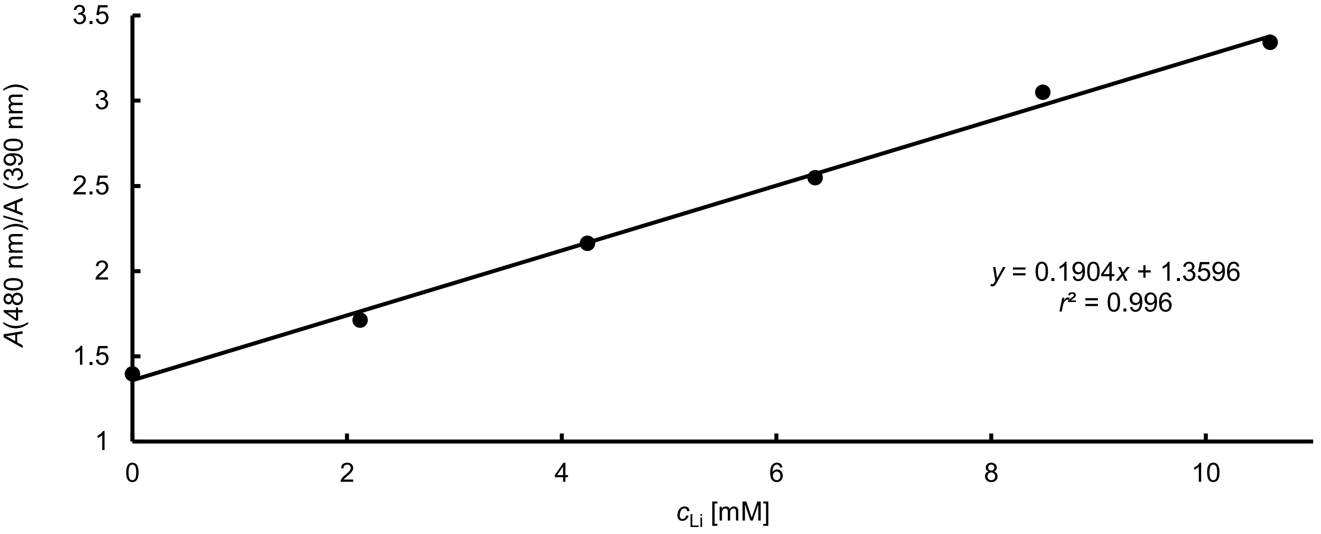

Figure S145. Calibration curve for the spectrophotometric method for the determination of lithium concentration.

Procedure for the spectrophotometric determination of lithium concentration

To a 10 mL volumetric flask, 50 µL of analyte with a lithium concentration up to 10 mM was added, followed by 0.2 mL of 20% $KOH_{(aq)}$, 7 mL of acetone, and 200 µL of aqueous Thorin solution (2 mg/mL). The solution was made up to volume with distilled water, shaken vigorously, and left for 30 minutes for the colour to develop. Analysis was performed three times for every sample, providing standard deviation, and for every analysis, the spectra were measured three times and then averaged.

ICP-MS and spectrophotometric analysis of samples after large-scale SLE

Samples were diluted 400 times before measurement on the Perkin Elmer NexION 2000 apparatus. For every sample, two portions of solution were subjected to measurement, and each portion of solution was measured 3 times. Standard deviation was < 1% for lithium, and <1.5% for sodium, potassium, magnesium, and calcium. The elements' concentrations in liquid samples were calculated by multiplying the measured values by the samples' dilution factor. Results of the analysis are given in Table S16.

Table S16. Loading [%] of **1b** with the given element in the solutions obtained after SLE from modified **M2** with **1b**.

Sample	Li	Na[b]	K[b]	Mg[b]	Ca[b]
1st extraction	64 ± 4[a]<br>60 ± 6[b]	0.05 ± 0.005	0.015 ± 0.002	0.028 ± 0.003	0.4 ± 0.04
2nd extraction	26 ± 4[a]<br>26 ± 3[b]	0.09 ± 0.009	0.017 ± 0.002	0.008 ± 0.0008	1.1 ± 0.1
Mixture[c]	95 ± 5[a]<br>86 ± 9[b]	0.13 ± 0.02	0.03 ± 0.003	0.035 ± 0.004	1.5 ± 0.2

[a] Determined spectrophotometrically. [b] Determined with ICP-MS. [c] Values multiplied by a factor of 2, accounting for dilution accompanying mixing of two solutions.

4.7 Thermodynamic analysis of $LiCl\cdot H_2O$ vs LiCl solubilization

To further rationalize the experimentally observed preference for the solubilization of hydrated LiCl over anhydrous LiCl under SLE conditions, a simple thermodynamic analysis was performed. The analysis compares model reactions describing the transfer of lithium chloride from the solid state into a low-polarity environment, approximated by the gas phase due to the availability of reliable thermodynamic data.

Specifically, the solubilization of $LiCl\cdot H_2O_{(s)}$ was modeled as:

(a) $LiCl\cdot H_2O_{(s)} \rightarrow Li(H_2O)^+_{(g)} + Cl^-_{(g)}$,

while the corresponding process for anhydrous $LiCl_{(s)}$ was treated as:

(b) $LiCl_{(s)} \rightarrow Li^+_{(g)} + Cl^-_{(g)}$.

Notably, the first reaction is a sum of the following steps:

1. $LiCl\cdot H_2O_{(s)} \rightarrow LiCl_{(s)} + H_2O_{(g)}$
2. $LiCl_{(s)} \rightarrow Li^+_{(g)} + Cl^-_{(g)}$ (equivalent to (b))
3. $Li^+_{(g)} + H_2O_{(g))} \rightarrow Li(H_2O)^+_{(g)}$

Based on data from NIST and Monnin et al.[41], the enthalpies of reaction (a) and (b) were directly calculated to be 786 $kJ\cdot mol^{-1}$ and 861 $kJ\cdot mol^{-1}$, respectively. Therefore, a single water molecule lowers the overall enthalpic penalty for lithium transfer by approximately 75 $kJ\cdot mol^{-1}$ relative to anhydrous LiCl, providing a thermodynamic basis for the experimentally observed hydration-assisted solubilization behavior.

5. Characterization of spectrophotometric sensor 1j

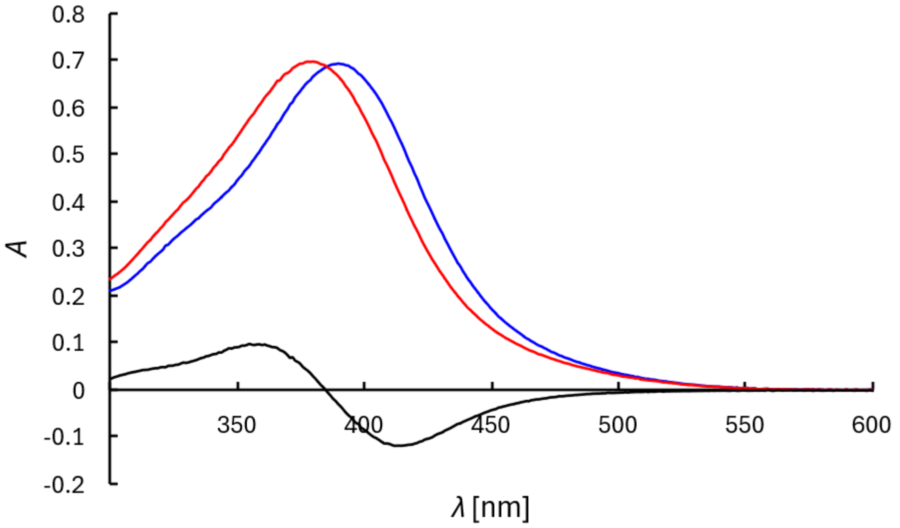

Figure S146. Spectra of $4 \cdot 10^{-6}$ M solution of **1j** in CH_3CN + 0.4% H_2O: blue line – without lithium chloride, red line – with $4.16 \cdot 10^{-5}$ M of lithium chloride, black line – differential spectra.

Calibration of the spectrophotometric method for the determination of lithium concentration with 1j

To 6 separate quartz UV-Vis cuvettes (screw-cap, 1 cm path length), 2.48 mL of CH_3CN, 10 μL of 1 mM solution of **1j** in CH_3CN, and 10 μL of LiCl solutions with concentrations 0 mM, 2.08 mM, 4.16 mM, 6.24 mM, 8.32 mM, 10.4 mM were added. For the content of every cuvette, the spectra were measured three times and then averaged. Good linear correlation between the ratio of absorbances at 355 nm and 415 nm was observed and therefore used to construct the calibration curve (Figure S147). Standard deviation of ($c_{exact} - c_{determined}$) was found to be 0.3.

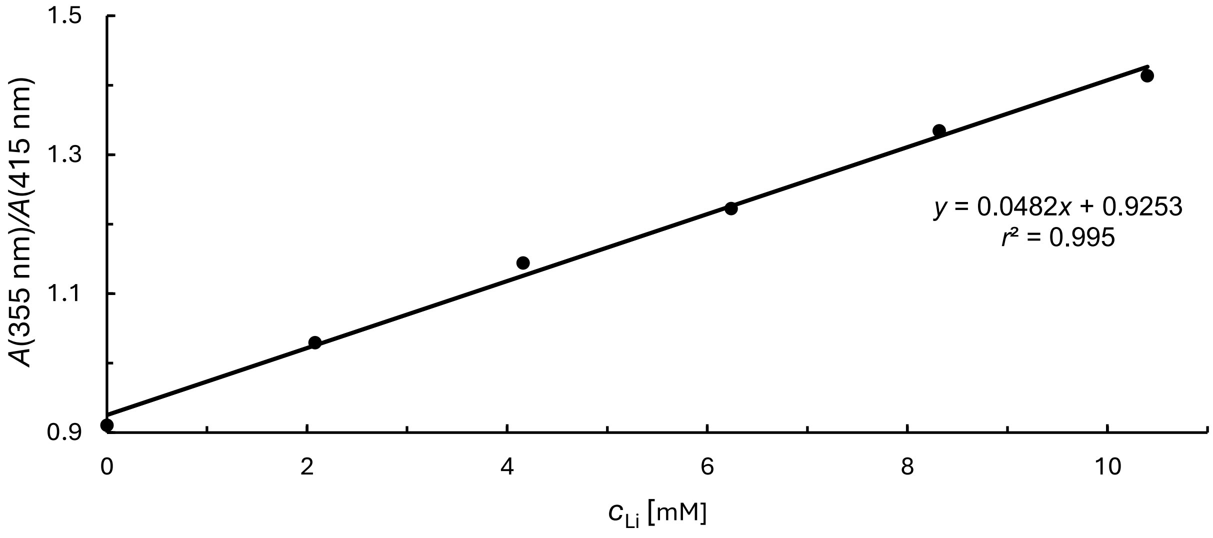

Figure S147. Calibration for spectrophotometric determination of lithium concentration with **1j**.

Procedure for spectrophotometric determination of lithium concentration with 1j

To a quartz UV-Vis cuvette (screw-cap, 1 cm path length), 2.48 mL of CH_3CN, 10 µL of a 1 mM solution of **1j** in CH_3CN, and 10 µL of analyte solution with a lithium concentration up to 10 mM was added. The spectra were measured three times and then averaged.

Selectivity evaluation

Addition of an equimolar amount of sodium or potassium to a 5.2 mM solution of LiCl led to determined lithium concentrations of 5.41 and 5.14 mM, respectively, in both cases within the standard deviation of the method.

6. DFT calculations

Energy-minimized structures of free molecular receptors and their complexes with alkali metal (Li, Na, K) triflates (TfO) were obtained by adopting a previous protocol.[42] The structures with the lowest energies were subjected to optimization and frequency calculations using Gaussian 16 (Rev. A.03) with the M06-2X[43] functional, which has good performance for describing noncovalent interactions and thermochemistry. Structure optimization and frequency calculations were performed at the def2-TZVP level of theory with a CPCM(MeCN) solvent model.[44] Solvated free energies were calculated as $G_{solv} = E_{SCF} + G_{corr}$, where E_{SCF} is taken from the output file of the solvent calculation ("SCF DONE: E(R_functional) = XX") and G_{corr} is taken from the output of the frequency calculation ("Thermal correction to Gibbs Free Energy = XX").

The interaction free enthalpies were calculated as $\Delta G_{int} = \Delta G_{complex} - [\Delta G_{host} + \Delta G_{MOTf}]$, where $\Delta G_{complex}$, ΔG_{host}, and ΔG_{MOTf} are the solvated free energies for the complex, receptor, and metal triflate salt, respectively. The stepwise 2:1 complex formations were calculated as $\Delta G_{2:1} = \Delta G(\mathbf{1b}_2 \cdot M^+) - [\Delta G(\mathbf{1b} \cdot M^+) + \Delta G_{\mathbf{1b}}]$, where $\Delta G(\mathbf{1b}_2 \cdot M^+)$, $\Delta G(\mathbf{1b} \cdot M^+)$, and $\Delta G_{\mathbf{1b}}$ are the solvated free energies for the 2:1 host:guest complex of **1b** with metal cation, 1:1 host:guest complex of **1b** with metal cation, and free host, respectively.

All final optimized structures were verified by vibrational frequency calculations, confirming the absence of imaginary frequencies and indicating that the obtained geometries correspond to true minima on the potential energy surface.

As the size of the alkali metal cation increases from Li^+ to Na^+ and K^+, the cation progressively moves out of the binding cleft of hosts **1f** and **1b**, and becomes weakly encapsulated. These trends are clearly visible in Figures S148 and S149, where the $M^+\cdots O$ distances systematically elongate, and the interaction free energy ΔG_{int} is most favorable for the complexes of **1f** and **1b** with lithium triflate (LiOTf).

Comparison of ΔG_{int} values for **1f**·MOTf and **1b**·MOTf indicates that **1b** forms significantly more stable complexes with LiOTf than **1f**, in agreement with the higher association constants obtained from the ^{1}H NMR titrations in CD_3CN + 0.5% H_2O (see Table S4-5 and Table 1 in the main text). In contrast, the average $M^+\cdots O$ bond lengths for a given cation are very similar in the **1b**·MOTf and **1f**·MOTf complexes, which suggests that the enhanced Li^+ binding by **1b** arises mainly from host preorganization.

The calculated structures of the 2:1 complexes $\mathbf{1b}_2 \cdot M^+$ (Figure S150) further support the solution data. The interaction free enthalpy for $\mathbf{1b}_2 \cdot Li^+$ is close to zero, and this species is not detected in ^{1}H NMR experiments, whereas $\mathbf{1b}_2 \cdot K^+$ is substantially more stable than $\mathbf{1b}_2 \cdot Na^+$, consistent with the more pronounced formation of ternary $\mathbf{1b}_2 \cdot K^+$ complexes in solution. Notably, the $K^+\cdots O$ distances in $\mathbf{1b}_2 \cdot K^+$ are very similar to those in **1b**·KOTf, indicating that K^+ can be accommodated with a comparable interaction efficiency in both 1:1 and 2:1 complexes. The coordination numbers obtained from the DFT structures also follow this trend: $\mathbf{1b}_2 \cdot Li^+$ has CN = 6, because two oxygen donors do not coordinate to Li^+, whereas $\mathbf{1b}_2 \cdot Na^+$ and $\mathbf{1b}_2 \cdot K^+$ both reach CN = 8. The Cartesian x, y, z coordinates of all optimized structures are provided in the Excel file "SI_Cartesian_Coordinates_of_Calculated_Structures.xlsx" in the Supporting Information.

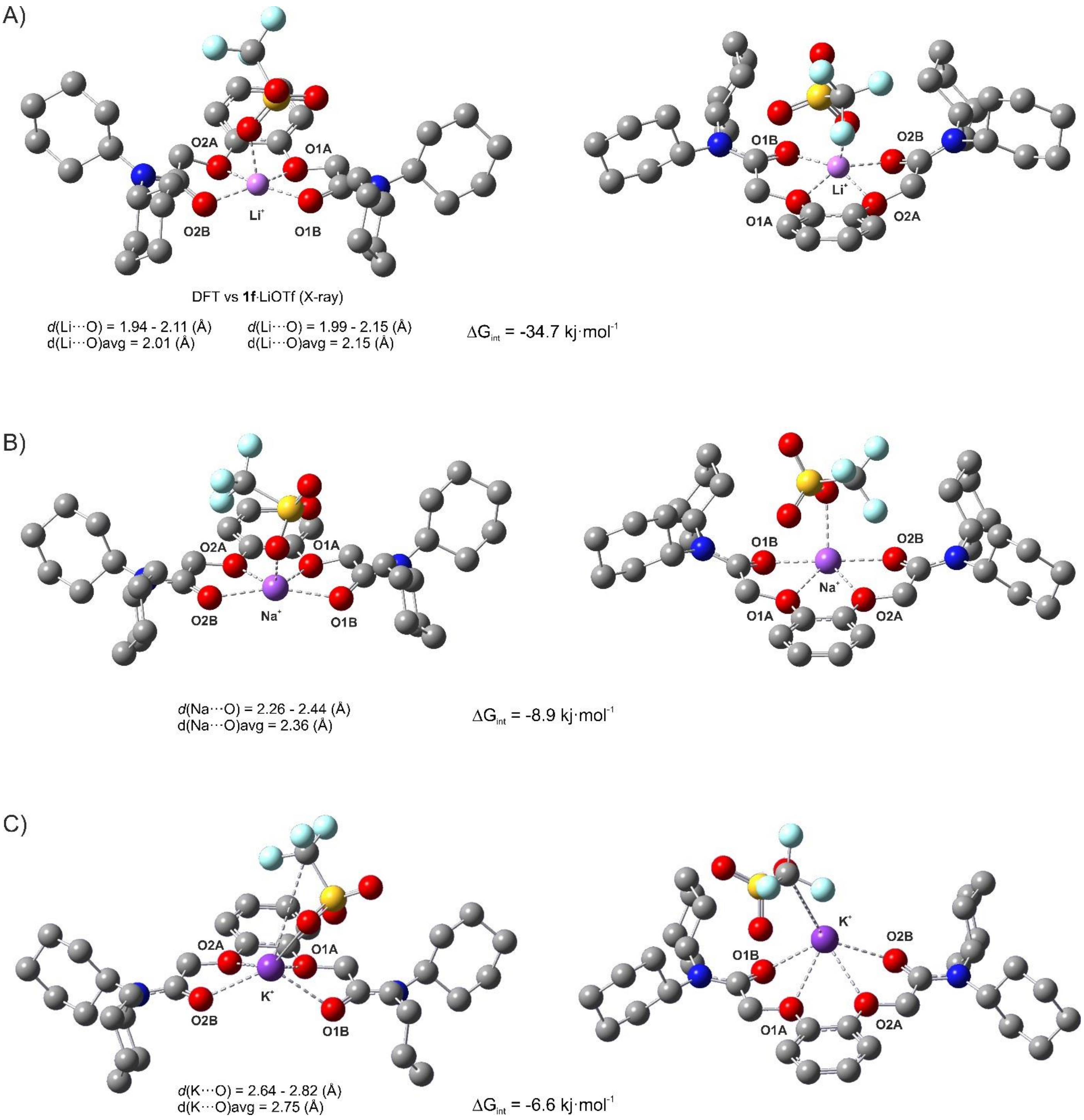

Figure S148. Front (left) and back (right) views of the DFT-optimized structures of complexes **1f**·LiOTf (A), **1f**·NaOTf (B), and **1f**·KOTf (C). Geometries were optimized at the DFT/M06-2X/def2-TZVP level with a CPCM(MeCN) solvent model. Hydrogen atoms omitted for clarity. In panel A, Li···O bond lengths obtained from DFT optimization are compared with those from the X-ray structure of **1f**·LiOTf, showing very good agreement.

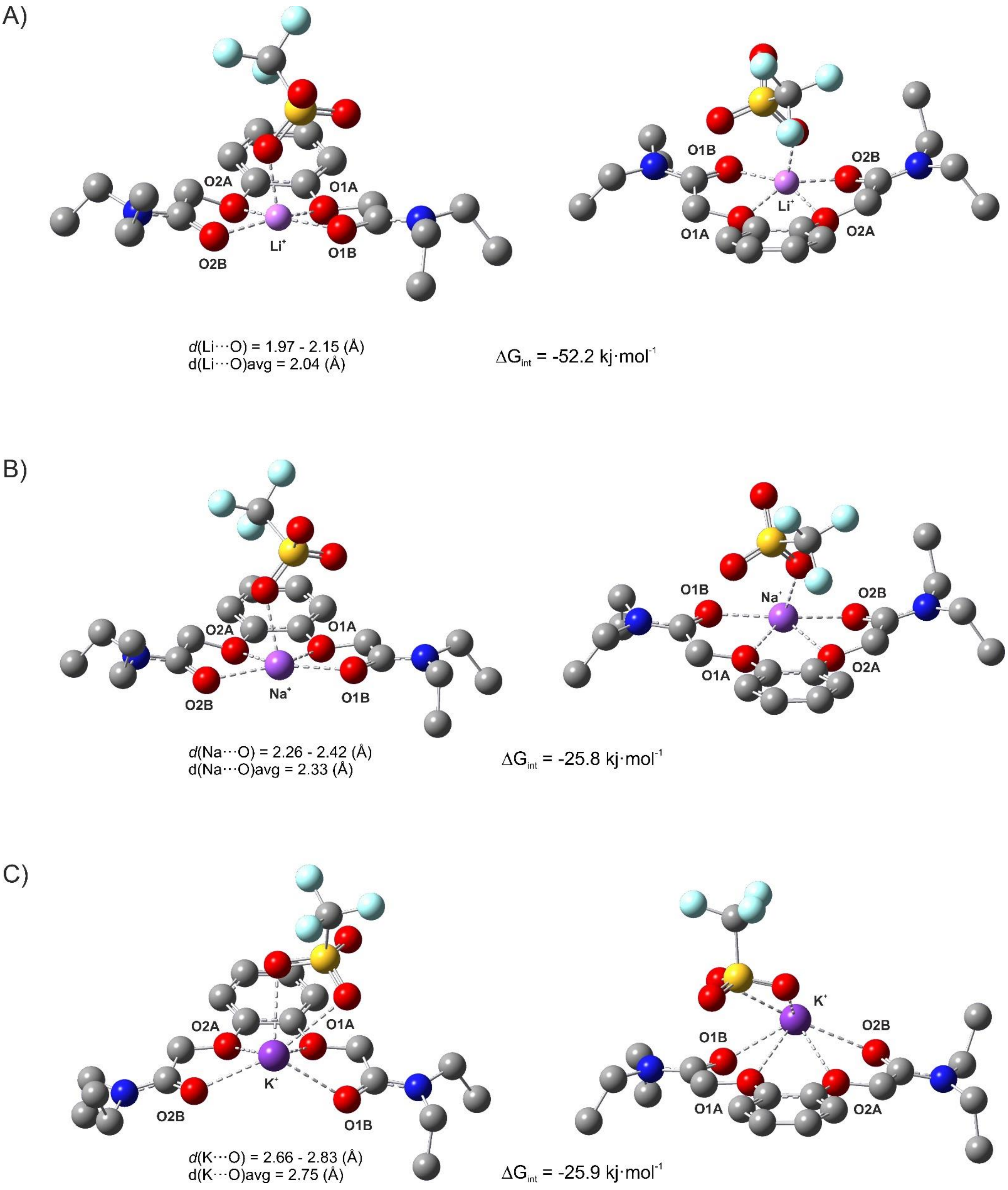

Figure S149. Front (left) and back (right) views of the DFT-optimized structures of complexes **1b**·LiOTf (A), **1b**·NaOTf (B), and **1b**·KOTf (C). Geometries were optimized at the DFT/M06-2X/def2-TZVP level with a CPCM(MeCN) solvent model. Hydrogen atoms omitted for clarity.

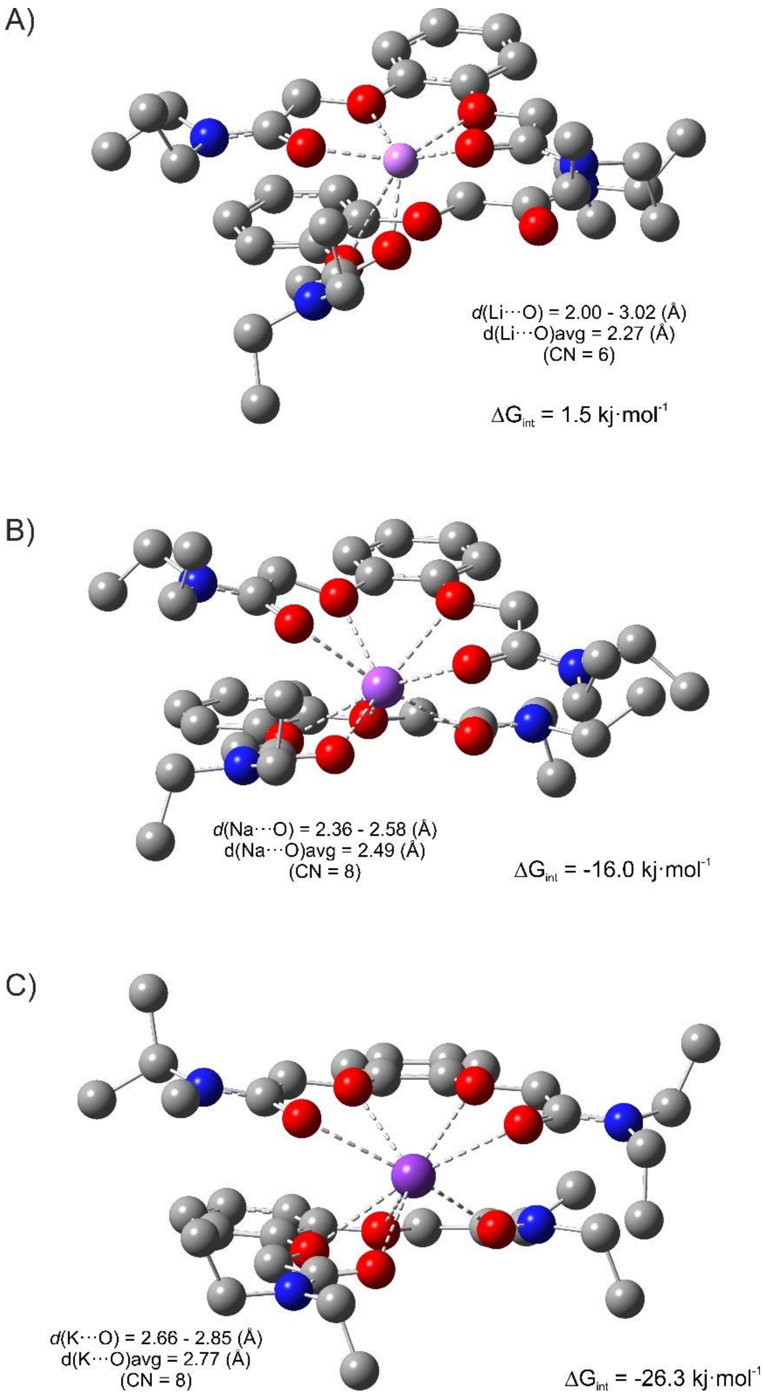

Figure S150. Front views of the DFT-optimized structures of 2:1 (host:guest) complexes $\mathbf{1b_2}\cdot Li^+$ (A), $\mathbf{1b_2}\cdot Na^+$ (B), and $\mathbf{1b_2}\cdot K^+$ (C). Geometries were optimized at the DFT/M06-2X/def2-TZVP level with a CPCM(MeCN) solvent model. Hydrogen atoms omitted for clarity. CN denotes the metal ion coordination number, defined here as the number of oxygen donor atoms within 3.5 Å from the metal center.

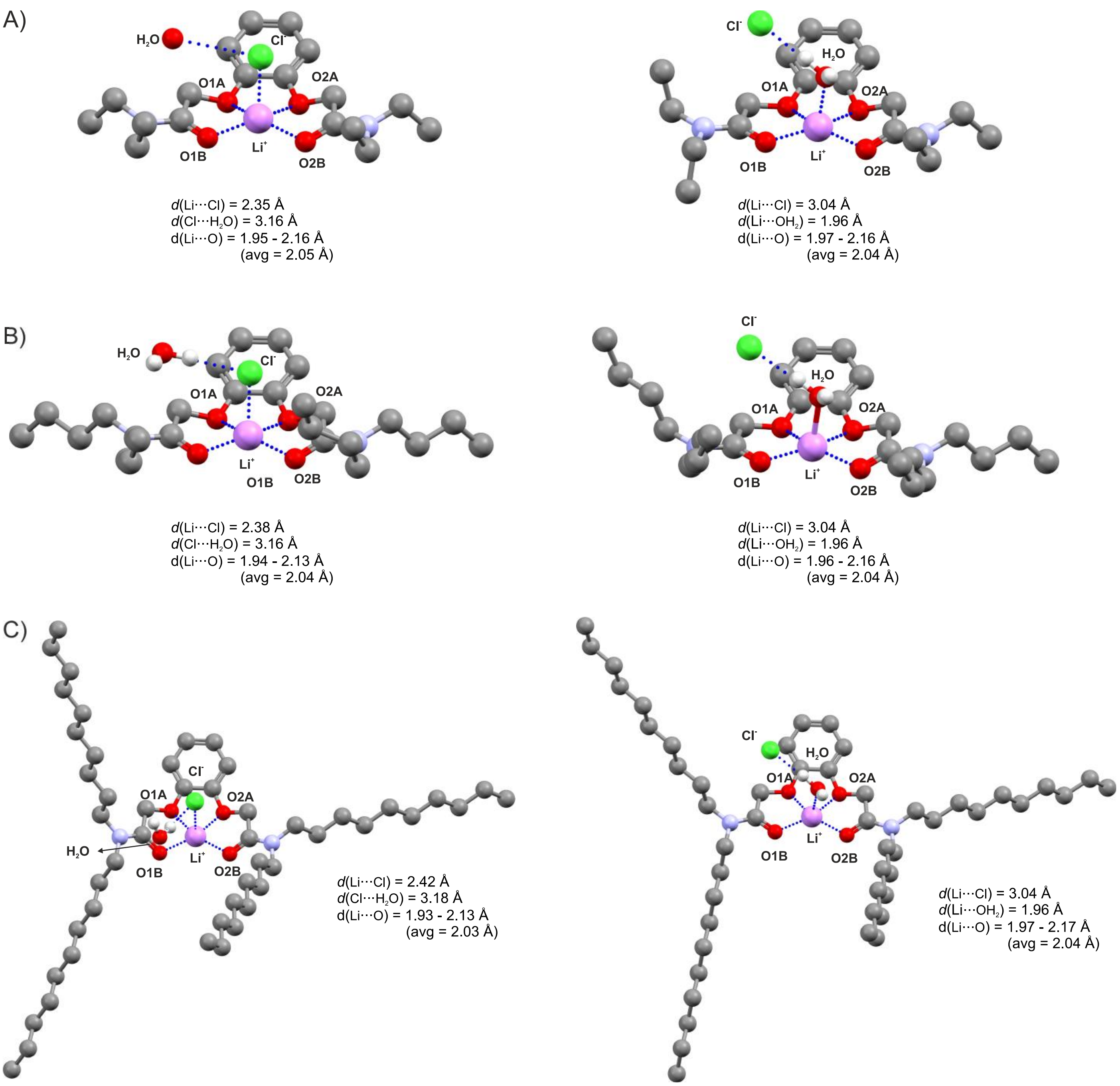

Figure S151. Front views of the DFT-optimized structures and selected bond lengths of complexes of hosts **1b–d** with LiCl·H_2O, illustrating contact ion pairing (CIP, left) and water bridged ion pairing (right) for **1b** (A), **1c** (B), and **1d** (C). Geometries were optimized at the DFT/M06-2X/def2-TZVP level with CPCM(chloroform). Non acidic hydrogen atoms are omitted for clarity. In all cases, the CIP arrangement is slightly more stable than the water-bridged ion pair, but the computed energy differences are small and remain within the expected method error ($\Delta\Delta G = -3.3$, -4.0, and -0.9 kJ·mol^{-1} for **1b**, **1c**, and **1d**, respectively). Calculated energies and XYZ coordinates are given in the Appendix.

7. NMR Spectra

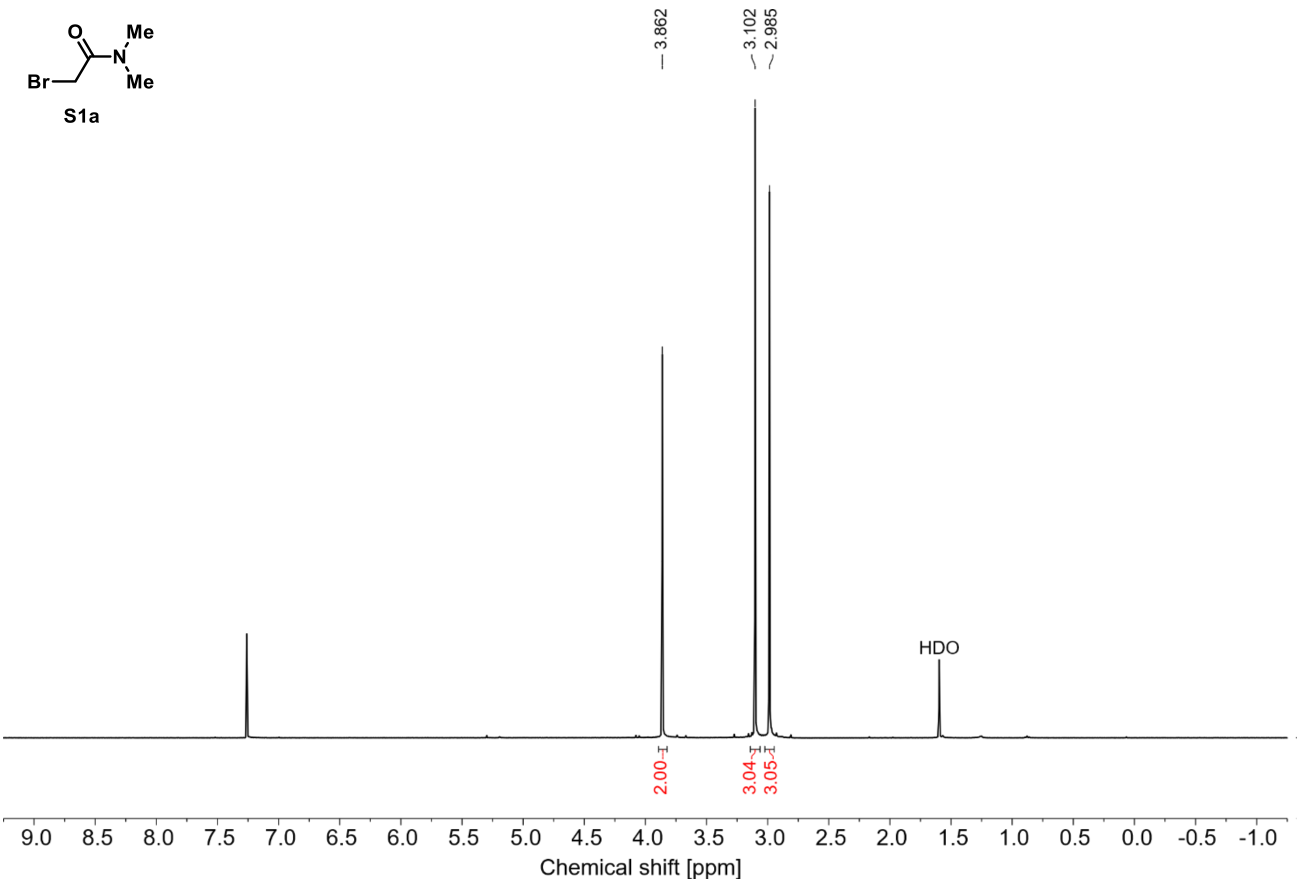

Figure S152. ^{1}H NMR spectrum of compound **S1a** (400 MHz, $CDCl_3$).

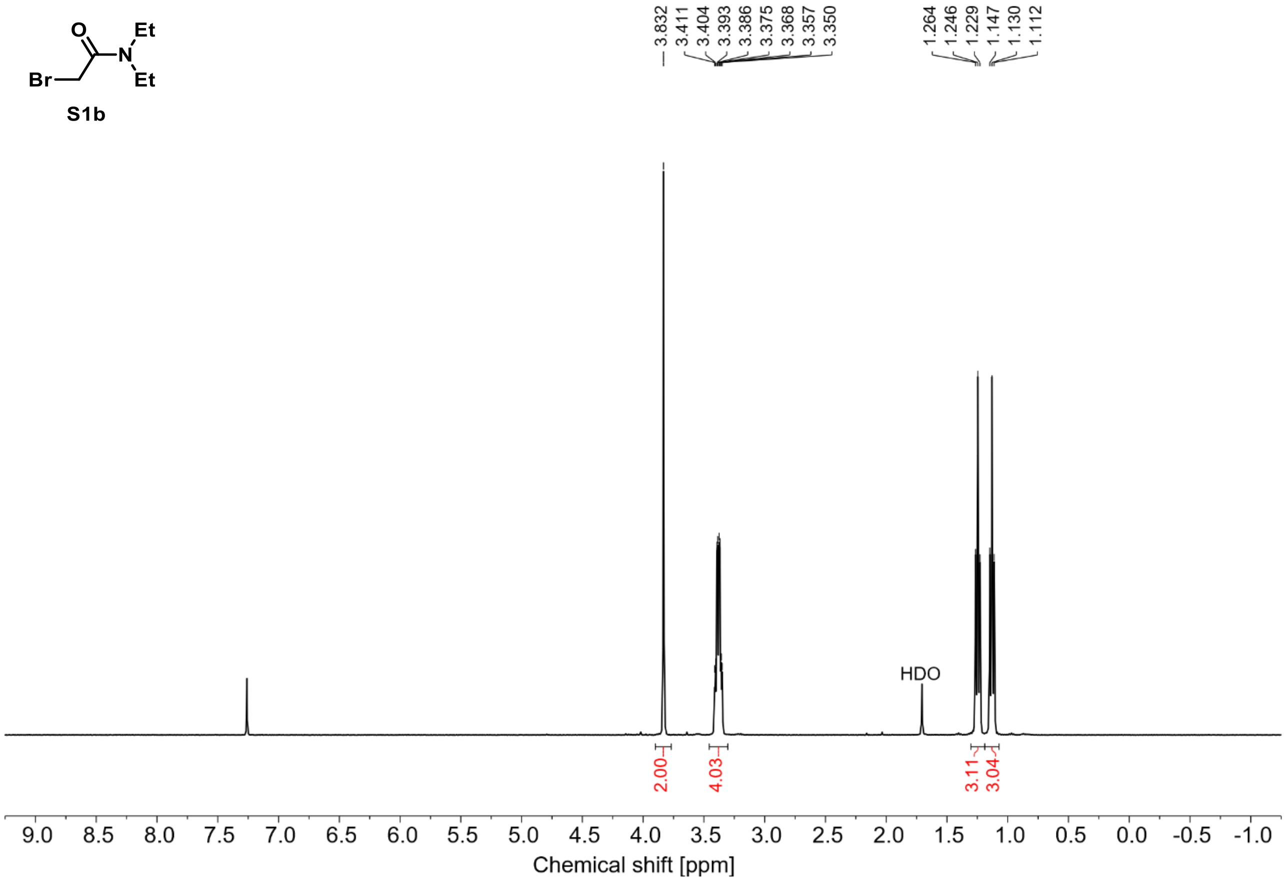

Figure S153. ^{1}H NMR spectrum of compound **S1b** (400 MHz, $CDCl_3$).

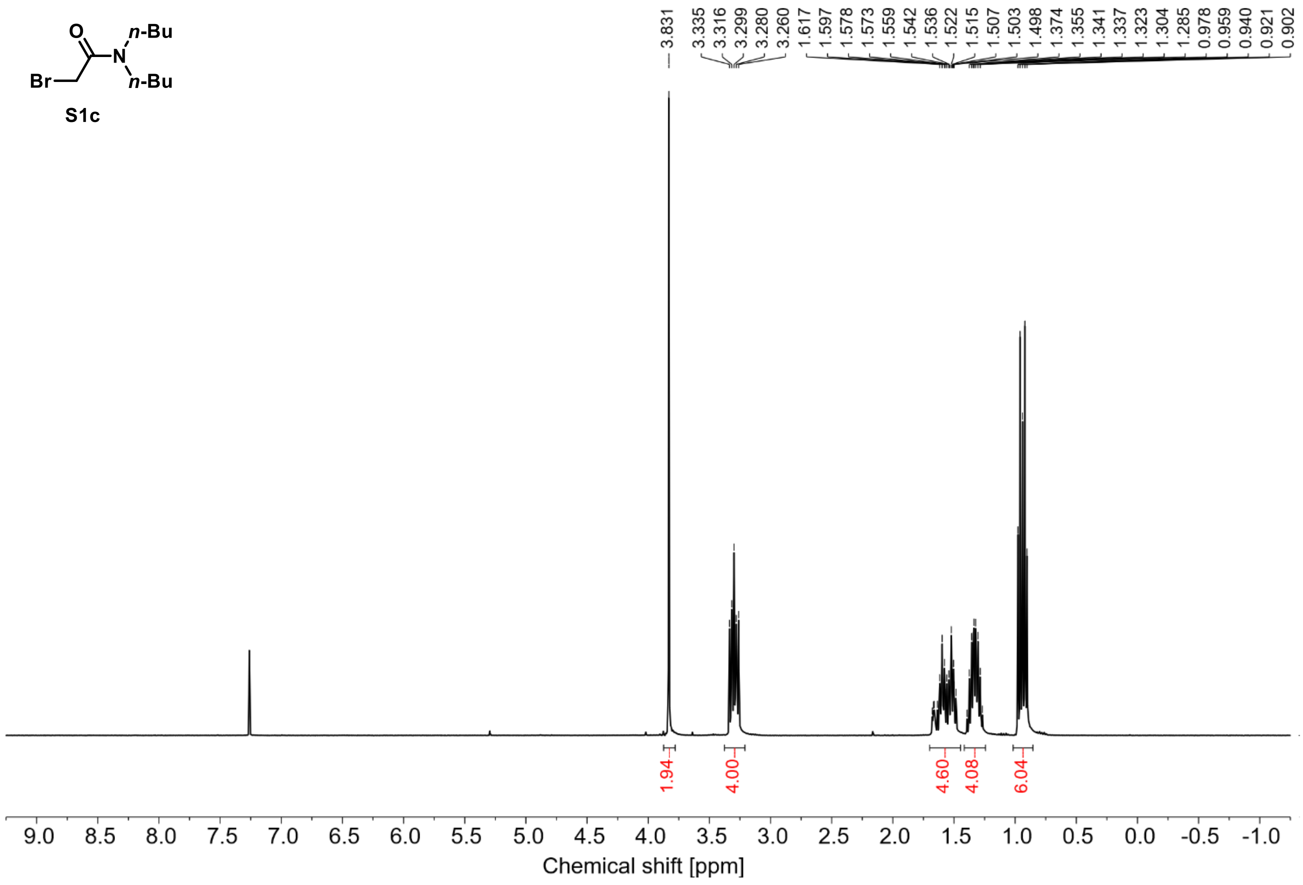

Figure S154. ^{1}H NMR spectrum of compound **S1c** (400 MHz, $CDCl_3$).

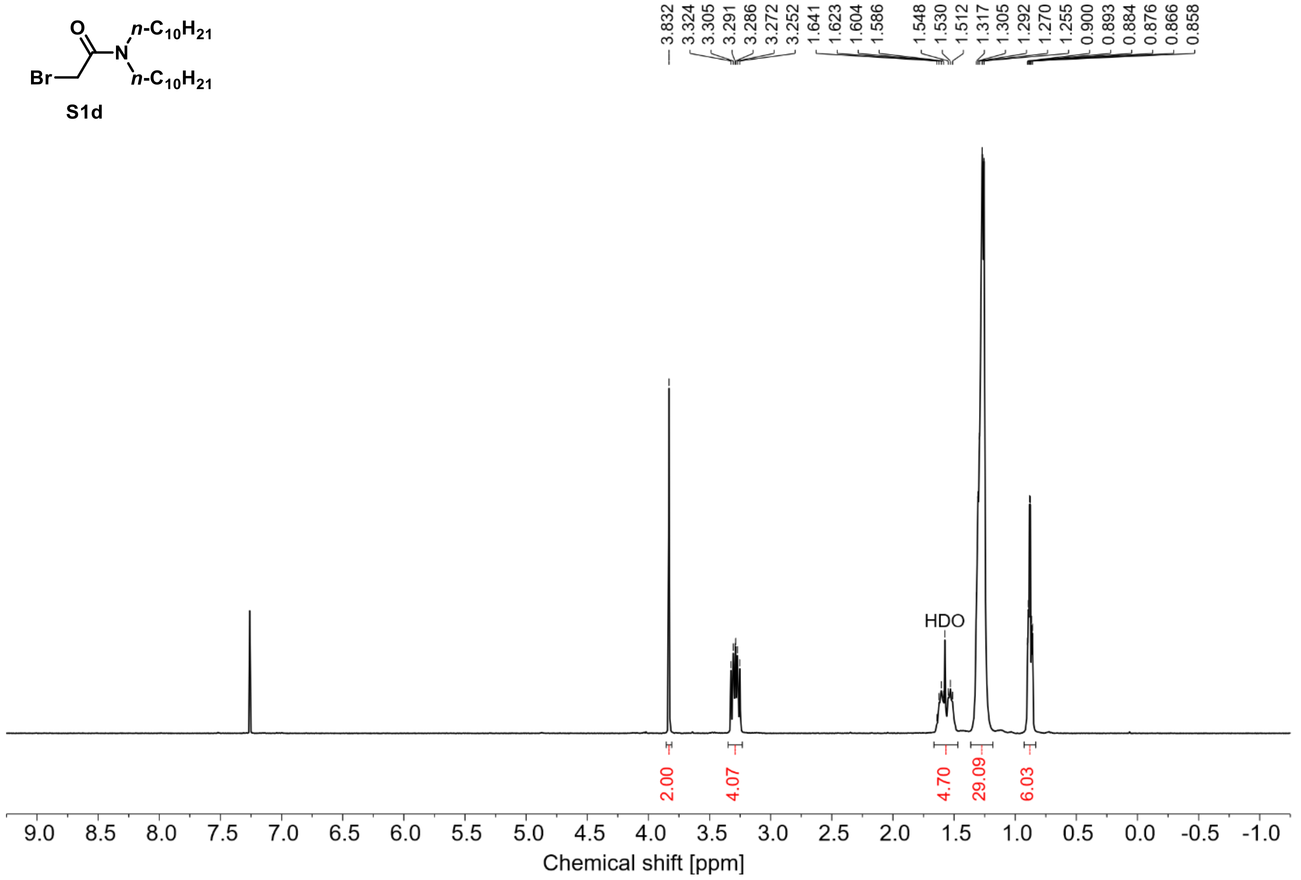

Figure S155. ^{1}H NMR spectrum of compound **S1d** (400 MHz, $CDCl_3$).

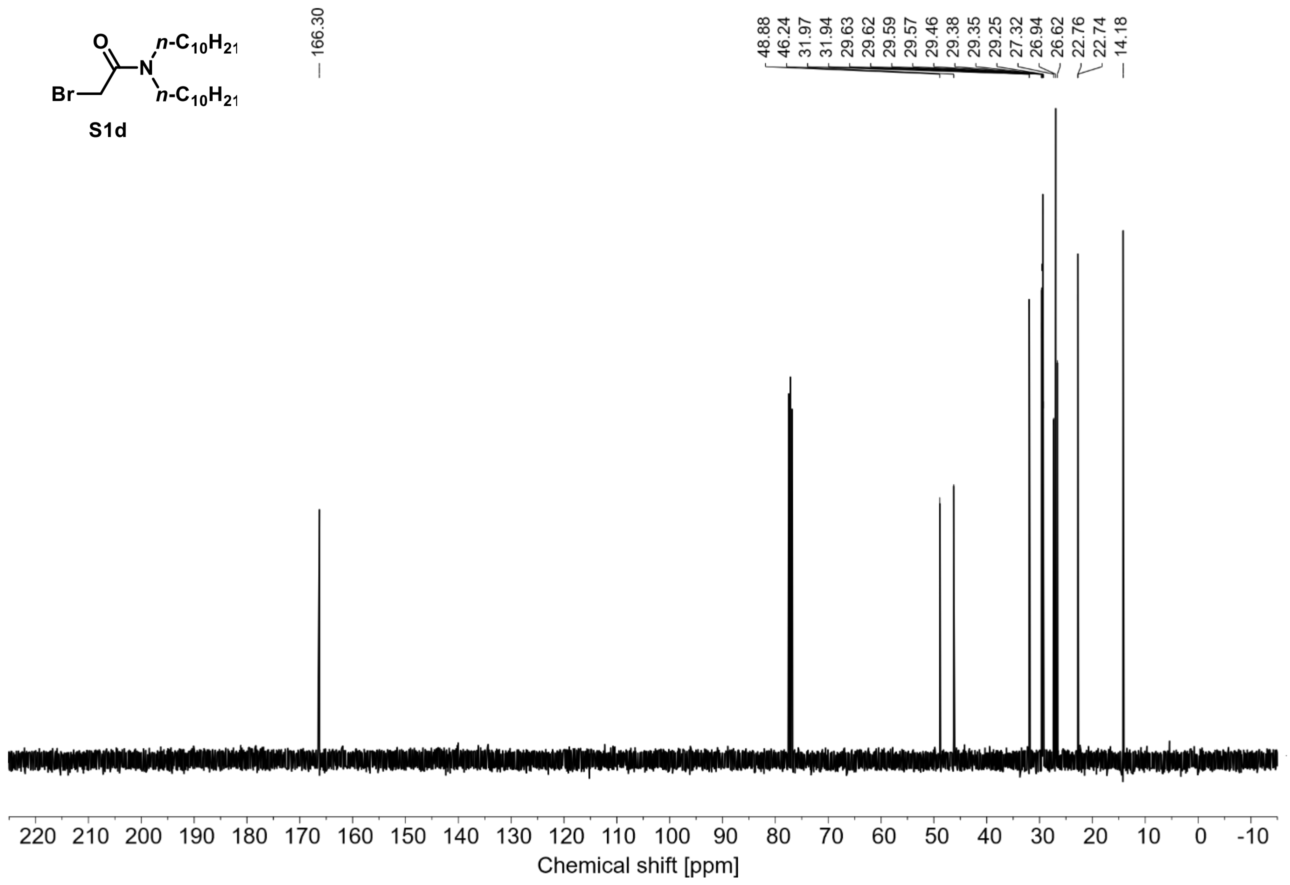

Figure S156. ^{13}C NMR spectrum of compound **S1d** (100 MHz, $CDCl_3$).

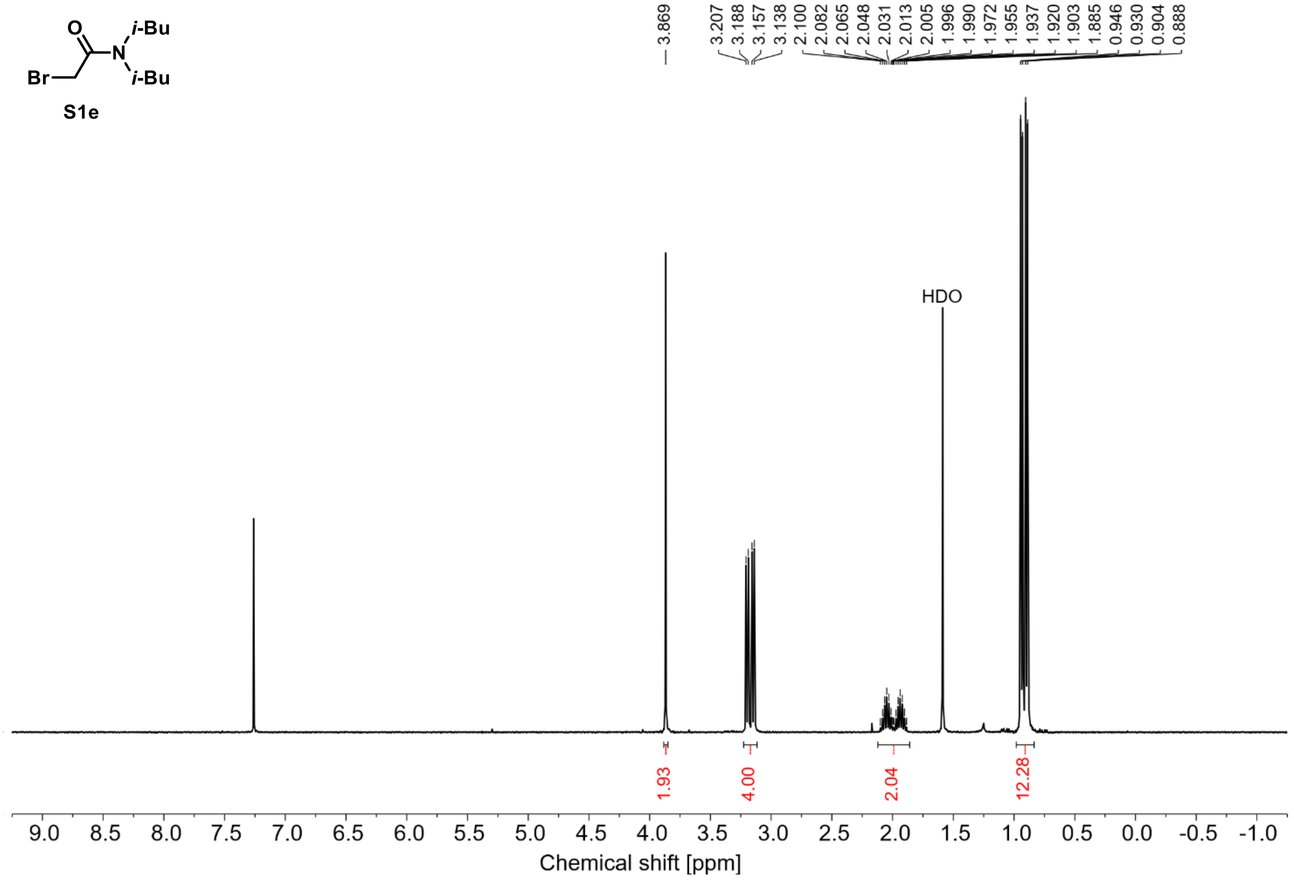

Figure S157. ^{1}H NMR spectrum of compound **S1e** (400 MHz, $CDCl_3$).

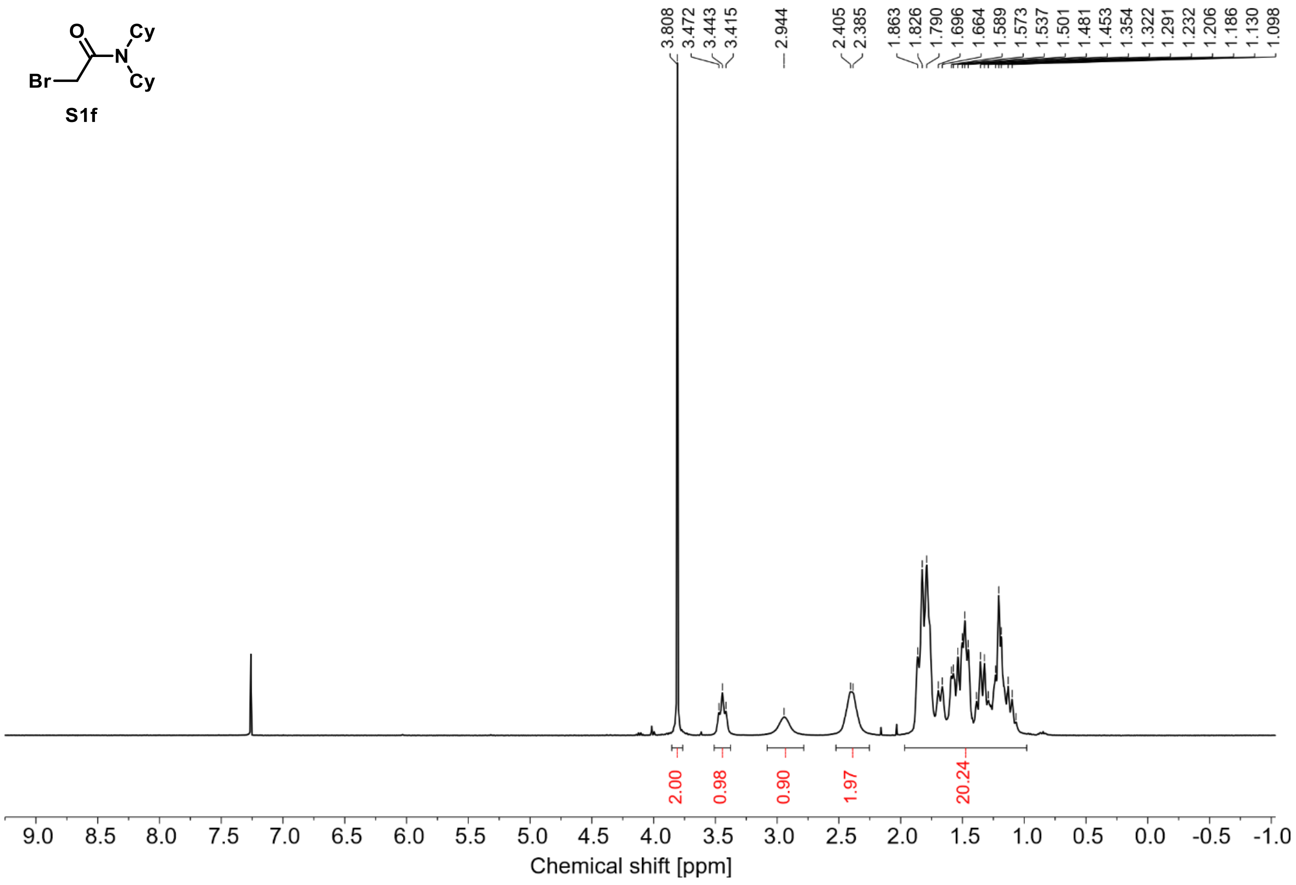

Figure S158. ^{1}H NMR spectrum of compound **S1f** (400 MHz, $CDCl_3$).

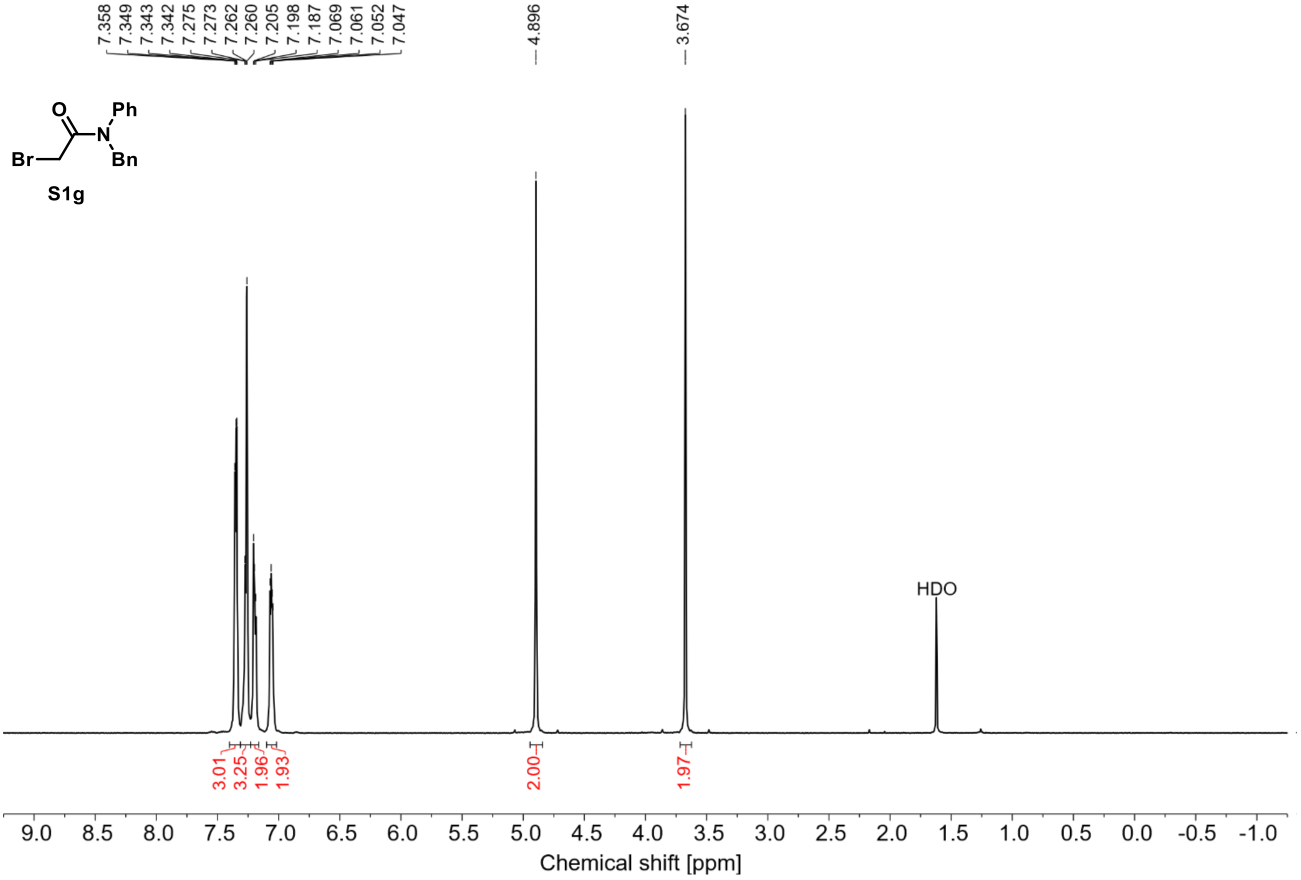

Figure S159. ^{1}H NMR spectrum of compound **S1g** (400 MHz, $CDCl_3$).

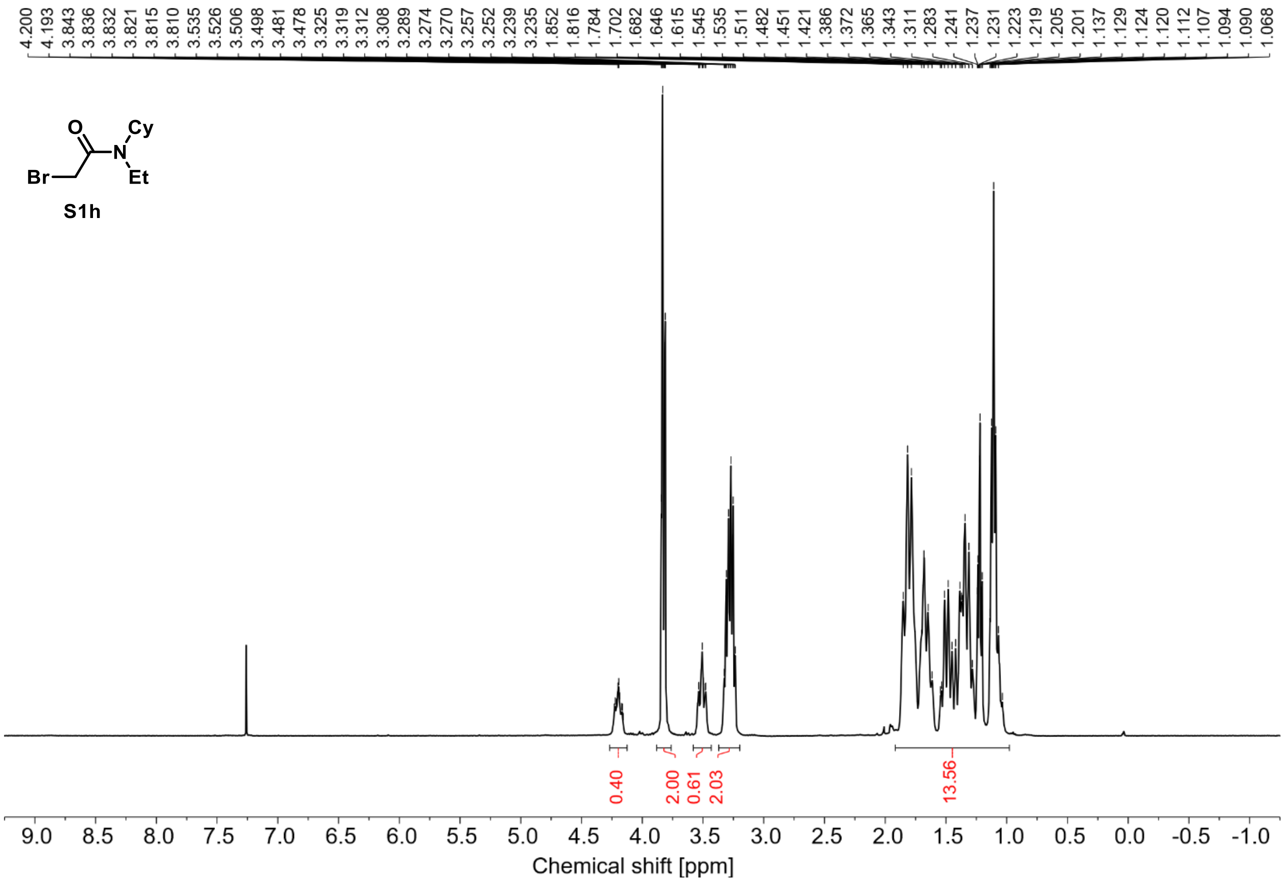

Figure S160. ^{1}H NMR spectrum of compound **S1h** (400 MHz, $CDCl_3$).

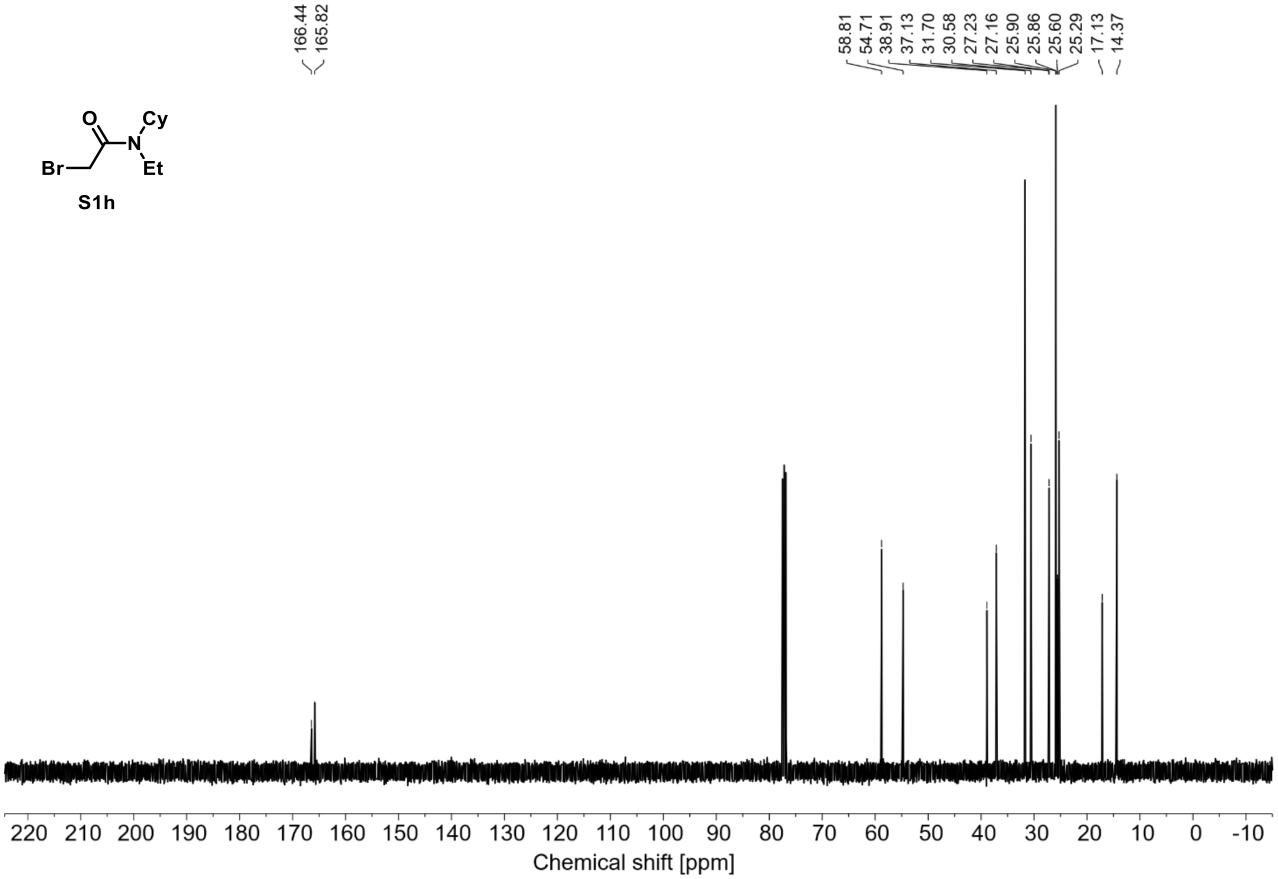

Figure S161. ^{13}C NMR spectrum of compound **S1h** (100 MHz, $CDCl_3$).

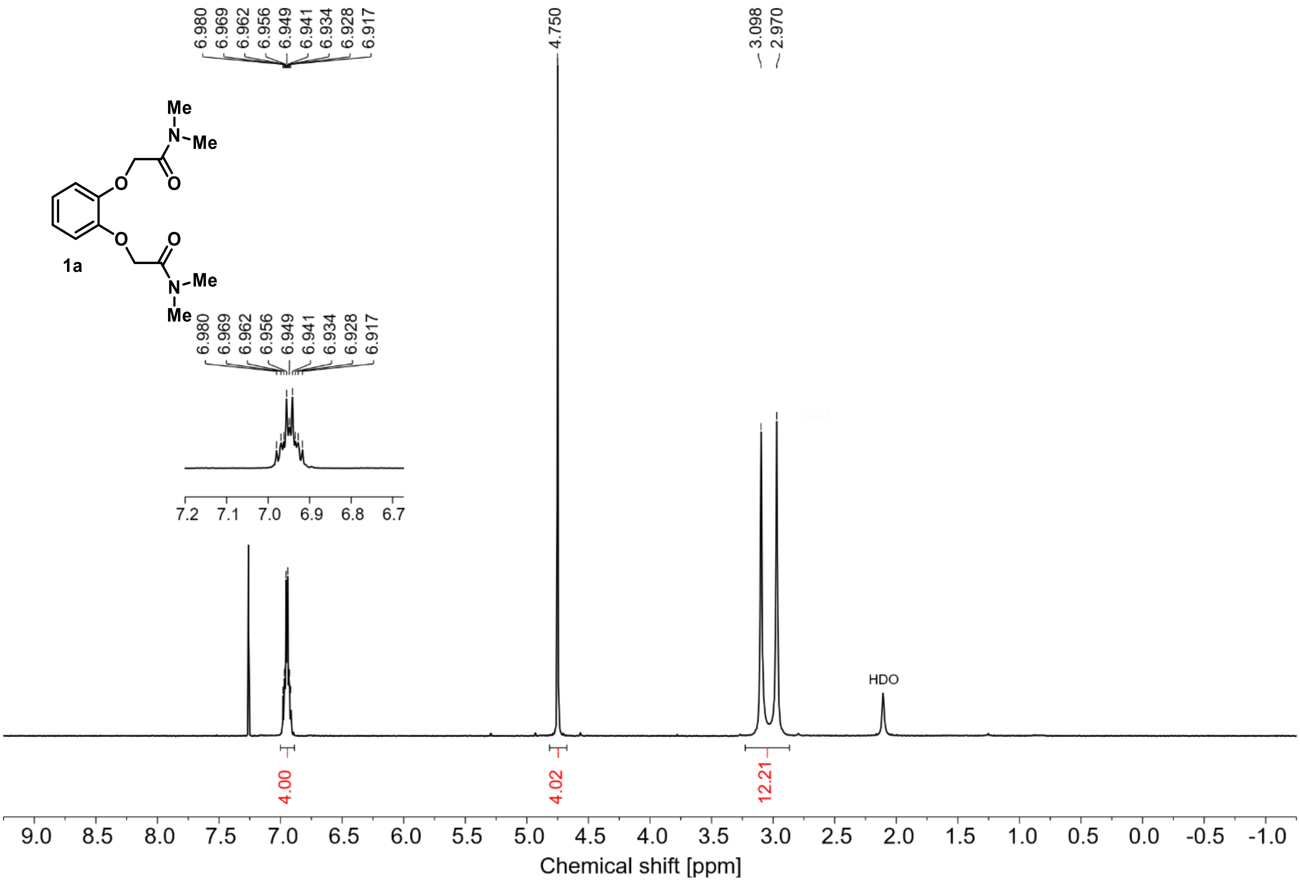

Figure S162. ^{1}H NMR spectrum of compound **1a** (400 MHz, $CDCl_3$).

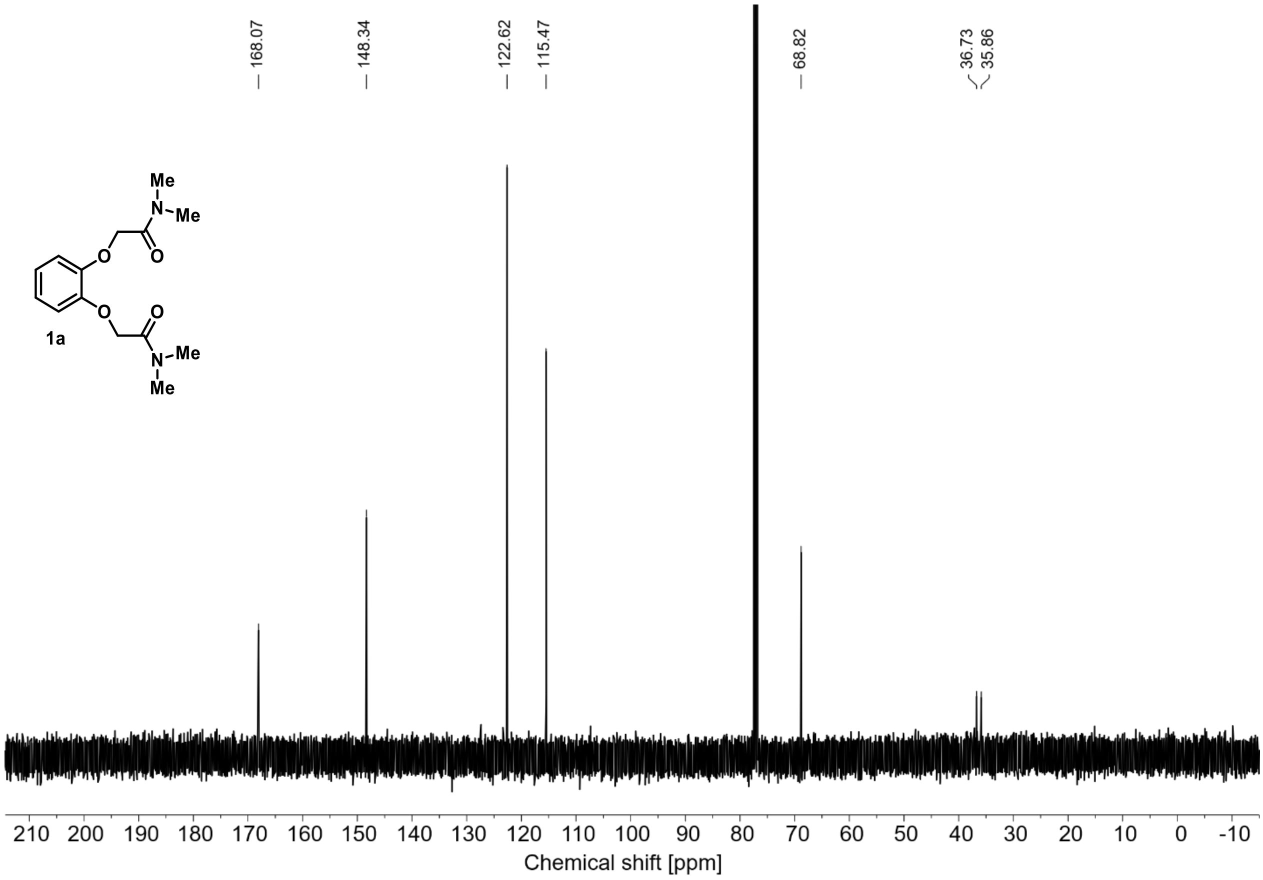

Figure S163. ^{13}C NMR spectrum of compound **1a** (100 MHz, $CDCl_3$).

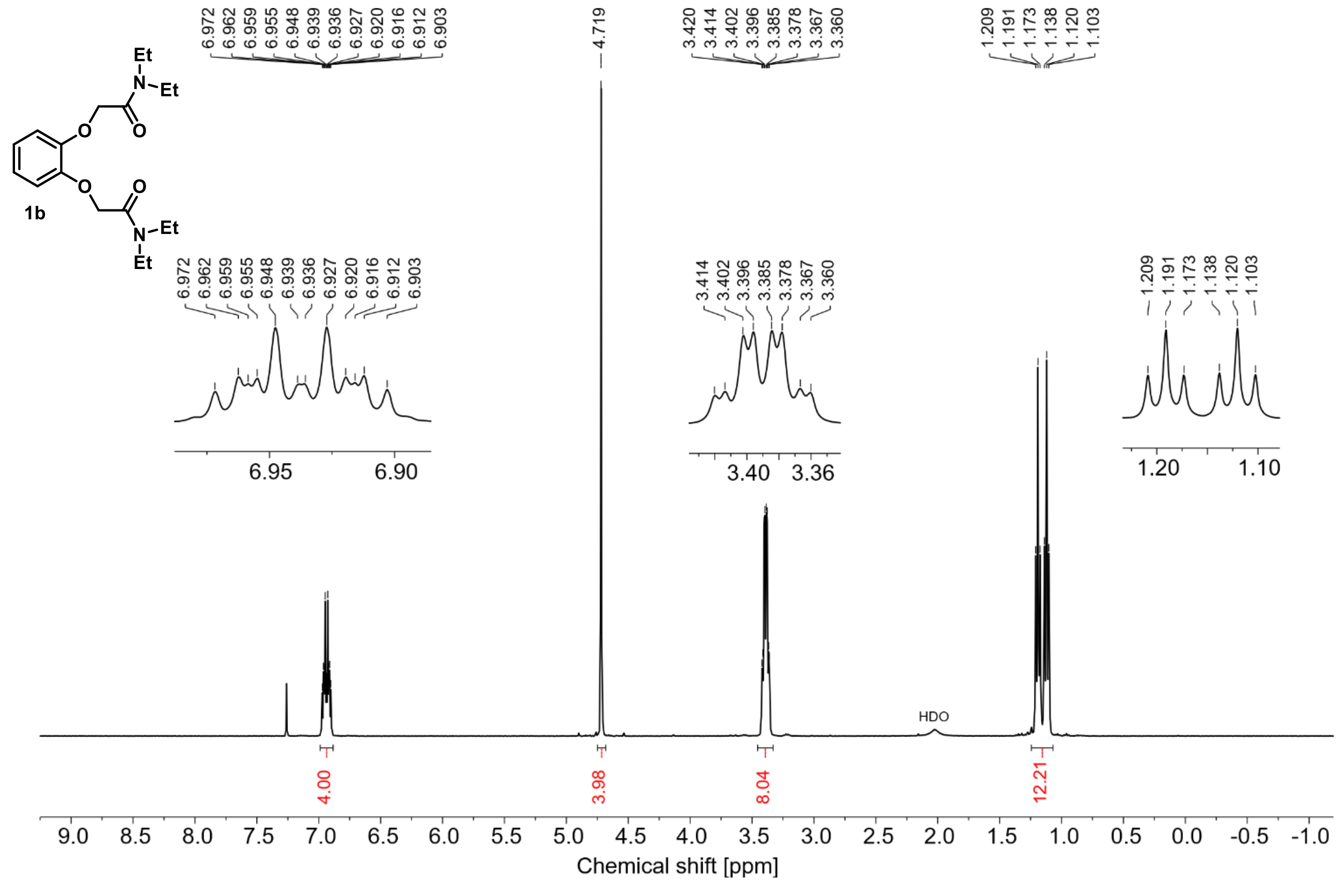

Figure S164. ^{1}H NMR spectrum of compound **1b** (400 MHz, $CDCl_3$).

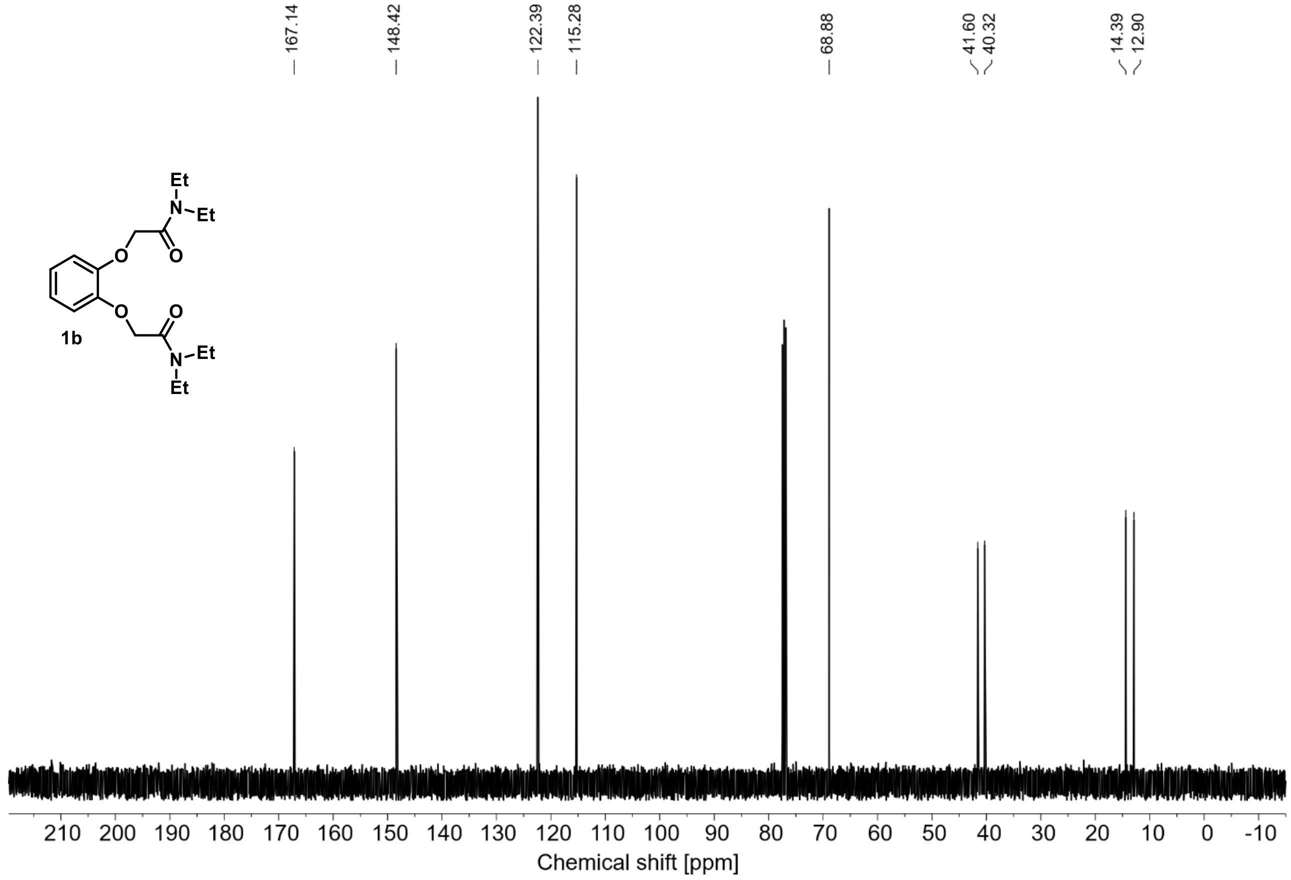

Figure S165. ^{13}C NMR spectrum of compound **1b** (100 MHz, $CDCl_3$).

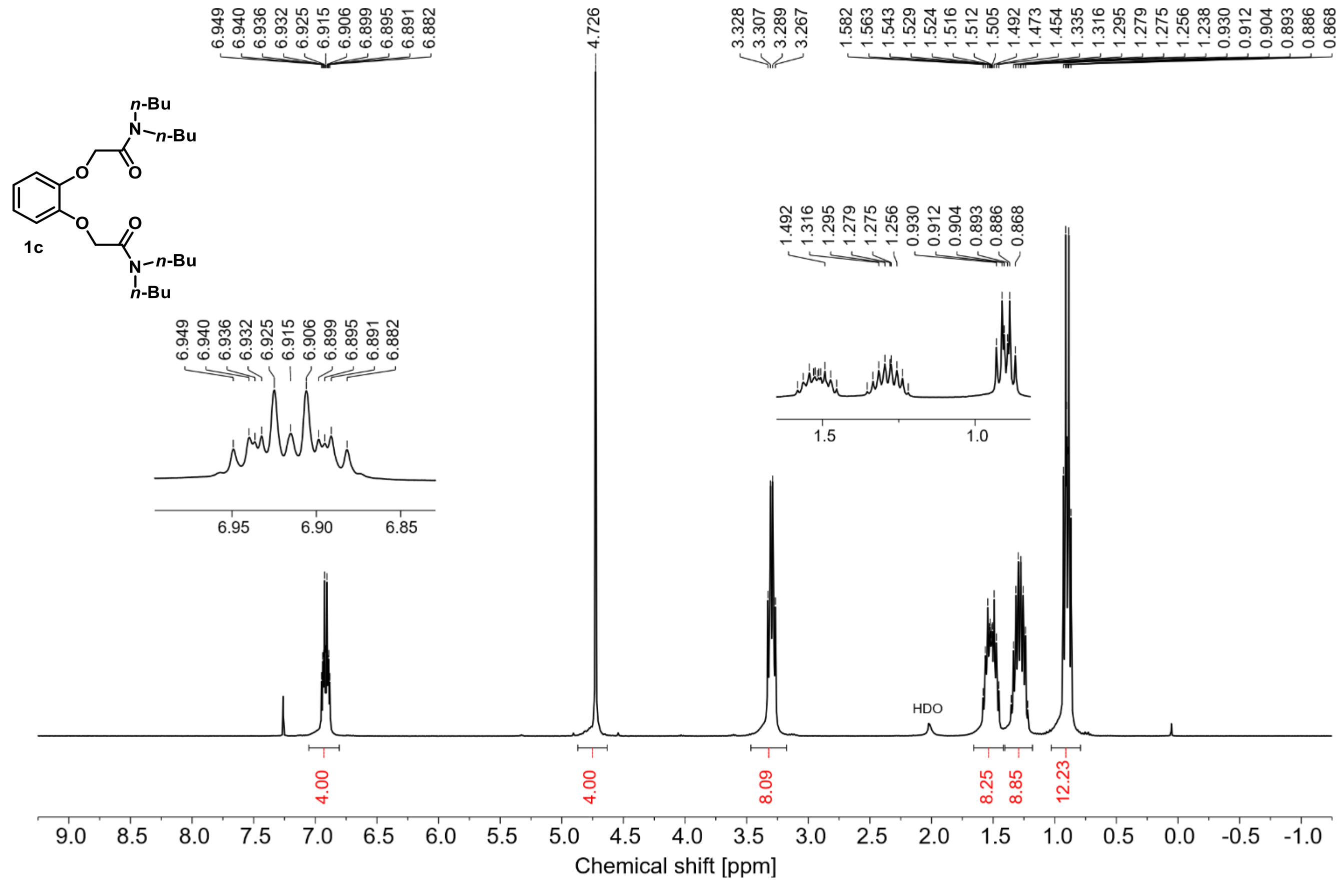

Figure S166. ^{1}H NMR spectrum of compound **1c** (400 MHz, $CDCl_3$).

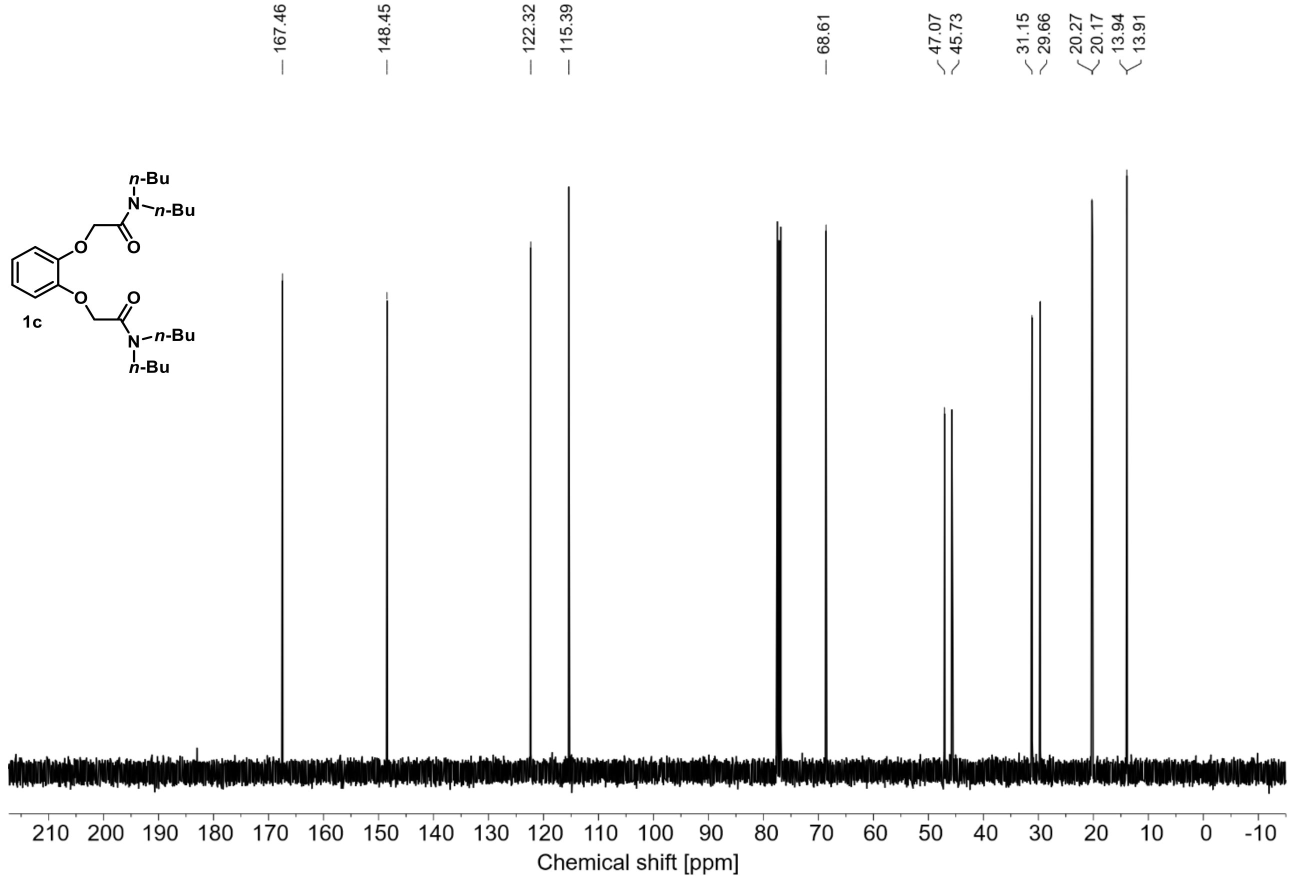

Figure S167. ^{13}C-NMR spectrum of compound **1c** (100 MHz, $CDCl_3$).

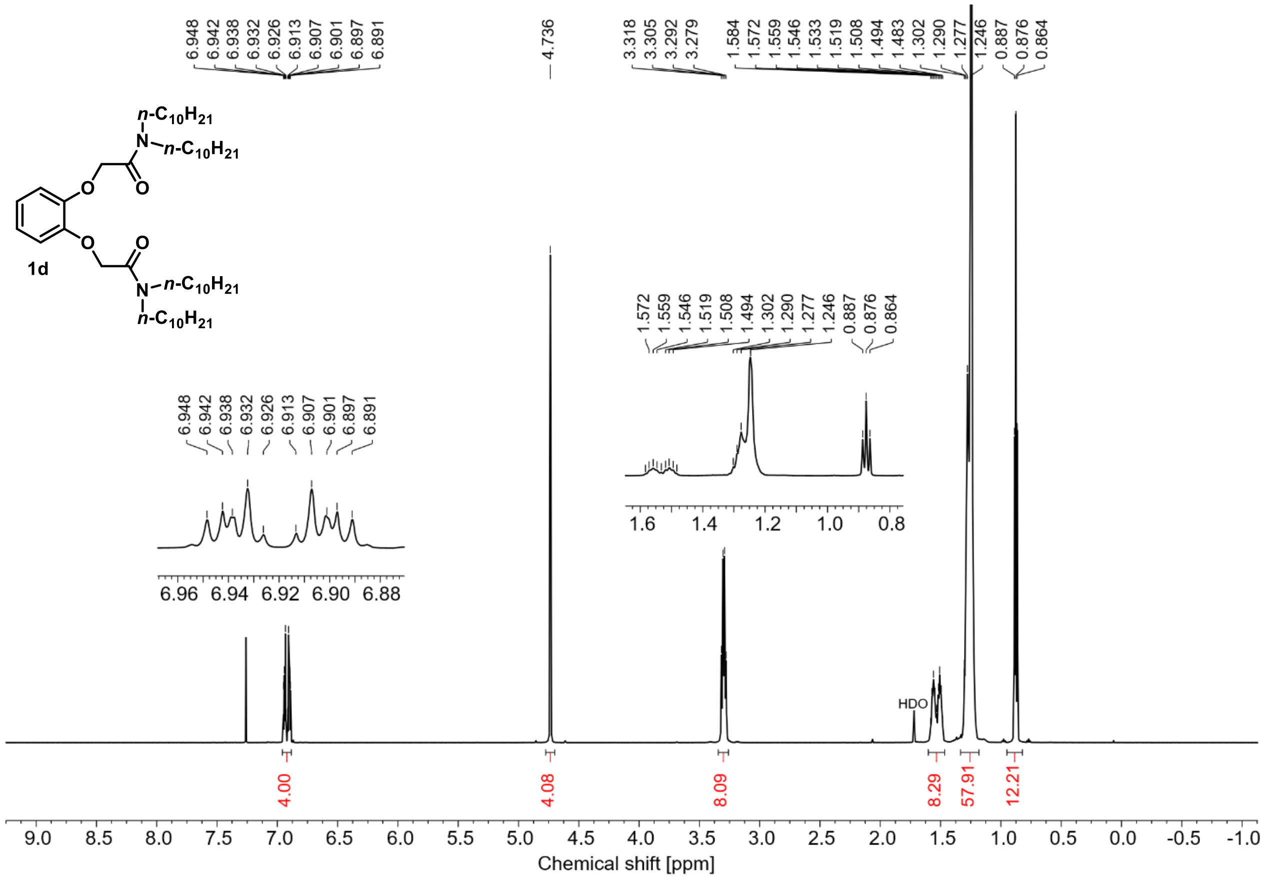

Figure S168. ^{1}H-NMR spectrum of compound **1d** (600 MHz, $CDCl_3$).

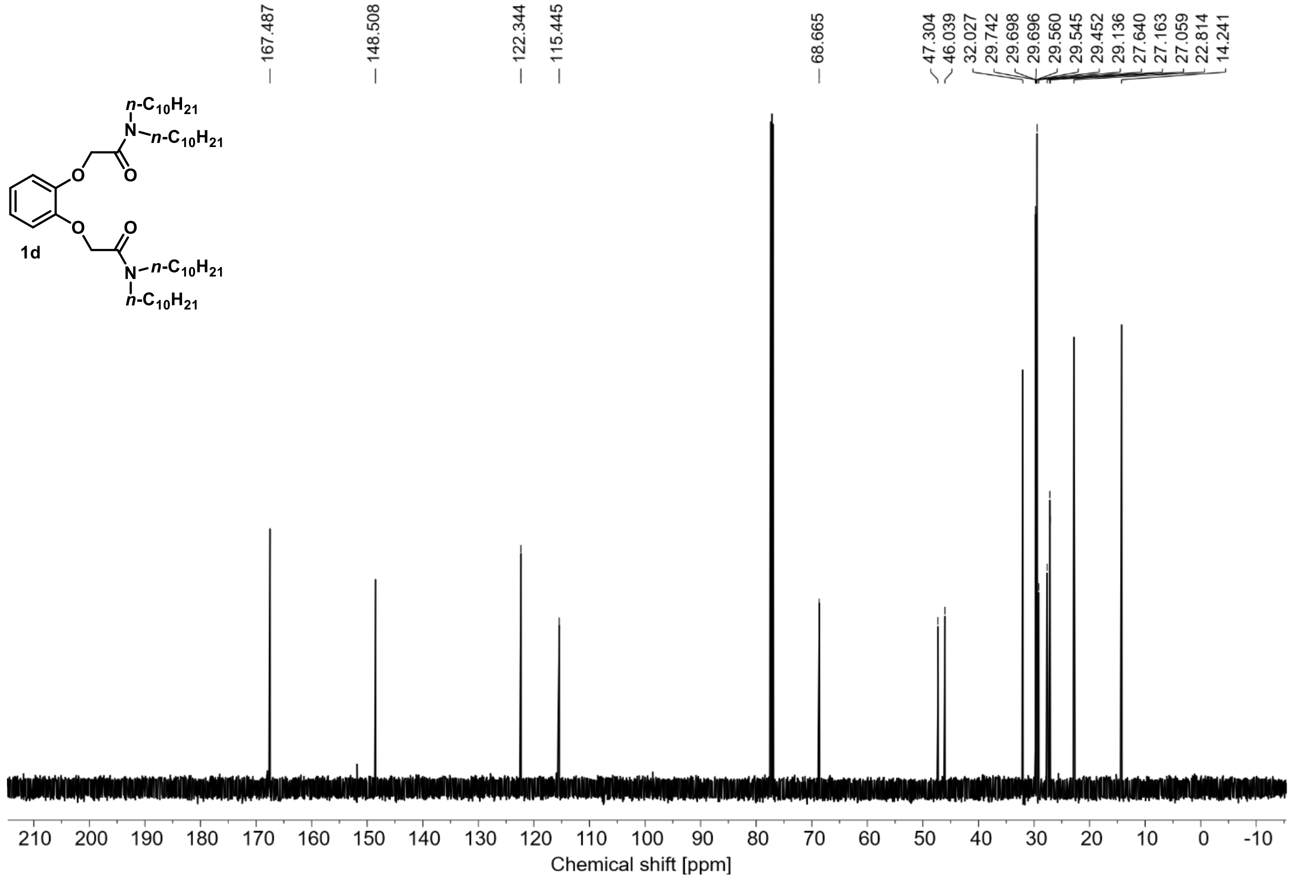

Figure S169. ^{13}C-NMR spectrum of compound **1d** (151MHz, $CDCl_3$).

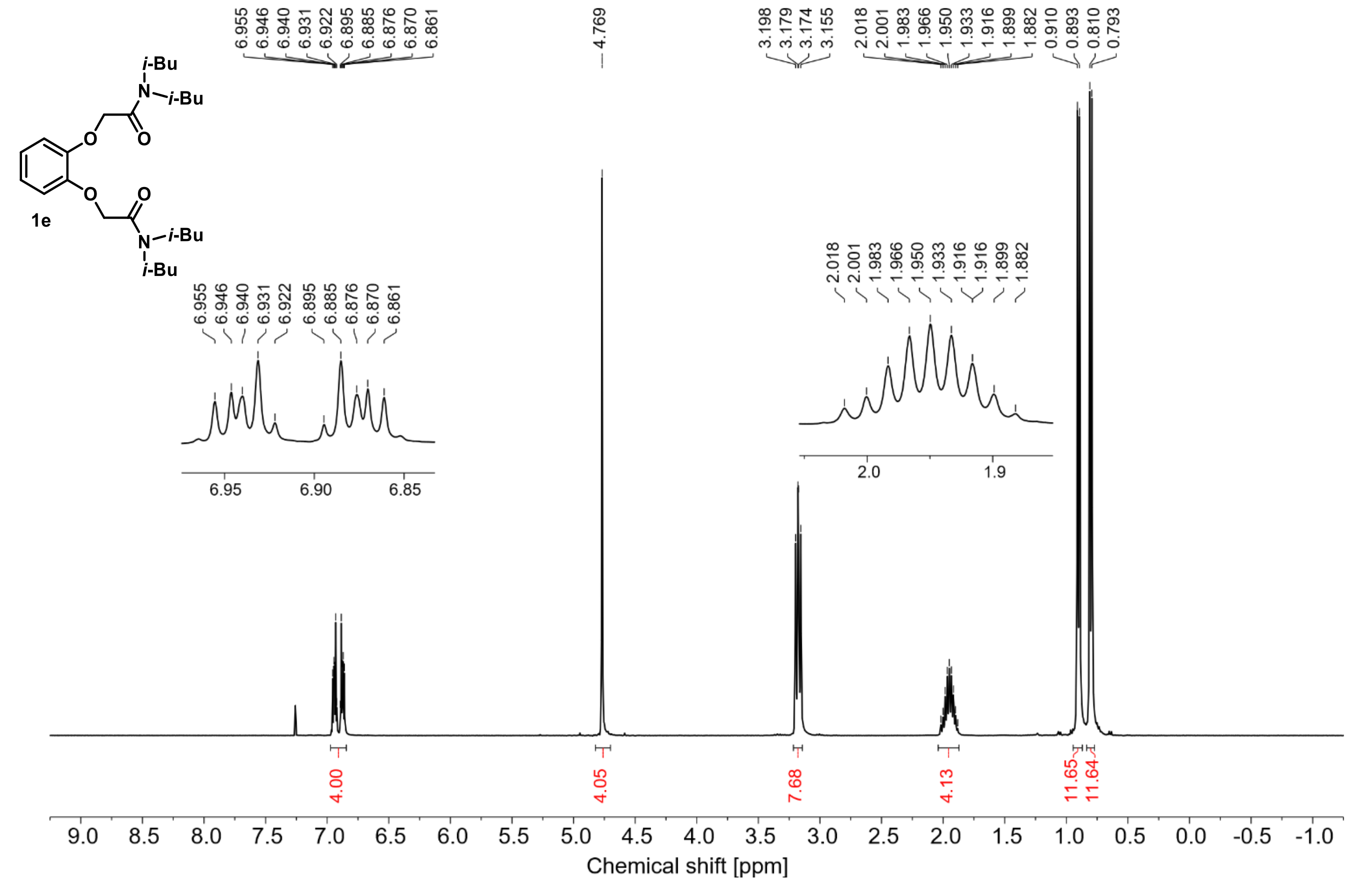

Figure S170. ^{1}H-NMR spectrum of compound **1e** (400 MHz, $CDCl_3$).

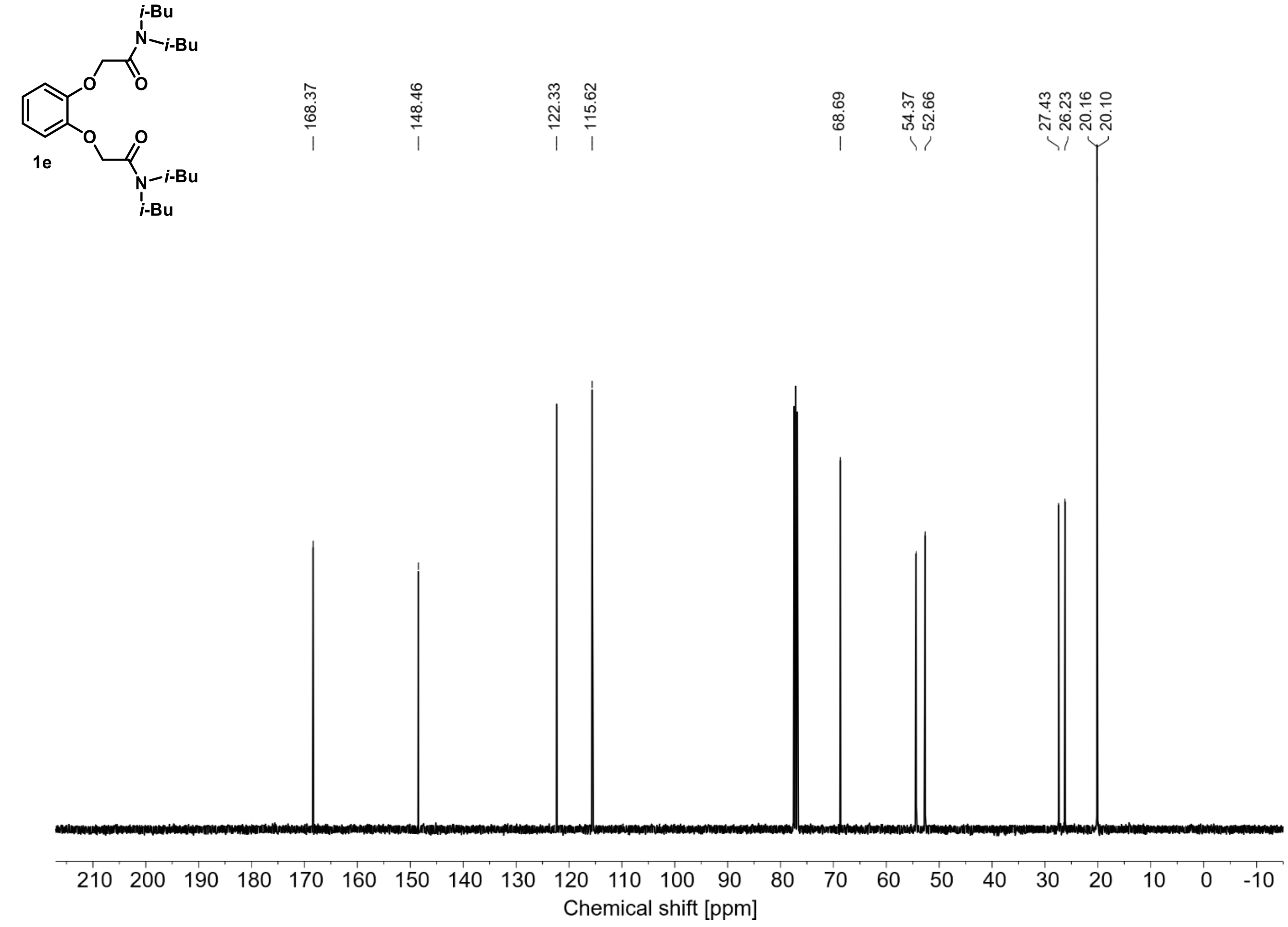

Figure S171. ^{13}C-NMR spectrum of compound **1e** (100 MHz, $CDCl_3$).

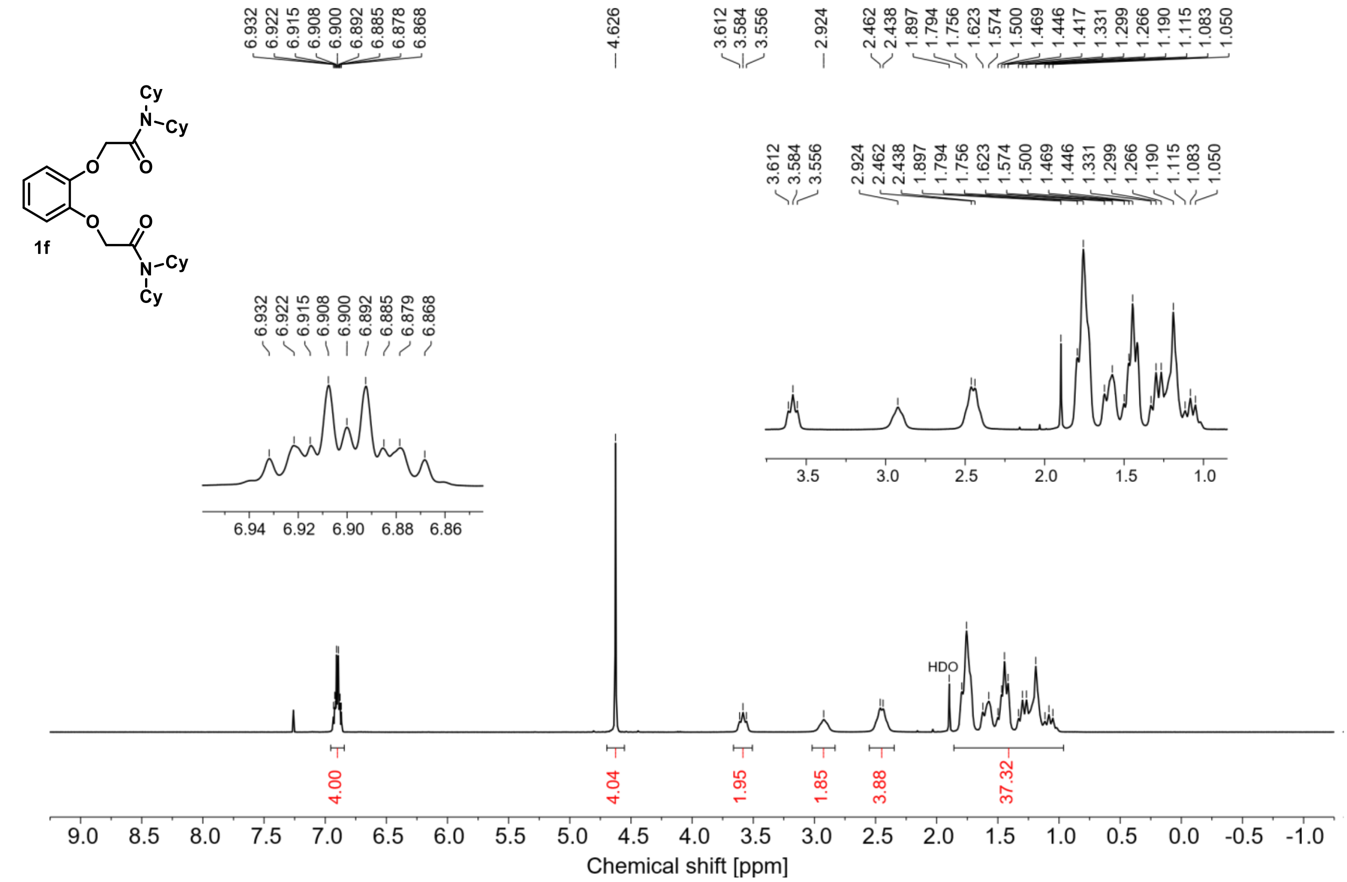

Figure S172. ^{1}H-NMR spectrum of compound **1f** (400 MHz, $CDCl_3$).

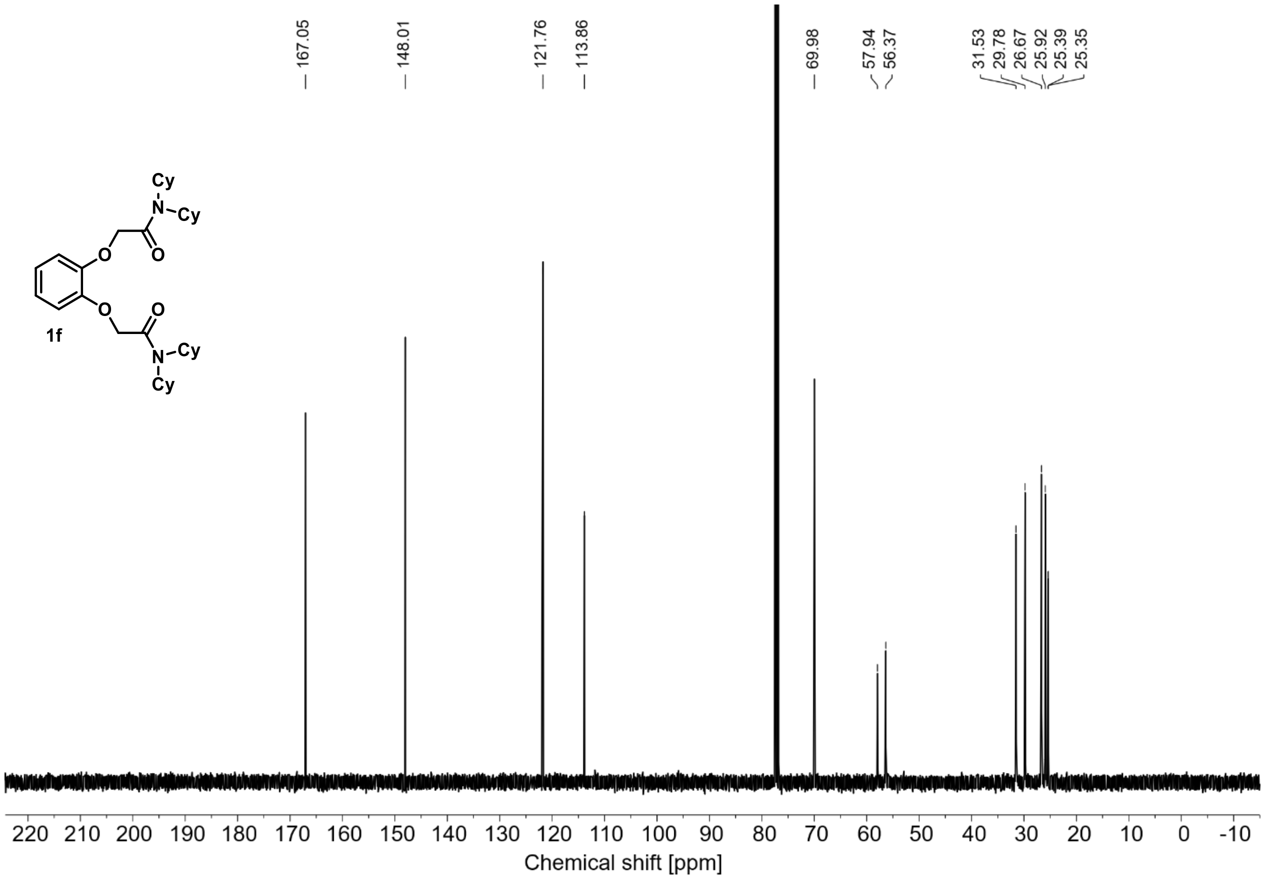

Figure S173. ^{13}C-NMR spectrum of compound **1f** (100 MHz, $CDCl_3$).

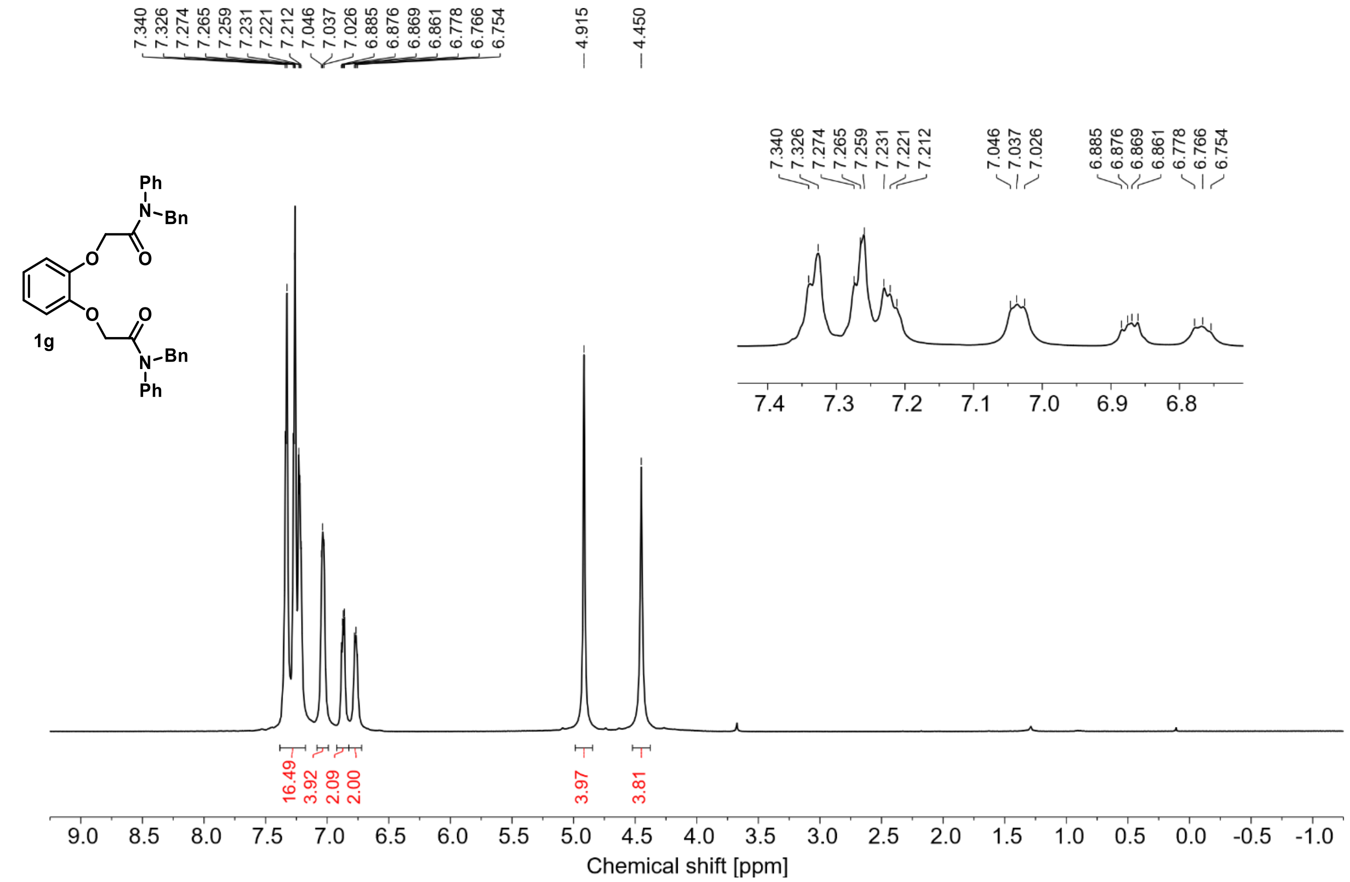

Fig S174. ^{1}H-NMR spectrum of compound **1g** (400 MHz, $CDCl_3$).

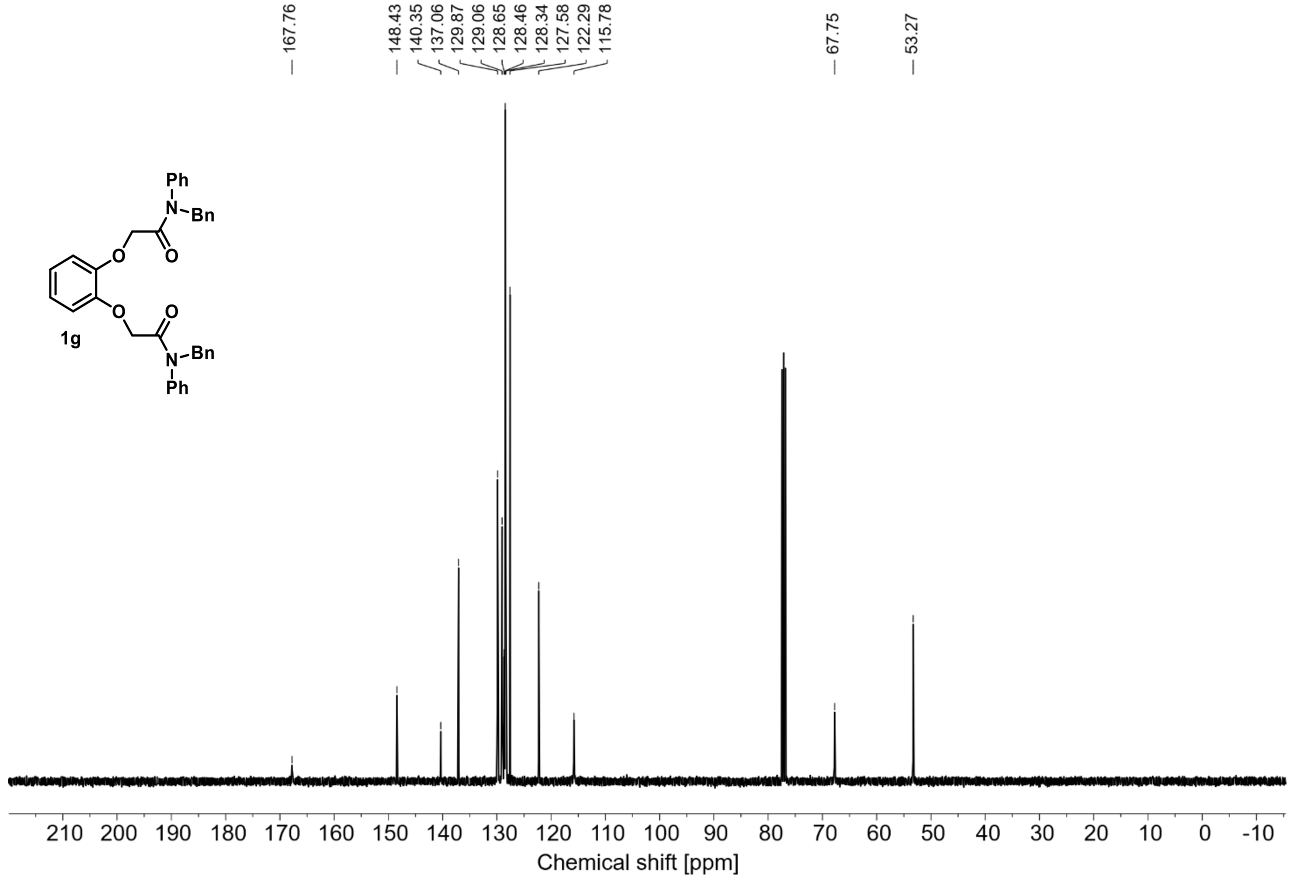

Figure S175. ^{13}C-NMR spectrum of compound **1g** (100 MHz, $CDCl_3$).

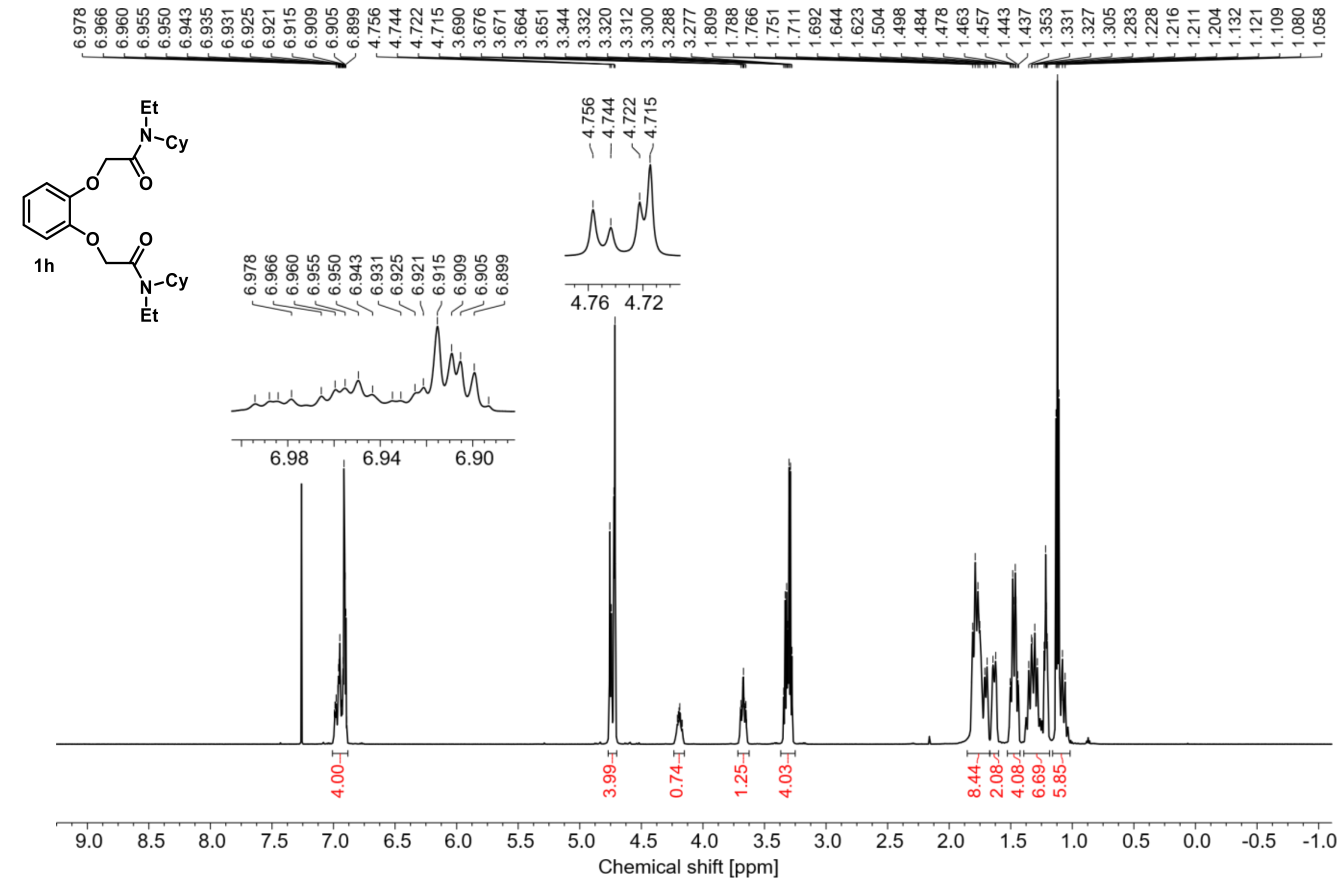

Figure S176. ^{1}H-NMR spectrum of compound **1h** (600 MHz, $CDCl_3$).

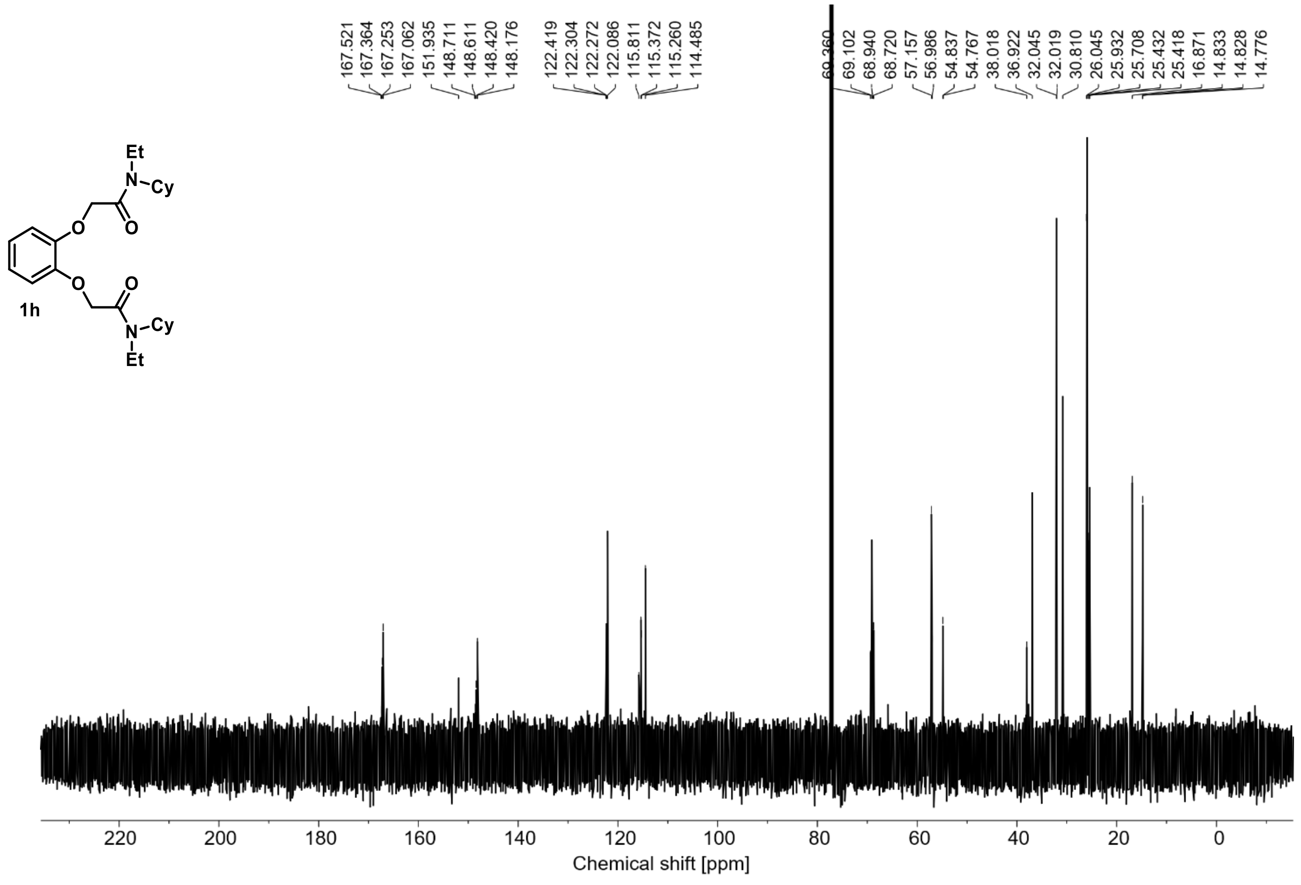

Figure S177. ^{13}C-NMR spectrum of compound **1h** (151MHz, $CDCl_3$).

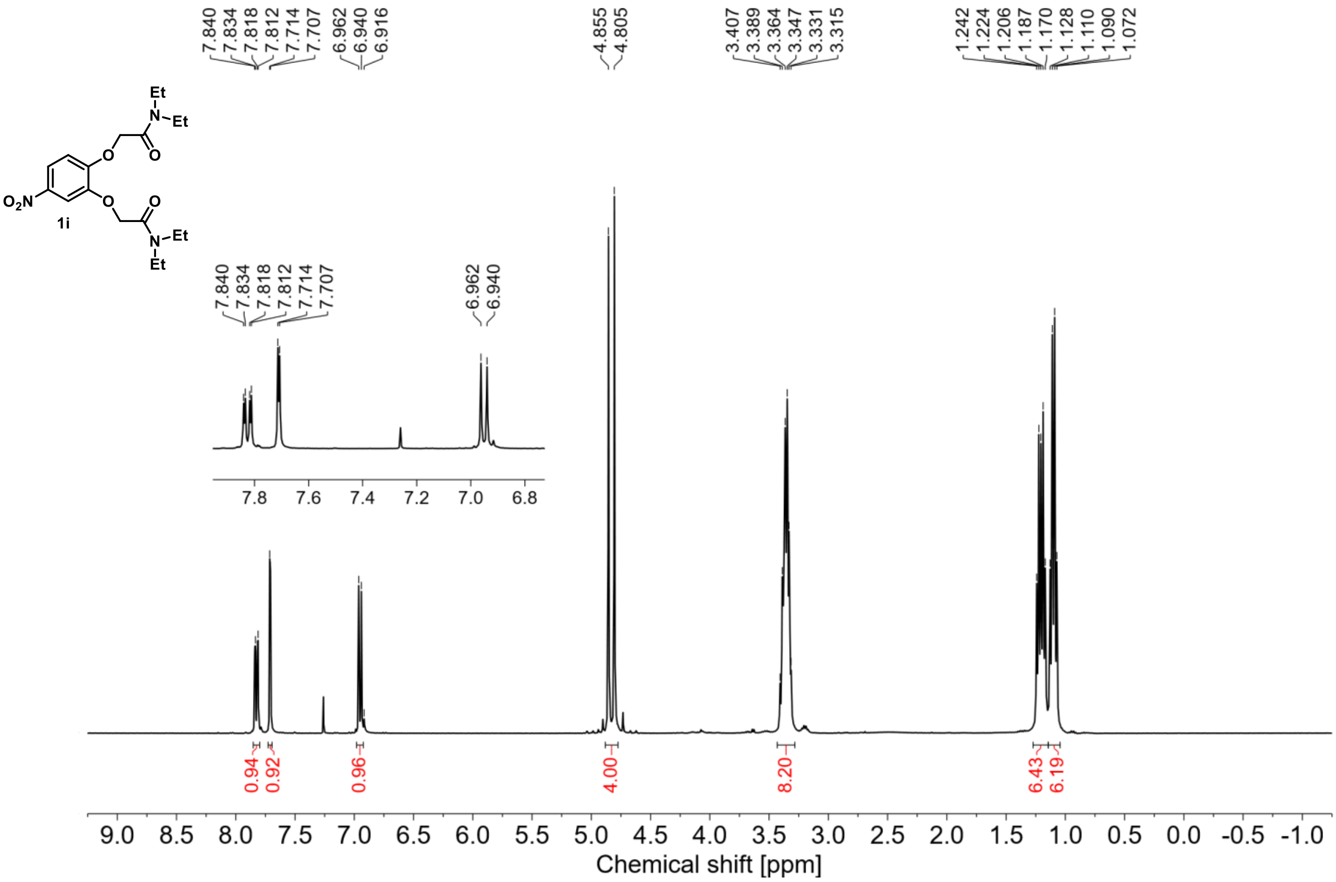

Figure S178. ^{1}H-NMR spectrum of compound **1i** (400 MHz, $CDCl_3$).

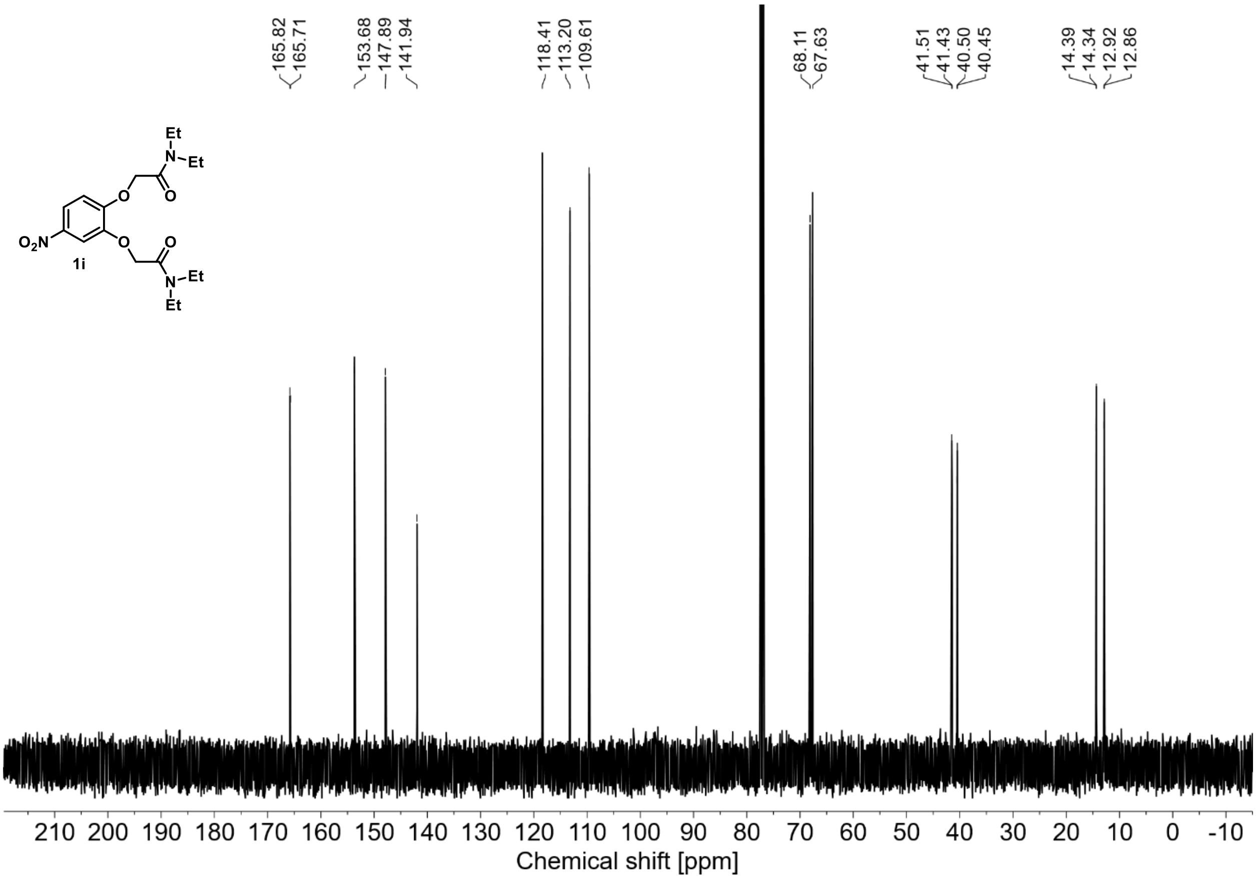

Figure S179. ^{13}C NMR spectrum of compound **1i** (100 MHz, $CDCl_3$).

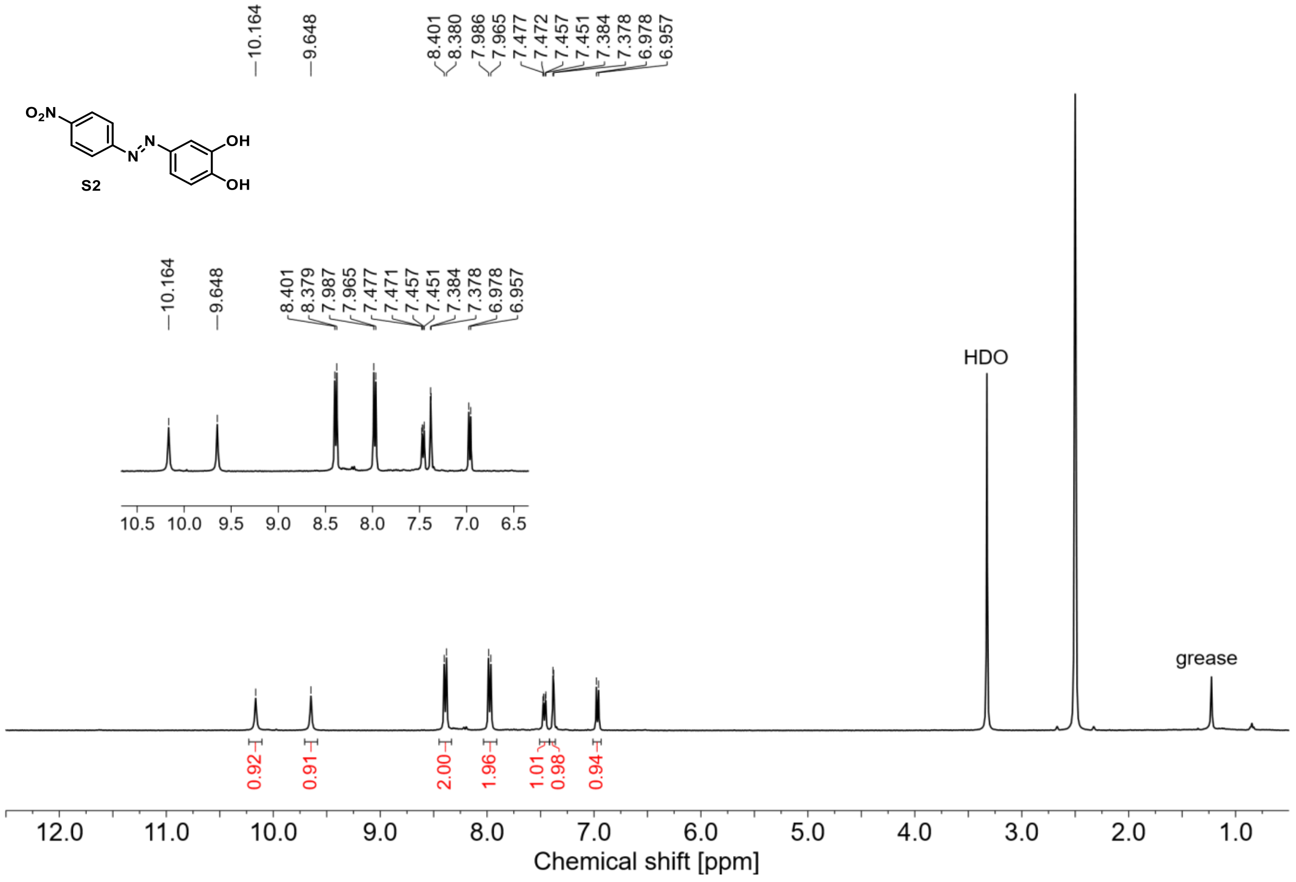

Figure S180. ^{1}H-NMR spectrum of compound **S2** (400 MHz, DMSO-d_6).

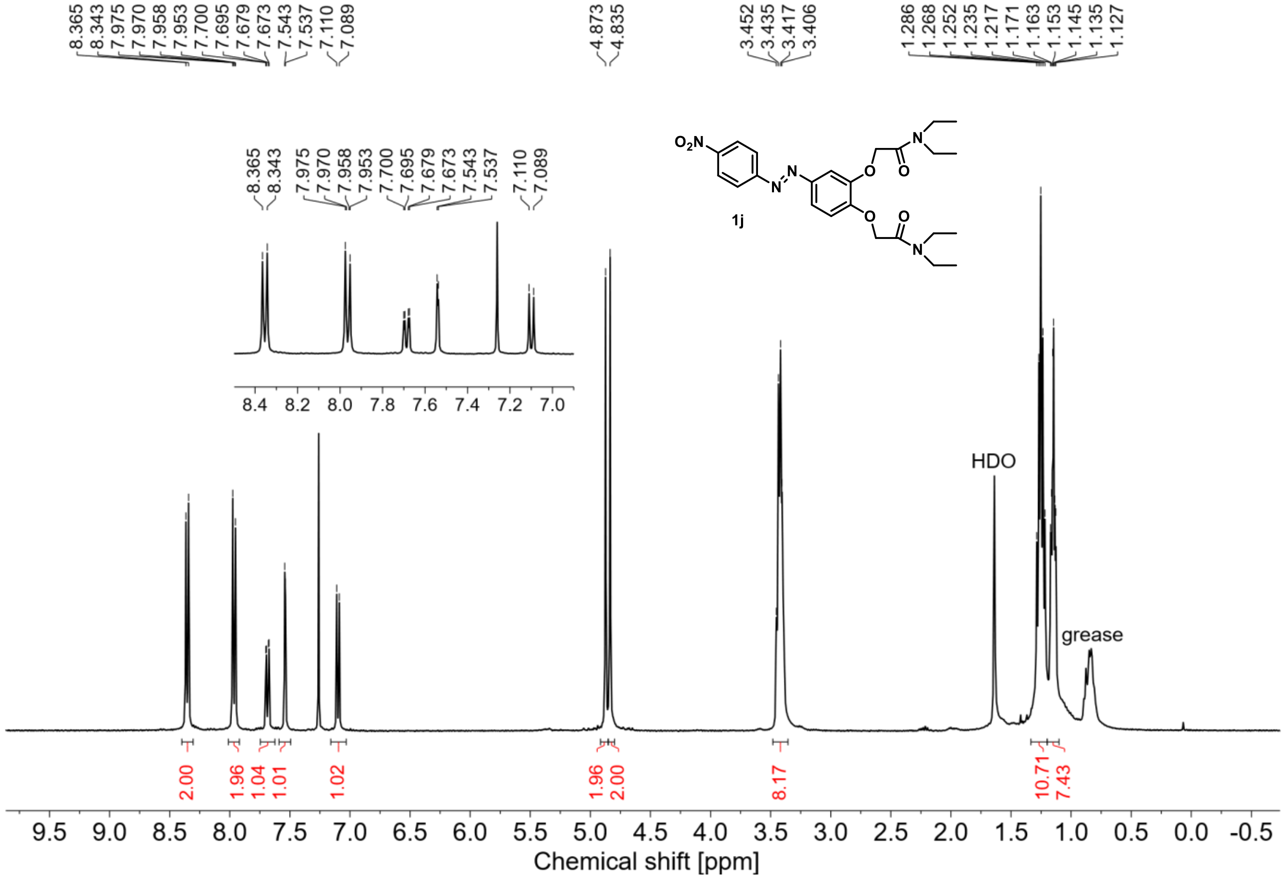

Figure S181. ^{1}H-NMR spectrum of compound **1j** (400 MHz, $CDCl_3$).

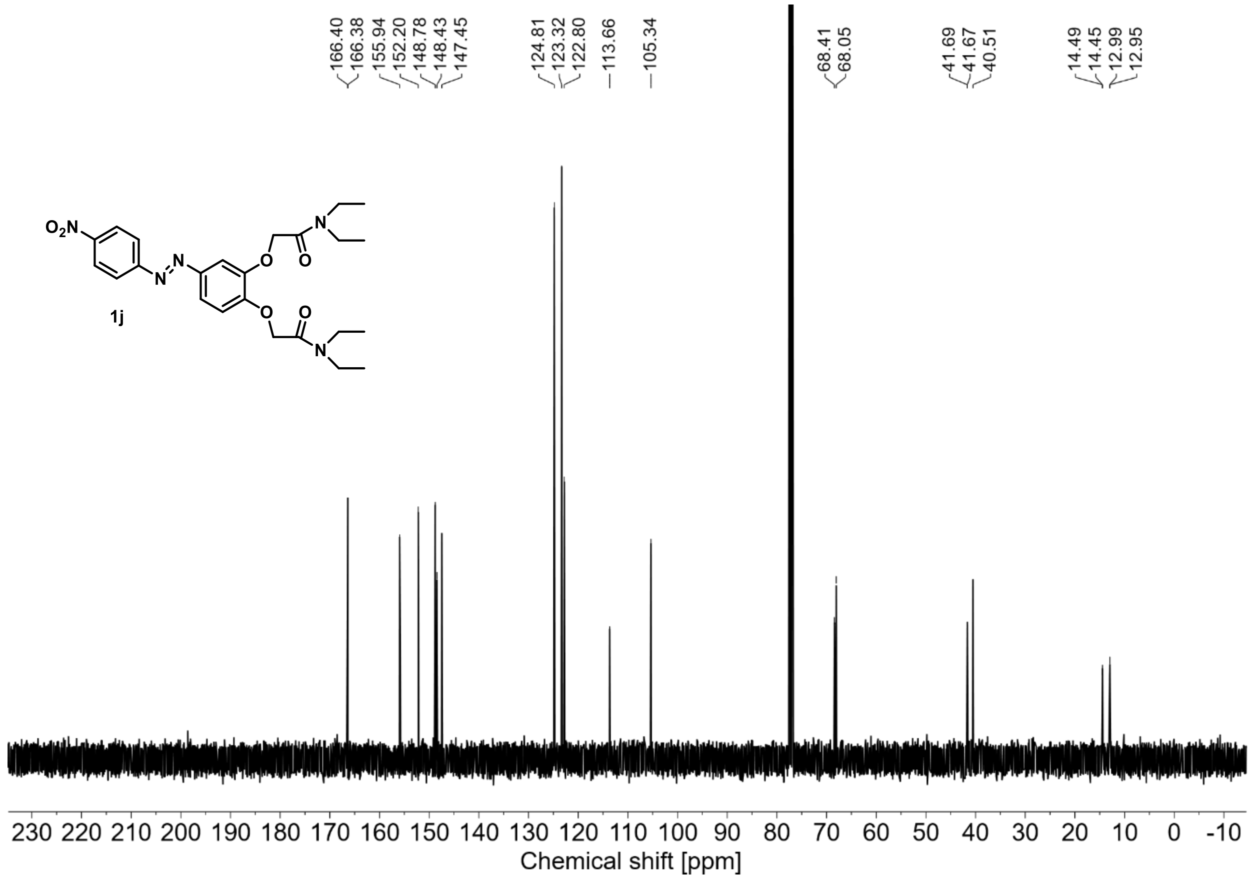

Figure S182. ^{13}C-NMR spectrum of compound **1j** (100 MHz, $CDCl_3$).

8. References

(1) Crapster, J. A.; Guzei, I. A.; Blackwell, H. E. A Peptoid Ribbon Secondary Structure. *Angew. Chem. Int. Ed.* **2013**, *52* (19), 5079-5084.

(2) Hama, T.; Liu, X.; Culkin, D. A.; Hartwig, J. F. Palladium-Catalyzed α-Arylation of Esters and Amides under More Neutral Conditions. *J. Am. Chem. Soc.* **2003**, *125* (37), 11176-11177.

(3) Clarke, L. A.; Ring, A.; Ford, A.; Sinha, A. S.; Lawrence, S. E.; Maguire, A. R. Enantioselective copper catalysed C–H insertion reaction of 2-sulfonyl-2-diazoacetamides to form γ-lactams. *Org. Biomol. Chem.* **2014**, *12* (38), 7612-7628.

(4) Pulukkody, K. P.; Norman, T. J.; Parker, D.; Royle, L.; Broan, C. J. Synthesis of charged and uncharged complexes of gadolinium and yttrium with cyclic polyazaphosphinic acid ligands for in vivo applications. *J. Chem. Soc., Perkin Trans. 2* **1993**, (4), 605-620.

(5) Huang, S.-Y.; Wang, H.; Celio, H.; Khashab, N. M.; Page, Z. A.; Sessler, J. L. Polystyrene-supported neutral lithium receptor for the recovery of high-purity LiPF6 from simulated degraded electrolyte. *J. Mater. Chem. A.* **2022**, *10* (28), 14788-14794.

(6) Hurst, T. E.; Gorman, R. M.; Drouhin, P.; Perry, A.; Taylor, R. J. K. A Direct C□H/Ar□H Coupling Approach to Oxindoles, Thio-oxindoles, 3,4-Dihydro-1 H-quinolin-2-ones, and 1,2,3,4-Tetrahydroquinolines. *Chem. Eur. J.* **2014**, *20* (43), 14063-14073.

(7) Bessières, M.; Plebanek, E.; Chatterjee, P.; Shrivastava-Ranjan, P.; Flint, M.; Spiropoulou, C. F.; Warszycki, D.; Bojarski, A. J.; Roy, V.; Agrofoglio, L. A. Design, synthesis and biological evaluation of 2-substituted-6-[(4-substituted-1-piperidyl)methyl]-1H-benzimidazoles as inhibitors of ebola virus infection. *Eur. J. Med. Chem.* **2021**, *214*, 113211.

(8) Bailey, K.; Cowling, R.; Tan, E. W.; Webb, D. A colormetric assay for catechol-O-methyltransferase. *Bioorg. Med. Chem.* **2004**, *12* (3), 595-601.

(9) Mahoney, J. M.; Beatty, A. M.; Smith, B. D. Selective Solid−Liquid Extraction of Lithium Halide Salts Using a Ditopic Macrobicyclic Receptor. *Inorg. Chem.* **2004**, *43* (24), 7617-7621.

(10) He, Q.; Zhang, Z.; Brewster, J. T.; Lynch, V. M.; Kim, S. K.; Sessler, J. L. Hemispherand-Strapped Calix[4]pyrrole: An Ion-pair Receptor for the Recognition and Extraction of Lithium Nitrite. *J. Am. Chem. Soc.* **2016**, *138* (31), 9779-9782.

(11) He, Q.; Williams, N. J.; Oh, J. H.; Lynch, V. M.; Kim, S. K.; Moyer, B. A.; Sessler, J. L. Selective Solid–Liquid and Liquid–Liquid Extraction of Lithium Chloride Using Strapped Calix[4]pyrroles. *Angew. Chem. Int. Ed.* **2018**, *57* (37), 11924-11928.

(12) Gohil, H.; Chatterjee, S.; Yadav, S.; Suresh, E.; Paital, A. R. An Ionophore for High Lithium Loading and Selective Capture from Brine. *Inorg. Chem.* **2019**, *58* (11), 7209-7219.

(13) Hong, K.-I.; Kim, H.; Kim, Y.; Choi, M.-G.; Jang, W.-D. Strapped calix[4]pyrrole as a lithium salts selective receptor through separated ion-pair binding. *Chem. Commun.* **2020**, *56* (72), 10541-10544.

(14) Tse, Y. C.; Docker, A.; Zhang, Z.; Beer, P. D. Lithium halide ion-pair recognition with halogen bonding and chalcogen bonding heteroditopic macrocycles. *Chem. Commun.* **2021**, *57* (40), 4950-4953.

(15) Wang, H.; Jones, L. O.; Hwang, I.; Allen, M. J.; Tao, D.; Lynch, V. M.; Freeman, B. D.; Khashab, N. M.; Schatz, G. C.; Page, Z. A.; Sessler, J. L. Selective Separation of Lithium Chloride by Organogels Containing Strapped Calix[4]pyrroles. *J. Am. Chem. Soc.* **2021**, *143* (48), 20403-20410.

(16) Yang, J. H.; Kim, J.; Hay, B. P.; Lee, K.; Kim, S. K. Tris(pyridin-2-ylmethyl)amine-Based Ion Pair Receptors for Selective Lithium Salt Recognition. *Eur. J. Org. Chem.* **2022**, *2022* (30), e202200808.

(17) Kim, S. H.; Yeon, Y.; Lee, A.; Lynch, V. M.; He, Q.; Sessler, J. L.; Kim, S. K. Tetraamidoindolyl calix[4]arene as a selective ion pair receptor for LiCl. *Organic Chemistry Frontiers* **2022**, *9* (24), 6888-6893.

(18) Xu, C.; Tran, Q.; Wojtas, L.; Liu, W. Harnessing ion–dipole interactions: a simple and effective approach to high-performance lithium receptors. *Journal of Materials Chemistry A* **2023**, *11* (23), 12214-12222.

(19) Choudhary, N.; Rajpurohit, D.; Saha, A.; Yadav, S.; Tothadi, S.; Ganguly, B.; Ranjan Paital, A. Lithium sequestration from dilute solutions and sea bittern inspired by the self-assembled complexation. *Chem. Eng. J. (Lausanne)* **2023**, *470*, 144408.

(20) Heo, N. J.; Oh, J. H.; Li, A.; Lee, K.; He, Q.; Sessler, J. L.; Kim, S. K. Ion pair extractant selective for LiCl and LiBr. *Chemical Science* **2024**, *15* (34), 13958-13965.

(21) Jagleniec, D.; Kopeć, A.; Dobrzycki, Ł.; Romański, J. A Squaramide-Crown Ether-Based Receptor and Polymer for Enhanced Lithium Chloride Extraction. *Inorg. Chem.* **2024**, *63* (52), 24797-24805.

(22) Mimuro, T.; Yoshida, A.; Kamo, K.; Hirasawa, M.; Kondo, S. I. Highly soluble bisurea derivatives for anion recognition. *Org. Biomol. Chem.* **2023**, *21* (25), 5281-5287.

(23) Mimuro, T.; Yoshino, S.; Hirasawa, M.; Kondo, S.-i. Solid–liquid extraction of lithium chloride with a simple and flexible heteroditopic receptor. *Bull. Chem. Soc. Jpn.* **2025**, *98* (2), uoaf002.

(24) Kondo, S.; Mimuro, T.; Yoshida, A.; Sugawara, R.; Hirasawa, M. Highly concentrated solution of lithium chloride in organic solvents with heteroditopic receptors. *Chem. Lett.* **2024**, *53* (11), upae198.

(25) Hyun Oh, J.; Joong Kim, M.; Choi, J.; Chan Jeong, H.; Kuk Kim, S. An Ion Pair Receptor for Selective Solid-Liquid Extraction of LiCl. *Chem. Eur. J.* **2025**, *31* (7), e202403868.

(26) Mimuro, T.; Sugawara, R.; Hirasawa, M.; Kondo, S. I. Low-Viscosity Concentrated Lithium Chloride Solution with Unsymmetrical Ditopic Receptors in Organic Solvents. *ChemPhysChem* **2025**, e202500601.

(27) Du, Y.; Ghosh, A.; Teeuwen, P. C. P.; Wales, D. J.; Nitschke, J. R. Light-Driven Lithium Extraction from Mixtures of Alkali Cations Using an Azobipyridine Ligand. *J. Am. Chem. Soc.* **2025**, *147* (24), 20205-20211.

(28) Khianjinda, T.; Vigromsitdet, S.; Srisawat, P.; Sawektreeratana, N.; Tantirungrotechai, J.; Sukwattanasinitt, M.; Harding, D. J.; Beer, P. D.; Tantirungrotechai, Y.; Bunchuay, T. Enhanced Cooperative Lithium Halide Recognition by Heteroditopic Halogen Bonding (XB) Macrocycles. *Inorg. Chem.* **2026**, *65* (1), 441-453.

(29) Ciura, K.; Dziomba, S.; Nowakowska, J.; Markuszewski, M. J. Thin layer chromatography in drug discovery process. *Journal of Chromatography A* **2017**, *1520*, 9-22.

(30) Komsta, Ł.; Skibiński, R.; Berecka, A.; Gumieniczek, A.; Radkiewicz, B.; Radoń, M. Revisiting thin-layer chromatography as a lipophilicity determination tool—A comparative study on several techniques with a model solute set. *J. Pharm. Biomed. Anal.* **2010**, *53* (4), 911-918.

(31) Bate-Smith, E. C.; Westall, R. G. Chromatographic behaviour and chemical structure I. Some naturally occuring phenolic substances. *Biochim. Biophys. Acta* **1950**, *4*, 427-440.

(32) Soczewiński, E.; Wachtmeister, C. A. The relation between the composition of certain ternary two-phase solvent systems and RM values. *J. Chromatogr. A* **1962**, *7*, 311-320.

(33) Frassineti, C.; Alderighi, L.; Gans, P.; Sabatini, A.; Vacca, A.; Ghelli, S. Determination of protonation constants of some fluorinated polyamines by means of 13C NMR data processed by the new computer program HypNMR2000. Protonation sequence in polyamines. *Anal. Bioanal. Chem.* **2003**, *376* (7), 1041-1052.

(34) Frassineti, C.; Ghelli, S.; Gans, P.; Sabatini, A.; Moruzzi, M. S.; Vacca, A. Nuclear Magnetic Resonance as a Tool for Determining Protonation Constants of Natural Polyprotic Bases in Solution. *Anal. Biochem.* **1995**, *231* (2), 374-382.

(35) Soloviev, D. O.; Hunter, C. A. Musketeer: a software tool for the analysis of titration data. *Chemical Science* **2024**, *15* (37), 15299-15310.

(36) Version 1.171.35.11 ed.; Agilent Technologies: 2011.

(37) Sheldrick, G. Crystal structure refinement with SHELXL. *Acta Crystallogr., Sect. C: Cryst. Struct. Commun.* **2015**, *71* (1), 3-8.

(38) Sheldrick, G. A short history of SHELX. *Acta Crystallogr. Sect. A* **2008**, *64* (1), 112-122.

(39) Avram, L.; Cohen, Y. The Role of Water Molecules in a Resorcinarene Capsule As Probed by NMR Diffusion Measurements. *Org. Lett.* **2002**, *4* (24), 4365-4368.

(40) Thomason, P. F. Spectrophotometric Determination of Lithium. *Anal. Chem.* **1956**, *28* (10), 1527-1530.

(41) Monnin, C.; Dubois, M.; Papaiconomou, N.; Simonin, J.-P. Thermodynamics of the LiCl + H2O System. *J. Chem. Eng. Data* **2002**, *47* (6), 1331-1336.

(42) Dabrowa, K.; Niedbala, P.; Jurczak, J. Anion-tunable control of thermal Z[rightward arrow]E isomerisation in basic azobenzene receptors. *Chem. Commun.* **2014**, *50* (99), 15748-15751.

(43) Zhao, Y.; Truhlar, D. G. The M06 suite of density functionals for main group thermochemistry, thermochemical kinetics, noncovalent interactions, excited states, and transition elements: two new functionals and systematic testing of four M06-class functionals and 12 other functionals. *Theor. Chem. Acc.* **2007**, *120* (1-3), 215-241.

(44) Takano, Y.; Houk, K. N. Benchmarking the Conductor-like Polarizable Continuum Model (CPCM) for Aqueous Solvation Free Energies of Neutral and Ionic Organic Molecules. *J. Chem. Theory Comput.* **2005**, *1* (1), 70-77.
